# Improved thermal preferences and a stressor index derived from modeled stream temperatures and regional taxonomic standards for freshwater macroinvertebrates of the Pacific Northwest, USA

**DOI:** 10.1016/j.ecolind.2024.111869

**Published:** 2024-03-11

**Authors:** Shannon Hubler, Jen Stamp, Sean P. Sullivan, Mark Fernandez, Chad Larson, Kate Macneale, Robert W. Wisseman, Rob Plotnikoff, Britta Bierwagen

**Affiliations:** a Oregon Department of Environmental Quality, 3150 NW 229th Ave., Hillsboro, OR 97124, USA; b Tetra Tech, Inc., Bristol, NH 03222, USA; c Rhithron Associates, Inc., 33 Fort Missoula Road, Missoula, Montana 59804, USA; d Tetra Tech, Inc., 4000 Sancar Way, Suite 200, Research Triangle Park, NC 27709, USA; e Washington State Department of Ecology, Environmental Assessment Program, Lacey, WA 98503, USA; f King County DNRP, 201 South Jackson St., Suite 5600, Seattle, WA 98104, USA; g Aquatic Biology Associates, Inc., 3490 NW Deer Run Street, Corvallis, OR 97330, USA; h Snohomish County Department of Conservation of Natural Resources, 3000 Rockefeller Avenue, Everett, WA 98201, USA; i EPA Office of Research and Development, 1200 Pennsylvania Ave., NW, Washington, DC 20460, USA

**Keywords:** Temperature, Stream, Benthic macroinvertebrate, Pacific Northwest, Thermal preference, Climate change, Tolerance

## Abstract

Benthic macroinvertebrate taxa vary in their sensitivities to water quality and habitat conditions, contributing to their extensive use as ecological indicators. As climate change and landscape alteration increasingly impact stream temperatures, interest is growing in expanding our knowledge of how macroinvertebrates are affected by current and future thermal conditions. Using samples from 3501 sites, we evaluated relationships between macroinvertebrate taxa and modeled stream temperatures across Oregon and Washington, in the U.S. Pacific Northwest. We used Maximum Weekly Maximum Temperature (MWMT) values from the NorWeST temperature dataset, which is the same metric used for numeric water temperature standards in Oregon and Washington. MWMT captures peak thermal stress, when cold-water adapted aquatic biota are closest to their upper physiological limits. For each macroinvertebrate taxon, we characterized relationships between MWMT and their distributions with three measures: 1) central thermal tendency, based on weighted average (WA) optima calculations and relative abundance data; 2) lower and upper thermal limits, based on the 10th and 90th percentiles of taxon occurrence, using presence data; and 3) thermal sensitivity curve shape, based on Generalized Additive Model (GAM) plots. We assigned 521 taxa, from species to phyla, to seven thermal preference categories, ranging from cold and warm stenotherms (narrow range) to eurythermal (wide range). Thermal sensitivity and variability within each taxonomic group were identified for establishing taxonomic targets for regional monitoring programs. We also developed the Macroinvertebrate Thermal Tolerance Index (MTTI) to represent the assemblage-level response to available thermal habitats, using WA optima and relative abundances for 324 taxa. The MTTI model had a strong relationship with modeled temperatures (R^2^ = 0.68) and a root-mean-square-error of 2.5 °C. Our work builds on previous regional and national efforts to identify thermal indicator taxa by using modeled stream network temperatures and a thermal metric that corresponds directly to regional water temperature standards. Both the taxa thermal preferences and the MTTI can be used to help identify causes of biological impairment, prioritize restoration and protection actions, and monitor assemblage-wide changes in thermal tolerance over time.

## 1. Introduction

Nearly all species have a limited range of temperatures they can tolerate while maintaining homeostasis. Shifts outside of these temperature ranges can result in the loss of suitable habitat, especially for species with narrow thermal requirements. Thus, temperature is an important factor influencing the distribution of species and patterns in biodiversity (e.g., Brown et al., 2004). Worldwide, many studies predict increasing stream temperatures due to global climate change and shifts in land use patterns (Wenger et al., 2011, Isaak et al., 2017a, May et al., 2018). Assemblage-level responses to altered thermal regimes will vary depending on taxon-specific temperature tolerances, as well as biotic interactions (Hynes, 1970, Magnuson et al., 1979, Vannote and Sweeney, 1980).

Increasing stream temperatures are likely to have detrimental effects on cold-water adapted organisms, such as endangered salmonids in the U.S. Pacific Northwest (PNW) (McCullough, 1999, Mantua et al., 2010, Beechie et al., 2012, Wu et al., 2012, Pound et al., 2021). These changes may also result in range expansions for non-native warm water species (Beechie et al., 2012, Isaak et al., 2015). Benthic macroinvertebrates (herein, macroinvertebrates) have received less attention, even though they are more diverse and more commonly assessed by freshwater biomonitoring programs (Bonada et al., 2006, Bonacina et al., 2022). Globally, many biomonitoring programs use macroinvertebrate assemblages to determine compliance with biocriteria standards and identify water quality limited streams (Rosenberg and Resh, 1993; Buss et al., 2015; Haase et al., 2004).

Improved knowledge of the thermal sensitivities of macroinvertebrate taxa has several uses. For example, the presence of cold-adapted macroinvertebrate taxa can be used to verify whether stream segments are maintaining cold-water habitat required for thermally sensitive salmonids (Maryland Department of the Environment, 2019), which are frequently the basis for temperature standards in the PNW. Anadromous salmonids have complex life cycles and most PNW populations are federally protected, making monitoring a challenge. Additionally, populations are heavily influenced by ocean conditions, complicating the ability to clearly assign impacts within the watershed to their overall status. Conversely, macroinvertebrate assemblages are largely dependent on conditions of the local watersheds. Identifying where thermally sensitive macroinvertebrate taxa thrive may also indicate the existence of microhabitats and localized refugia that might otherwise be missed by measures of temperature alone. Likewise, identifying where thermally tolerant taxa have replaced sensitive taxa may help determine causes of impairments and over time provide insights into the impacts that climate change is having on stream biota. Beyond individual taxa responses, an index conveying the thermal tolerances of the entire macroinvertebrate assemblage would provide strong evidence of whether a stream is likely to provide full aquatic life support for targeted species, such as endangered salmonids. Furthermore, an assemblage level thermal tolerance index could convey important information about stream reach conditions within the framework of causal assessment/stressor identification at sites with documented impairment (Suter et al., 2010, Norton et al., 2014).

Macroinvertebrate traits, including temperature preferences, have been published in various European countries (Graf et al., 2008, Buffagni et al., 2009, Conti et al., 2014), as well as for the conterminous U.S. (e.g., Vieira et al., 2006, Poff et al., 2006, Carlisle et al., 2007, Twardochleb et al., 2021). Some researchers have performed thermal tolerance analyses from a particular state or region (e.g., Yuan, 2006, Huff et al., 2008, Richards et al., 2013; 2018). However, most thermal preference designations were based on instantaneous temperature which represents only a narrow snapshot of the annual thermal regime, potentially underestimating thermal maximum tolerances of many taxa, and providing limited direct linkage to water quality standards.

Recent efforts have vastly increased the availability of high-resolution modeled stream temperature data in the western U.S., including the NorWeST temperature database (Isaak et al., 2017a). NorWeST used continuous data from 22,751 sites, of all disturbance levels, over a period of approximately 20 years, to provide modeled stream temperature metrics (historical and future predictions) for the entire stream network (at 1-km intervals) of the Western U.S. The NorWeST models for Oregon and Washington show high predictive accuracy to field-measured stream temperatures (R^2^ = 0.88–0.94), with relatively low error (Root Mean Square Prediction Error (RMSPE) = 0.86–1.06°C) (Isaak et al., 2017a). These high-quality estimates of stream temperatures provided an opportunity to estimate thermal preferences for many taxa across a broad geographic region and develop an assemblage-level thermal tolerance index. A benefit of this approach is the ability to include many more macroinvertebrate samples where no direct measure of temperature was collected, increasing considerably the number of samples for thermal tolerance estimates.

Our objectives were to:

1. Develop an objective approach for thermal preference assignments reflecting multiple aspects of a taxon’s thermal niche, using data encompassing a wide thermal gradient and a metric corresponding directly to regional water temperature standards.
2. Assign thermal preferences to as many macroinvertebrate taxa and scales of taxonomic resolution as possible, identify groups with varying thermal sensitivities, and set taxonomic identification targets.
3. Develop a stressor index, scaled to regional management standards, that summarizes the assemblage-level response to available thermal habitat.
4. Calculate thermal preference metrics that describe changes in the benthic community and correspond to temperature classes in established water quality standards.

## 2. Methods

### 2.1. Data compilation

#### Study area.

We compiled data from freshwater wadeable stream sites in Oregon and Washington in the PNW. Both states have similar biotic and abiotic conditions (of the 12 Level 3 ecoregions that cover the two states, six span both states; Omernik, 1987). Topography is complex and varied, ranging from flat valley bottoms, to foothills, to steep, high-elevation mountains, with high desert in the east. Climate also varies widely, with abundant rainfall west of the Cascade Mountains, and xeric conditions east of the Cascades. Hydrologic regimes are rainfall- or snowmelt-dominated, depending on elevation and aspect.

#### Modeled stream temperature.

We downloaded 1-km stream segment shapefiles with NorWeST modeled stream temperature data (Isaak et al., 2016; Isaak et al., 2017a) for seven processing units: Oregon Coast, Washington Coast, Mid-Columbia, Upper Columbia-Yakima, Kootenai/Pend Oreille/Spokane, South-Central Oregon and Middle Snake. We selected the Maximum Weekly Maximum Temperature metric (MWMT; defined as the highest seven-day moving average of the maximum daily temperatures) because it is the same metric used in temperature standards for Oregon and Washington (Sturdevant, 2008, Washington State, 2020). MWMT came in various iterations, including modeled values for each year from 1993 to 2015 and an average model value covering 1993–2011. MWMT was not available for the 58 sites in the Kootenai/Pend Oreille/Spokane processing unit, but we estimated MWMT values for this unit based on the relationship between mean August temperature and MWMT in the larger NorWeST dataset, using a Deming regression (Adcock, 1878; Deming, 1985).

#### Macroinvertebrate data.

We compiled biomonitoring samples from state agencies, federal agencies, Native American tribes, universities, cities, and counties (S1). Samples were collected between 1991 and 2019, during summer low-flow conditions, using either targeted-riffle or reach-wide collection protocols (Hayslip, 2007). The reach-wide samples were multi-habitat composites from random transects (WSDOE, 2010, U.S. EPA., 2007; U.S. EPA, 2013). Methods comparability studies by Rehn et al. (2007) and Gerth and Herlihy (2006) found that targeted-riffle and reach-wide samples were generally interchangeable when used for biomonitoring assessments. All macroinvertebrate samples had sampling areas of at least 0.74 m^2^. All data sources targeted a minimum subsample of 500 total organisms. We included only samples collected using wadeable stream protocols.

### 2.2. Data preparation

We used ArcGIS (Esri, Inc. version 10.7.1) to join macroinvertebrate sites with NorWeST 1-km stream segments. We verified sites were associated with the correct stream segment and excluded sites that could not positively be matched. We linked macroinvertebrate samples with MWMT values from the year of the macroinvertebrate sampling event where available (81 % of samples); if not available, we used the 19-year average (1993–2011), which was highly correlated with the annual value (R^2^ = 0.97).

To ensure equal representation among sites, we included only one macroinvertebrate sample per 1-km NorWeST stream segment. When more than one macroinvertebrate sample was available for a given segment, we selected the sample with the highest total taxa richness to encompass as many taxa as possible. If more than one sample had the same number of total taxa, we selected the most recent sample. We excluded samples with fewer than 150 total individuals. For samples exceeding the target of 500 total individuals, we considered randomly subsampling to 500 individuals but decided against it based on the Loess regression fit, which revealed a ‘flat’ relationship between number of total taxa and total individuals in samples with 500 or more organisms (Fig. S1-1).

To harmonize the multiple macroinvertebrate datasets, regional biologists updated names of taxa that were affected by recent changes in taxonomic nomenclature, corrected misspellings, standardized naming schemes and checked final taxa names for concordance with the current standard taxonomic effort (STE) in the PNW (Sullivan et al., 2023). We also evaluated potential source bias stemming from differences in levels of taxonomic resolution, which occurred within and across datasets. Chironomidae (Diptera), Acari (Trombidiformes and Sarcoptiformes) and Oligochaeta worms were particularly affected. For example, some datasets had mostly subfamily-level Chironomidae identifications for earlier time periods, which could potentially affect the species-, genus- and tribal-level results for Chironomidae by truncating spatial distributions and temperature gradients. We flagged taxa that were most likely affected.

Finally, we aggregated abundance data from child-taxa to parent-taxa for those identified to genus or coarser levels of taxonomic resolution, using methods described in Cuffney et al. (2007). (Child-taxa represents taxa that are more-resolved, taxonomically, while parent-taxa are less-resolved. For example, abundances from species were added to abundances of genera.) The goal was to explore thermal tolerances from species to family-level, so that results would be applicable to as many PNW bioassessment programs as possible, regardless of the taxonomic levels used. We also performed this step to identify the most sensitive taxonomic levels for detecting thermal stress. After the aggregation step, we calculated presence/absence and relative abundance of each taxon within each sample.

**Fig. 1.**
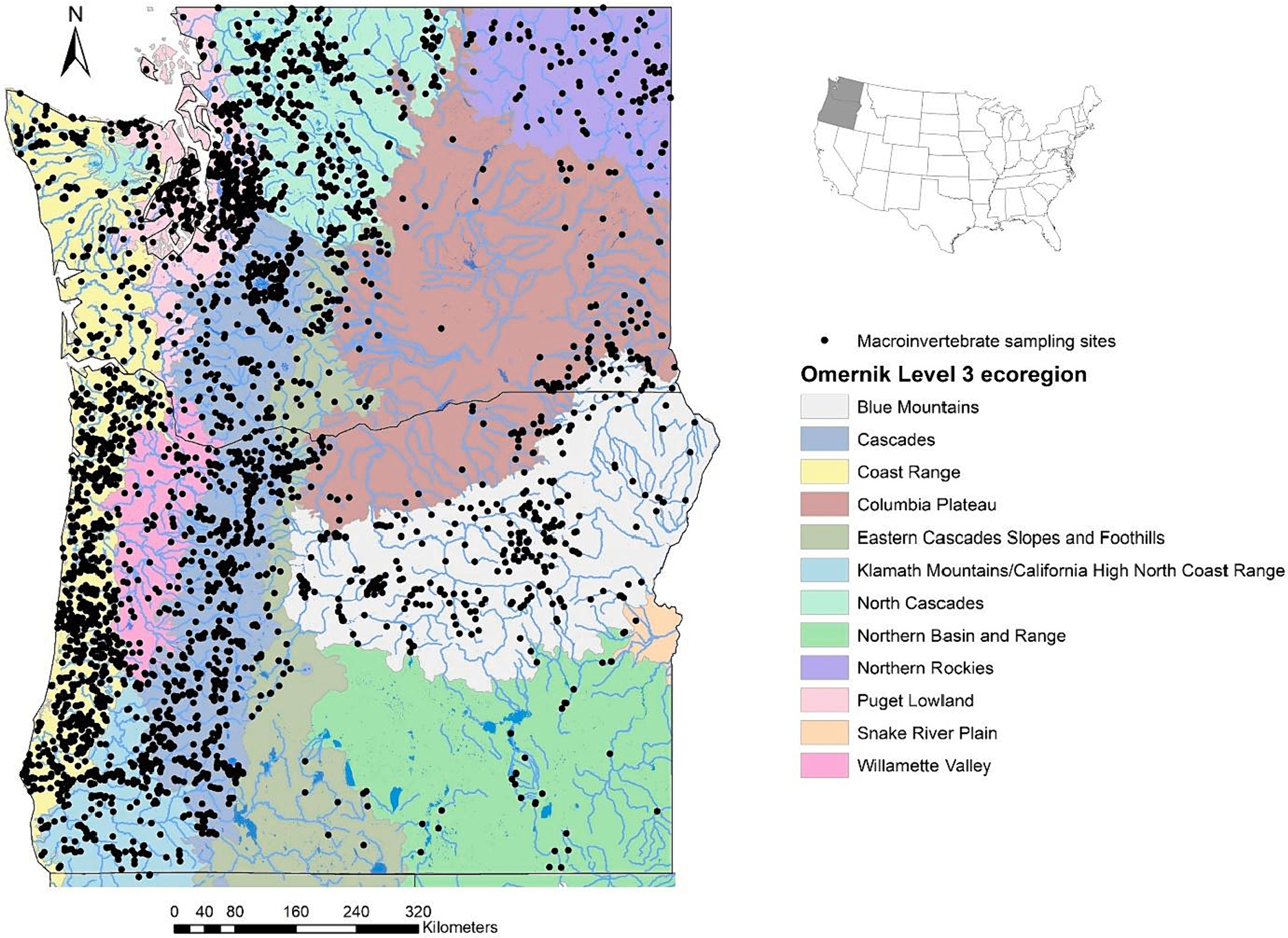
Study region and sample sites (n = 3501) shown against an Omernik Level 3 ecoregion backdrop.

The final dataset consisted of 3501 sites spanning all 12 Omernik Level 3 ecoregions in our study area (Fig. 1). Most sites were in western Oregon and Washington, with the highest percentage of sites in the Coast Range and Cascades ecoregions. Some ecoregions, in particular the eastern desert of the Columbia Plateau and Northern Basin and Range, had relatively few sites due to the xeric landscape and limited number of perennial streams. Targeted-riffle and reach-wide field methods were used for 76 % and 24 % of samples, respectively.

### 2.3. Thermal preference assignments

We characterized the stressor-response relationship between macroinvertebrate taxa and modeled stream temperature (MWMT) with three distinct thermal response metrics, each representing a different aspect of a taxon’s thermal niche: 1) central tendency; 2) lower and upper thermal limits; and 3) thermal response shape (Appendix A). We then developed quantitative rules based on the three thermal response metrics to designate each taxon into one of seven thermal preference classes. Regional biologists then reviewed these designations, with the opportunity to override them with consensus agreement on the final designation.

#### 2.3.1. Thermal response metrics

##### Thermal Optima.

Thermal optima for taxa were estimated with weighted averaging (WA, ter Braak and Looman, 1986). Optima were calculated by multiplying MWMT by taxa relative abundances (the weighting factor) for each sample, summing the resulting products, then dividing that by the sum of all the relative abundances. Values were calculated with the “analogue” R package (Simpson, 2007).

##### Thermal Limits.

Lower and upper thermal limits were calculated based on presence data. We selected the 10th and 90th percentiles to exclude potential outliers. Thermal limits helped identify taxa likely to disappear (sensitive) and those able to persist (tolerant) with increased thermal stress.

##### Response Shapes.

Generalized Additive Models (GAM; Hastie and Tibshirani, 1999) plots were used to estimate taxa probabilities of occurrence across the MWMT gradient, then subsequently to determine taxa thermal response shapes (Yuan, 2006). Plots were based on presence/absence data and fitted using the “mgcv” R package (Wood, 2004). GAMs are an extension of multiple linear regression, where the relationship between the response and predictor can be nonlinear. To aid in interpretation of results, we customized plots of GAM results (S2). First, all MWMT values were divided into 10 bins of equal temperature increments. For each taxon, the presence/absence data within a MWMT bin were averaged to calculate the probability of occurrence for that bin. (The binned data were used for visualization purposes only in the GAM plots and were not used in any other calculations.) Then, we plotted a vertical line showing the WA optima, a horizontal line showing the range of the 10th to 90th percentiles and shading to show uncertainty, as measured by 90 % confidence intervals. Following the approach used by Yuan (2006), we grouped shapes of the modeled curve lines into seven categories, as follows (Figure A1). *Decreaser*: probability of capture (POC) decreasing across the majority of the temperature gradient. *Increaser*: POC increasing across the majority of the temperature gradient. *Unimodal*: minimum values approach zero at both low and high ends of the gradient. *Uni-decreaser*: rapidly decreasing probabilities with warmer temperatures, but modestly declining probabilities at low MWMT. *Uni-increaser*: rapidly increasing probabilities with warmer temperature, but modestly declining probabilities at high MWMT. *Flat*: probabilities remained consistent across the MWMT gradient (range of POC < 0.02). Unclear: taxa with low maximum POC values (<0.15, where confidence intervals were <0.1) or that showed conflicting signs of increasing/decreasing. The automated curve shape assignments for taxa were reviewed by regional biologists and 66 were changed based on best professional judgment. These were marked with an asterisk in Appendix B.

#### 2.3.2. Thermal preferences

Taxa were assigned to one of seven thermal preferences (cold stenotherm, cold, cool, cool/warm, warm, warm stenotherm, eurythermal). Thermal preferences were linked to the numeric thresholds used in water temperature standards aimed at protecting salmonid habitat in Oregon and Washington (Table A1; Sturdevant, 2008, Washington State, 2020). Assignments were made for taxa occurring in 30 or more samples. Initial assignments were based on a combination of the thermal response metrics, per the criteria in Table 1. We developed customized R code to automate the assignments. If taxa met criteria for more than one preference, they were assigned to the last thermal preference they met, in this order: Cool-Warm, Cool, Cold, Cold Stenotherm, Warm, Warm Stenotherm, Eurythermal. Five taxa failed to meet criteria for any preference; these were initially marked as ‘inconclusive’, then assigned to one of the seven thermal preferences by regional biologists. All assignments were reviewed by regional biologists and the final designation was changed if at least three biologists agreed on the change. More detailed information on thermal preference assignments can be found in Appendix A.

#### 2.3.3. Thermal preference metrics

We used R code (R Core Team, 2022) to calculate richness and percent composition metrics for each of the seven thermal preferences (S3). We also calculated several sets of combination metrics (e.g., cold stenotherm + cold + cool). Taxa flagged for variability (Appendix C) were excluded from all thermal preference metrics. Non-distinct taxa were excluded from the richness metrics, only. We used box plots to relate thermal preference metrics to numeric thresholds used in water temperature standards established to protect salmonid habitat in Oregon and Washington (Table A1).

### 2.4. Development of the Macroinvertebrate thermal tolerance index

**Fig. 2.**
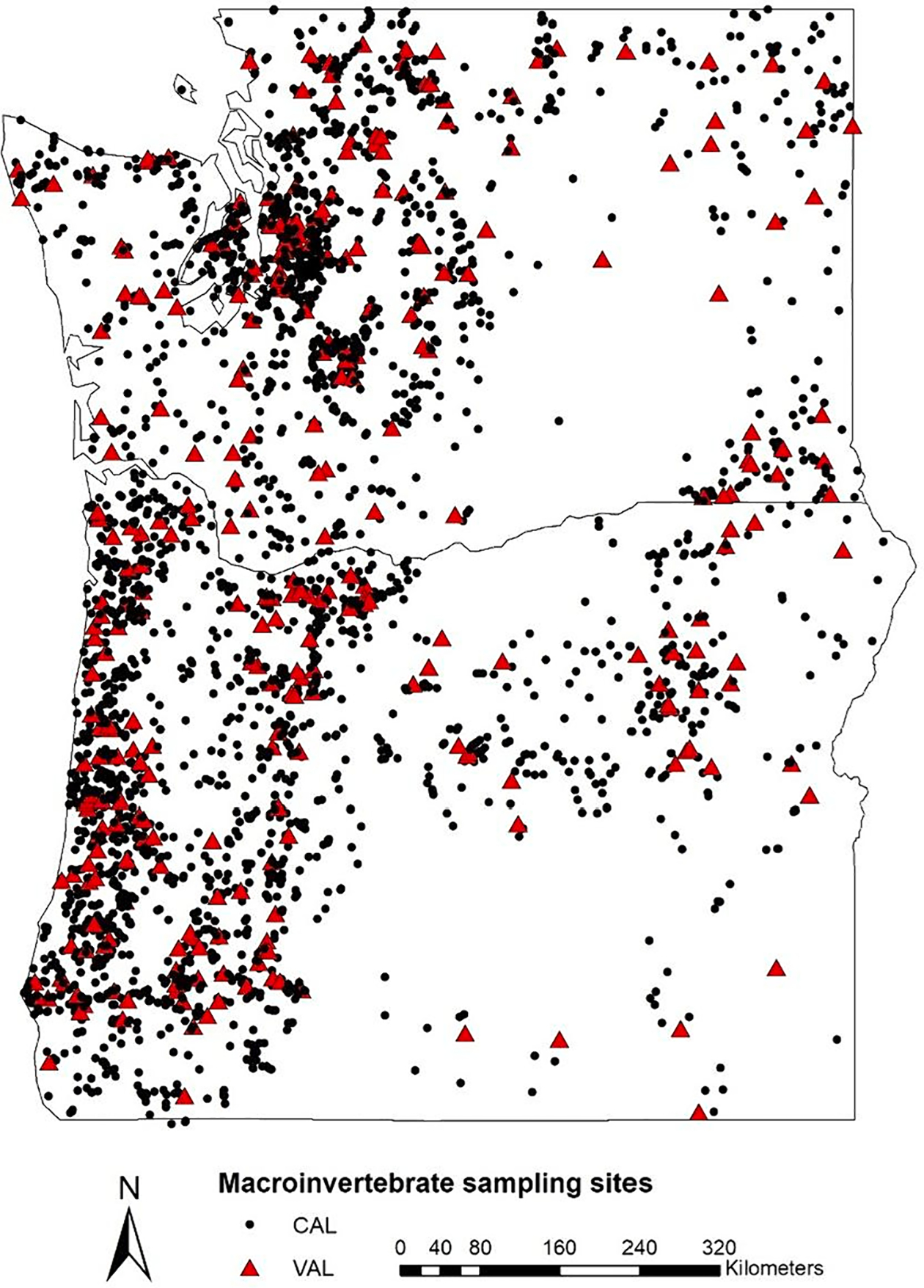
Black dots represent the calibration sites used in MTTI model development (n = 2891). Red triangles represent independent validation sites (n = 319).

The Macroinvertebrate Thermal Tolerance Index (MTTI) represents the assemblage-level thermal preference for a stream reach. The MTTI is on the same scale as MWMT (°C) and reflects the abundance-weighted mean optima of all taxa present in a sample. The first step in MTTI development was to establish operational taxonomic units (OTUs), designed to remove ambiguous taxa from the dataset (i.e., no sample has both child- and parent-taxa). Our goal in establishing OTUs was to retain as many taxa as possible—especially those children-taxa with distinct thermal sensitivities, while retaining the most information within a given taxonomic group. For each sample, all non-distinct taxa and their abundances were removed. Next, we aggregated abundances of the remaining taxa for each OTU (e.g., if an OTU was at the genus level, abundances for all children-species were combined). After aggregating all taxa into OTUs, we recalculated the total number of individuals within each sample, then dropped 291 samples with fewer than 150 individuals remaining. Next, all remaining samples were split into a calibration dataset (CAL, n = 2891) to build the MTTI and a validation dataset (VAL, n = 319) to provide an independent test of MTTI model performance (Fig. 2). To construct the VAL, 10 % of the dataset was randomly selected within each NorWeST unit-MWMT bin combination, where five temperature bins (based on quantiles) were designated independently for each NorWeST unit.

Multiple analyses were performed to ensure the VAL dataset was representative of the CAL dataset (S2). First, we associated each site with the StreamCat dataset (Hill et al., 2016), which provided catchment-level metrics for natural and disturbance gradients, as well as site-specific location metrics. With summary statistics for each metric, we qualitatively evaluated whether there were differences between CAL and VAL. This was followed with a principal components analysis (PCA, McCune and Grace, 2002) to examine multi-dimensional relationships among sites and the values of the natural and disturbance metrics. Prior to running PCA, we transformed variables as necessary to meet expectations of normality, then centered and scaled each using the R base function ‘scale’ (R Core Team, 2022). We also removed one sample where watershed area was identified as an extreme outlier (after verifying this site was indeed a wadeable stream, below a large reservoir). PCA was performed using the ‘FactoMineR’ package in R (Le et al., 2008). We used biplots to observe the degree of multivariate spatial overlap among the two datasets.

To calculate the MTTI, we used WA calibration and regression (ter Braak and van Dame, 1989). In the calibration step, optima were calculated for OTUs, as described above. In the regression step, each OTU’s thermal optimum was weighted by the OTU’s relative abundance, then these weighted values were summed across all OTUs in a sample. We explored multiple WA modeling options (S4), but ultimately settled on WA with tolerance downweighting and classical deshrinking. Tolerance downweighting (Birks et al., 1990) was used to reduce the influence of taxa with broad thermal tolerances, while deshrinking was used to spread the resulting index values back out the range of MWMT values observed in the CAL dataset. CAL model performance was assessed by the RMSEP, R^2^, and maximum bias in final MTTI predictions. We also used bootstrapped cross-validation (n = 1000) as another measure of CAL model performance. Finally, we calculated the same model performance measures for the VAL dataset. All MTTI analyses were performed using the ‘rioja’ package (Juggins, 2020) in R Statistical software (R Core Team, 2022).

## 3. Results

### 3.1. Thermal preferences

#### 3.1.1. Thermal preference assignments

**Fig. 3.**
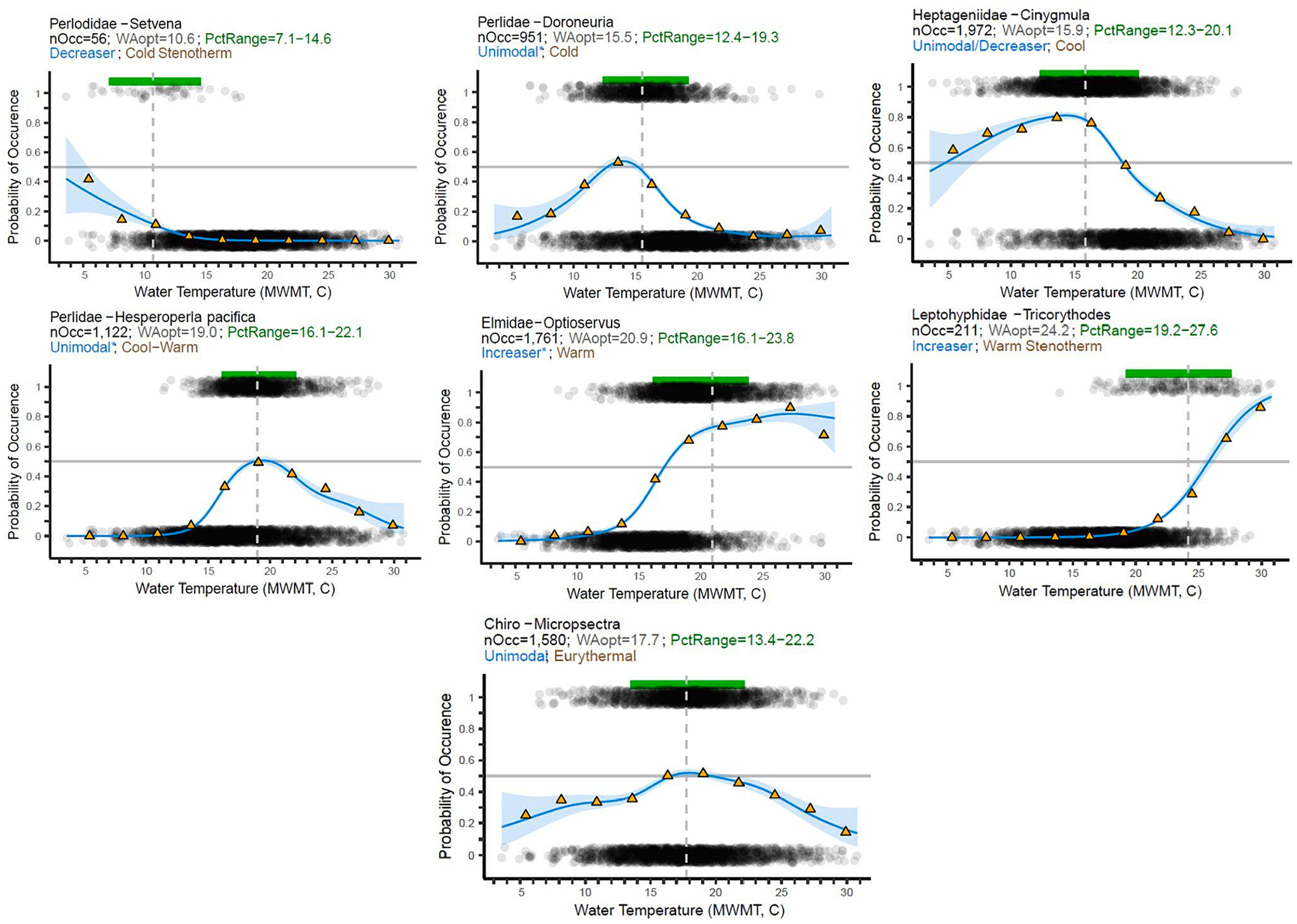
Examples of typical patterns seen in GAM plots for representative taxa from each thermal preference category. For guidance on interpretation, see Figure A1. ‘nOcc’ = number of samples where a taxon was present.

Thermal response metrics were plotted (S2) and thermal preference assignments were made for 521 macroinvertebrate taxa (Appendix B). During the review process, biologists changed initial assignments for 12 taxa, which are marked in Appendix B. We summarized the distribution of taxa across thermal preferences and listed representative taxa in each group (Table 2). Representative taxa showed strong affinities for each thermal preference (displaying response patterns like those shown in Fig. 3), were widespread and common in wadeable streams in Oregon and Washington and had levels of taxonomic resolution in concordance with the current regional STE.

Most representative taxa in the cold stenotherm, cold and cool groups were Ephemeroptera, Plecoptera, and Trichoptera (EPT), although midges (Chironomidae) and other Dipterans were well represented. For historically less-studied groups, thermal preferences were assigned to 89 genera and species of Chironomidae, 16 genera of aquatic mites (class Arachnida: subclass Acari) and 30 genera and species of other non-insects (worms, leeches, mollusks, and crustaceans). Four mite (Acari) and three worm (Oligochaeta) genera were assigned cold water status.

Results showed that finer levels of taxonomic resolution were important for achieving the best thermal sensitivity and improving chances of detecting thermal shifts (Appendix C). For example, 16 of the 17 genera that had more than one child-taxa showed variability, with *Rhyacophila*, *Epeorus* and *Baetis* each having children-taxa in four or more thermal preference categories (Table 3). Ten of the 11 tribes that had more than one child-taxon showed thermal variability, as did 27 of 29 subfamilies and 39 of 43 families.

#### 3.1.2. Thermal preference metrics

**Fig. 4.**
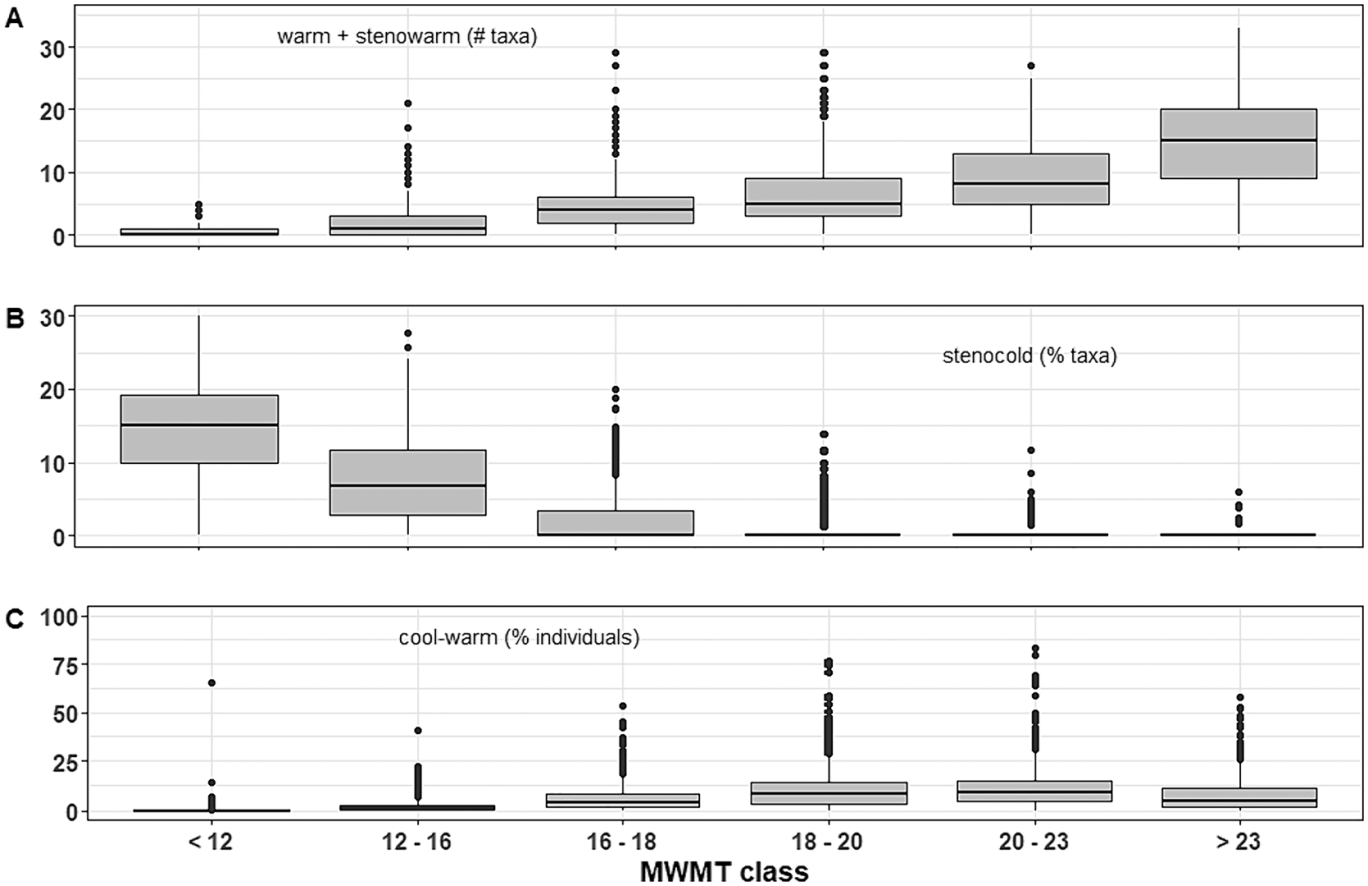
Examples of three different relationships between thermal preference metrics and MWMT classes. MWMT classes were established to match temperature standards for designated aquatic life uses in Oregon and Washington. Boxes represent interquartile range, horizontal black lines are the median, vertical black lines are non-outlier ranges, and black dots are outliers.

We generated a total of 33 different thermal preference metrics that evaluate thermal habitat support within streams (S5). We qualitatively explored the relationship between thermal preference metrics and MWMT classes that correspond with state water quality standards. Thermal preference metrics based on the most thermally sensitive or tolerant taxa showed the greatest distinction between MWMT classes. Generally, combination metrics allowed for distinguishing among most, or all, MWMT classes; metrics based on cold or warm stenotherm preferences typically distinguished among the two or three coldest or warmest classes, with zero to few taxa present in the three to four warmest or coldest classes (respectively); and cool or cool-warm preference metrics were minimally effective (Fig. 4). As expected, metrics based on eurythermal taxa were ineffective at distinguishing among MWMT classes.

### 3.2. Macroinvertebrate thermal tolerance index (MTTI)

#### Comparisons between CAL and VAL datasets.

**Fig. 5.**
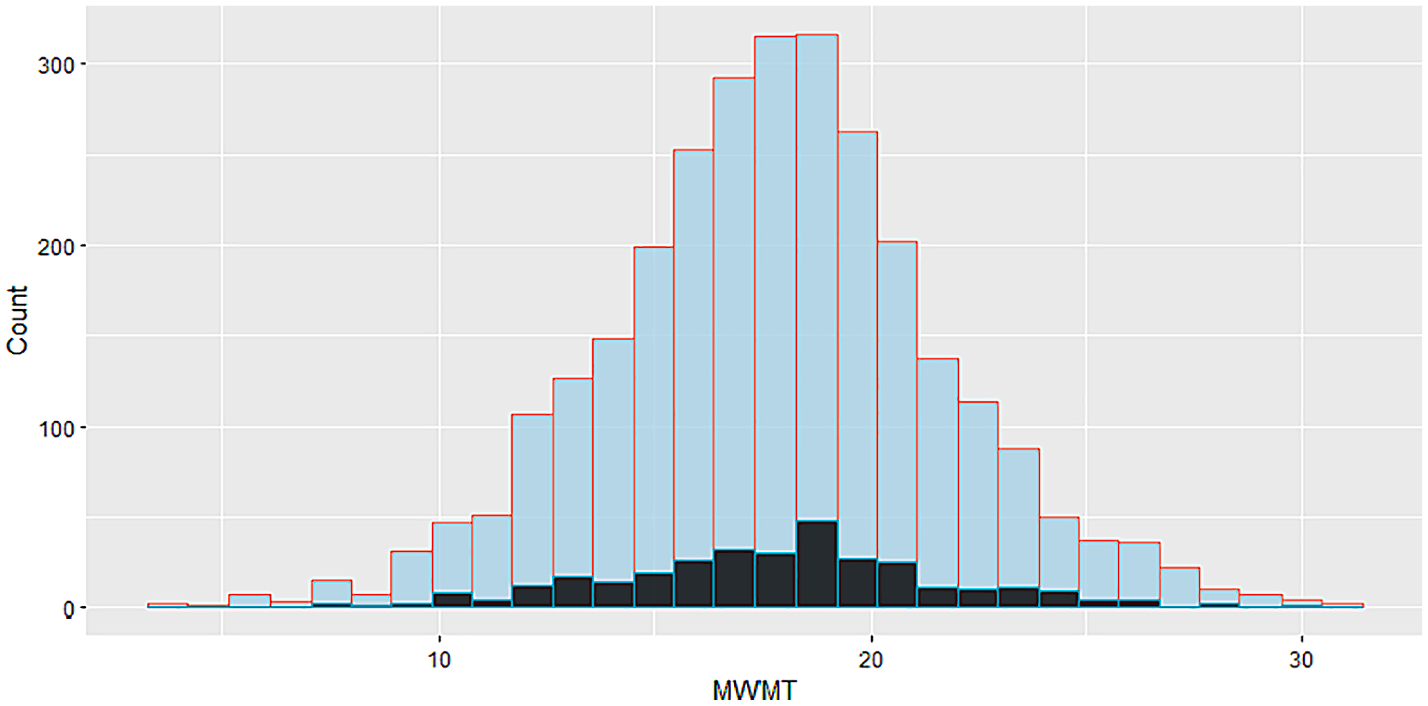
Frequencies of modeled stream temperatures (MWMT, °C) in both the calibration (blue) and validation (black) datasets.

Environmental and disturbance metrics were similar between the CAL and VAL datasets (Table S4-1, Fig. S4-1). PCA biplots showed extensive overlap in ordination space between the two datasets (Fig. S4-2). The CAL dataset had a slightly larger range in MWMT (3.6–30.8 °C) than was observed in VAL (7.5–29.7 °C). Both datasets showed low frequencies of MWMT values at the extreme ends of the thermal gradient (Fig. 5).

#### Taxonomic composition.

A total of 324 OTUs were used in the MTTI.^1^ Most OTUs were at the genus (65 %) and sub-generic (25 %) levels. Family level comprised only 6 % of OTUs, representing a mix of non-insect families and low-diversity insect families commonly associated with lentic environments. Taxonomic levels less-resolved than family accounted for 4 % of OTUs.

#### Model performance.

WA modeling showed RMSEP values were approximately 15 % lower for inverse and monotonic models (data not shown), suggesting better global model fit for these two techniques. R^2^ values were nearly identical across all models regardless of deshrinking method used; however, tolerance downweighting showed slight improvements. The greatest model differences were observed in maximum bias, where inverse and monotonic deshrinking models had maximum biases approximately 2x greater than observed in classical deshrinking models. These biases for inverse and monotonic deshrinking models were associated with the lower and upper ends of the thermal gradient. Given our objectives were to use the MTTI values as a tool for determining causes of biological impairments, where we are concerned with deviations from expected or natural conditions along the entire length of the thermal gradient, we chose tolerance downweighting and classical deshrinking for the final MTTI model.

**Fig. 6.**
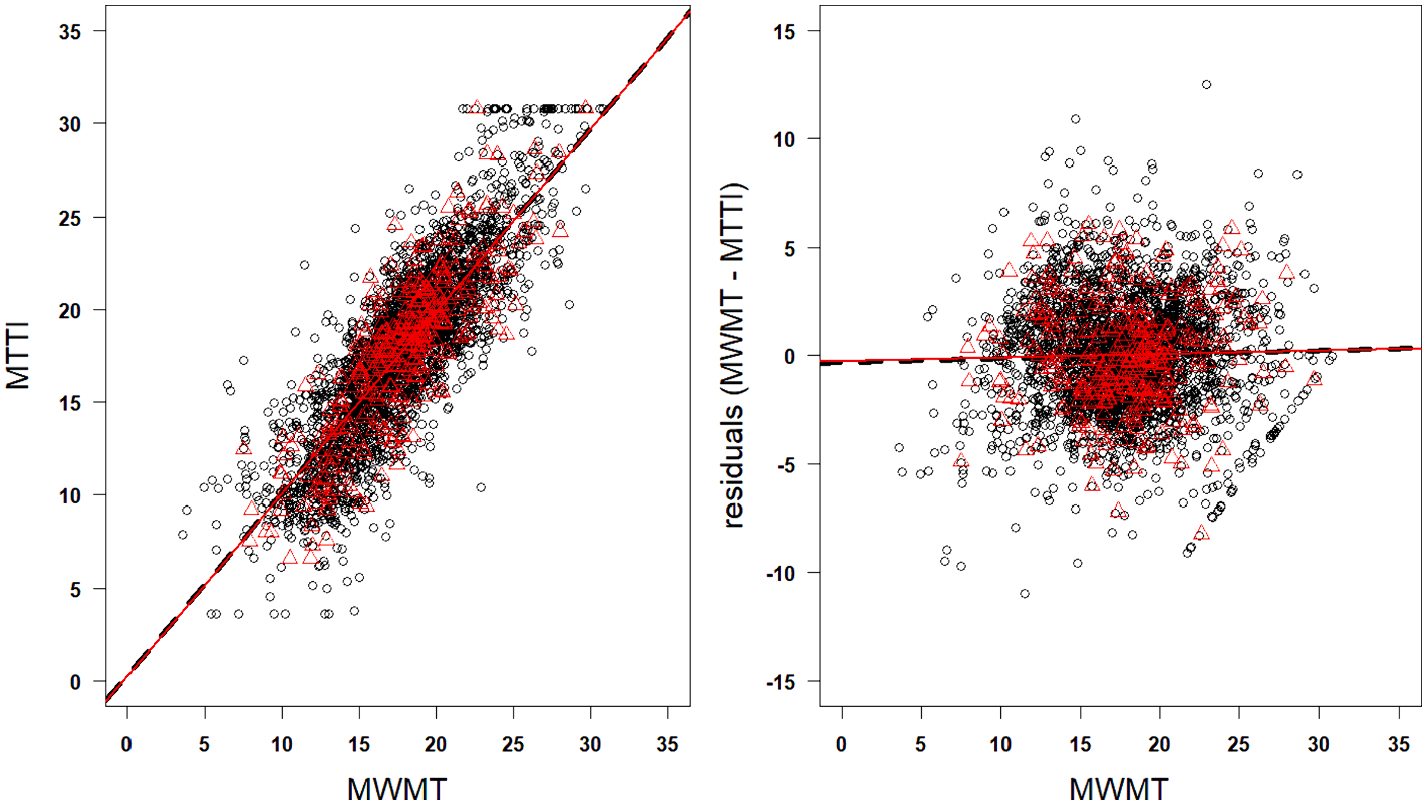
Macroinvertebrate thermal tolerance index (MTTI) vs. modeled NorWeST stream temperatures (MWMT) (left panel). MTTI residuals along the thermal gradient (right panel). Black circles = calibration (CAL) dataset, red triangles = validation (VAL) dataset, black dashed line = linear regression fit to calibration dataset, red solid line = linear regression fit to validation dataset.

Cross validation and independent validation datasets showed similar model statistics to the calibration model, suggesting the MTTI will perform consistently with novel datasets across the entire study area (Table 4, Fig. 6). RMSEP and R^2^ were consistent across all three iterations, showing a strong, positive correlation with the thermal gradient. MTTI residual errors showed no signs of bias along the modeled thermal gradient.

## 4. Discussion

Modeled stream temperatures have been used to estimate thermal tolerances for select individual aquatic vertebrates (Isaak et al., 2017b), but to our knowledge this study represents the first to use a modeled stream network to derive thermal stressor-responses for an entire assemblage. This allowed us to compile a dataset an order-of-magnitude larger than previously available datasets which relied on paired continuous temperate and macroinvertebrates. We generated thermal preferences for a considerable number of taxa, across varying taxonomic levels, and identified the most sensitive taxonomic targets. Nearly half of the thermal preferences generated in our dataset were for taxa with no prior thermal designation from other published lists. In addition, we used a novel approach for identifying preferences encompassing multiple aspects of a taxon’s thermal niche. The modeled thermal stream network also allowed us to develop an assemblage-level index of thermal tolerance with much greater spatial representativeness than previous efforts.

Making direct comparisons of thermal preferences among studies (S6) is not particularly straightforward, as complications arise from differences in thermal gradients, the thermal metrics used, and taxonomic resolution. In the U.S., national-scale thermal preferences were reported by Vieira et al. (2006), Poff et al. (2006), Carlisle et al. (2007), and Twardochleb et al. (2021). For example, the thermal tolerances in Carlisle et al. (2007) were based on a synthetic PCA gradient of both instantaneous temperature and dissolved oxygen. It is unclear whether these tolerances are directly comparable to ours. Additionally, the thermal traits in Twardochleb et al. (2021) were derived from multiple sources and studies and numerous taxa were assigned to more than one thermal preference, indicating a potential need for regional assessments when determining thermal tolerances. Moreover, the level of taxonomic resolution in the national datasets appears to be limiting, as thermal preferences have primarily been developed for genera or family levels. Our results show that even at a regional scale these taxonomic levels can show high variability in thermal preferences. Relying on less-resolved taxonomy may limit the ability to detect future shifts in the thermal regime.

### 4.1. Modeled temperatures provide equivalent tolerances and indexes to field-measured temperatures

Using modeled temperature to generate the MTTI came without a reduction in model performance, when compared to a similar index based on field temperature. We compared the MTTI and MWMT to field-measured temperatures used in Huff et al. (2008) (S2). MTTI values were highly correlated (R^2^ = 0.70) to field temperatures, as were MWMT values (R^2^ = 0.83) (Figure S4-3). The model statistics reported by Huff et al. (2008) were also remarkably similar to the MTTI model results (Table 4). Furthermore, the WA optima for shared taxa in this study and Huff et al. (2008) were highly correlated (R^2^ = 0.82; Figure S4-2). These results show the efficacy of using modeled stream network data to determine taxa preferences and developing stressor indexes, at least with comprehensive and high-quality models like NorWeST (Isaak et al., 2017a).

### 4.2. The utility of thermal preference classifications

The thermal preference assignments and WA optima for 521 macroinvertebrate taxa (Appendix B) can be linked to other databases. In addition, we provided R code (R Core Team, 2022) to calculate the MTTI and thermal preference metrics (S4) and encourage use of them in combination since they measure slightly different aspects of an assemblage’s thermal niche. If both outputs align well, this provides greater evidence for a given thermal aquatic life use designation within a stream reach. When they don’t, there is cause for further investigation.

**Fig. 7.**
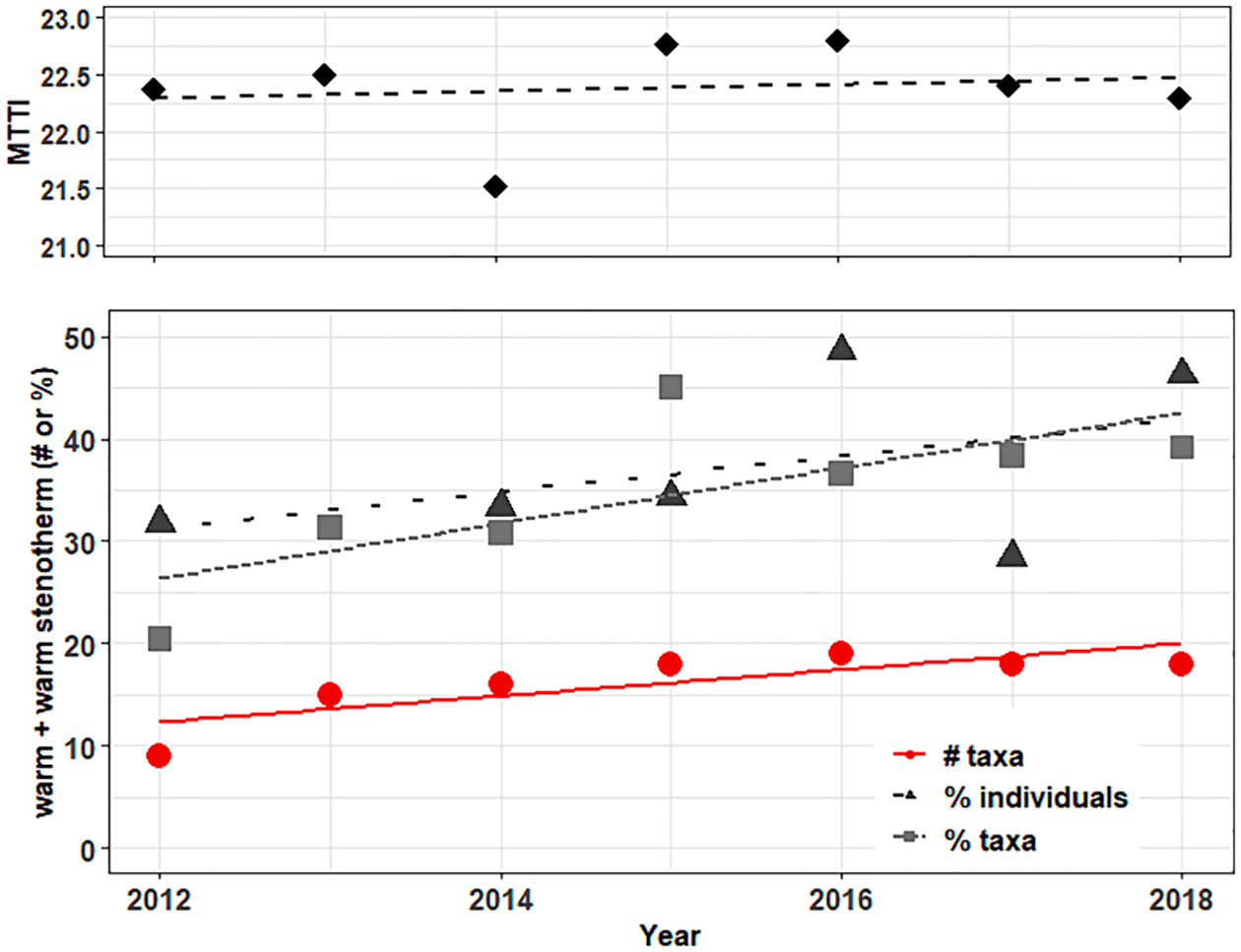
Time series plots for the MTTI and warm + warm stenotherms metrics at Bear Creek in northwestern Washington (USA). The top panel shows the MTTI, while the bottom panel shows three different metric types for the combination of warm and warm stenotherms. Lines represent linear regressions between the index/metric and sample collection year (# taxa: p < 0.05, % taxa: p < 0.1).

An example of the use of thermal metrics as an additional early screening tool is provided for Bear Creek, a highly urbanized watershed in northwestern Washington (USA) that was sampled repeatedly over the course of seven years (Fig. 7). MTTI results show a macroinvertebrate assemblage tolerant to warm water (21.5–22.8°C), with an insignificant change over the study period. Yet the thermal metrics suggest another pattern is occurring in Bear Creek. Both warm + warm stenotherm taxa richness (p < 0.05) and percent taxa (p < 0.1) significantly increased over time. That richness metrics would show a response prior to abundance metrics or the MTTI indicates that as thermal habitat changes, it may take time for (in this case) more thermally tolerant taxa to establish robust populations following colonization. With this example in context, it may be that richness based thermal metrics offer the earliest opportunities for detecting biological impacts due to rising temperatures.

### 4.3. Taxonomic resolution is important in modeling stressor-responses

The taxonomic resolution required to achieve the greatest sensitivity for detecting shifts within the thermal regime align closely with the resolution specified by the current STE for this region (Sullivan et al., 2023). Of the 521 taxa with thermal preferences in our study, most variability-flagged taxa were at the family level. Even some genera usually thought of as sensitive showed variability in children-taxa (e.g., *Rhyacophila*; Table 3). Several recent studies have projected impacts of climate change on the future composition of stream macroinvertebrate assemblages. In Spain, Kroll et al. (2017) and Alba-Tercedor et al. (2017) made predictions for macroinvertebrate families in response to projected climate scenarios. In our study, 91% of families were flagged for high thermal preference variability. Pyne and Poff (2017) make predictions of extirpation risk for taxa across a region that includes, but is much broader than, our study area. Their future climate scenarios predict near-zero extirpation risks for Chironominae (a subfamily of midges), minimal extirpations of *Baetis* and modest extirpations of *Rhyacophila*. All three of these taxa were flagged for high taxonomic variability in our study, suggesting projections are likely to vary considerably at the local scale, depending on the specific children-taxa involved.

### 4.4. Correlations with other stressors

When developing taxa preferences or a stressor index using field data, the relationships associated with the stressor of interest are potentially confounded by correlations with other stressors. For instance, temperature is negatively correlated to dissolved oxygen, due to reduced oxygen solubility as temperature increases. These relationships are associated with location in a watershed: progressing downstream, increasing flow and decreasing slopes result in increases in both temperature and fine sediments.

We examined correlations between the MTTI and six water quality parameters, fine sediments, and the Biological Sediment Tolerance Index (BSTI, Hubler et al., 2016). Correlations to water quality parameters were weak (r^2^ = 0.07–0.11) (S4). Fine sediments showed a marginally higher correlation to MTTI (r^2^ = 0.13). The strongest correlation was observed between MTTI and the BSTI (r^2^ = 0.26). The BSTI is a similar index to the MTTI, representing an assemblage-level tolerance to fine sediments. The increased correlations between the two biological indexes, compared to measured fine sediment, provides evidence that the MTTI, and other biological stressor-indexes, should be validated with additional lines of evidence before moving forward with any determinations of causes of biological impairments or beneficial use support. The use of the thermal preference metrics and sample taxa lists, alongside the MTTI, can help to strengthen conclusions.

### 4.5. Example applications

#### 4.5.1. Identifying streams suitable for sensitive or endangered fish species

There are precedents for using macroinvertebrate taxa to predict and validate suitable reach-scale thermal habitat for sensitive and endangered native species and thus the appropriate regulatory standard to apply to a stream reach. The State of Idaho (bordering both Oregon and Washington) uses macroinvertebrate taxa to identify streams supporting cold and seasonal cold-water aquatic life uses (Richards et al., 2018); while the State of Maryland (Northeastern U.S.) uses two Plecopteran taxa, which share cold stenotherm preferences with native brook trout (*Salvelinus fontinalis*), to protect cold water streams and identify potential areas for brook trout reintroductions (Maryland Department of the Environment, 2019). Similarly, a sample showing a low MTTI or multiple cold stenotherm macroinvertebrate taxa could identify stream reaches suitable for the reintroduction of fish species with similar thermal sensitivities, like the endangered bull trout (*Salvelinus confluentus*), which was previously widespread throughout the PNW (Hayes and Banish, 2017).

**Fig. 8.**
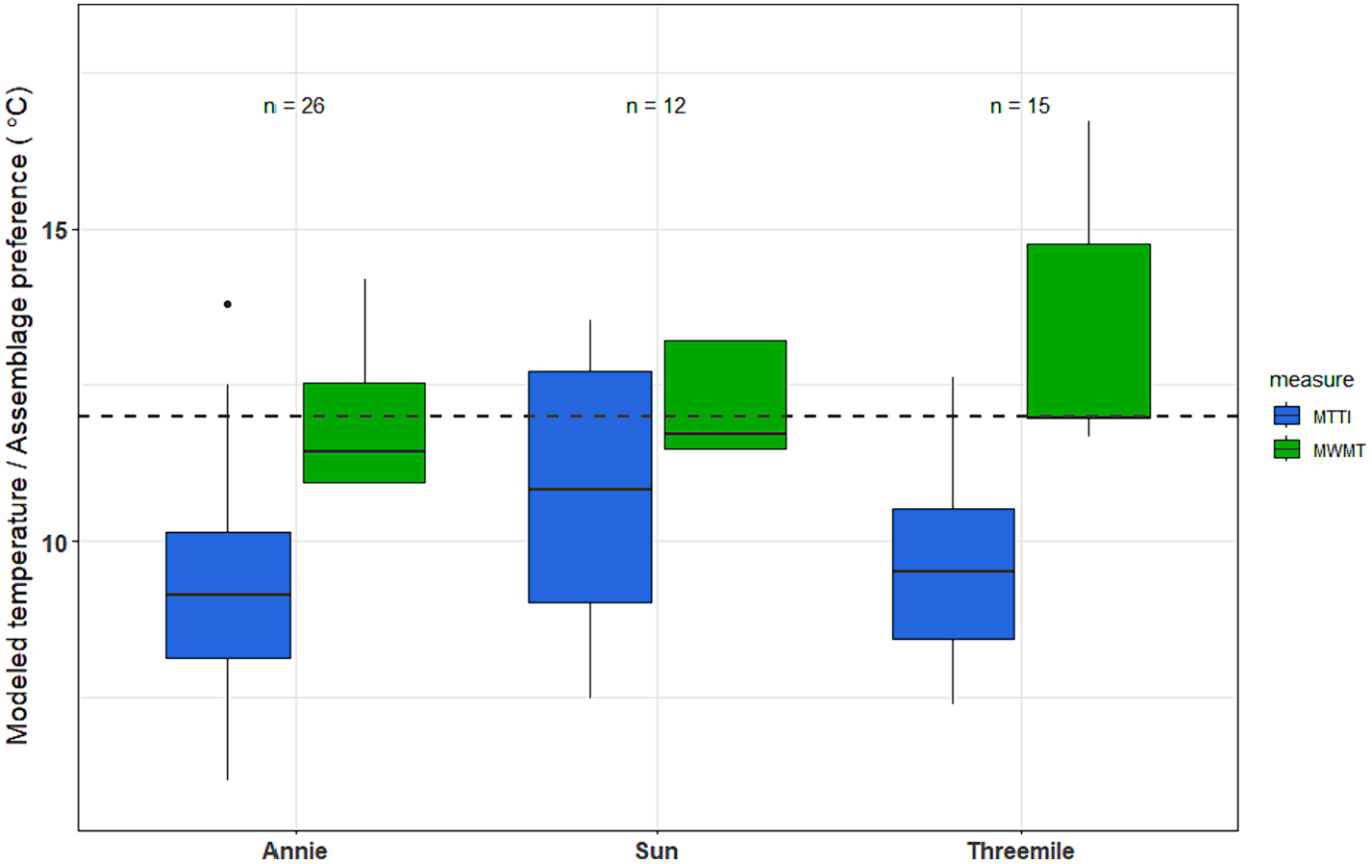
Macroinvertebrate Thermal Tolerance Index (MTTI) and modeled stream temperatures (MWMT) for three watersheds in Oregon, USA. Sun Creek and Threemile Creek currently support bull trout (*Salvelinus confluentus*) populations, while bull trout have been extirpated from Annie Creek. The horizontal dashed line represents the temperature water quality standard for ‘bull trout spawning & juvenile rearing’. Boxes represent interquartile range, horizontal black lines are the median, vertical black lines are non-outlier ranges, and black dots are outliers.

**Fig. 9.**
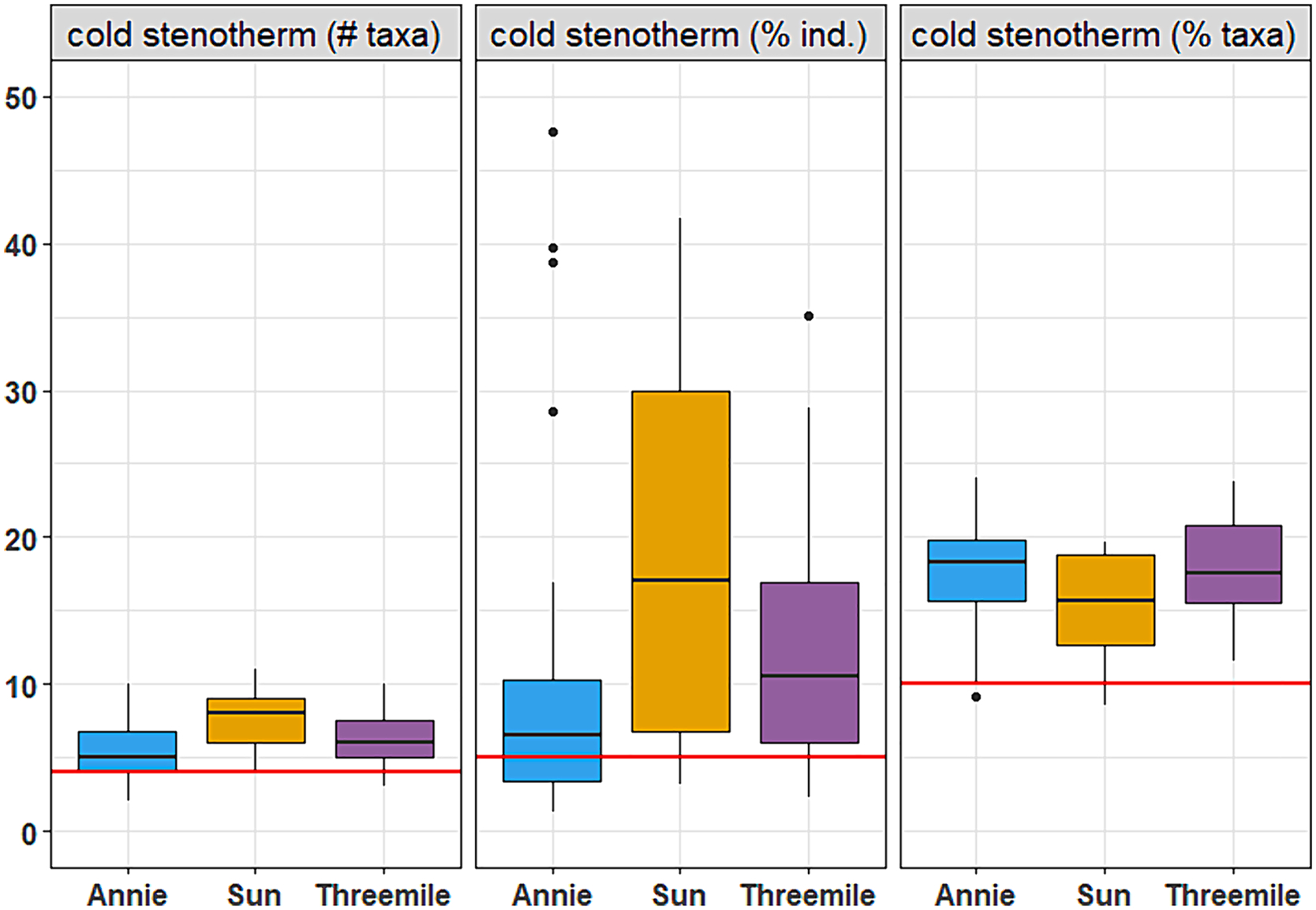
Cold stenotherm metrics for three watersheds in Oregon, USA that have current (Sun and Threemile) and historic (Annie) bull trout (*Salvelinus confluentus*) populations. Red lines represent the lower 25th percentile of cold stenotherm metrics for sites associated with MWMT values less than 12°C (the temperature standard associated with bull trout). Y-axis units are included in each boxplot header; “ind.” = individuals. Boxes represent interquartile range, horizontal black lines are the median, vertical black lines are non-outlier ranges, and black dots are outliers.

As a case study, we examined thermal preference metrics and MTTI results for three watersheds in the Upper Klamath River basin, located in south-central Oregon (USA). Two watersheds, Sun Creek and Threemile Creek, currently support populations of bull trout. A third watershed, Annie Creek, historically supported bull trout populations, but currently they are considered extirpated. Fish-use designation for these watersheds is ‘bull trout spawning & juvenile rearing’, with a temperature standard of 12.0°C. Each watershed was sampled between 12–26 times, between 2003 and 2018. MWMT values for these stream reaches suggest partial support of the designated fish use, with the majority of sites showing modeled temperatures between 11.4–14.8°C, and MWMT in the lowest site in Threemile at 16.7°C (Fig. 8). MTTI values suggest the potential for all three watersheds to support bull trout, with most samples (43 of 53) below the 12°C target. Cold stenotherm thermal metrics also show the potential to identify suitable thermal habitat for bull trout (Fig. 9). To identify potential bull trout targets for the cold stenotherm metrics, we limited the thermal preference metrics calibration dataset to only sites with MWMT values less than 12°C (n = 229), then calculated the 25th percentile. We used the 25th percentile as a lower threshold to show cold stenotherm metric values below which may suggest less support for bull trout thermal requirements. Almost all samples from Sun and Threemile fell above these potential targets, for all three cold stenotherm metrics. Annie showed slightly lower values, overall, for the number of taxa and percent individuals metrics; but most samples were above these possible targets.

#### 4.5.2. Identifying candidates for stream restoration

**Fig. 10.**
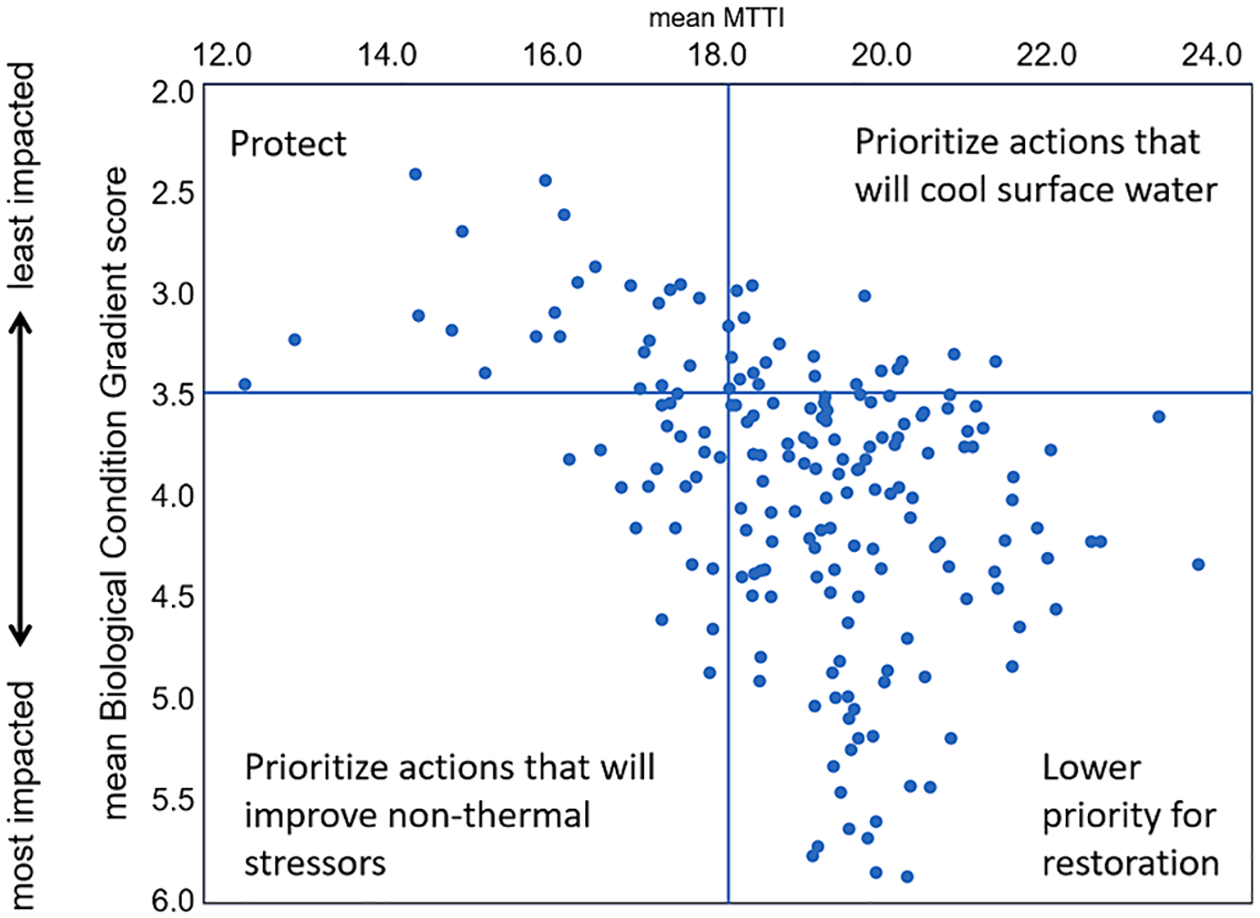
Plot of mean MTTI and mean Biological Condition Gradient scores from 151 sites in King County, Washington (USA). Sites fall within one of four quadrants, indicating if they should be prioritized for protection or restoration. Mean values are from samples collected annually from 2012 to 2022.

Another potential application is to use MTTI or thermal preference metric values to help prioritize stream reaches for restoration and protection and identify what actions may be needed. We show an example from King County, Washington (USA), where salmon recovery is a critical management priority (Good et al., 2007). Bioassessment indexes, such as the Biological Condition Gradient (Davies and Jackson, 2006), are useful for characterizing overall stream condition, but by pairing this with MTTI values we can identify where restoration actions may need to address thermal stress (Fig. 10). For example, streams could be prioritized for protection if they currently support macroinvertebrate communities indicating thermal conditions are suitable for salmonid rearing (MTTI < 18°C), and overall biological condition is good (<3.5) (Davies and Jackson, 2006, Stamp, 2022). Alternatively, streams with good overall biological condition supporting warmer macroinvertebrate communities (MTTI > 18°C) may still be candidates for thermal restoration. While there may not be a need for improved biological condition, actions that cool surface water (e.g., riparian planting to increase shading, or stormwater management to increase infiltration and limit retention in ponds) could result in reduced MTTI, showing assemblage-level shifts in thermal preferences more in alignment with fisheries management goals.

### 4.6. Future research

Biomonitoring programs worldwide have made great progress in developing tools for identifying biological impairments, yet advancements associated with identifying causes of biological impairments have lagged. Correctly identifying the causes of biological impairments is important to fully integrate bioassessment into effective watershed management. Without tools designed to identify likely causes of impairments, management actions are limited (or ineffective) if incorrect assumptions are made about the responsible stressor(s) contributing to biological impairment. To perform highly effective stressor identifications, tools must be available for a large suite of potential stressors impacting biological communities. At their root, these tools rely on taxa stressor-response relationships, either quantitative (e.g., optima) or categorical (e.g., preferences). Unlike bioassessment condition models, there appears to be few standardized approaches to developing stressor identification models.

Stressor identification, or Casual Assessment (Cormier et al., 2003), will continue to be an area of further exploration and both the MTTI and thermal preference metrics represent a step forward in evaluating temperature as a cause of biological impairment in our programs. A logical next step for integrating the MTTI and thermal preferences into water quality management in the PNW is to establish site-specific expectations using a reference condition approach (Stoddard et al., 2006). Deviations in these expected local conditions provide evidence of shifts in the macroinvertebrate assemblage due to an altered thermal regime.

Additionally, now that a large regional macroinvertebrate dataset has been compiled and harmonized, we can perform similar tolerance analyses on other stressor variables. Those analyses could supplement existing indices used for stressor identification, such as the Biological Sediment Tolerance Index (Hubler et al., 2016) and the Metals Tolerance Index (McGuire, 2009). Stressor traits could also be used to develop additional stressor indexes. For example, indexes have been developed that relate macroinvertebrate assemblage composition to pH (Hamalainen and Huttunen, 1996) and nutrients (Hilsenhoff, 1998). Evaluating stressor indexes or taxa tolerances in combination improves the ability to tease out differential effects of correlated stressors, like sediment and temperature (Yuan, 2007). In turn, this improves confidence in assigning causes of biological impairments to specific stressors. Also, assemblages other than macroinvertebrates may be better suited to certain stressors, such as diatoms for identifying excess nutrients (Pan et al., 1996, Hering et al., 2006, Ponader et al., 2007, Justus et al., 2010).

Moving forward, it will be important to continue to innovate. For example, the KLIWA Index (Sundermann et al., 2022), developed in Germany, also uses WA to determine taxa optima and ultimately an assemblage-level index of thermal tolerance. However, they use a novel approach to bin temperature values prior to calculating taxa optima, which reduces the effects of unequal sample sizes on WA (Telford and Birks 2011). The KLIWA index, after linear regression adjustments for German stream types, showed the same relationship to water temperature as observed in the (unadjusted) MTTI (R^2^ = 0.69). Future efforts in our study area should consider the methods employed by Sundermann et al. (2022) to correct for uneven sampling, or to complement existing random sampling designs that are predominantly used in the PNW by adding targeted sampling in the extremes of the stressor gradient.

Finally, as demonstrated here, it is possible to use modeled environmental gradients to develop taxa preferences or stressor indexes, with results as accurate as field collected stressor data. We hope our work inspires future development of modeled stream networks, like the NorWeST models (Isaak et al., 2017a), for additional stressors. Large-scale monitoring programs, like the U.S. EPA’s National Rivers and Streams Assessment (NRSA; Paulsen et al., 2008) that use standardized protocols across large geographic areas are likely sources of such research (Hill et al., 2017; Hill et al., 2023).

## 5. Conclusions

Trait-based approaches to monitoring environmental changes have great utility, yet often these approaches are hindered in practice by attribute tables that are incomplete for many taxa. Our analysis underscores the importance and value of collaborative research in helping to address this issue. The large size of the macroinvertebrate and temperature dataset allowed for expansion of earlier analyses and improved knowledge of taxonomic groups that have traditionally been limited by low sample sizes and coarse levels of taxonomic resolution, such as Chironomidae, aquatic mites and worms. This was made possible by agencies and organizations sharing decades of macroinvertebrate data, as well as continuous temperature sensor data, which allowed the NorWeST group to generate high-resolution modeled stream temperature data for the western U.S. (Isaak et al., 2016; 2017a). While our results are focused on Oregon and Washington, similar efforts, using similar methods, can be applied elsewhere, especially as high-resolution modeled stream temperature continues to become more readily available and macroinvertebrate data are collected in other regions. We encourage regional efforts to compile macroinvertebrate data, use advancements in modeled stream temperature data, adopt standard taxonomic efforts, refine taxa attributes, and develop stressor indices like ours. We hope this information will be used to supplement existing temperature attributes for freshwater wadeable streams and enhance monitoring of freshwater macroinvertebrate communities in the face of warming temperatures from land-use alterations and climate change.

## Supplementary Material

Supplement1

Supplement2

Supplement3

Supplement4

Supplement5

Supplement6

Supplement7

Supplement8

Supplement9

Supplement10

Supplement11

Supplement12

Supplement13

Supplement14

Supplement15

Supplement16

Supplement17

Supplement18

Supplement19

Supplement20

## Figures and Tables

**Table 1:** Rules for assigning taxa to the seven thermal preferences, using the three sets of thermal response metrics (weighted average optima, 10th/90th percentiles, and GAM thermal response shapes) in combination.

Thermal preference	MWMT (°C)			GAM thermal response shape	Indicator narrative
	Weighted average optima	Percentiles			
	(WA_opt_)	10th	90th		
Cold stenotherm	< 16	≤12	≤18	not an increaser or uni-increaser	Very cold habitat suitable for bull trout adult/sub-adult use
Cold	<18	≤14	≤20	not an increaser or uni-increaser	Suitable for salmon and steelhead rearing
Cool	<20	≤16	≤22	not an increaser or uni-increaser	Suitable for inland resident trout and cool water fish species (non-salmonid)
Cool-Warm	17.5 < WA_opt_ < 21.5	> 14	< 25	not an increaser or decreaser	Inconclusive from a management standpoint; straddle the 20 °C threshold
Warm	> 19	> 14.5	>23.5	not a decreaser or uni-decreaser	Suitable for certain indigenous warm water species
Warm stenotherm	> 23	≥19	≥26	not a decreaser or uni-decreaser	Very warm habitat
Eurythermal	–	≤14.5	≥22	–	Inconclusive from a management standpoint; ubiquitous across a wide thermal gradient

**Table 2:** Number and percent of taxa in each thermal preference. Representative taxa showed strong affinities for each category, were widespread and common in freshwater wadeable streams in Oregon and Washington and had levels of taxonomic resolution in concordance with the current regional standard taxonomic effort (Sullivan et al., 2023). Appendix B has the full list of taxa preference assignments.

Thermal preference	Number of taxa	Percent of taxa	Representative taxa
Cold stenotherm	22	4.2 %	*Megarcys, Neothremma, Parapsyche elsis, Visoka cataractae, Zapada columbiana*
Cold	61	11.7 %	*Doroneuria, Drunella coloradensis/flavilinea, Epeorus grandis group, Moselia, Yoraperla*
Cool	140	26.9 %	*Ameletus, Cinygmula, Drunella doddsii, Heterlimnius corpulentus, Sweltsa*
Cool-Warm	66	12.7 %	*Antocha, Calineuria californica, Diphetor hageni, Hesperoperla pacifica, Wormaldia*
Warm	133	25.5 %	*Ferrissia, Hemerodromia, Hydropsyche, Hydroptila, Optioservus*
Warm stenotherm	19	3.6 %	*Argia, Cheumatopsyche, Helicopsyche, Psephenus, Tricorythodes*
Eurythermal	80	15.4 %	*Baetis tricaudatus complex, Micropsectra, Neoleptophlebia/Paraleptophlebia, Parametriocnemus, Simulium*
***Total***	***521***	***100 %***	

**Table 3:** Sixteen genera were flagged for thermal variability because they had children-taxa at the species- or subgenera-level that were assigned to more than one thermal preference category. Appendix C includes comparable results for tribe-, subfamily-, and family-levels.

Genus	Thermal preference	Number of children-taxa							# Thermal preference categories	Total # children-taxa
		Cold stenotherm	Cold	Cool	Cool-warm	Warm	Warm stenotherm	Eurythermal		
*Baetis*	Eurythermal	1	1	1		2		2	5	7
*Rhyacophila*	Cool	3	4	7		1			4	15
*Epeorus*	Cool	1	1	1				1	4	4
*Eukiefferiella*	Eurythermal			1	2			3	3	6
*Zapada*	Cool	1	2	1					3	4
*Cricotopus*	Warm					3		1	2	4
*Drunella*	Cool		1	2					2	3
*Ephemerella*	Cool			2		1			2	3
*Attenella*	Cool			1	1				2	2
*Brachycentrus*	Warm					1		1	2	2
*Caudatella*	Cold	1	1						2	2
*Dicosmoecus*	Warm			1		1			2	2
*Microtendipes*	Warm				1	1			2	2
*Neophylax*	Cool			1	1				2	2
*Parapsyche*	Cold	1			1				2	2
*Pteronarcys*	Eurythermal		1			1			2	2

**Table 4:** Macroinvertebrate Thermal Tolerance Index (MTTI) modeling results. The MTTI was developed using weighted averaging with tolerance downweighting and classical deshrinking. CAL = calibration dataset, VAL = validation dataset.

Model statistic	CAL (n = 2891)	Cross validation	VAL (n = 319)
RMSEP	2.6	2.7	2.6
R^2^	0.68	0.67	0.68
Maximum Bias	2.5	2.8	1.9

## Data Availability

Data will be made available on request.

## Footnotes

1 A complete list of OTUs (ORWA_TaxaTranslator) and weighted average optima values (ORWA_Attributes) used for the MTTI calculation are available on GitHub: https://github.com/leppott/BioMonTools_SupportFiles/tree/main/data/taxa_official
