# Supplementary material for: Improved thermal preferences and a stressor index derived from modeled stream temperatures and regional taxonomic standards for freshwater macroinvertebrates of the Pacific Northwest, USA
Source: Ecol Indic. Author manuscript; Available in PMC 2025 Apr 9. (PMC11980781; doi:10.1016/j.ecolind.2024.111869)

Trichoptera

Apataniidae
nOcc=657; WAopt=16.7; PctRange=12.7−19.7
Unimodal*; Cold

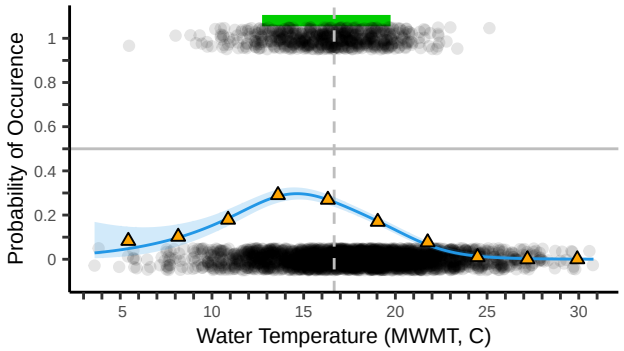

Apataniidae −Apatania
nOcc=572; WAopt=17.0; PctRange=13.2−19.9
Unimodal*; Cold

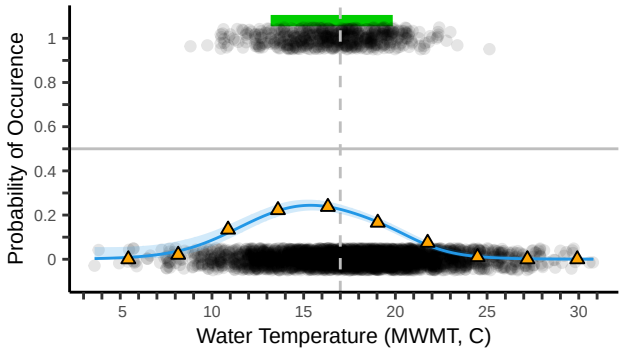

Apataniidae −Pedomoecus sierra
nOcc=94; WAopt=14.6; PctRange=12.3−17.3
Unclear; Cold

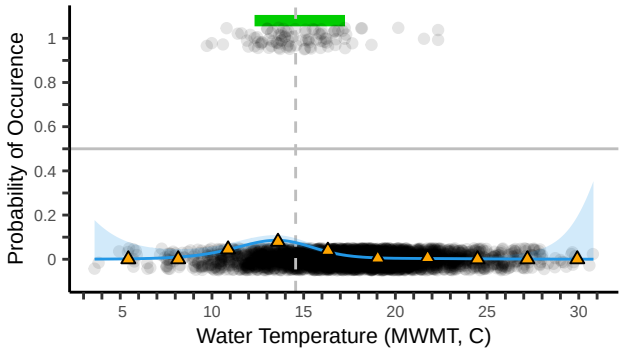

Brachycentridae
nOcc=1,932; WAopt=17.5; PctRange=13.1−21.6
Unimodal; Cool

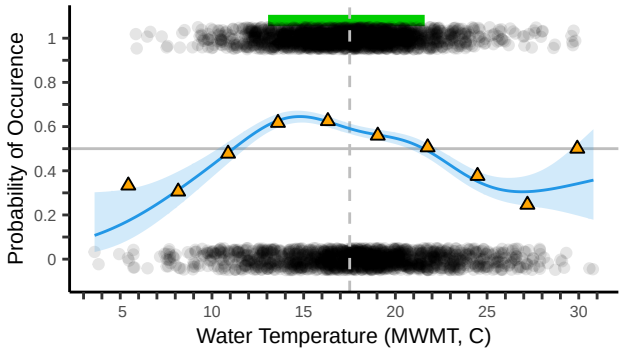

Brachycentridae −Amiocentrus
nOcc=445; WAopt=19.3; PctRange=15.1−22.4
Unimodal*; Cool−Warm

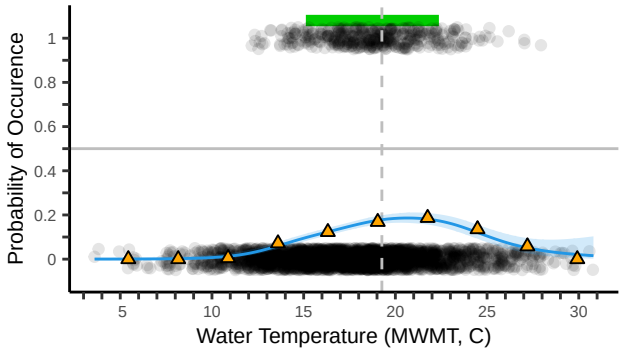

Brachycentridae −Brachycentrus
nOcc=286; WAopt=20.1; PctRange=14.9−24.2
Increaser; Warm

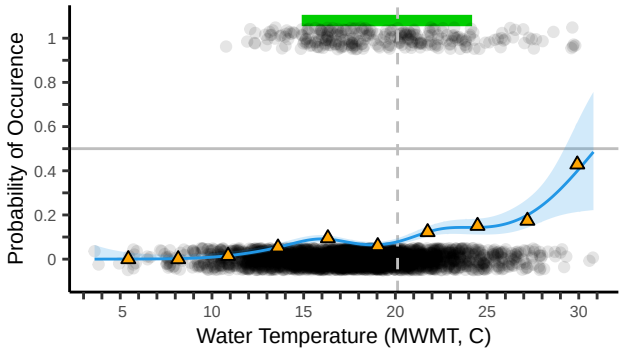

Brachycentridae −Brachycentrus americanus
nOcc=141; WAopt=18.4; PctRange=14.0−22.2
Unclear; Eurythermal

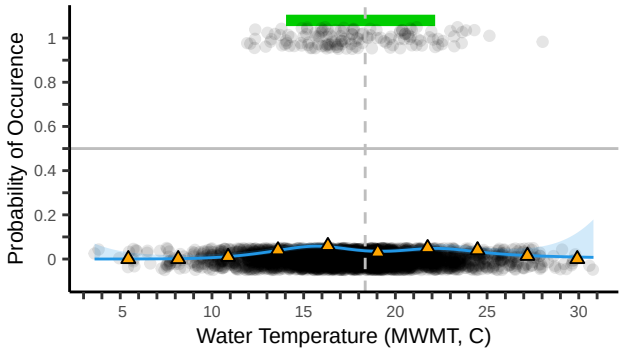

Brachycentridae −Brachycentrus occidentalis
nOcc=76; WAopt=21.3; PctRange=18.3−27.5
Increaser; Warm

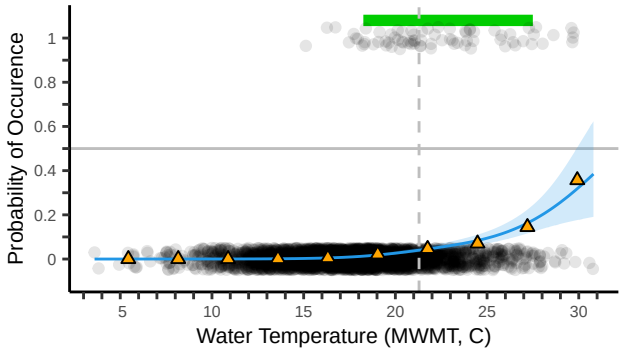

Brachycentridae −Micrasema
nOcc=1,662; WAopt=16.7; PctRange=12.8−20.8
Unimodal*; Cool

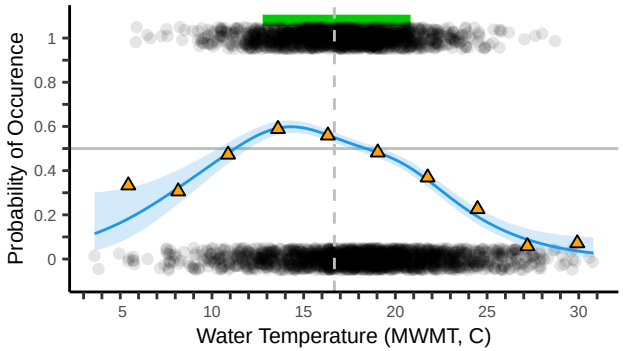

Trichoptera

Calamoceratidae −Heteroplectron californicum
nOcc=268; WAopt=18.8; PctRange=15.8−21.0
Unclear; Cool

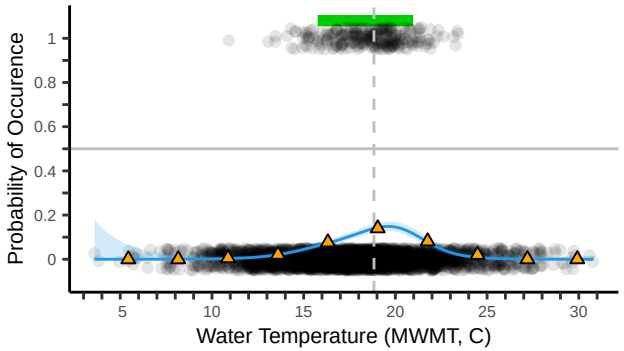

Glossosomatidae
nOcc=2,374; WAopt=17.8; PctRange=13.5−21.7
Unimodal; Cool

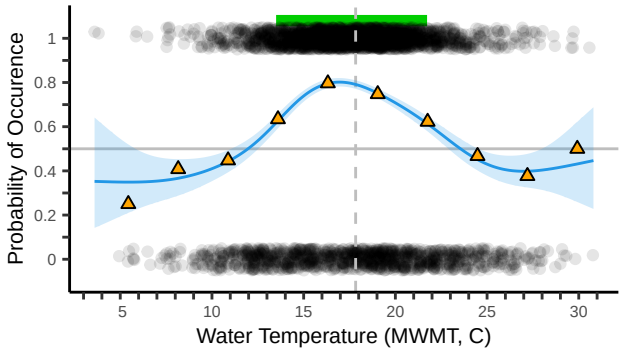

Glossosomatidae −Agapetus
nOcc=154; WAopt=19.4; PctRange=15.8−22.3
Unclear; Cool−Warm

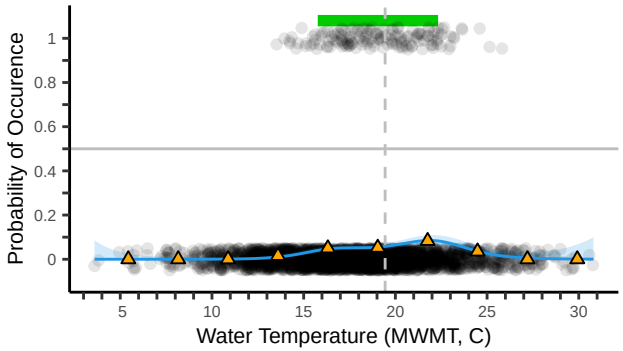

Glossosomatidae −Anagapetus
nOcc=192; WAopt=14.4; PctRange=11.1−18.5
Unimodal/Decreaser; Cold

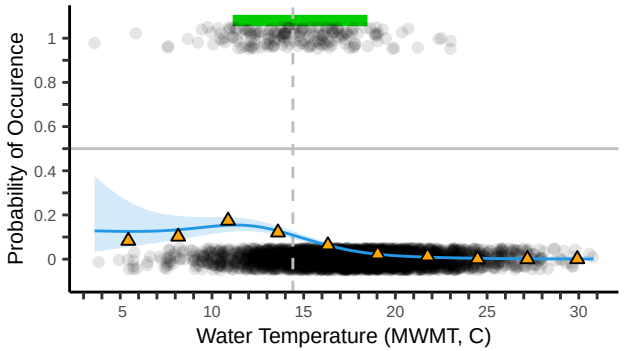

Glossosomatidae −Glossosoma
nOcc=2,010; WAopt=17.9; PctRange=14.1−21.5
Unimodal; Cool

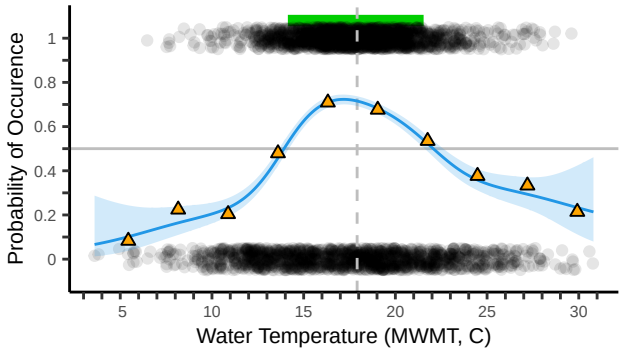

Glossosomatidae −Protoptila
nOcc=31; WAopt=23.6; PctRange=20.9−29.6
Unclear; Warm Stenotherm

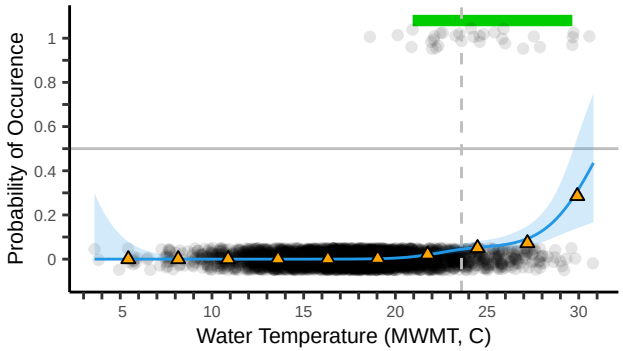

Goeridae
nOcc=97; WAopt=21.1; PctRange=17.8−23.9
Unclear; Warm

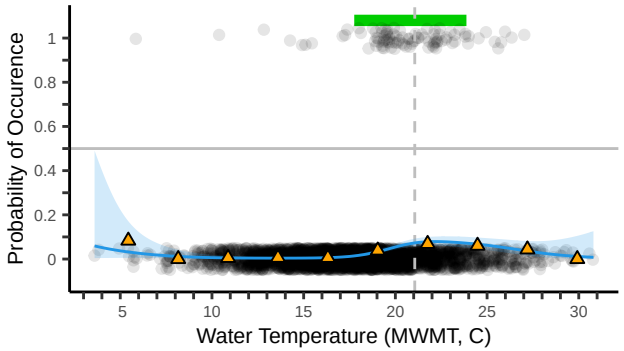

Goeridae −Goera archaon
nOcc=75; WAopt=21.9; PctRange=19.2−23.9
Unclear; Warm

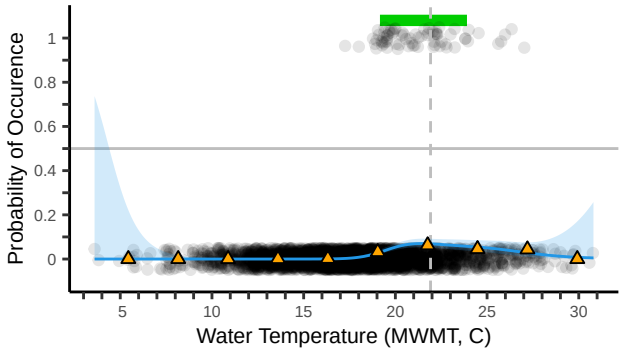

Helicopsychidae −Helicopsyche
nOcc=116; WAopt=25.5; PctRange=21.9−28.1
Increaser; Warm Stenotherm

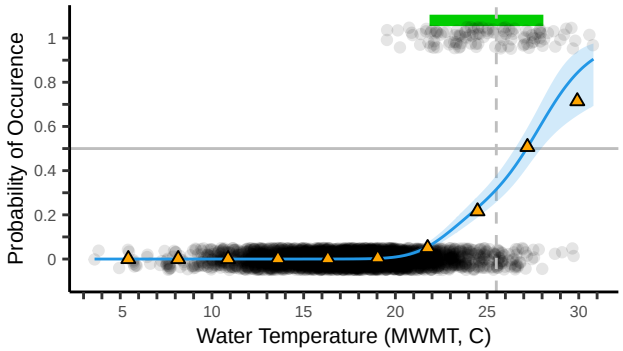

Trichoptera

Hydropsychidae
nOcc=2,886; WAopt=19.7; PctRange=13.0−22.7
Increaser; Eurythermal

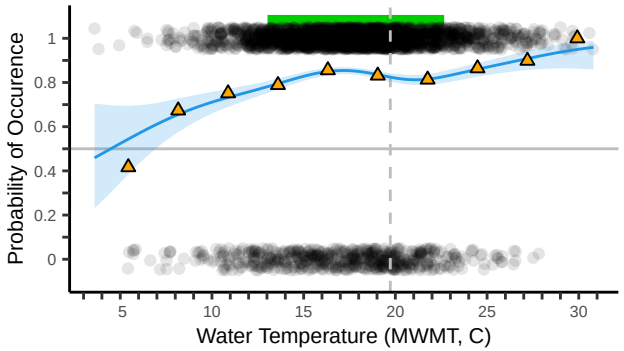

Hydropsychidae −Arctopsyche
nOcc=485; WAopt=17.0; PctRange=14.5−20.5
Unimodal*; Cool

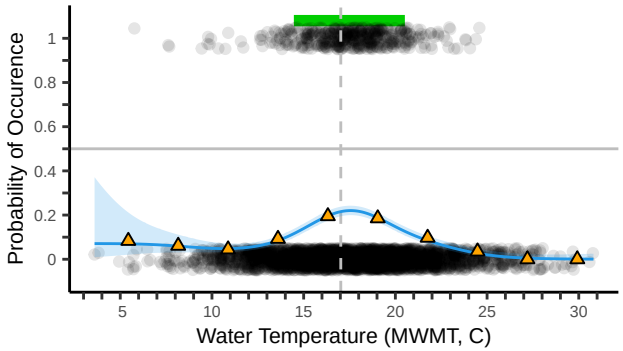

Hydropsychidae −Arctopsychinae
nOcc=1,518; WAopt=15.9; PctRange=11.9−20.0
Unimodal/Decreaser; Cool*

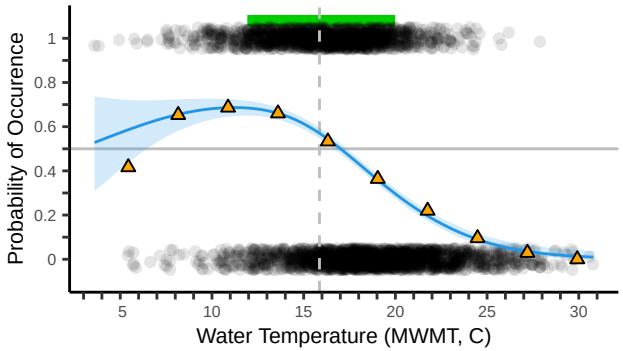

Hydropsychidae −Cheumatopsyche
nOcc=229; WAopt=24.5; PctRange=19.2−27.1
Increaser; Warm Stenotherm

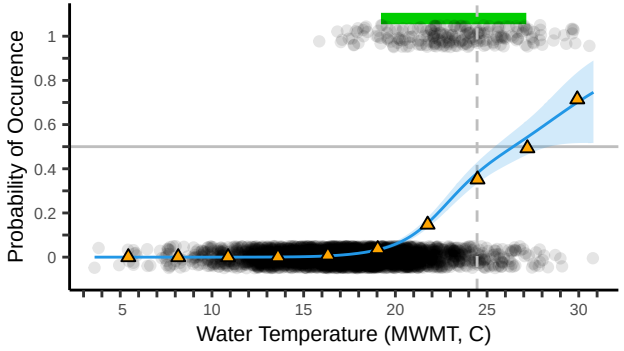

Hydropsychidae −Hydropsyche
nOcc=1,765; WAopt=20.2; PctRange=15.9−23.6
Increaser; Warm

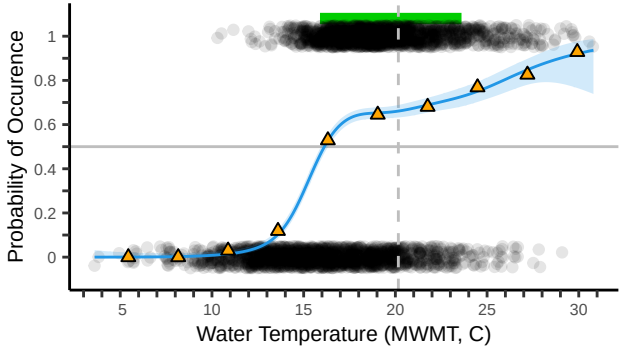

Hydropsychidae −Parapsyche
nOcc=1,103; WAopt=15.4; PctRange=11.3−19.5
Unimodal/Decreaser; Cold

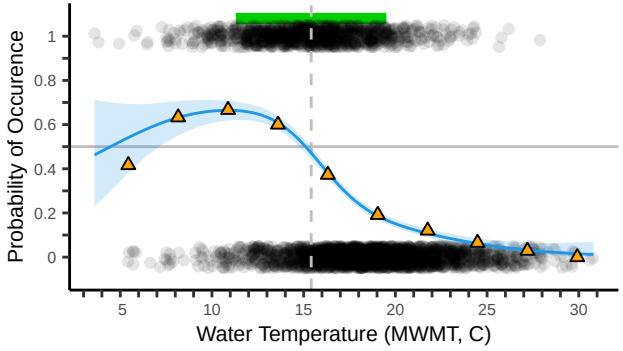

Hydropsychidae −Parapsyche almota
nOcc=158; WAopt=18.1; PctRange=14.9−22.3
Unclear; Cool−Warm

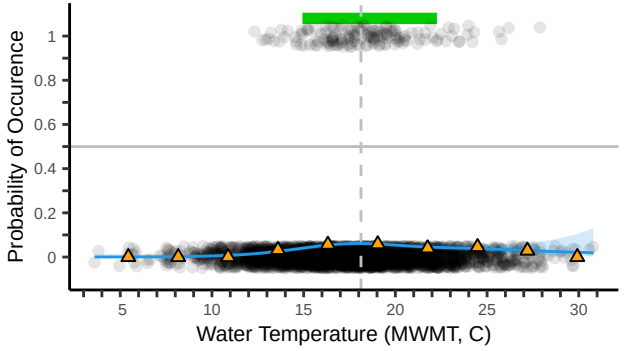

Hydropsychidae −Parapsyche elsis
nOcc=452; WAopt=13.4; PctRange=10.5−17.1
Unimodal*; Cold Stenotherm

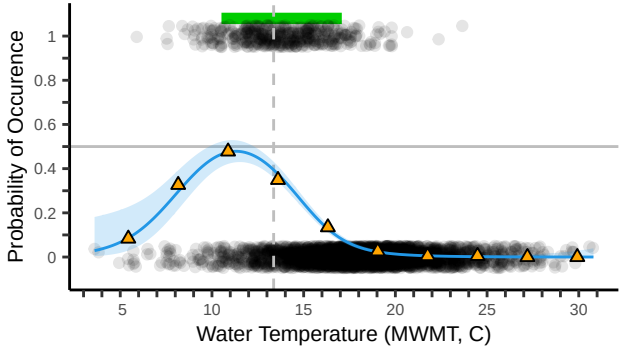

Hydroptilidae
nOcc=593; WAopt=21.0; PctRange=15.3−25.4
Increaser; Warm

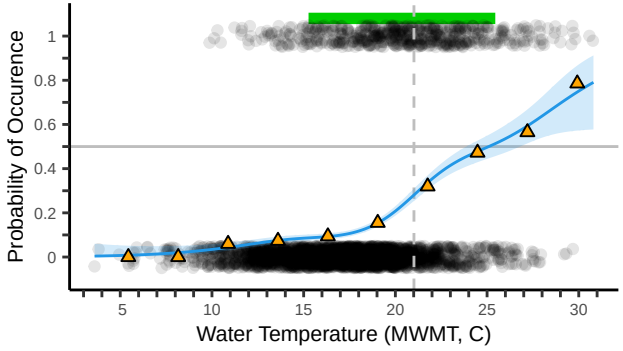

Trichoptera

Hydroptilidae −Agraylea
nOcc=38; WAopt=15.1; PctRange=12.3−18.9
Unclear; Cold

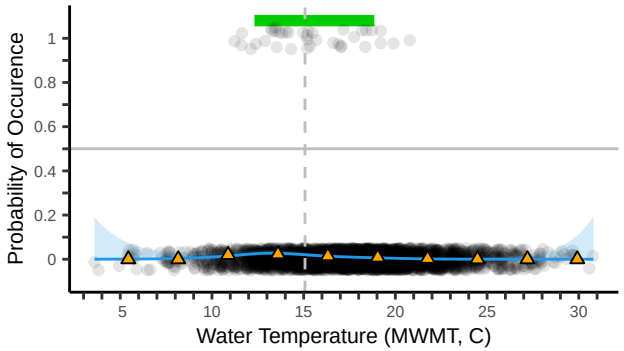

Hydroptilidae −Hydroptila
nOcc=322; WAopt=21.9; PctRange=18.0−25.9
Increaser; Warm

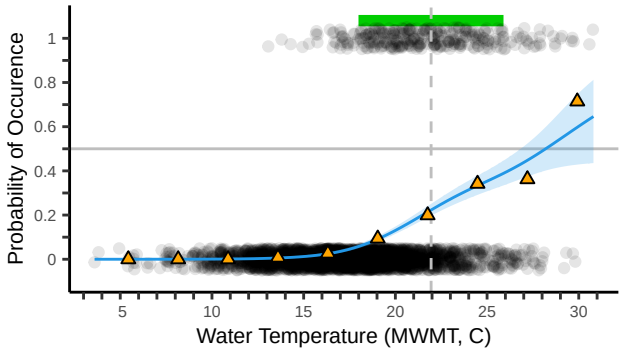

Hydroptilidae −Leucotrichia
nOcc=45; WAopt=22.5; PctRange=19.4−27.0
Unclear; Warm

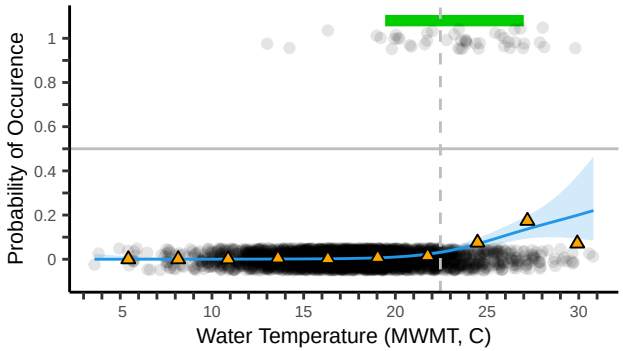

Hydroptilidae −Ochrotrichia
nOcc=51; WAopt=20.0; PctRange=16.1−27.3
Unclear; Warm

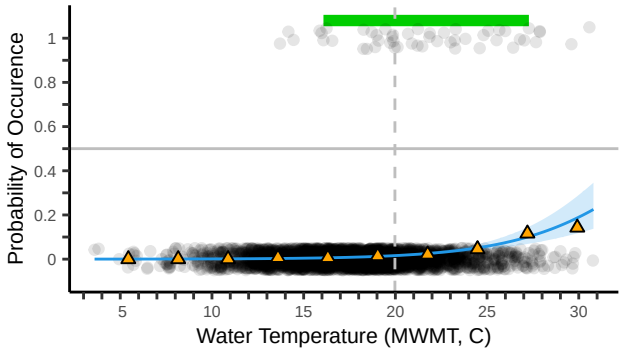

Hydroptilidae −Oxyethira
nOcc=30; WAopt=19.6; PctRange=16.7−24.1
Unclear; Warm

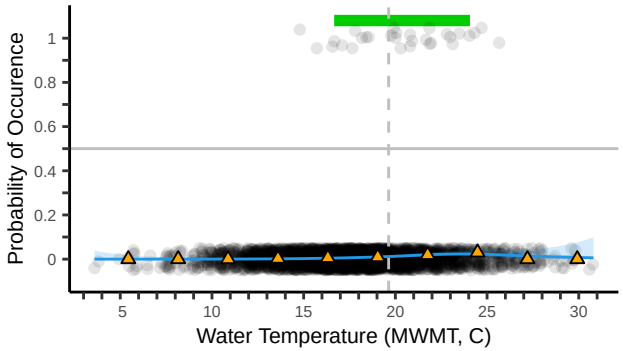

Hydroptilidae −Palaeagapetus nearcticus
nOcc=41; WAopt=16.2; PctRange=12.0−19.7
Unclear; Cold

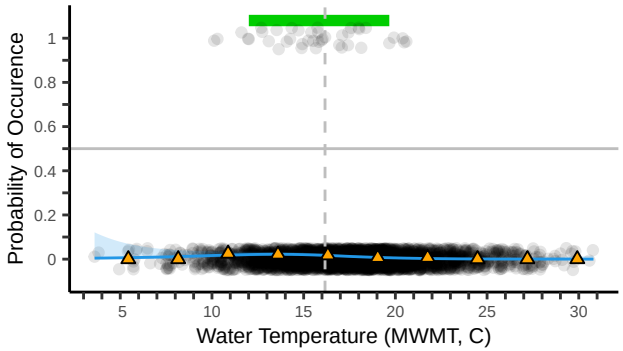

Lepidostoma
nOcc=1,914; WAopt=18.8; PctRange=13.8−22.0
Unimodal; Eurythermal

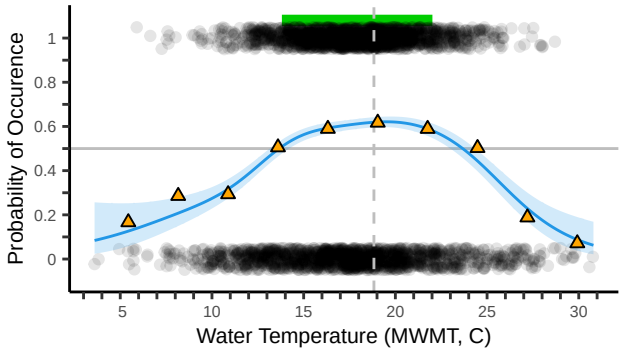

Leptoceridae
nOcc=150; WAopt=23.2; PctRange=18.9−27.3
Increaser; Warm

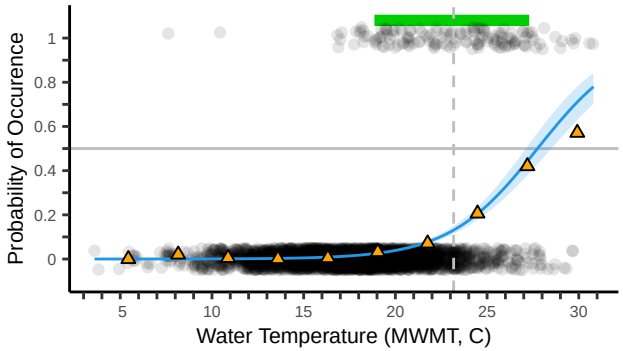

Leptoceridae −Mystacides
nOcc=41; WAopt=21.1; PctRange=18.3−24.7
Unclear; Warm

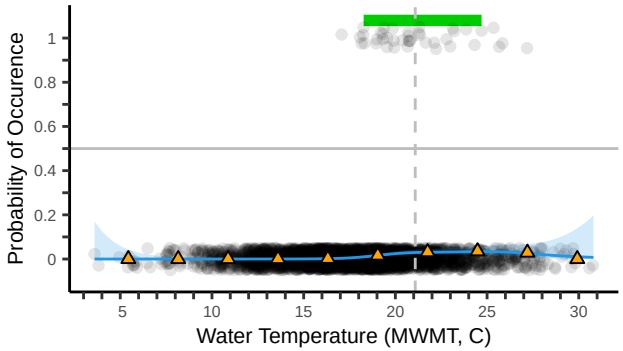

Trichoptera

Leptoceridae −Oecetis
nOcc=66; WAopt=25.8; PctRange=22.2−28.4
Increaser; Warm Stenotherm

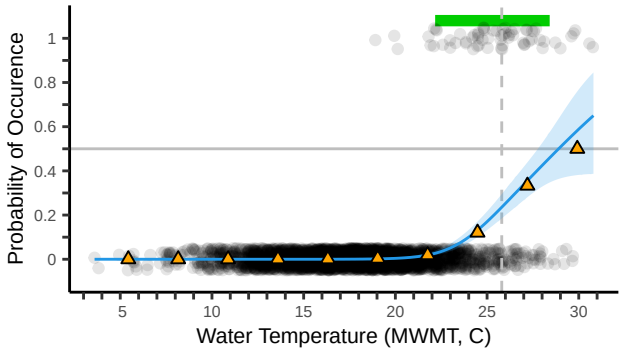

Limnephilidae
nOcc=1,683; WAopt=16.0; PctRange=12.0−21.4
Unimodal/Decreaser; Cool

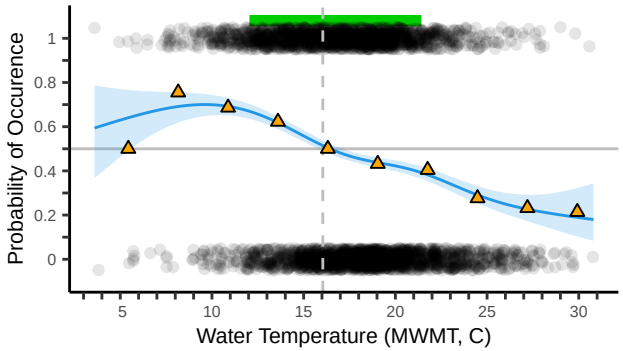

Limnephilidae−Allocosmoecus partitus
nOcc=45; WAopt=17.0; PctRange=14.6−20.5
Unclear; Cool

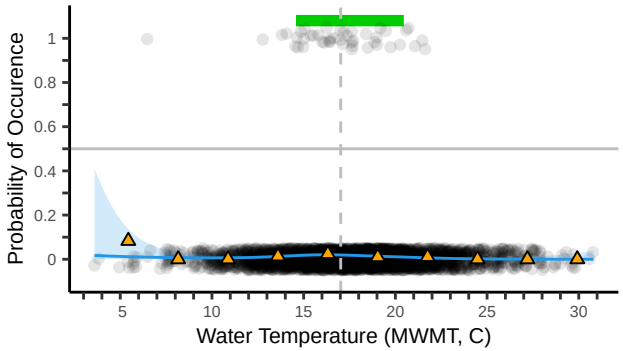

Limnephilidae−Cryptochia
nOcc=122; WAopt=13.6; PctRange=10.0−17.7
Unimodal/Decreaser; Cold*

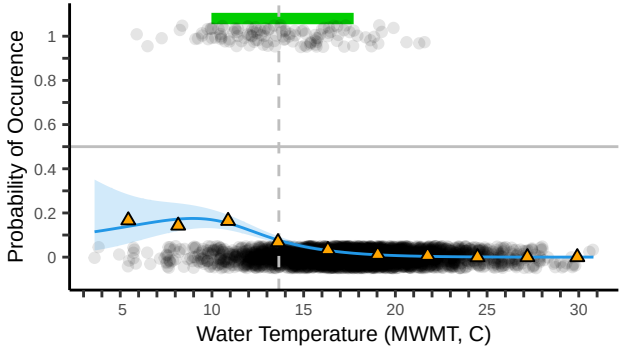

Limnephilidae−Dicosmoecinae
nOcc=880; WAopt=16.3; PctRange=11.8−22.0
Decreaser; Eurythermal

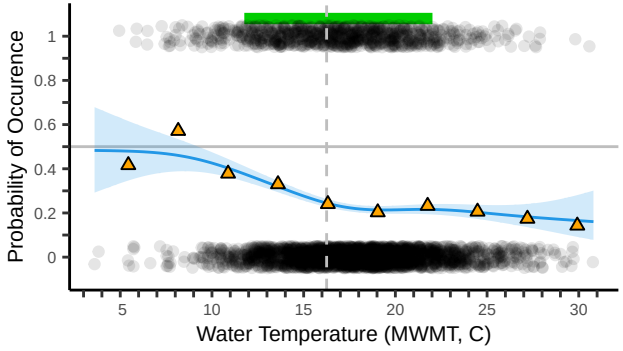

Limnephilidae−Dicosmoecus
nOcc=350; WAopt=20.0; PctRange=16.0−23.5
Unimodal/Increaser; Warm

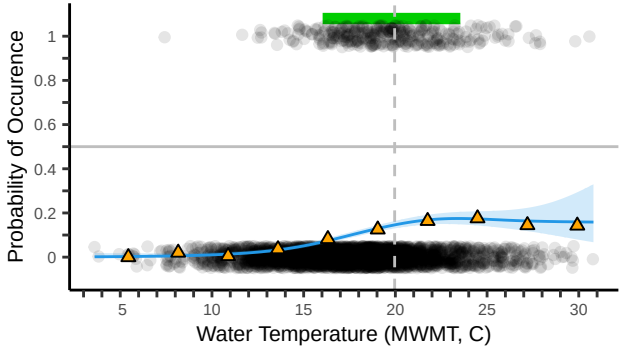

Limnephilidae−Dicosmoecus atripes
nOcc=42; WAopt=19.2; PctRange=15.1−21.4
Unclear; Cool

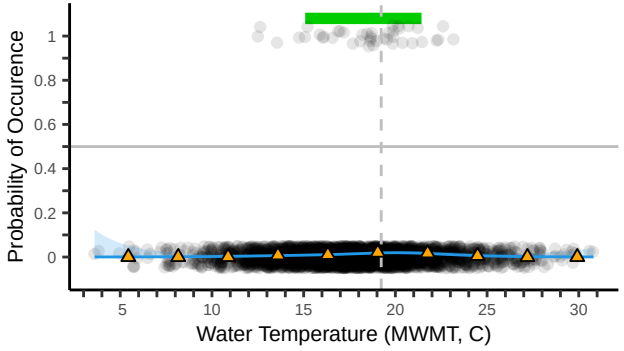

Limnephilidae−Dicosmoecus gilvipes
nOcc=213; WAopt=20.3; PctRange=16.4−24.5
Unclear; Warm

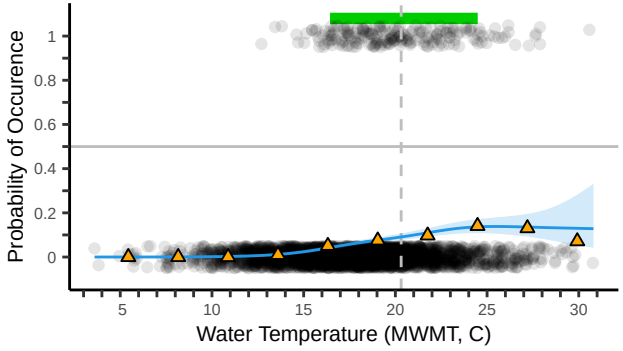

Limnephilidae−Ecclisocosmoecus scylla
nOcc=126; WAopt=14.3; PctRange=10.2−17.5
Unclear; Cold Stenotherm

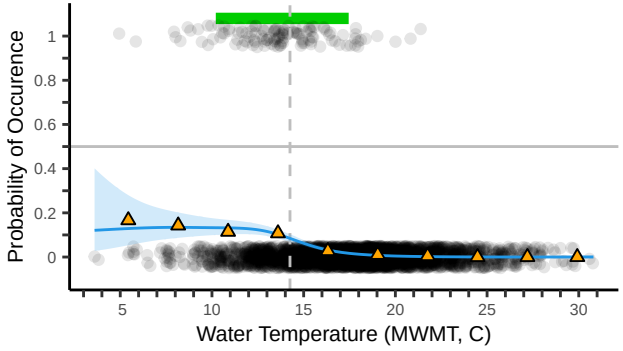

Trichoptera

Limnephilidae−Ecclisomyia
nOcc=262; WAopt=14.4; PctRange=10.5−17.8
Unimodal/Decreaser; Cold Stenotherm

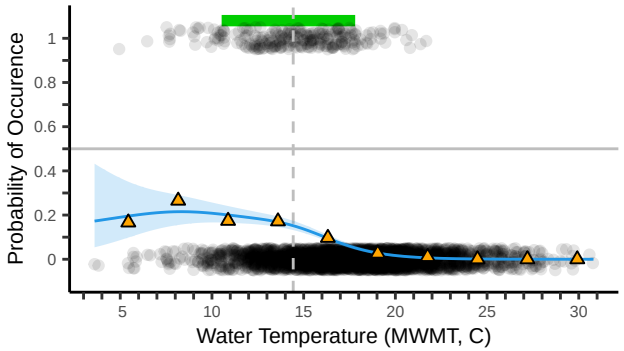

Limnephilidae−Hydatophylax
nOcc=113; WAopt=18.6; PctRange=15.8−20.3
Unclear; Cool

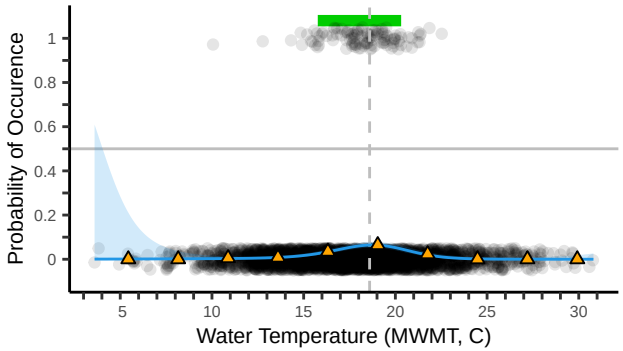

Limnephilidae−Onocosmoecus unicolor
nOcc=132; WAopt=19.7; PctRange=16.5−23.2
Unclear; Cool−Warm

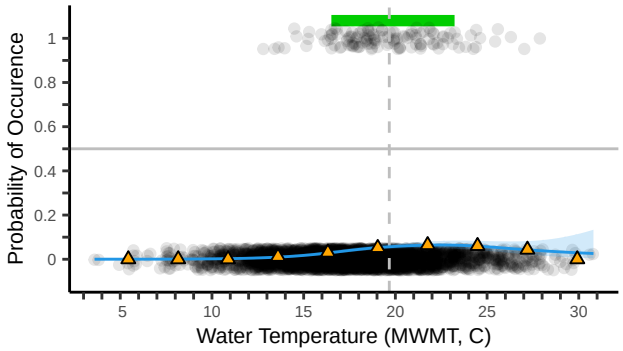

Limnephilidae−Psychoglypha
nOcc=294; WAopt=16.7; PctRange=11.4−21.2
Decreaser; Cool

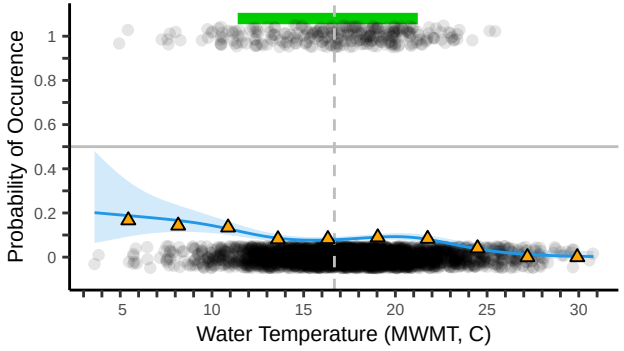

Philopotamidae
nOcc=1,444; WAopt=18.8; PctRange=14.3−22.3
Unimodal/Increaser; Eurythermal

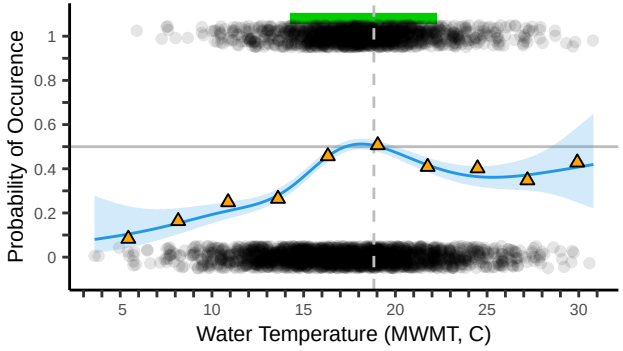

Philopotamidae−Chimarra
nOcc=37; WAopt=27.3; PctRange=20.7−29.0
Increaser; Warm Stenotherm

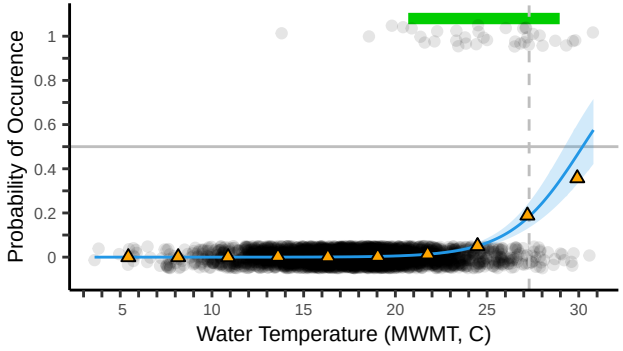

Philopotamidae−Dolophilodes
nOcc=274; WAopt=16.1; PctRange=12.0−19.7
Unclear; Cold

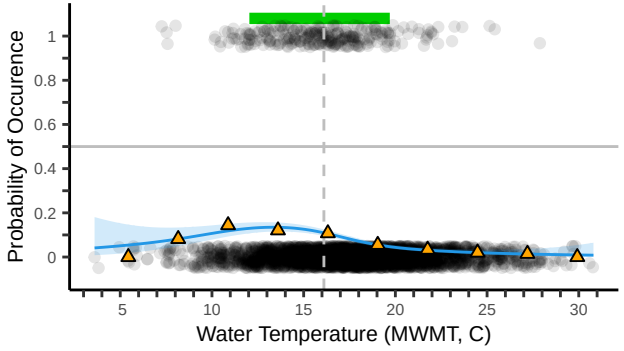

Philopotamidae−Wormaldia
nOcc=1,112; WAopt=18.8; PctRange=15.4−22.2
Unimodal*; Cool−Warm

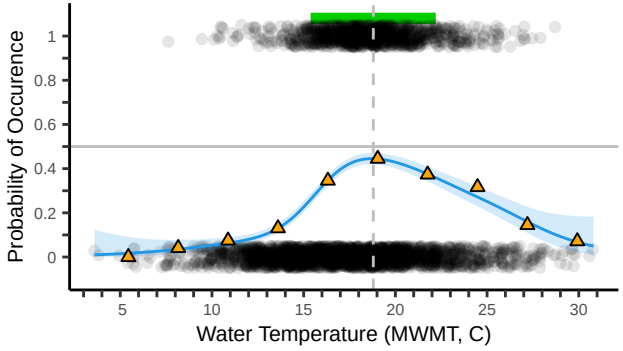

Polycentropodidae
nOcc=228; WAopt=18.3; PctRange=15.0−21.3
Unclear; Cool

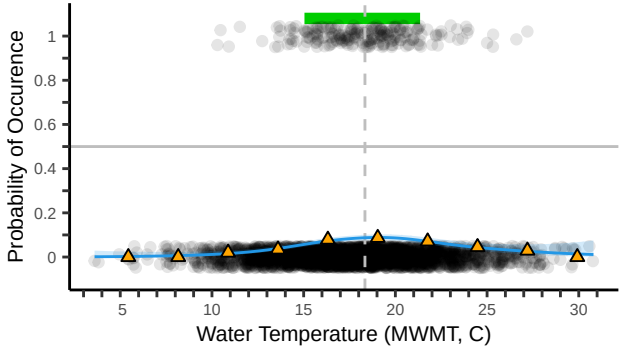

Trichoptera

Polycentropodidae −Polycentropus
nOcc=182; WAopt=18.4; PctRange=15.1−21.4
Unclear; Cool

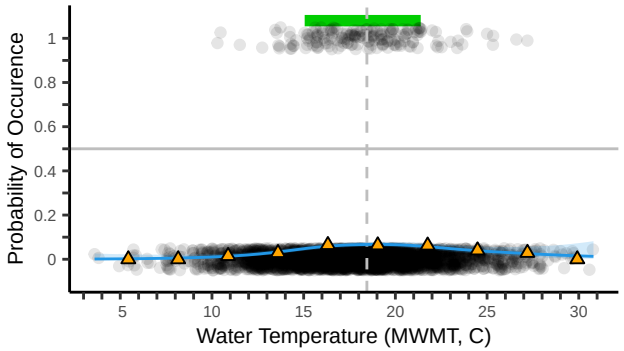

Psychomyiidae
nOcc=91; WAopt=19.8; PctRange=16.9−24.0
Unclear; Warm

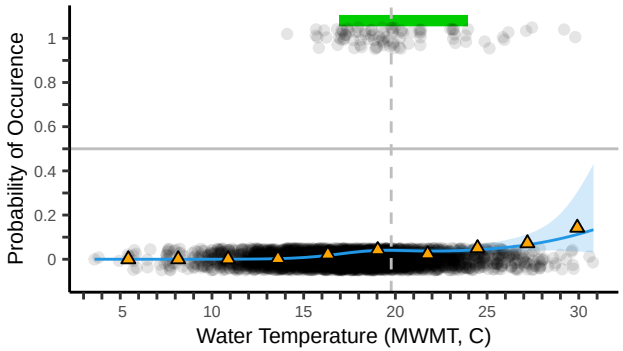

Psychomyiidae −Psychomyia
nOcc=74; WAopt=19.8; PctRange=17.0−23.7
Unclear; Warm

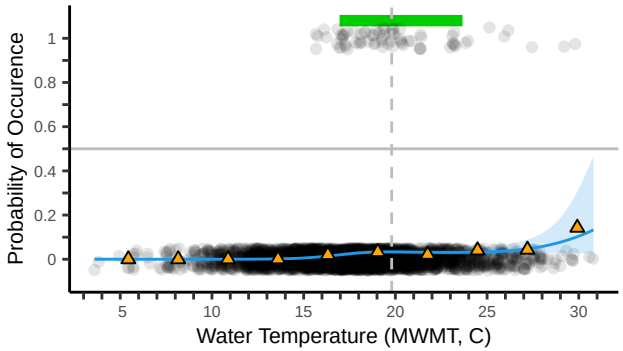

Rhyacophilidae −Rhyacophila
nOcc=3,019; WAopt=16.4; PctRange=12.5−21.1
Decreaser; Cool

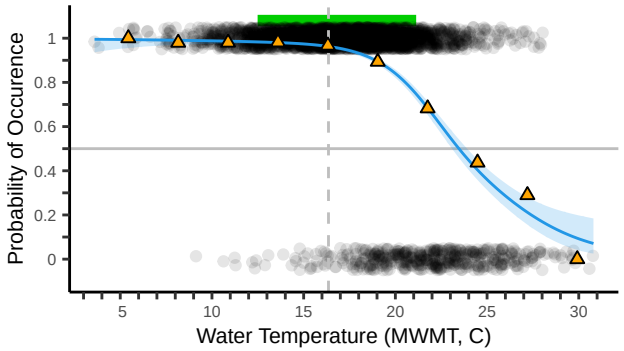

Rhyacophilidae −Rhyacophila alberta group
nOcc=79; WAopt=15.0; PctRange=12.0−18.8
Unclear; Cold

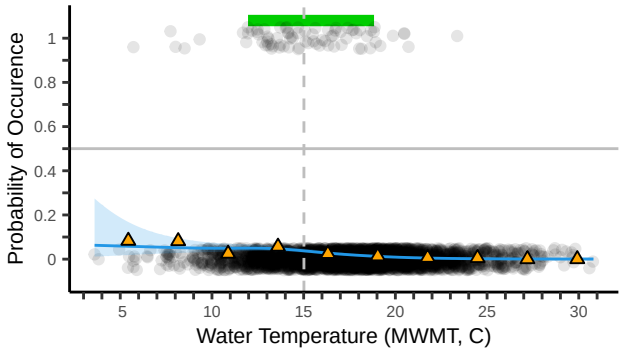

Rhyacophilidae −Rhyacophila angelita group
nOcc=331; WAopt=17.6; PctRange=14.4−20.6
Unclear; Cool

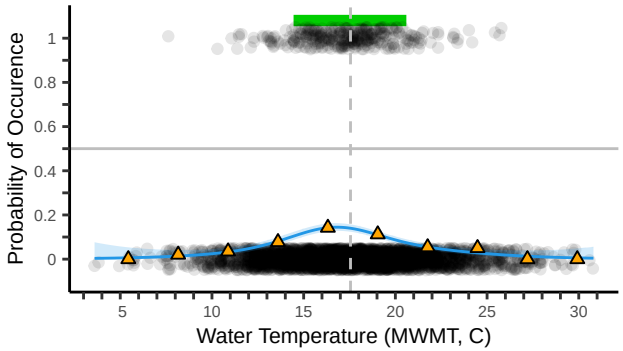

Rhyacophilidae −Rhyacophila arnaudi
nOcc=282; WAopt=18.1; PctRange=14.9−21.0
Unclear; Cool

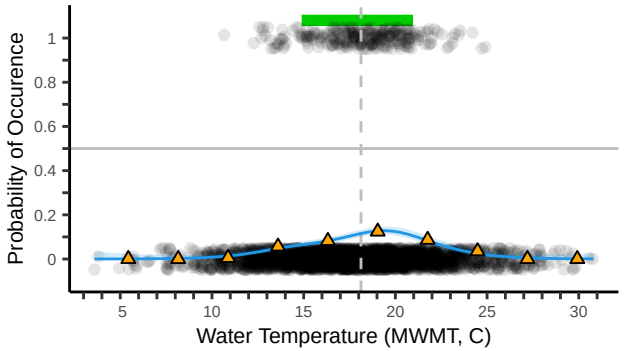

Rhyacophilidae −Rhyacophila atrata complex
nOcc=349; WAopt=14.5; PctRange=11.4−18.9
Unimodal/Decreaser; Cold

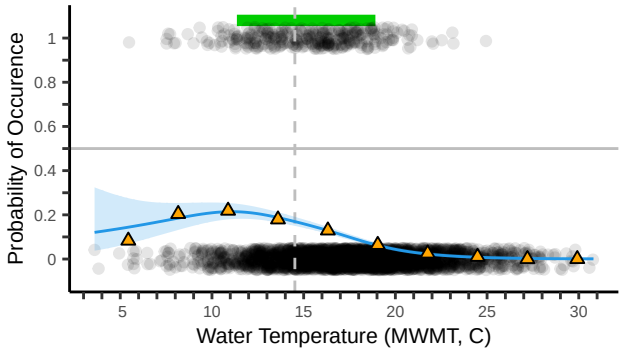

Rhyacophilidae −Rhyacophila betteni group
nOcc=1,963; WAopt=16.4; PctRange=12.6−20.5
Unimodal*; Cool

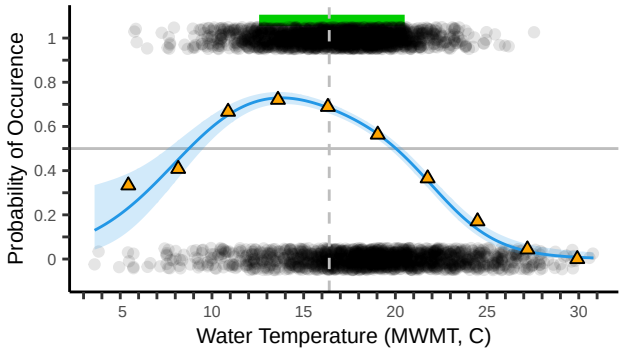

Rhyacophilidae −Rhyacophila blarina
nOcc=513; WAopt=18.6; PctRange=16.0−20.9
Unimodal; Cool

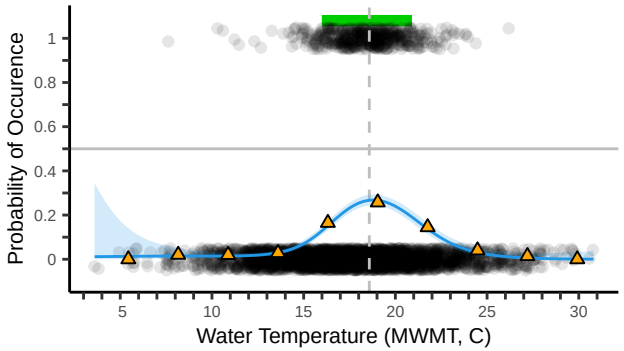

Rhyacophilidae −Rhyacophila brunnea/vemna groups
nOcc=1,734; WAopt=16.5; PctRange=12.5−20.5
Unimodal/Decreaser; Cool

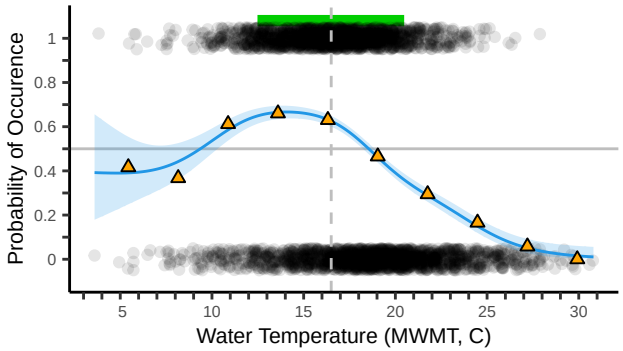

Rhyacophilidae −Rhyacophila grandis group
nOcc=141; WAopt=16.4; PctRange=12.8−19.5
Unclear; Cold

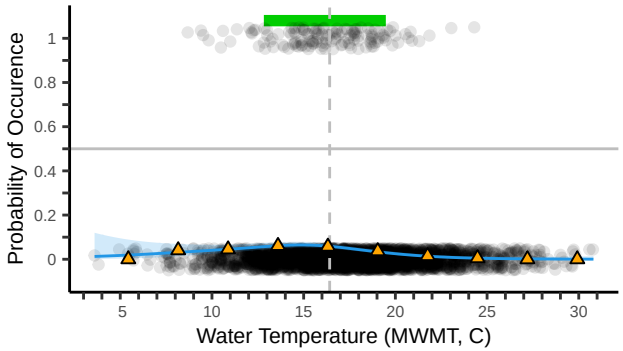

Rhyacophilidae −Rhyacophila hyalinata group
nOcc=709; WAopt=14.8; PctRange=11.5−20.2
Decreaser*; Cool

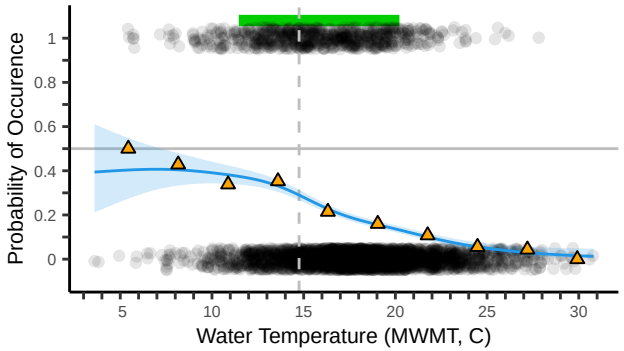

Rhyacophilidae −Rhyacophila malkini
nOcc=53; WAopt=22.5; PctRange=18.4−24.5
Unclear; Warm

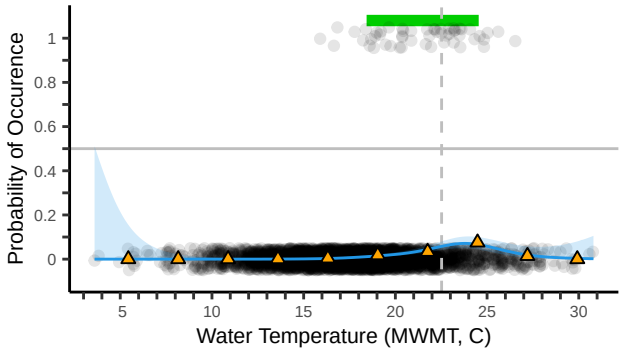

Rhyacophilidae −Rhyacophila narvae
nOcc=942; WAopt=16.5; PctRange=12.5−20.2
Unimodal/Decreaser; Cool

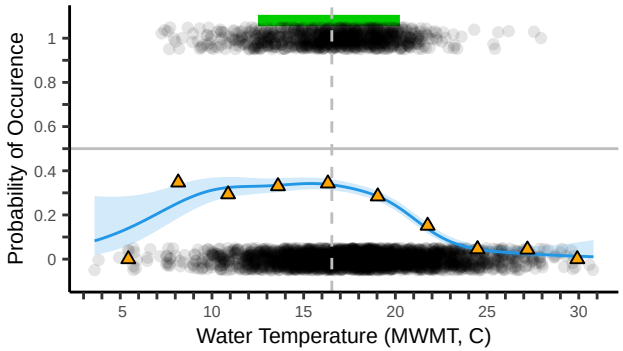

Rhyacophilidae −Rhyacophila sibirica group
nOcc=108; WAopt=13.7; PctRange=10.4−19.5
Unclear; Cold

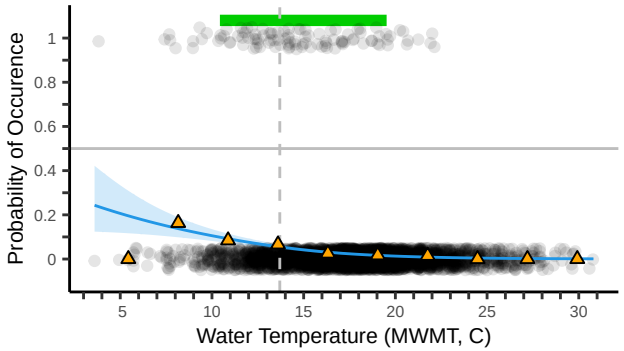

Rhyacophilidae −Rhyacophila vagrita group
nOcc=73; WAopt=14.6; PctRange=11.5−17.9
Unclear; Cold Stenotherm

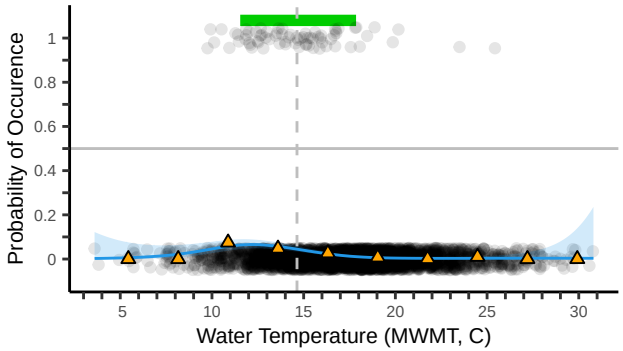

Rhyacophilidae −Rhyacophila verrula group
nOcc=86; WAopt=12.1; PctRange=9.3−18.8
Decreaser; Cold Stenotherm*

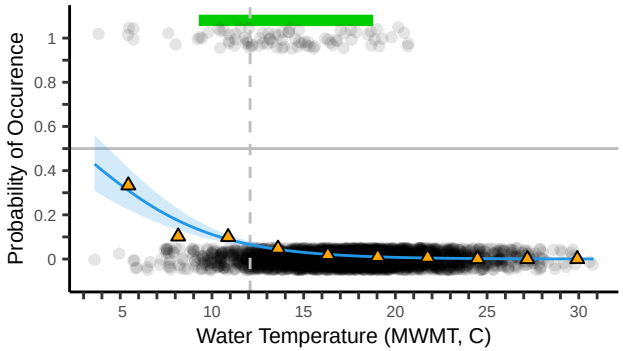

Trichoptera

Rhyacophilidae −Rhyacophila vofixa group
nOcc=252; WAopt=13.1; PctRange=9.8−17.3
Decreaser*; Cold Stenotherm

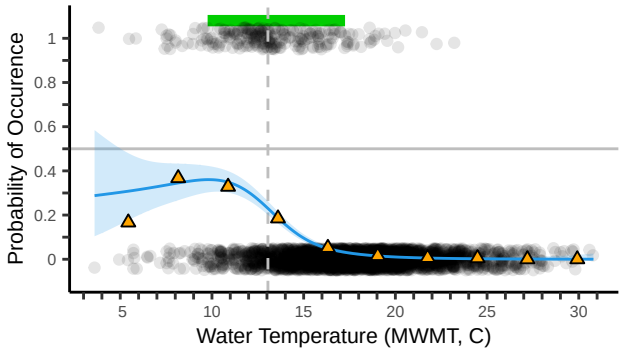

Sericostomatidae −Gumaga
nOcc=240; WAopt=18.9; PctRange=16.5−21.6
Unclear; Cool−Warm

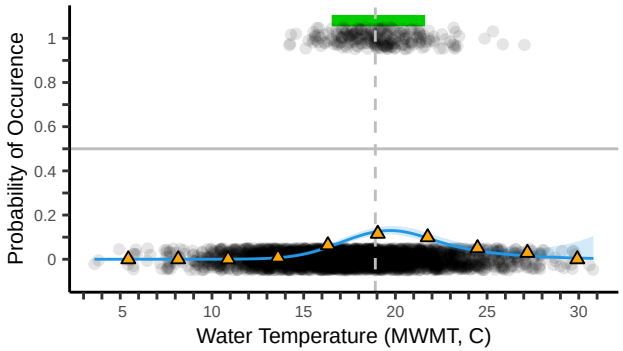

Uenoidae
nOcc=1,640; WAopt=14.9; PctRange=12.2−20.7
Decreaser; Cool

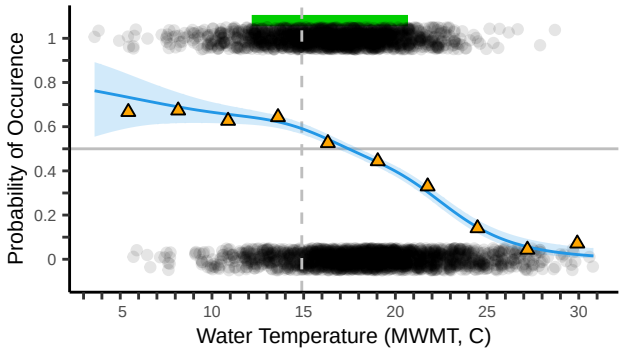

Uenoidae −Farula
nOcc=41; WAopt=17.9; PctRange=15.1−20.0
Unclear; Cool

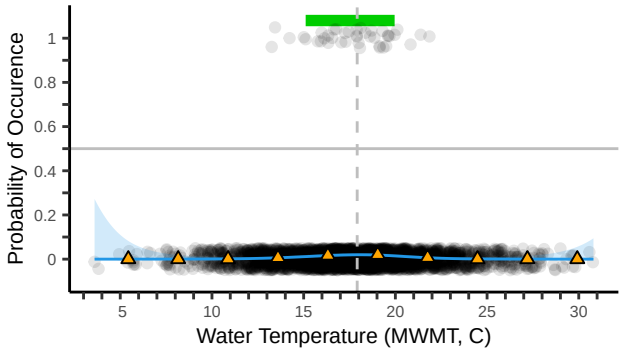

Uenoidae −Neophylax
nOcc=1,174; WAopt=16.5; PctRange=13.3−21.0
Unimodal*; Cool

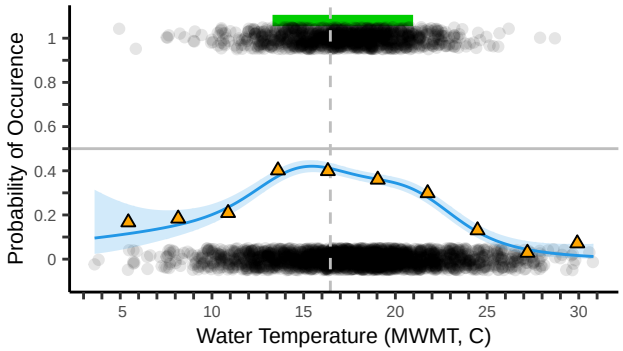

Uenoidae −Neophylax rickeri
nOcc=349; WAopt=19.2; PctRange=16.4−22.1
Unimodal*; Cool−Warm

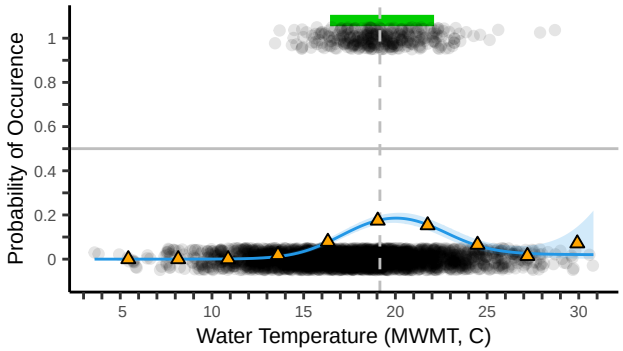

Uenoidae −Neophylax splendens
nOcc=272; WAopt=17.1; PctRange=13.8−20.4
Unclear; Cool

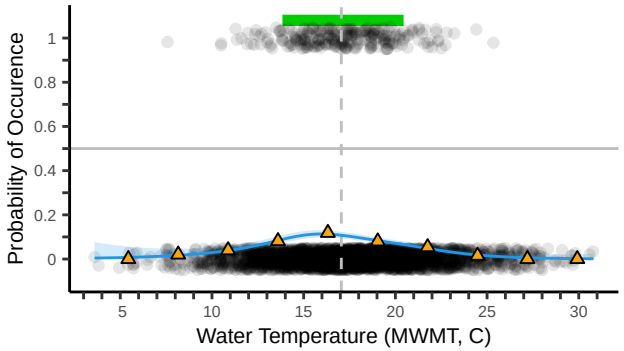

Uenoidae −Neothremma
nOcc=359; WAopt=12.9; PctRange=9.8−17.8
Decreaser; Cold Stenotherm

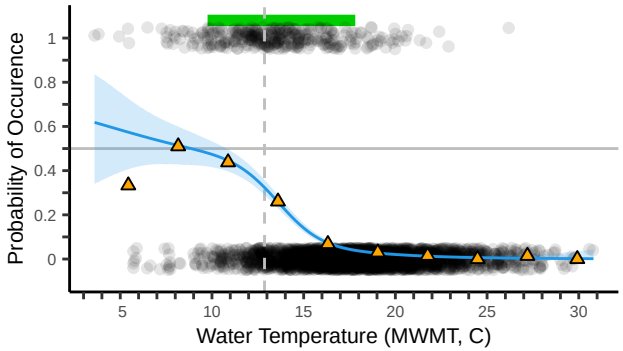

Uenoidae −Oligophlebodes
nOcc=320; WAopt=14.3; PctRange=11.1−18.4
Decreaser; Cold

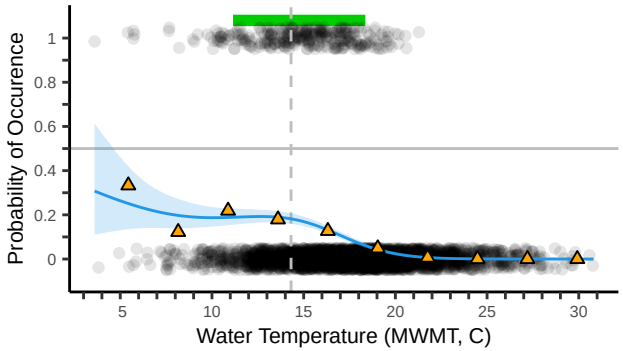

Supplement: Supplement4 [file NIHMS2055599-supplement-Supplement4.pdf]
