# Supplementary material for: Improved thermal preferences and a stressor index derived from modeled stream temperatures and regional taxonomic standards for freshwater macroinvertebrates of the Pacific Northwest, USA
Source: Ecol Indic. Author manuscript; Available in PMC 2025 Apr 9. (PMC11980781; doi:10.1016/j.ecolind.2024.111869)

Diptera_NotChiros

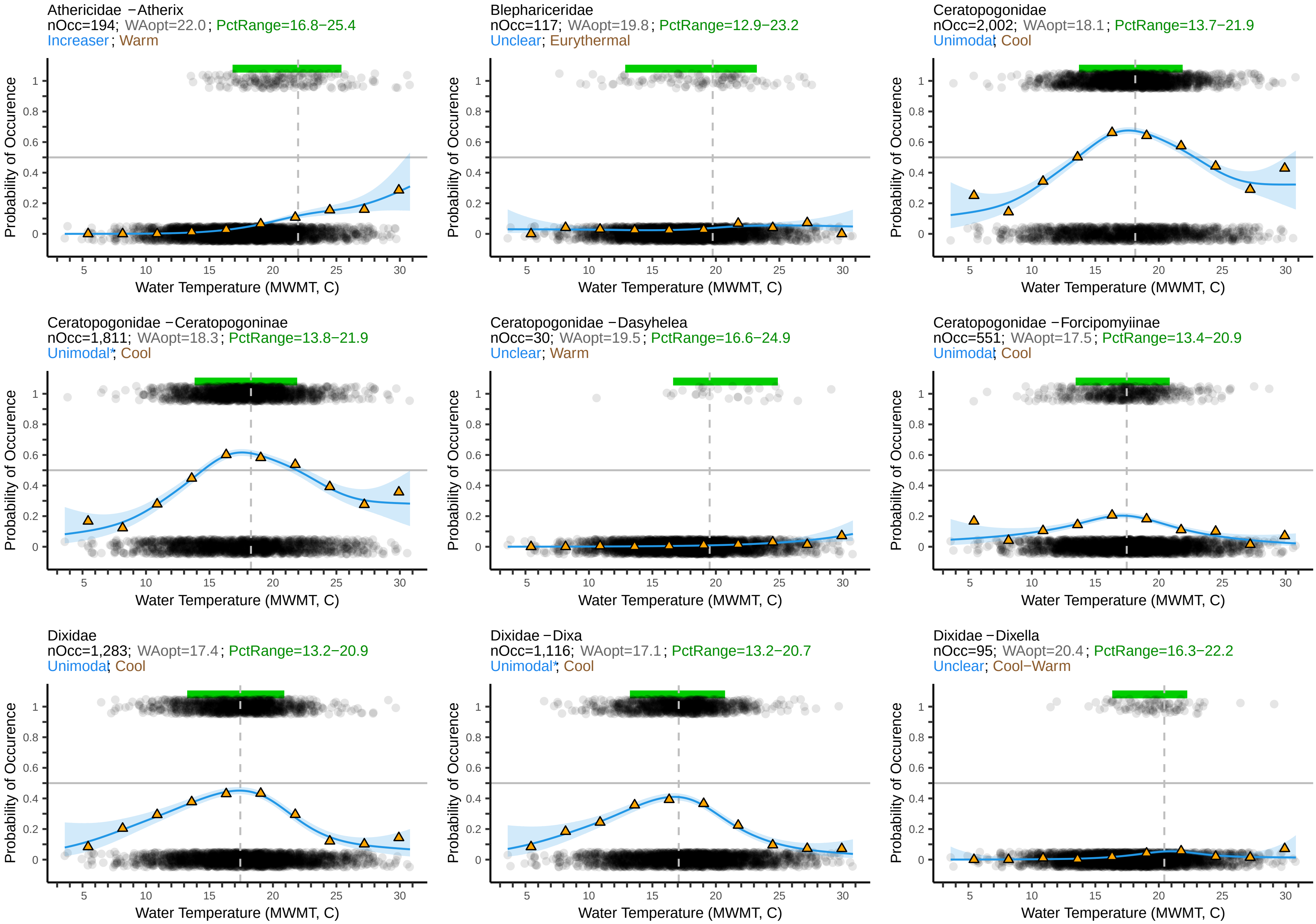

Diptera_NotChiros

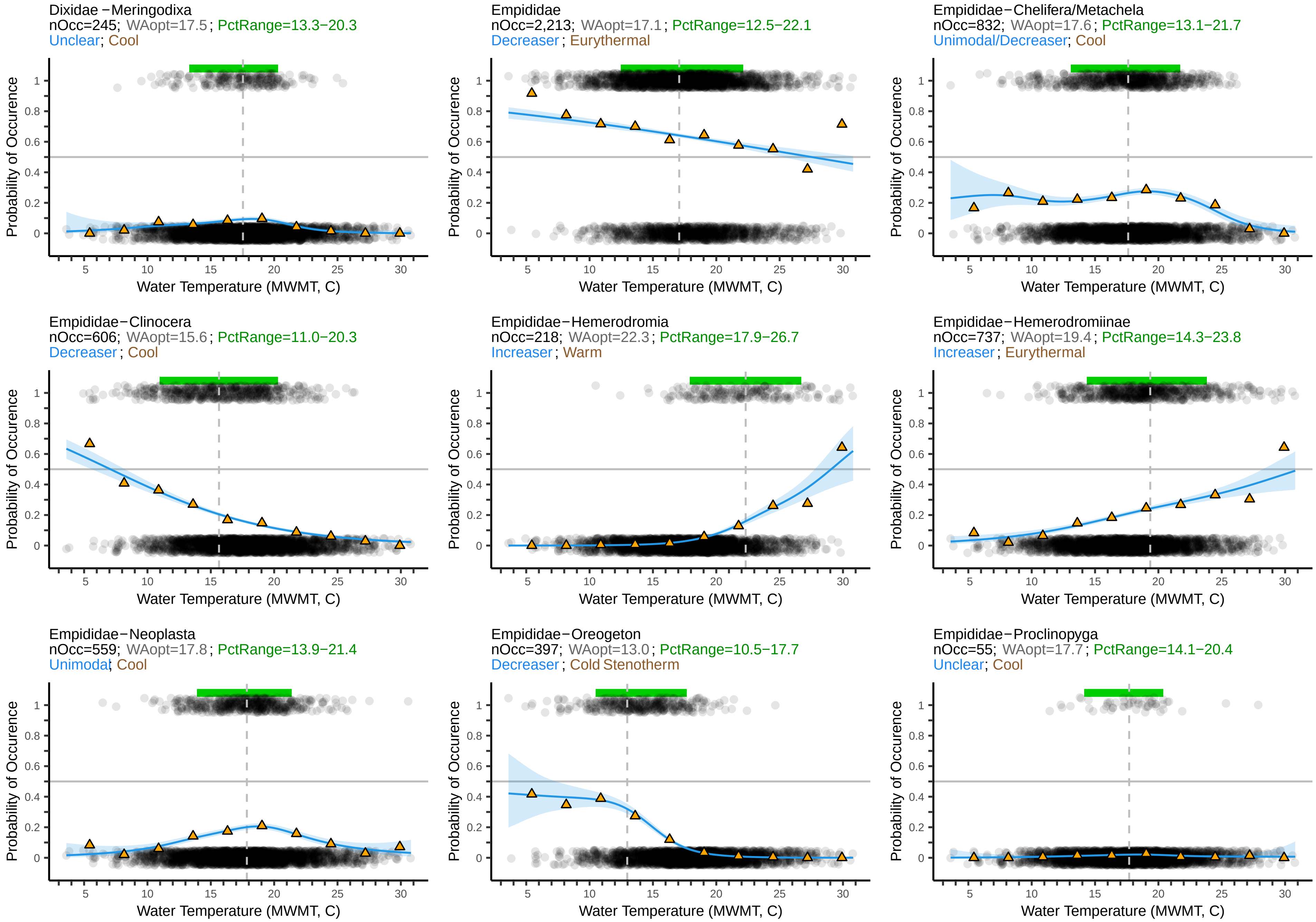

Diptera_NotChiros

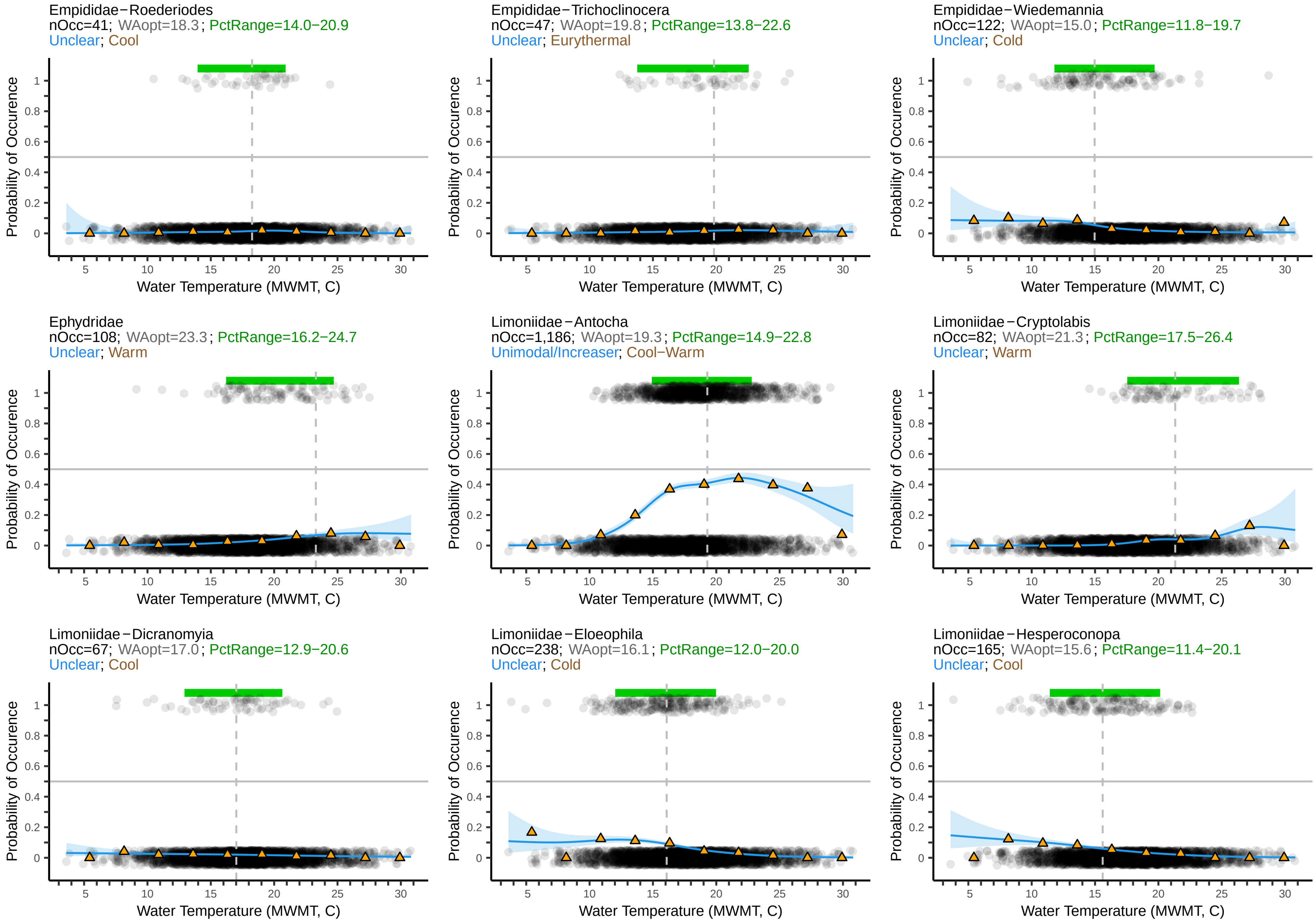

Diptera_NotChiros

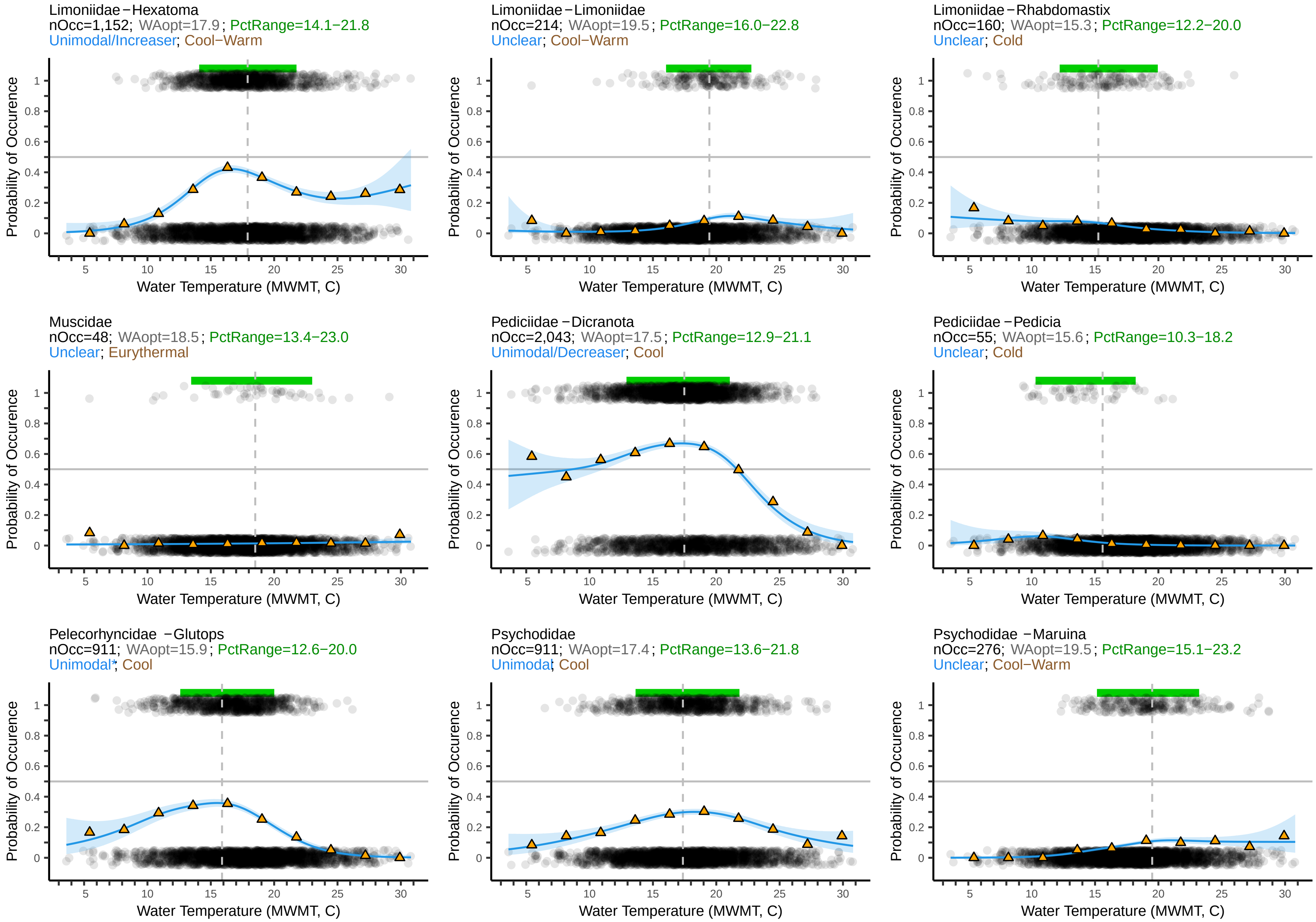

Diptera_NotChiros

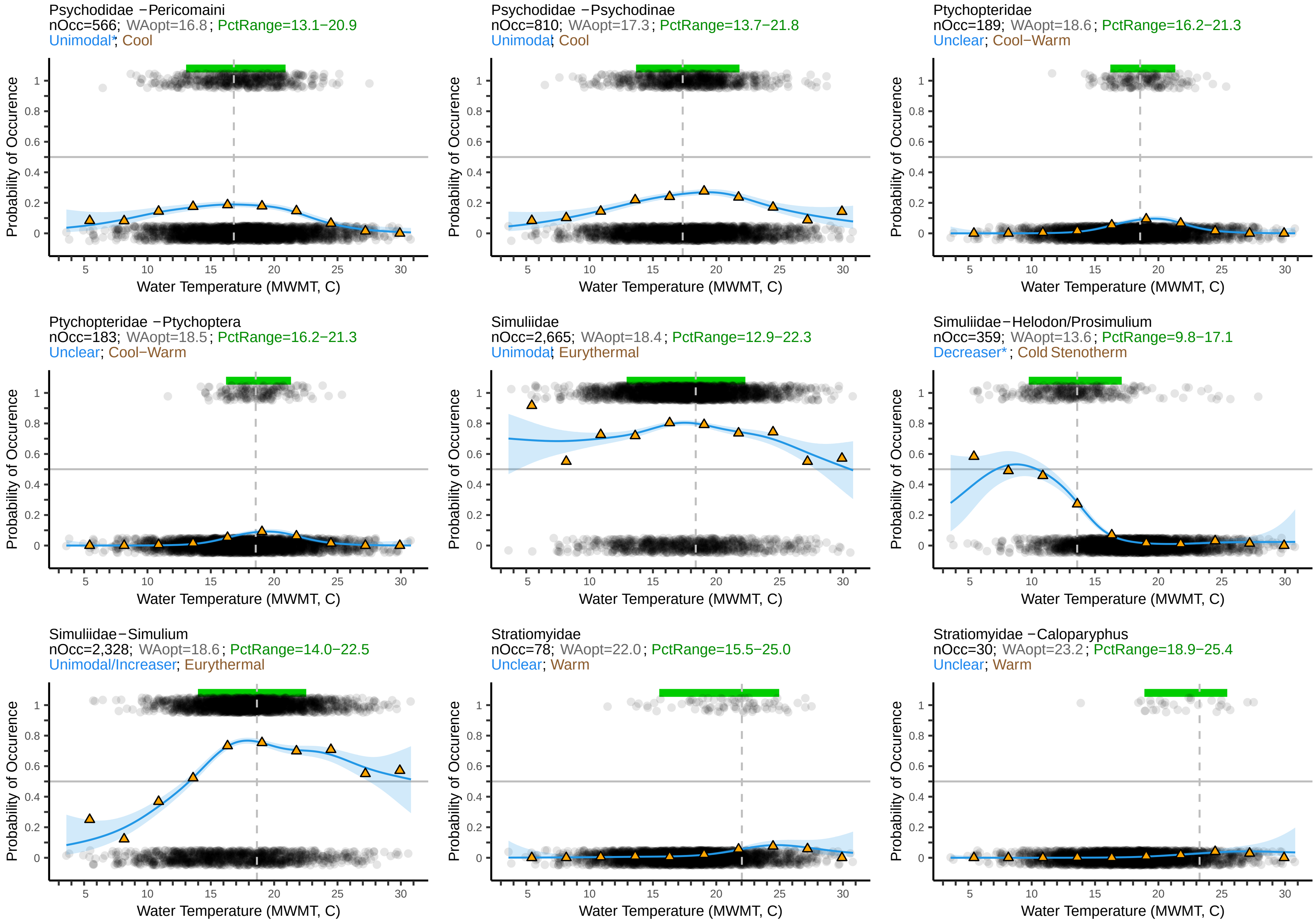

Diptera_NotChiros

Tabanidae
nOcc=169; WAopt=19.9; PctRange=17.1−23.6
Unclear; Warm

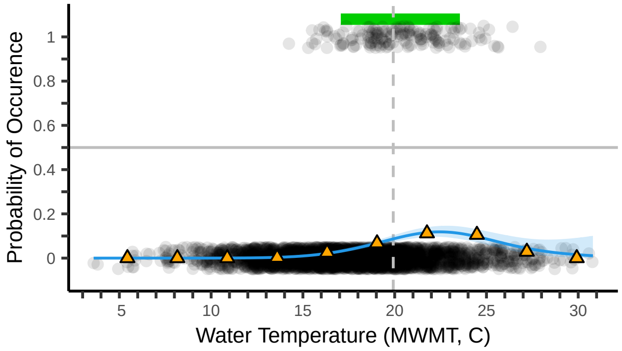

Thaumaleidae
nOcc=200; WAopt=14.7; PctRange=11.3−19.1
Unclear; Cold

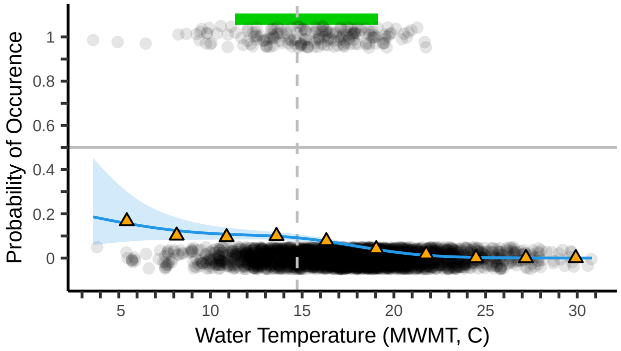

Tipulidae
nOcc=2,962; WAopt=18.1; PctRange=13.0−22.0
Unimodal; Cool

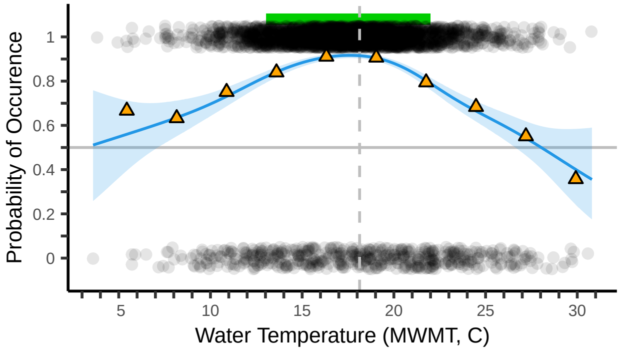

Tipulidae−Tipula
nOcc=388; WAopt=18.4; PctRange=14.4−22.3
Unclear; Eurythermal

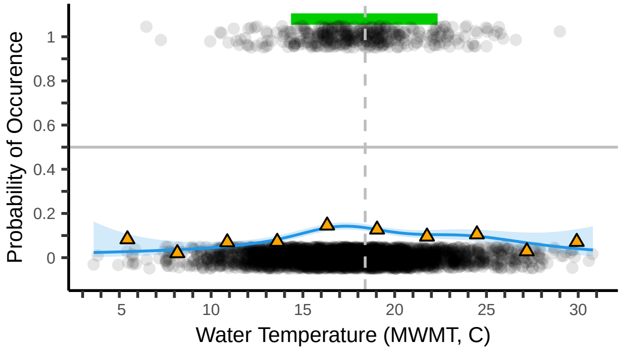

Supplement: Supplement5 [file NIHMS2055599-supplement-Supplement5.pdf]
