# Supplementary material for: Improved thermal preferences and a stressor index derived from modeled stream temperatures and regional taxonomic standards for freshwater macroinvertebrates of the Pacific Northwest, USA
Source: Ecol Indic. Author manuscript; Available in PMC 2025 Apr 9. (PMC11980781; doi:10.1016/j.ecolind.2024.111869)
Supplement: Supplement8 [file NIHMS2055599-supplement-Supplement8.pdf]

Supplement 5

Thermal Metric plots by MWMT class

Thermal Metric plots by MWMT class

Table S5-1. 'Translations' of the abbreviated metric names in the output

Metric_abbrev	Full metric description
ni_total	# total individuals
nt_total	# total taxa
nt_ti_stenocold	# cold stenotherm taxa
nt_ti_cold	# cold taxa
nt_ti_cool	# cool taxa
nt_ti_warm	# warm taxa
nt_ti_stenowarm	# warm stenotherm taxa
nt_ti_eury	# eurythermal taxa
nt_ti_cowa	# cool/warm taxa
nt_ti_na	# taxa no assignment
nt_ti_stenocold_cold	# cold stenotherm + cold taxa
nt_ti_stenocold_cold_cool	# cold stenotherm + cold + cool taxa
nt_ti_cowa_warm_stenowarm	# cool/warm + warm + warm stenotherm taxa
nt_ti_warm_stenowarm	# warm + warm stenotherm taxa
pi_ti_stenocold	% cold stenotherm indiv
pi_ti_cold	% cold indiv
pi_ti_cool	% cool indiv
pi_ti_warm	% warm indiv
pi_ti_stenowarm	% warm stenotherm indiv
pi_ti_eury	% eurythermal indiv
pi_ti_cowa	% cool/warm indiv
pi_ti_na	% indiv no assignment
pi_ti_stenocold_cold	% cold stenotherm + cold indiv
pi_ti_stenocold_cold_cool	% cold stenotherm + cold + cool indiv
pi_ti_cowa_warm_stenowarm	% cool/warm + warm + warm stenotherm indiv
pi_ti_warm_stenowarm	% warm + warm stenotherm indiv
pt_ti_stenocold	% cold stenotherm taxa
pt_ti_cold	% cold taxa
pt_ti_cool	% cool taxa
pt_ti_warm	% warm taxa
t_ti_stenowarm	% warm stenotherm taxa
pt_ti_eury	% eurythermal taxa
pt_ti_cowa	% cool/warm taxa
pt_ti_na	% taxa no assignment
pt_ti_stenocold_cold	% cold stenotherm + cold taxa
pt_ti_stenocold_cold_cool	% cold stenotherm + cold + cool taxa
pt_ti_cowa_warm_stenowarm	% cool/warm + warm + warm stenotherm taxa
pt_ti_warm_stenowarm	% warm + warm stenotherm taxa

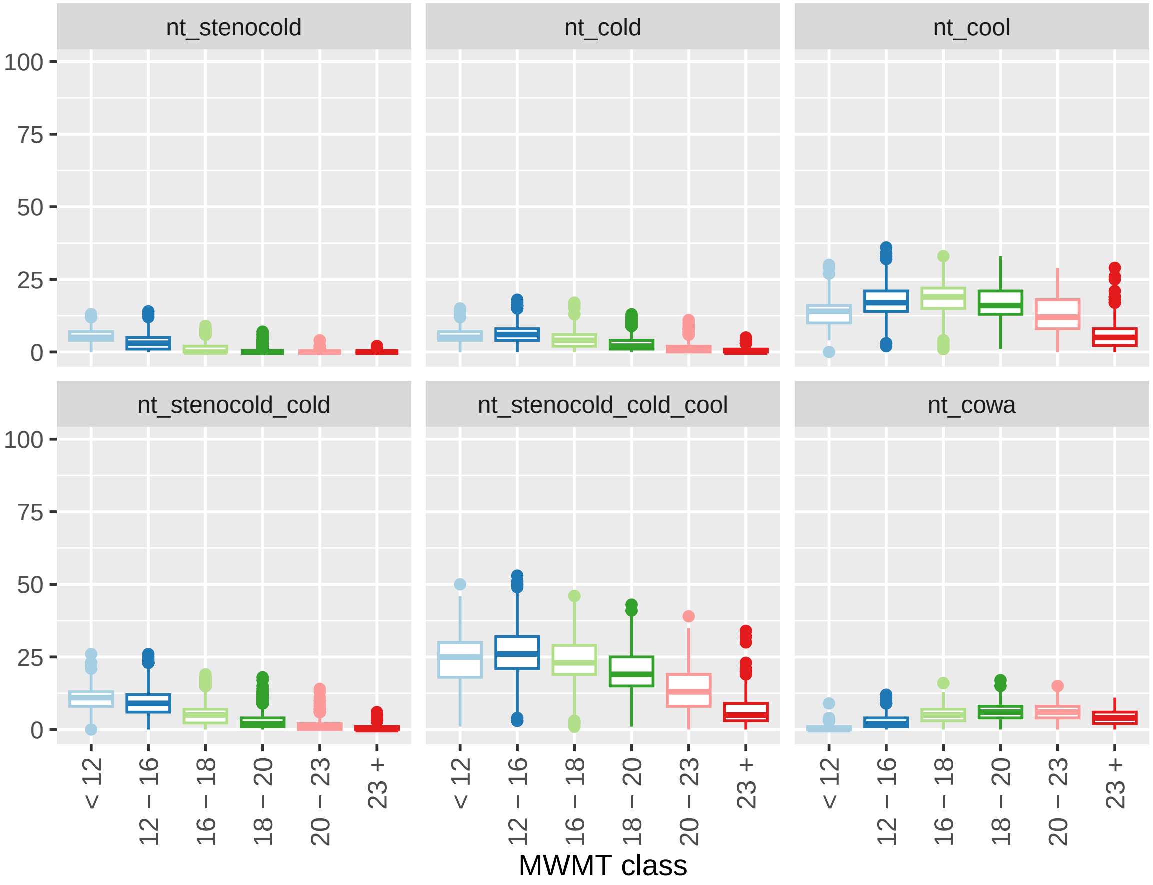
nt_stenocold
nt_cold
nt_cool
nt_stenocold_cold
nt_stenocold_cold_cool
nt_cowa
100
75
50
25
0
< 12
12 – 16
16 – 18
18 – 20
20 – 23
23 +
MWMT class

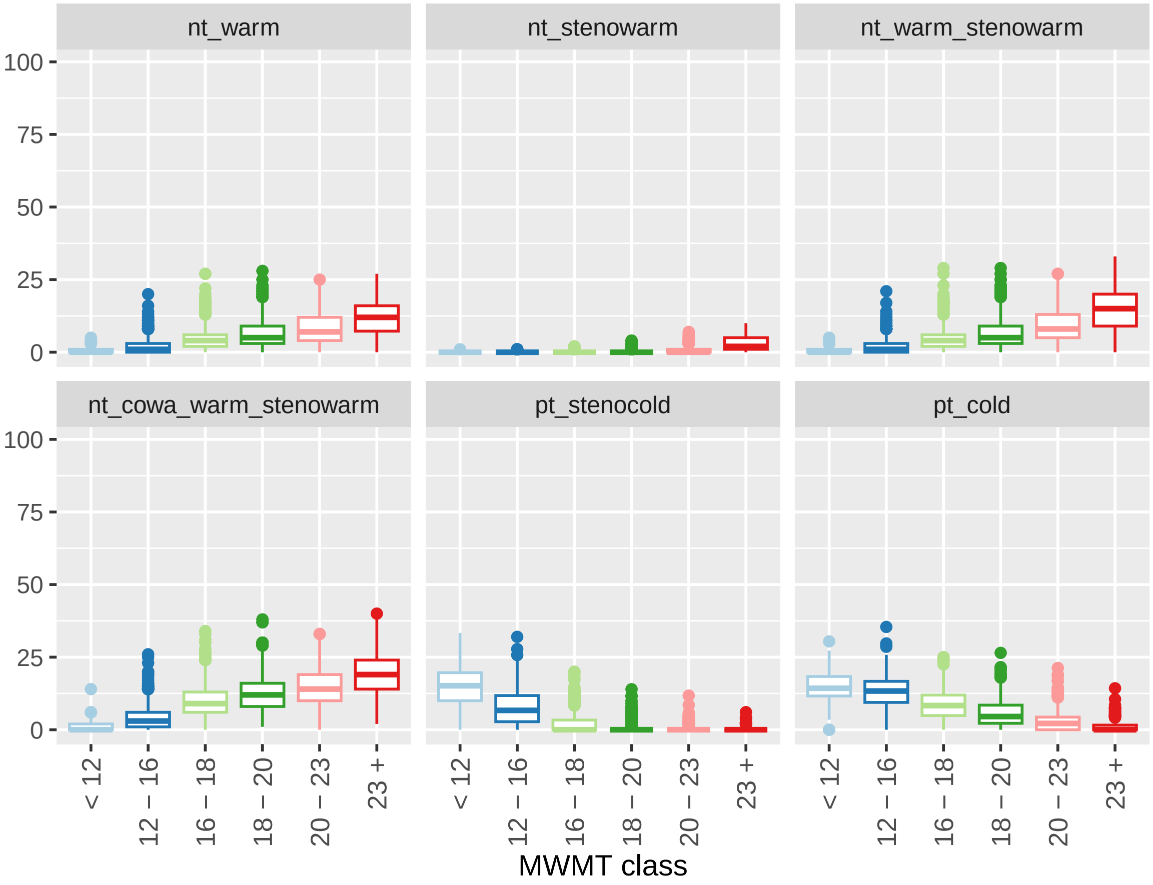

nt_warm
nt_stenowarm
nt_warm_stenowarm
nt_cowa_warm_stenowarm
pt_stenocold
pt_cold
100
75
50
25
0
< 12
12 – 16
16 – 18
18 – 20
20 – 23
23 +
MWMT class

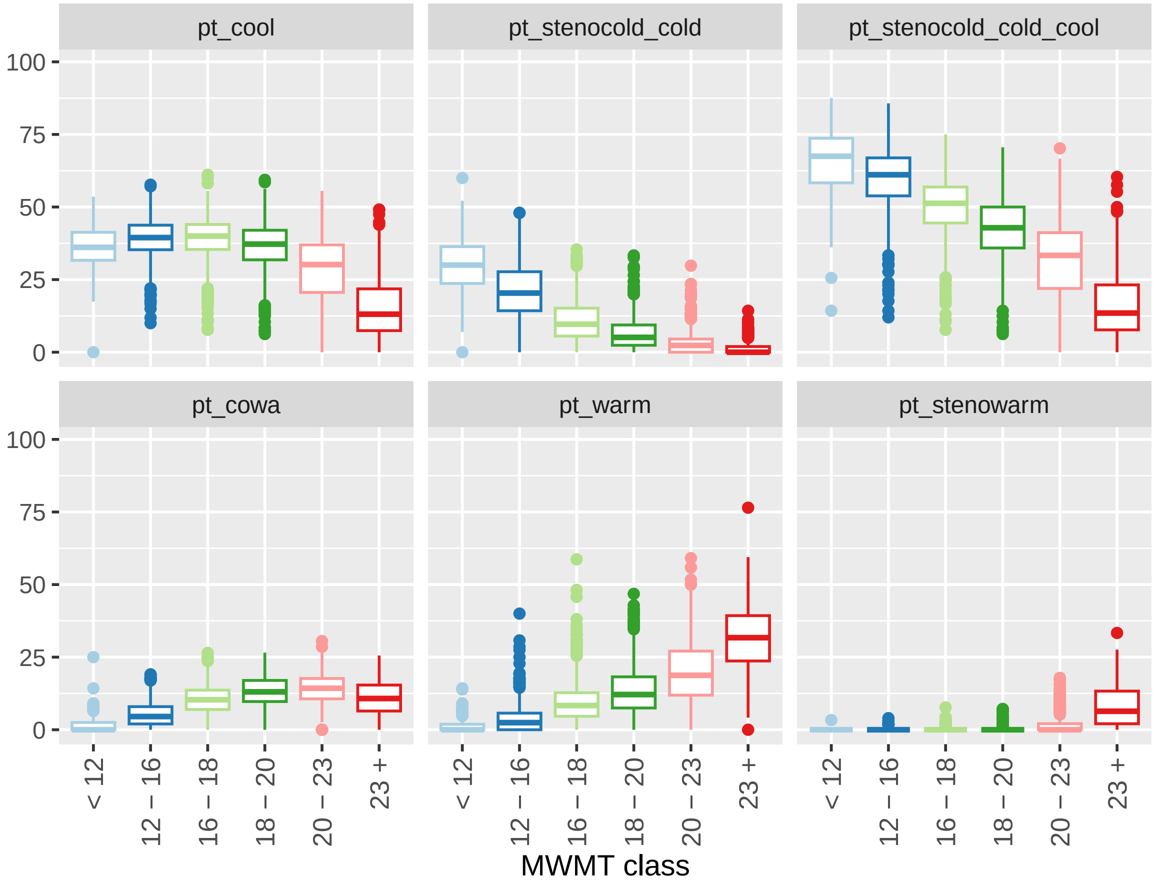

pt_cool
pt_stenocold_cold
pt_stenocold_cold_cool
pt_cowa
pt_warm
pt_stenowarm
100
75
50
25
0
< 12
12 – 16
16 – 18
18 – 20
20 – 23
23 +
MWMT class

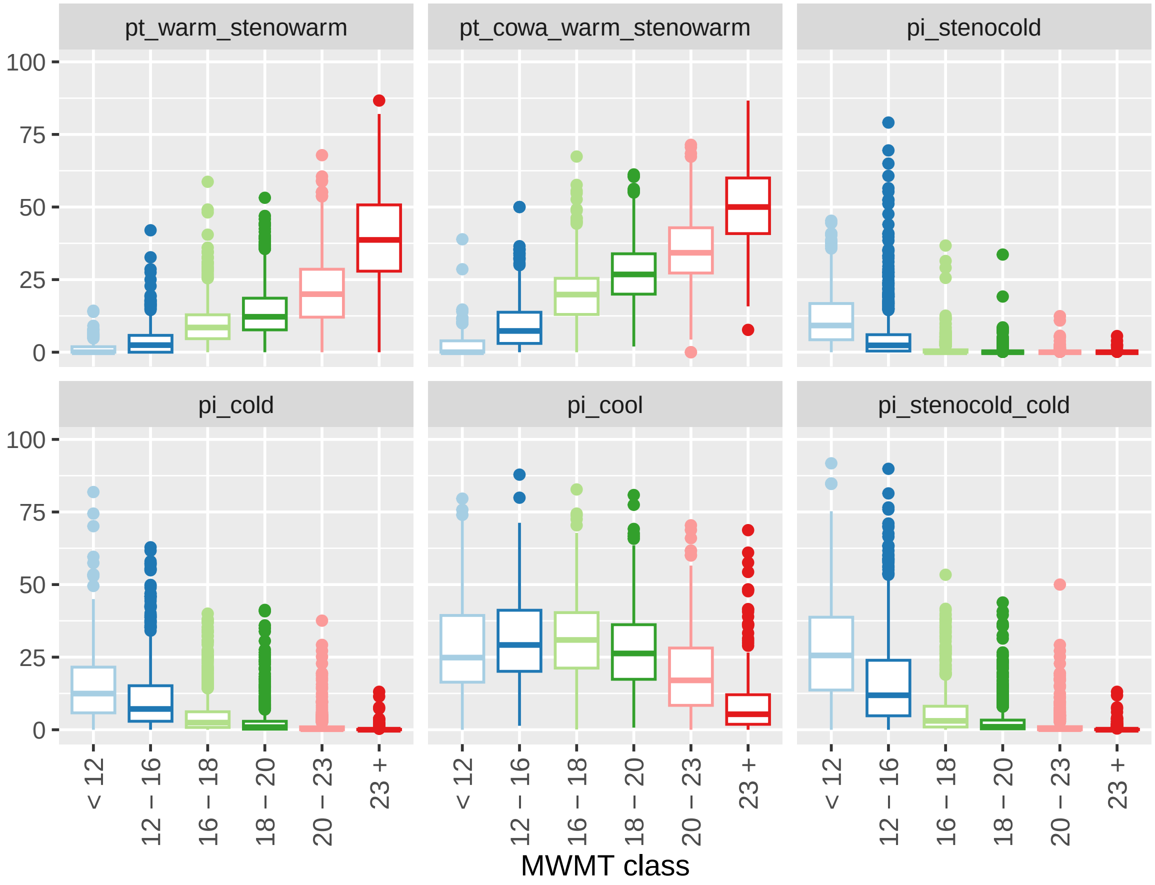
pt_warm_stenowarm
pt_cowa_warm_stenowarm
pi_stenocold
pi_cold
pi_cool
pi_stenocold_cold
100
75
50
25
0
< 12
12 – 16
16 – 18
18 – 20
20 – 23
23 +
MWMT class

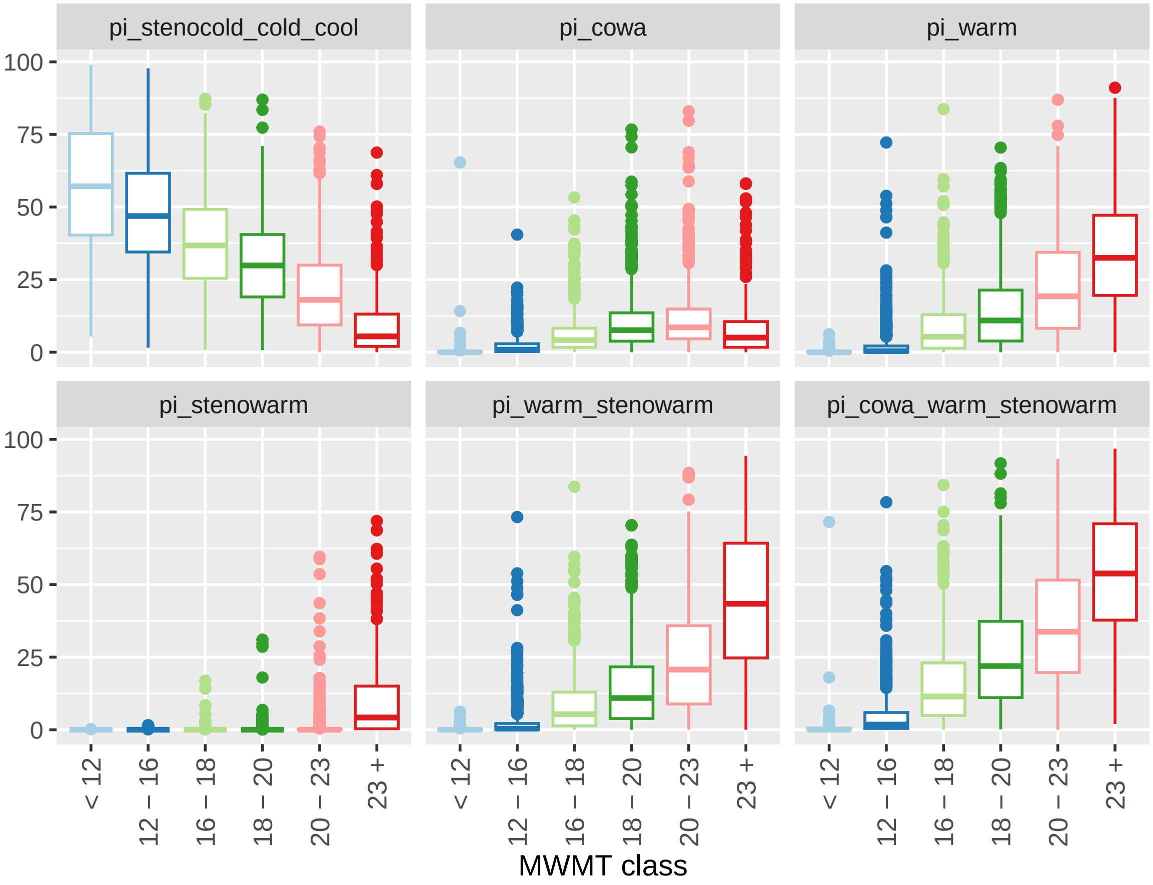

pi_stenocold_cold_cool
pi_cowa
pi_warm
pi_stenowarm
pi_warm_stenowarm
pi_cowa_warm_stenowarm
100
75
50
25
0
< 12
12 – 16
16 – 18
18 – 20
20 – 23
23 +
MWMT class

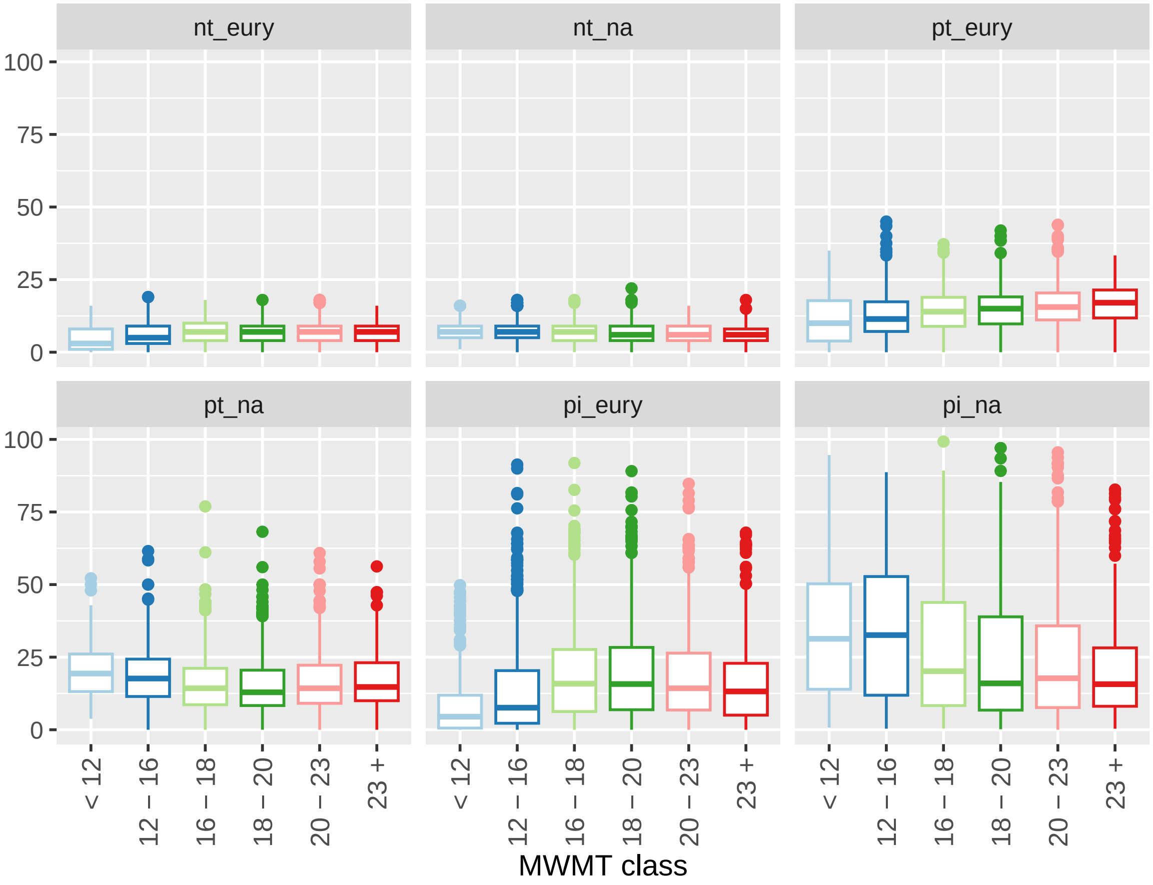

nt_eury
nt_na
pt_eury
pt_na
pi_eury
pi_na
100
75
50
25
0
< 12
12 – 16
16 – 18
18 – 20
20 – 23
23 +
MWMT class
