# Supplementary material for: Improved thermal preferences and a stressor index derived from modeled stream temperatures and regional taxonomic standards for freshwater macroinvertebrates of the Pacific Northwest, USA
Source: Ecol Indic. Author manuscript; Available in PMC 2025 Apr 9. (PMC11980781; doi:10.1016/j.ecolind.2024.111869)

NonInsect_NotMites

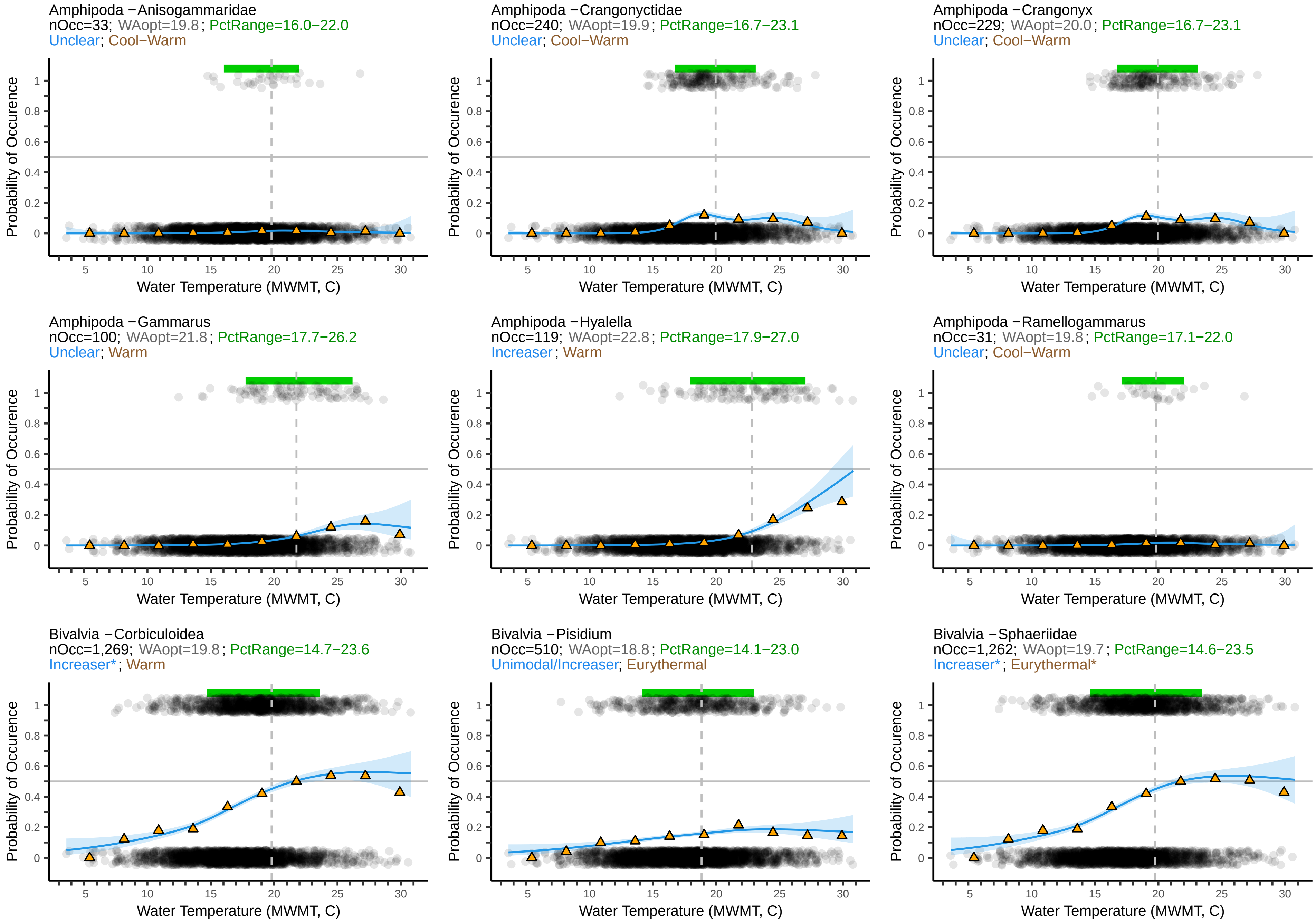

NonInsect_NotMites

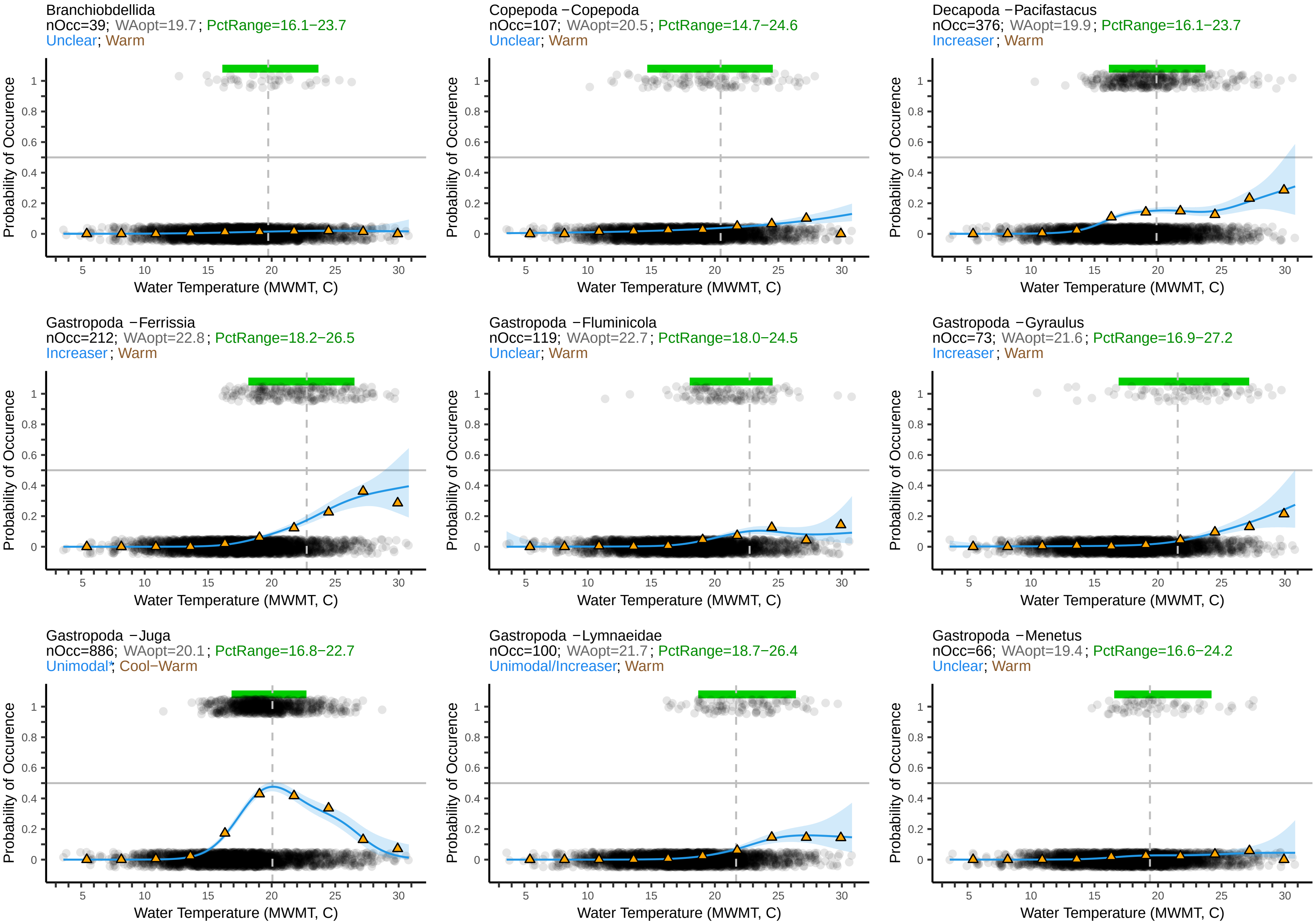

NonInsect_NotMites

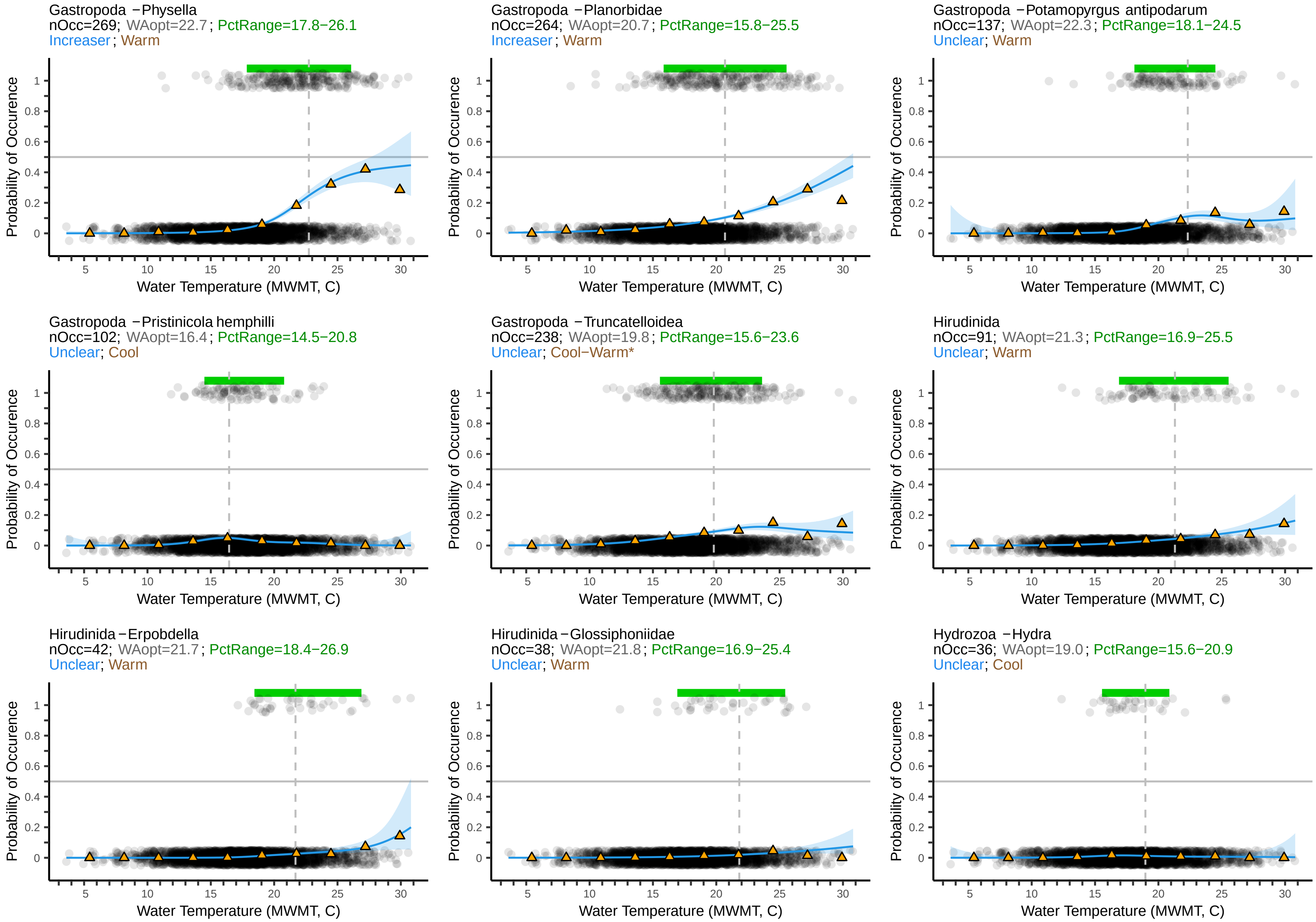

NonInsect_NotMites

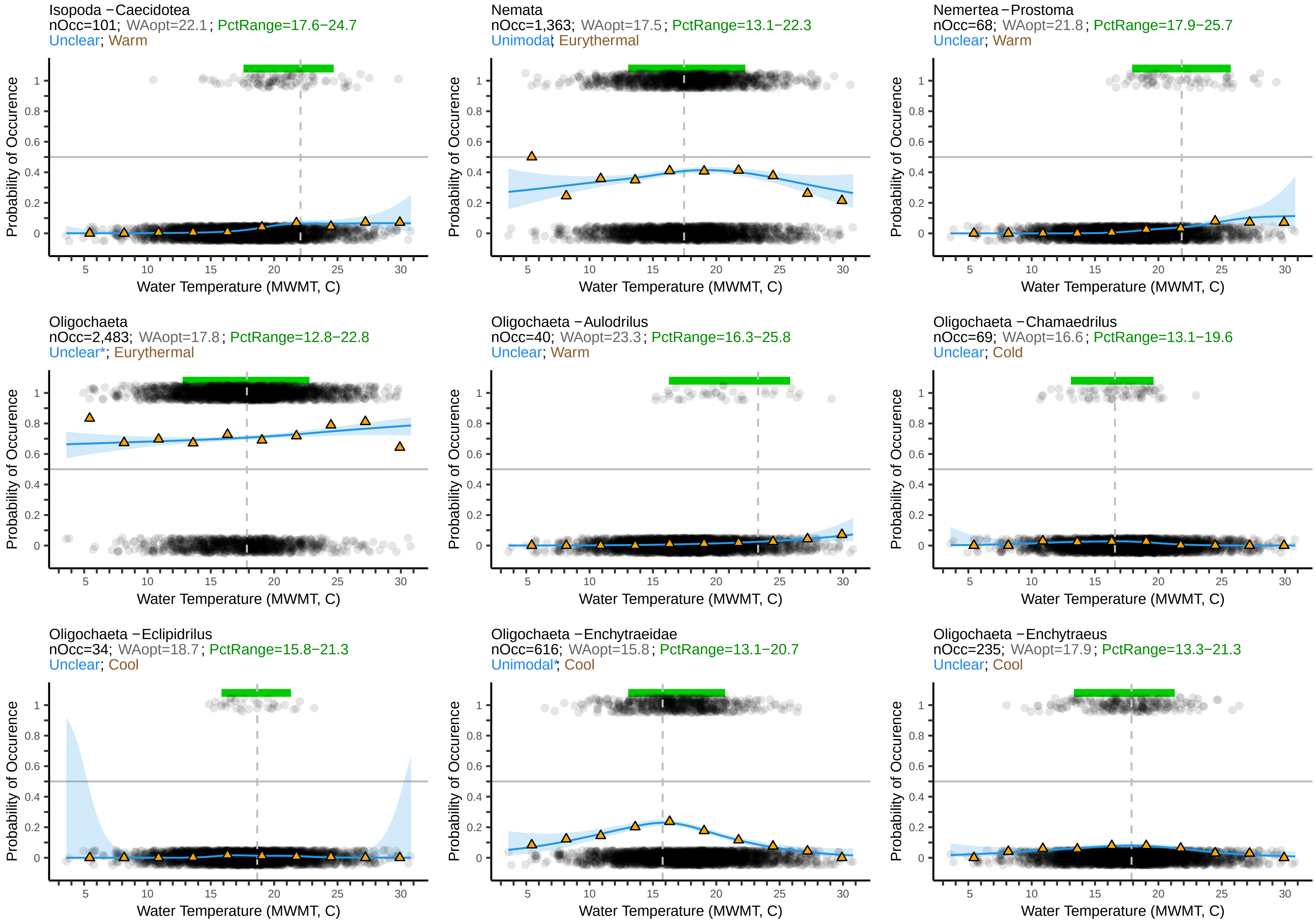

NonInsect_NotMites

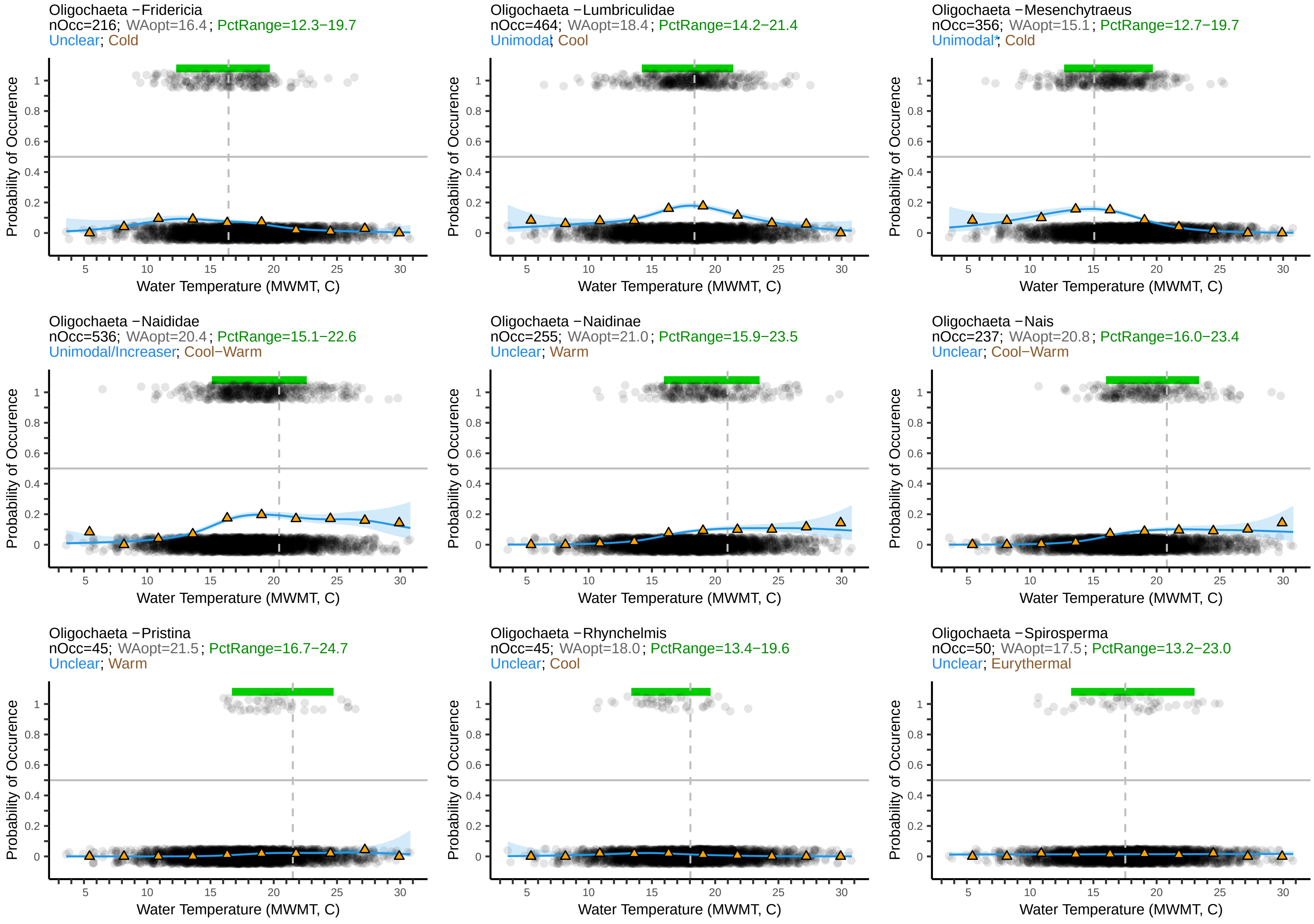

NonInsect_NotMites

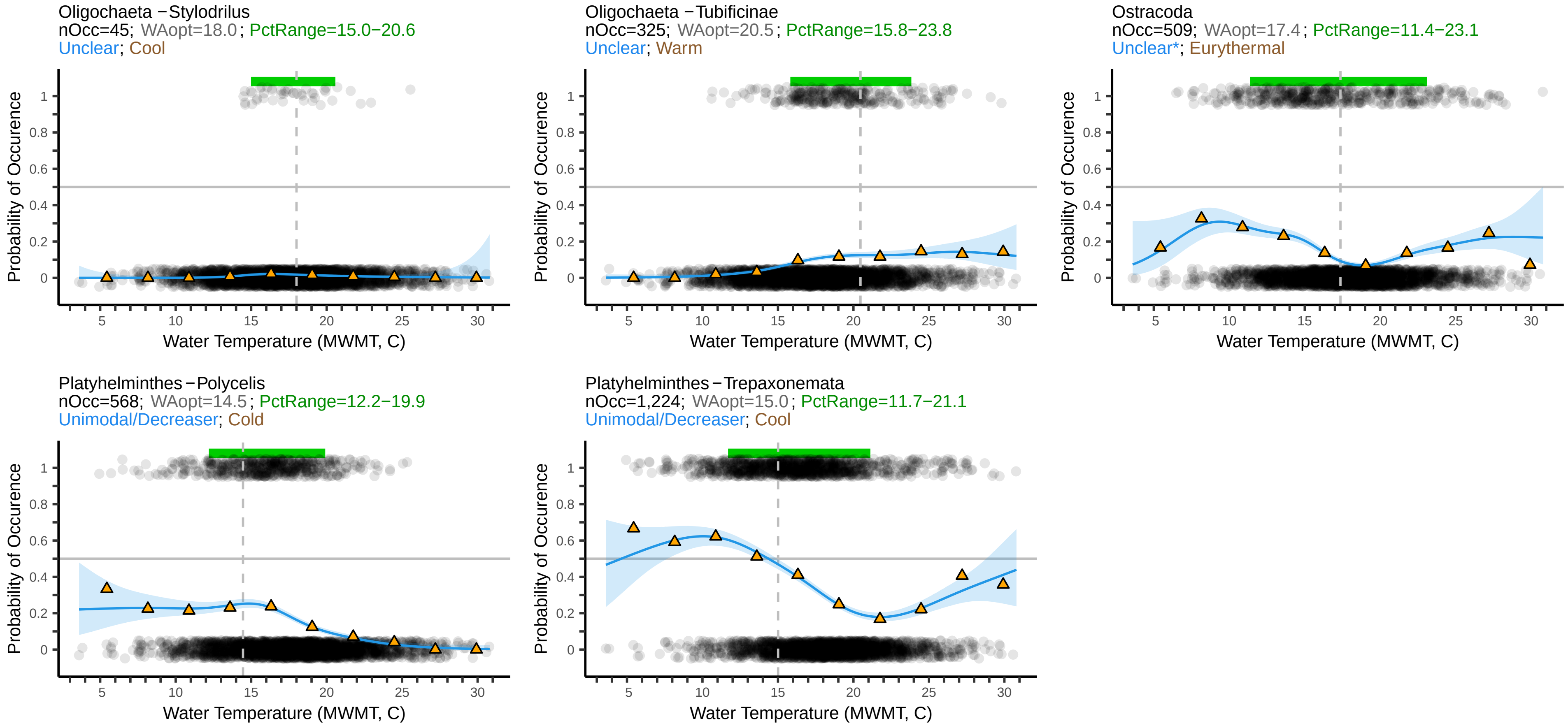

Supplement: Supplement9 [file NIHMS2055599-supplement-Supplement9.pdf]
