# Supplementary figures and images for: Improved thermal preferences and a stressor index derived from modeled stream temperatures and regional taxonomic standards for freshwater macroinvertebrates of the Pacific Northwest, USA
Source: Ecol Indic. Author manuscript; Available in PMC 2025 Apr 9. (PMC11980781; doi:10.1016/j.ecolind.2024.111869)

## Coleoptera

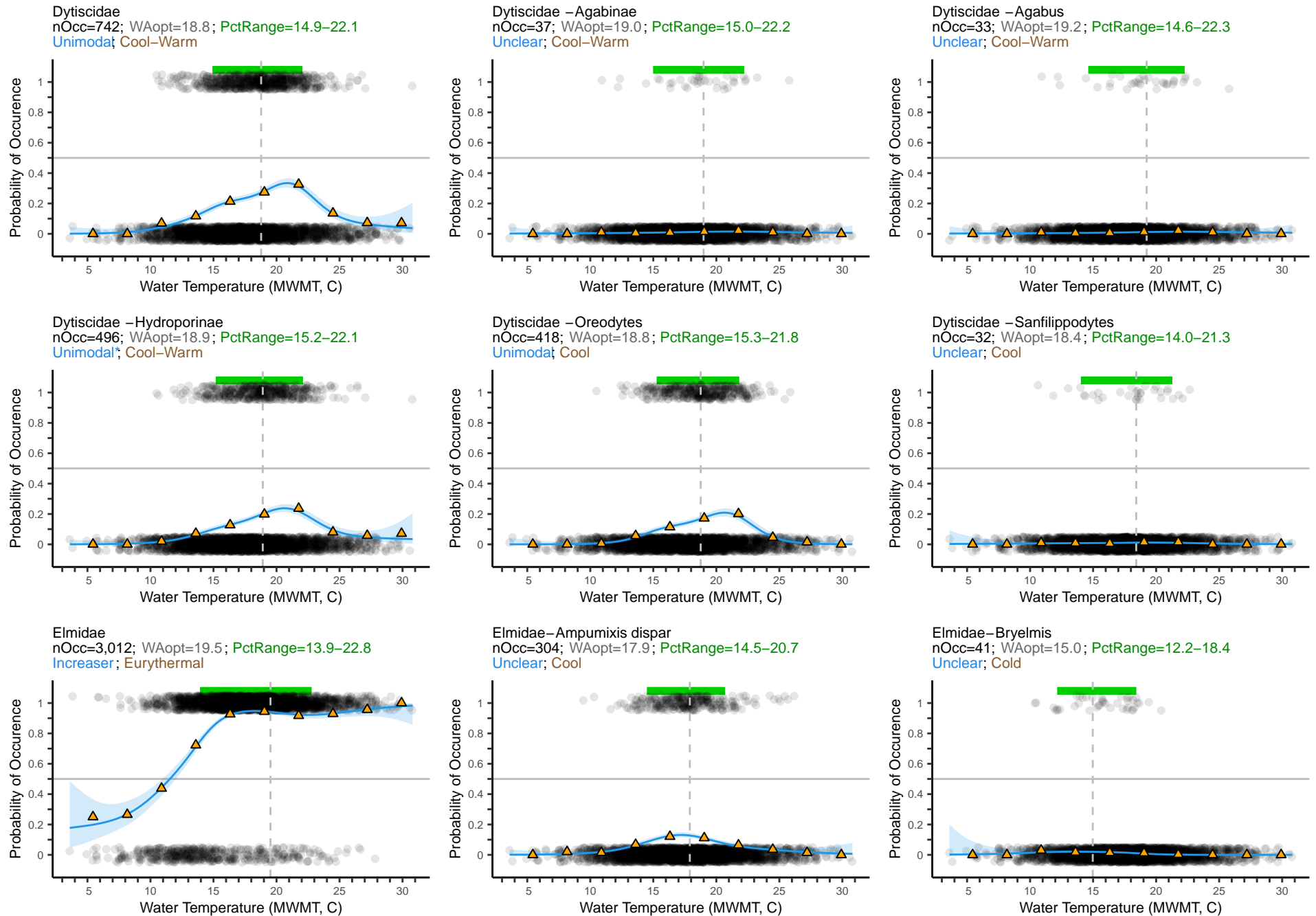

## Coleoptera

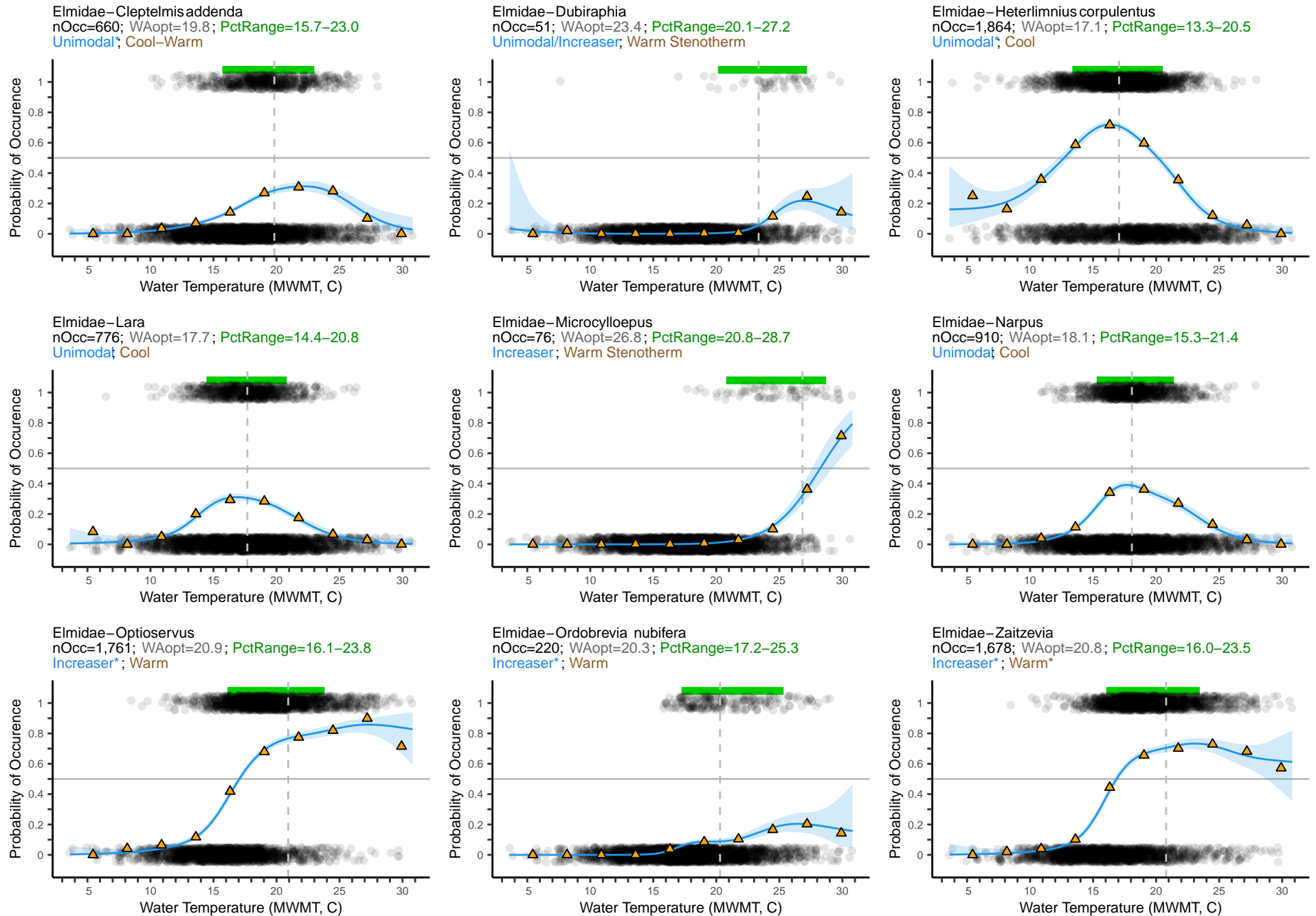

## Coleoptera

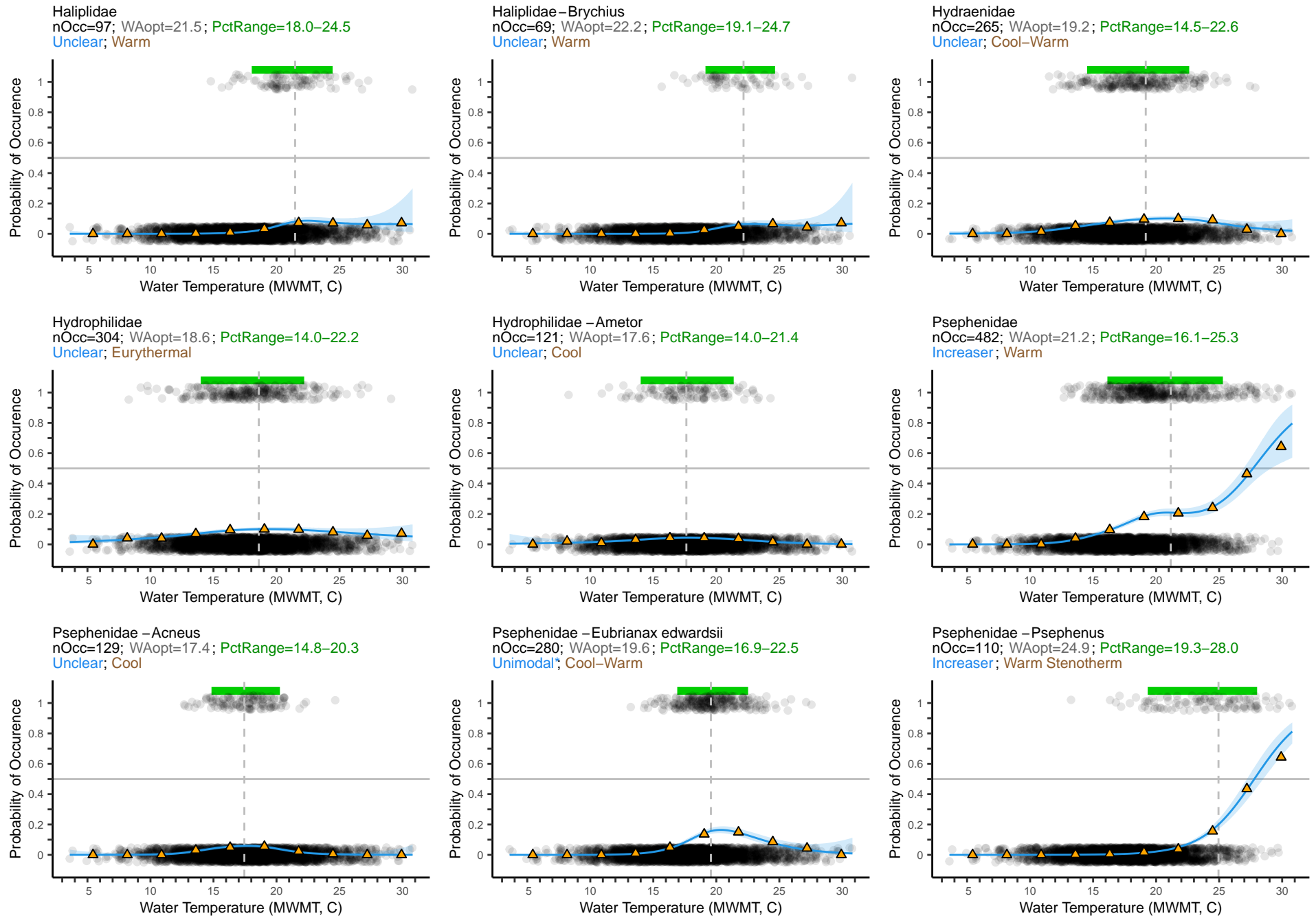

Supplement: Supplement10 [file NIHMS2055599-supplement-Supplement10.pdf]

## NonInsect\_Mites

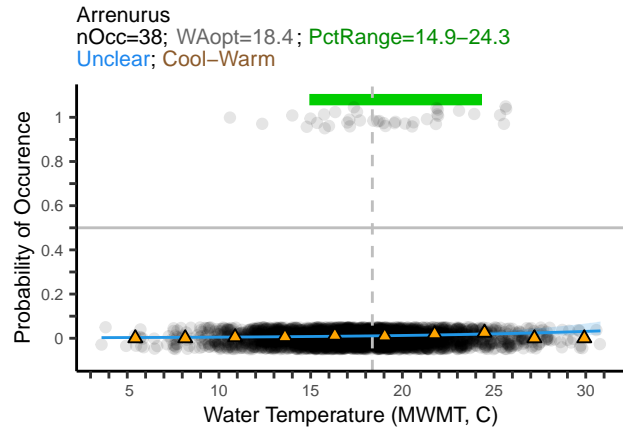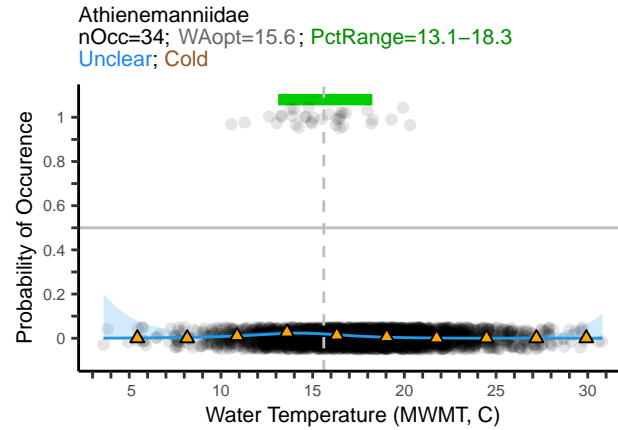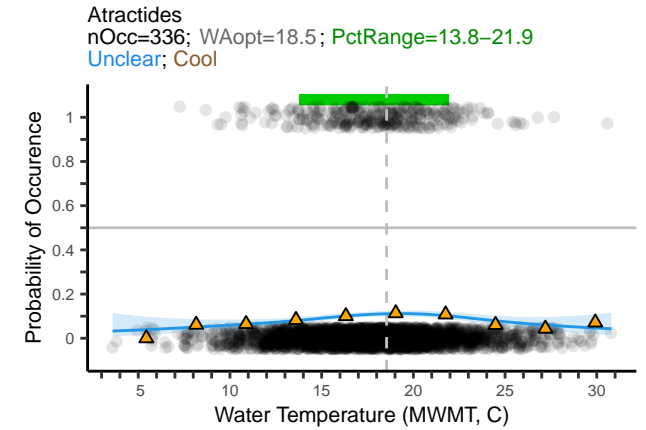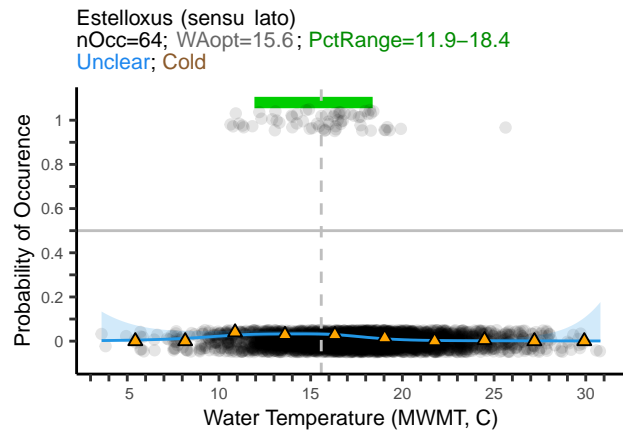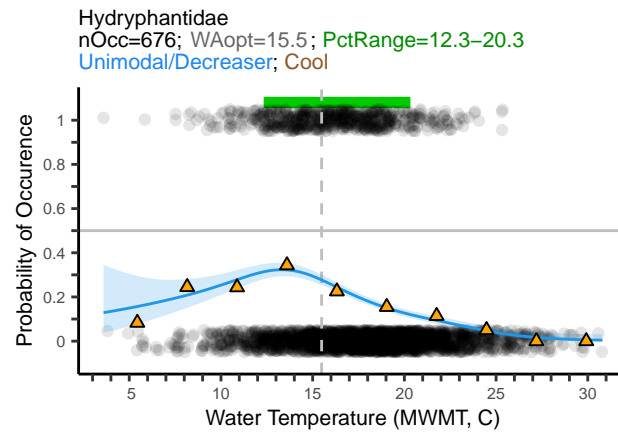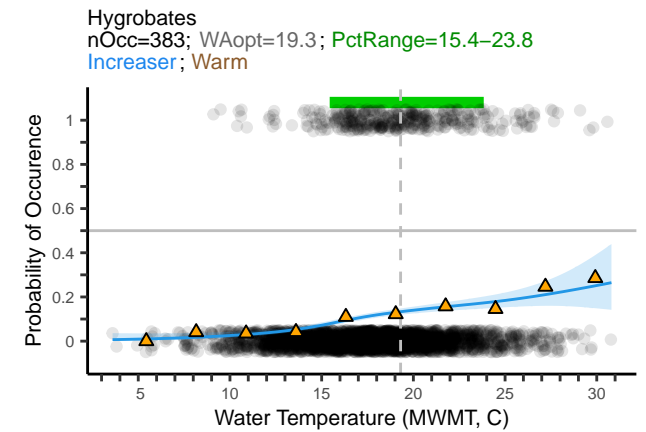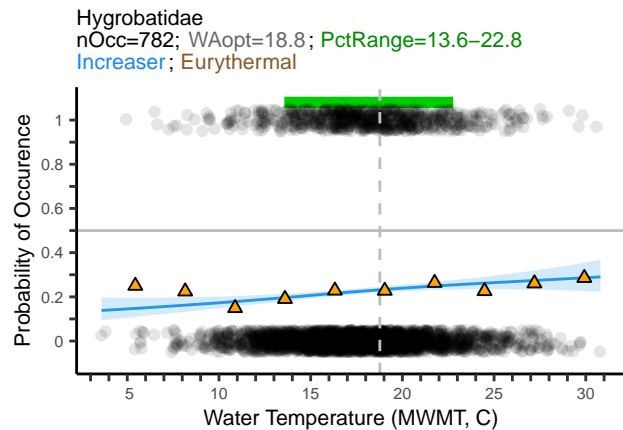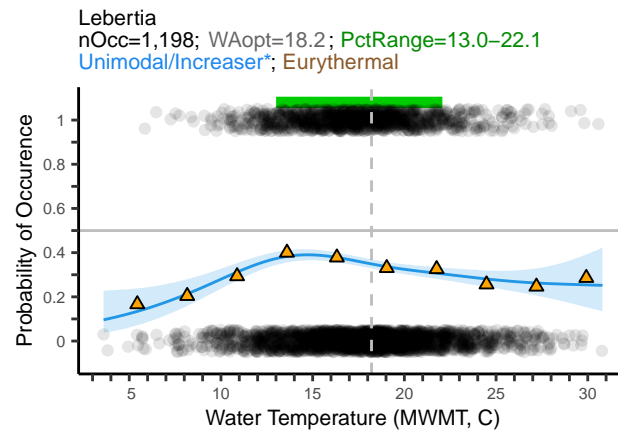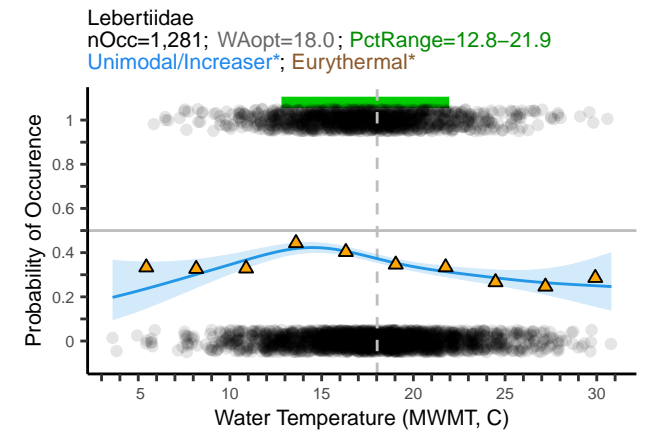

## NonInsect\_Mites

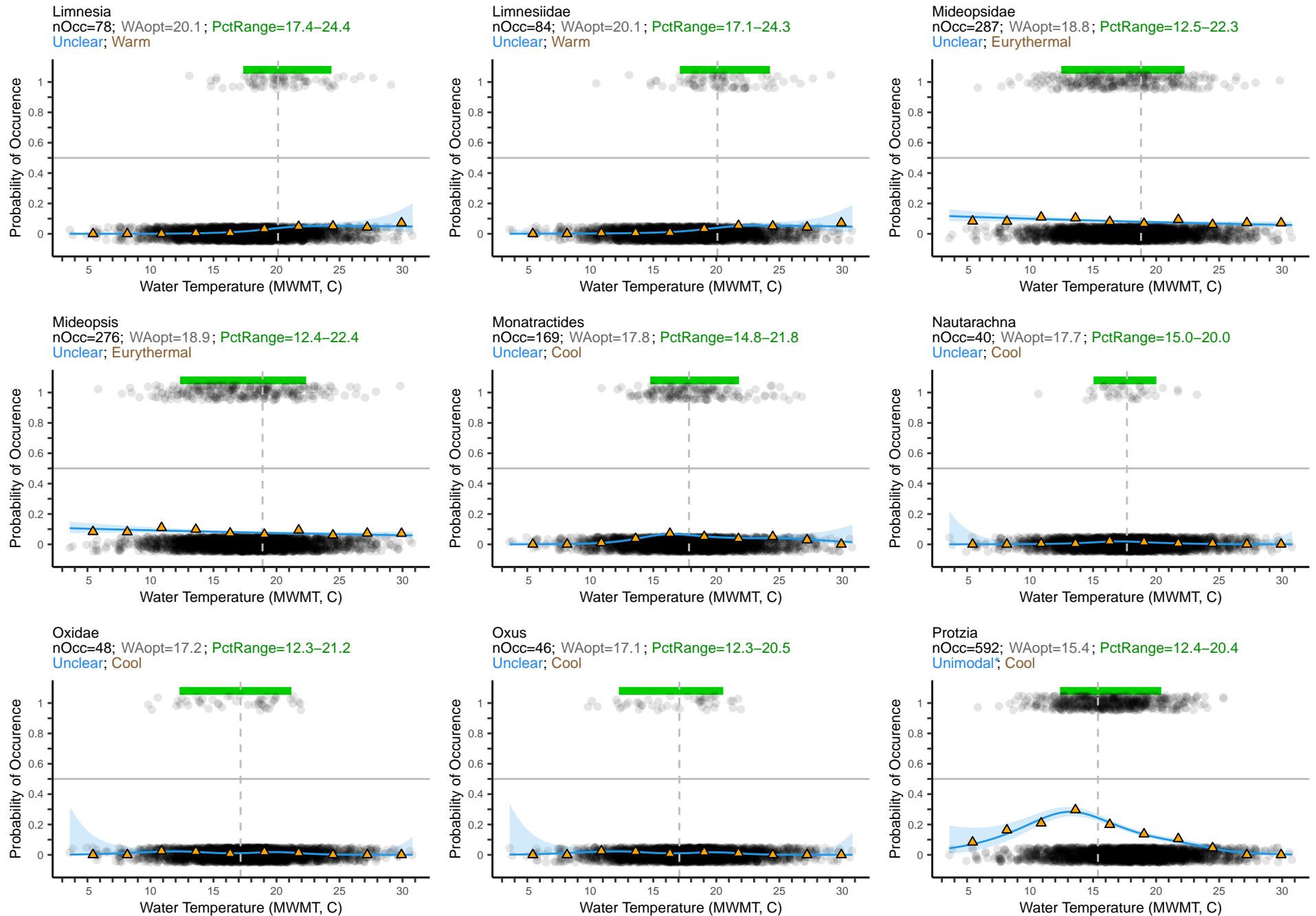

## NonInsect\_Mites

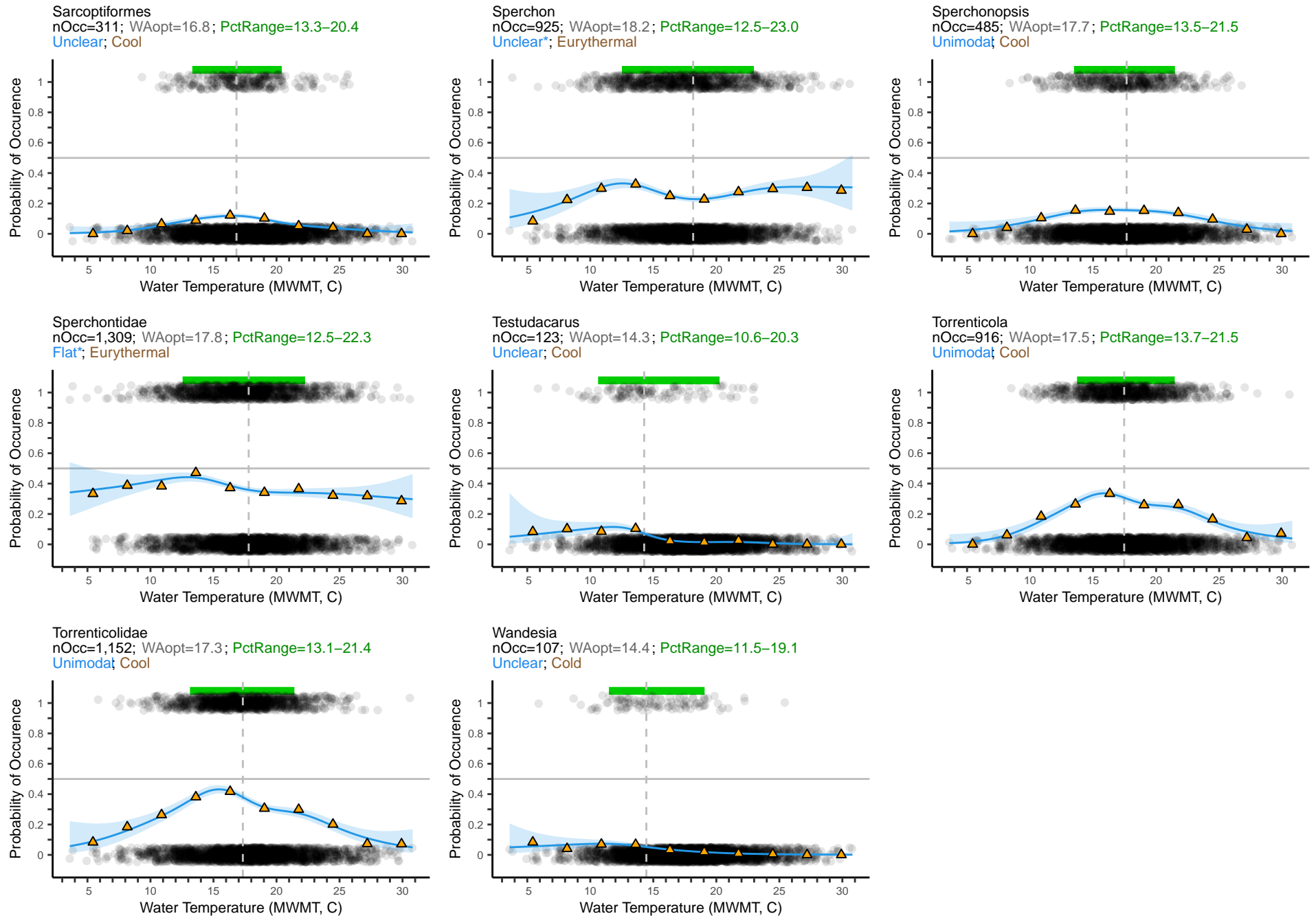

Supplement: Supplement15 [file NIHMS2055599-supplement-Supplement15.pdf]
