# Supplementary material for: Improved thermal preferences and a stressor index derived from modeled stream temperatures and regional taxonomic standards for freshwater macroinvertebrates of the Pacific Northwest, USA
Source: Ecol Indic. Author manuscript; Available in PMC 2025 Apr 9. (PMC11980781; doi:10.1016/j.ecolind.2024.111869)
Supplement: Supplement11 [file NIHMS2055599-supplement-Supplement11.docx]

Supplement 1

**Additional benthic macroinvertebrate data**

- Scatterplot of total number of individuals vs total number of taxa
- Full list of BMI data sources
- Map of BMI sampling sites, color-coded by data source
- List of taxa that were in the tolerance analysis dataset but occurred in fewer than 30 samples

Figure S1-1. Relationship between total number of individuals and total number of taxa. We did not see a need to subsample when total individuals exceeded the target of 500 organisms (red line = LOESS).

Table S1-1. Full list of BMI data sources.

| **Source** | **Source - collapsed** | **# Samples** | **Year - minimum** | **Year - maximum** |
| --- | --- | --- | --- | --- |
| EPA National Aquatic Resource Surveys (NARS) | EPA NARS | 159 | 2000 | 2014 |
| National Park Service (NPS) - Crater Lake | NPS | 25 | 2012 | 2018 |
| NPS - Mount Rainier |  | 70 | 2003 | 2006 |
| NPS - North Cascades |  | 71 | 1995 | 2016 |
| Oregon Department of Environmental Quality (ODEQ) | ODEQ | 1165 | 2000 | 2019 |
| City of Bainbridge Island | Puget Sound Benthos (PSSB) | 2 | 2017 | 2018 |
| City of Bellevue |  | 10 | 2012 | 2017 |
| City of Kirkland |  | 6 | 2015 | 2017 |
| City of Redmond |  | 12 | 2003 | 2018 |
| City of Sammamish |  | 1 | 2016 | 2016 |
| City of Seattle |  | 14 | 2011 | 2018 |
| Clallam County |  | 40 | 2011 | 2018 |
| King County - DNRP |  | 191 | 2010 | 2018 |
| Kitsap County |  | 55 | 2002 | 2018 |
| Pierce County |  | 18 | 2016 | 2018 |
| Skokomish Tribal Nation |  | 3 | 2006 | 2006 |
| Snohomish County |  | 57 | 2013 | 2018 |
| Stillaguamish Tribe |  | 3 | 2010 | 2011 |
| Vashon Nature Center |  | 8 | 2013 | 2018 |
| US Forest Service | USFS | 66 | 1998 | 2002 |
| BLM/USU National Aquatic Monitoring Center | BLM/USU National Aquatic Monitoring Center | 994 | 1991 | 2018 |
| Washington Department of Ecology (WSDOE) | WA ECY | 531 | 2009 | 2019 |
|  | ***Total*** | ***3501*** | ***1991*** | ***2019*** |


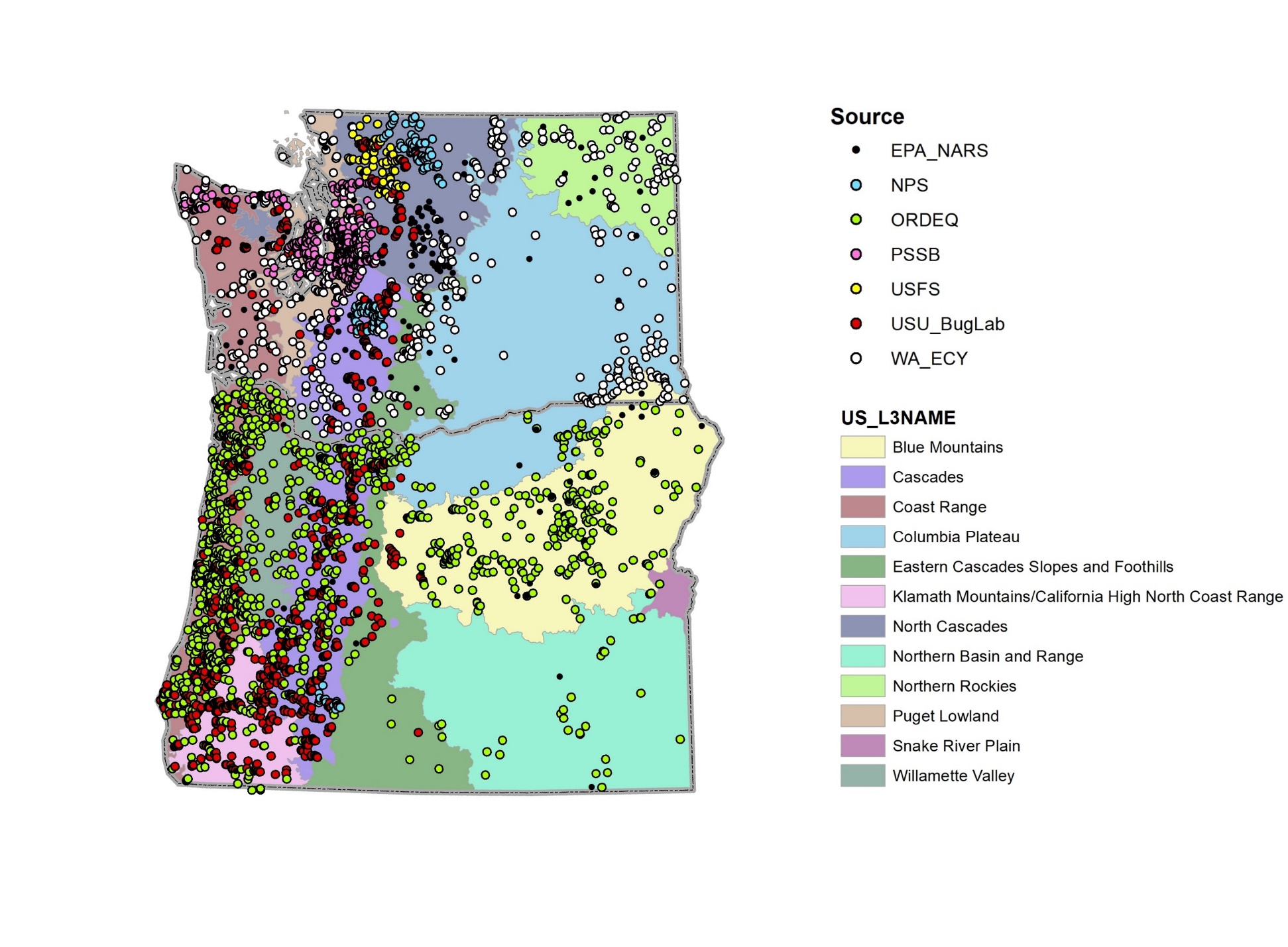


Figure S1-2. Macroinvertebrate sampling sites, color coded by source, with Omernik Level 3 ecoregion as the backdrop.

Table S1-2. List of taxa that were in the tolerance analysis dataset but occurred in fewer than 30 samples (thus, their results were not reported).

| **Taxon Group** | **Taxon** | **# Occurrences in dataset** |
| --- | --- | --- |
| Chironomidae | Acricotopus | 2 |
| Chironomidae | Allocladius | 3 |
| Chironomidae | Apsectrotanypus | 3 |
| Chironomidae | Bethbilbeckia | 5 |
| Chironomidae | Bilyjomyia | 29 |
| Chironomidae | Bryophaenocladius | 7 |
| Chironomidae | Cladopelma | 2 |
| Chironomidae | Clinotanypus | 1 |
| Chironomidae | Compterosmittia | 1 |
| Chironomidae | Cricotopus tremulus group | 9 |
| Chironomidae | Cryptotendipes | 15 |
| Chironomidae | Demicryptochironomus | 14 |
| Chironomidae | Derotanypus | 1 |
| Chironomidae | Doncricotopus | 5 |
| Chironomidae | Endochironomus | 4 |
| Chironomidae | Eretmoptera | 1 |
| Chironomidae | Eukiefferiella brevicalcar group | 11 |
| Chironomidae | Eukiefferiella coerulescens group | 22 |
| Chironomidae | Euryhapsis | 8 |
| Chironomidae | Glyptotendipes | 3 |
| Chironomidae | Gymnometriocnemus | 4 |
| Chironomidae | Helopelopia | 3 |
| Chironomidae | Heterotanytarsus | 29 |
| Chironomidae | Hydrosmittia | 2 |
| Chironomidae | Krenopelopia | 19 |
| Chironomidae | Krenopsectra | 1 |
| Chironomidae | Labrundinia | 13 |
| Chironomidae | Lauterborniella | 1 |
| Chironomidae | Meropelopia | 4 |
| Chironomidae | Microchironomus | 1 |
| Chironomidae | Monopelopia | 1 |
| Chironomidae | Nilothauma | 2 |
| Chironomidae | Orthocladius (Euorthocladius) | 5 |
| Chironomidae | Orthocladius (Mesorthocladius) | 1 |
| Chironomidae | Orthocladius (Orthocladius) | 1 |
| Chironomidae | Pagastiella | 4 |
| Chironomidae | Parachironomus | 6 |
| Chironomidae | Paracricotopus | 15 |
| Chironomidae | Paralauterborniella | 15 |
| Chironomidae | Parochlus | 5 |
| Chironomidae | Platysmittia | 18 |
| Chironomidae | Podonomini | 5 |
| Chironomidae | Potthastia longimanus group | 29 |
| Chironomidae | Protanypini | 2 |
| Chironomidae | Protanypus | 2 |
| Chironomidae | Psectrotanypus | 16 |
| Chironomidae | Pseudochironomini | 28 |
| Chironomidae | Pseudochironomus | 28 |
| Chironomidae | Pseudorthocladius | 12 |
| Chironomidae | Rheopelopia | 23 |
| Chironomidae | Rheosmittia | 13 |
| Chironomidae | Robackia | 16 |
| Chironomidae | Saetheria | 4 |
| Chironomidae | Sergentia | 6 |
| Chironomidae | Smittia | 11 |
| Chironomidae | Stenochironomus | 27 |
| Chironomidae | Symbiocladius | 3 |
| Chironomidae | Sympotthastia | 2 |
| Chironomidae | Tanypodini | 10 |
| Chironomidae | Tanypus | 10 |
| Chironomidae | Thienemannia | 3 |
| Chironomidae | Tokunagaia | 3 |
| Chironomidae | Tvetenia discoloripes group | 22 |
| Chironomidae | Xenochironomus | 3 |
| Chironomidae | Zavrelia | 2 |
| Coleoptera | Acilius | 1 |
| Coleoptera | Agabinus | 4 |
| Coleoptera | Amphizoa | 17 |
| Coleoptera | Amphizoidae | 17 |
| Coleoptera | Anacaena | 21 |
| Coleoptera | Araeopidius | 2 |
| Coleoptera | Araeopidius monachus | 2 |
| Coleoptera | Atractelmis | 4 |
| Coleoptera | Berosus | 3 |
| Coleoptera | Boreonectes | 7 |
| Coleoptera | Coptotomus | 1 |
| Coleoptera | Crenitis | 8 |
| Coleoptera | Cymbiodyta | 3 |
| Coleoptera | Dryopidae | 12 |
| Coleoptera | Enochrus | 8 |
| Coleoptera | Gyrinidae | 19 |
| Coleoptera | Haliplus | 18 |
| Coleoptera | Helichus | 12 |
| Coleoptera | Helochares | 3 |
| Coleoptera | Helophoridae | 18 |
| Coleoptera | Helophorus | 18 |
| Coleoptera | Heterosternuta | 2 |
| Coleoptera | Hydrobius | 14 |
| Coleoptera | Hydrochidae | 5 |
| Coleoptera | Hydrochus | 5 |
| Coleoptera | Laccobius | 11 |
| Coleoptera | Laccophilus | 3 |
| Coleoptera | Lutrochidae | 1 |
| Coleoptera | Lutrochus | 1 |
| Coleoptera | Neoporus | 2 |
| Coleoptera | Paracymus | 7 |
| Coleoptera | Peltodytes | 8 |
| Coleoptera | Ptilodactylidae | 4 |
| Coleoptera | Rhizelmis | 24 |
| Coleoptera | Scirtidae | 6 |
| Coleoptera | Stenelmis | 4 |
| Coleoptera | Tropisternus | 11 |
| Diptera_NotChiros | Agathon | 14 |
| Diptera_NotChiros | Anopheles | 7 |
| Diptera_NotChiros | Austrolimnophila | 2 |
| Diptera_NotChiros | Bibiocephala | 8 |
| Diptera_NotChiros | Blepharicera | 15 |
| Diptera_NotChiros | Chaoboridae | 12 |
| Diptera_NotChiros | Chaoborus | 1 |
| Diptera_NotChiros | Cheilotrichia | 1 |
| Diptera_NotChiros | Culicidae | 20 |
| Diptera_NotChiros | Dactylolabis | 1 |
| Diptera_NotChiros | Deuterophlebia | 23 |
| Diptera_NotChiros | Deuterophlebiidae | 23 |
| Diptera_NotChiros | Dolichopodidae | 26 |
| Diptera_NotChiros | Empididae Genus A | 4 |
| Diptera_NotChiros | Erioptera | 13 |
| Diptera_NotChiros | Eucorethra | 8 |
| Diptera_NotChiros | Euparyphus | 9 |
| Diptera_NotChiros | Gonomyia | 3 |
| Diptera_NotChiros | Gonomyodes | 1 |
| Diptera_NotChiros | Helius | 6 |
| Diptera_NotChiros | Holorusia | 4 |
| Diptera_NotChiros | Lipsothrix | 1 |
| Diptera_NotChiros | Metacnephia | 1 |
| Diptera_NotChiros | Molophilus | 23 |
| Diptera_NotChiros | Nemotelus | 5 |
| Diptera_NotChiros | Odontomyia | 2 |
| Diptera_NotChiros | Ormosia | 18 |
| Diptera_NotChiros | Paradelphomyia | 1 |
| Diptera_NotChiros | Pediciidae | 23 |
| Diptera_NotChiros | Pilaria | 15 |
| Diptera_NotChiros | Pseudolimnophila | 2 |
| Diptera_NotChiros | Psychodini | 9 |
| Diptera_NotChiros | Rhabdomastix (Lurdia) | 22 |
| Diptera_NotChiros | Sciomyzidae | 24 |
| Diptera_NotChiros | Stegopterna | 2 |
| Diptera_NotChiros | Syrphidae | 1 |
| Diptera_NotChiros | Tanyderidae | 5 |
| Diptera_NotChiros | Twinnia | 2 |
| Diptera_NotChiros | Ulomorpha | 8 |
| Ephemeroptera | Acerpenna | 2 |
| Ephemeroptera | Acerpenna pygmaea | 2 |
| Ephemeroptera | Asioplax | 9 |
| Ephemeroptera | Camelobaetidius | 4 |
| Ephemeroptera | Caudatella cascadia | 3 |
| Ephemeroptera | Caudatella edmundsi | 19 |
| Ephemeroptera | Caudatella jacobi | 11 |
| Ephemeroptera | Choroterpes | 2 |
| Ephemeroptera | Drunella pelosa | 10 |
| Ephemeroptera | Ephemera | 3 |
| Ephemeroptera | Ephemerella alleni | 22 |
| Ephemeroptera | Ephemeridae | 9 |
| Ephemeroptera | Ephoron | 4 |
| Ephemeroptera | Eurylophella | 1 |
| Ephemeroptera | Hexagenia | 4 |
| Ephemeroptera | Isonychia | 11 |
| Ephemeroptera | Isonychiidae | 11 |
| Ephemeroptera | Iswaeon | 14 |
| Ephemeroptera | Leucrocuta | 14 |
| Ephemeroptera | Maccaffertium | 11 |
| Ephemeroptera | Paracloeodes | 3 |
| Ephemeroptera | Polymitarcyidae | 4 |
| Ephemeroptera | Serratella | 25 |
| Ephemeroptera | Serratella levis | 5 |
| Ephemeroptera | Serratella micheneri | 20 |
| Ephemeroptera | Siphlonuridae | 3 |
| Ephemeroptera | Siphlonurus | 2 |
| Ephemeroptera | Stenacron | 1 |
| Insect_Other | Aeshna/Rhionaeschna | 15 |
| Insect_Other | Anax | 1 |
| Insect_Other | Archilestes | 1 |
| Insect_Other | Calopterygidae | 5 |
| Insect_Other | Calopteryx | 5 |
| Insect_Other | Corduliidae | 2 |
| Insect_Other | Dysmicohermes | 2 |
| Insect_Other | Erpetogomphus | 1 |
| Insect_Other | Gomphus | 2 |
| Insect_Other | Lestidae | 1 |
| Insect_Other | Libellulidae | 6 |
| Insect_Other | Macromia | 1 |
| Insect_Other | Macromiidae | 1 |
| NonInsect_Mites | Albertathyas | 4 |
| NonInsect_Mites | Albertathyas montana | 1 |
| NonInsect_Mites | Anisitsiellidae | 5 |
| NonInsect_Mites | Aturidae | 15 |
| NonInsect_Mites | Aturus | 12 |
| NonInsect_Mites | Bandakiopsis | 1 |
| NonInsect_Mites | Brachypoda | 3 |
| NonInsect_Mites | Chelomideopsis | 6 |
| NonInsect_Mites | Corticacarus | 1 |
| NonInsect_Mites | Eylaidae | 2 |
| NonInsect_Mites | Eylais | 2 |
| NonInsect_Mites | Feltria | 1 |
| NonInsect_Mites | Feltriidae | 1 |
| NonInsect_Mites | Forelia | 1 |
| NonInsect_Mites | Gereckea | 2 |
| NonInsect_Mites | Hydrachna | 3 |
| NonInsect_Mites | Hydrachnidae | 3 |
| NonInsect_Mites | Hydrodroma | 5 |
| NonInsect_Mites | Hydrodromidae | 5 |
| NonInsect_Mites | Hydrovolzia | 15 |
| NonInsect_Mites | Hydrovolziidae | 16 |
| NonInsect_Mites | Hydryphantes | 1 |
| NonInsect_Mites | Koenikea | 4 |
| NonInsect_Mites | Limnochares | 11 |
| NonInsect_Mites | Limnocharidae | 11 |
| NonInsect_Mites | Mesobates | 11 |
| NonInsect_Mites | Midea | 1 |
| NonInsect_Mites | Mideidae | 1 |
| NonInsect_Mites | Momoniidae | 3 |
| NonInsect_Mites | Najadicola | 1 |
| NonInsect_Mites | Neoacaridae | 1 |
| NonInsect_Mites | Neumania | 6 |
| NonInsect_Mites | Panisopsis | 2 |
| NonInsect_Mites | Panisus | 1 |
| NonInsect_Mites | Parathyas | 1 |
| NonInsect_Mites | Partnunia | 1 |
| NonInsect_Mites | Piona | 9 |
| NonInsect_Mites | Pionacercus | 1 |
| NonInsect_Mites | Pionidae | 21 |
| NonInsect_Mites | Platyhydracarus | 27 |
| NonInsect_Mites | Pseudofeltria | 3 |
| NonInsect_Mites | Stygomomonia | 3 |
| NonInsect_Mites | Stygothrombiidae | 9 |
| NonInsect_Mites | Stygothrombium | 7 |
| NonInsect_Mites | Tartarothyas | 2 |
| NonInsect_Mites | Thyas | 2 |
| NonInsect_Mites | Thyasidae | 2 |
| NonInsect_Mites | Thyopsella | 8 |
| NonInsect_Mites | Thyopsis | 3 |
| NonInsect_Mites | Unionicola | 4 |
| NonInsect_Mites | Unionicolidae | 15 |
| NonInsect_Mites | Utaxatax | 4 |
| NonInsect_Mites | Volsellacarus | 1 |
| NonInsect_Mites | Xystonotus | 6 |
| NonInsect_NotMites | Achaeta | 8 |
| NonInsect_NotMites | Altmanella | 3 |
| NonInsect_NotMites | Americorophium | 5 |
| NonInsect_NotMites | Bothrioneurum | 3 |
| NonInsect_NotMites | Cernosvitoviella | 16 |
| NonInsect_NotMites | Chaetogaster | 4 |
| NonInsect_NotMites | Corbicula | 10 |
| NonInsect_NotMites | Corbiculidae | 10 |
| NonInsect_NotMites | Corophiidae | 5 |
| NonInsect_NotMites | Cystobranchus | 11 |
| NonInsect_NotMites | Cystobranchus salmositicus | 11 |
| NonInsect_NotMites | Dero | 11 |
| NonInsect_NotMites | Eiseniella | 4 |
| NonInsect_NotMites | Eiseniella tetraedra | 4 |
| NonInsect_NotMites | Glossiphonia | 9 |
| NonInsect_NotMites | Guestphalinus | 2 |
| NonInsect_NotMites | Haplotaxis | 16 |
| NonInsect_NotMites | Helisoma | 2 |
| NonInsect_NotMites | Helobdella | 27 |
| NonInsect_NotMites | Henlea | 24 |
| NonInsect_NotMites | Kincaidiana | 8 |
| NonInsect_NotMites | Lanx | 2 |
| NonInsect_NotMites | Limnodrilus | 22 |
| NonInsect_NotMites | Lumbricidae | 10 |
| NonInsect_NotMites | Margaritiferidae | 24 |
| NonInsect_NotMites | Musculium | 12 |
| NonInsect_NotMites | Nematomorpha | 14 |
| NonInsect_NotMites | Nootkadrilus | 1 |
| NonInsect_NotMites | Ophidonais | 20 |
| NonInsect_NotMites | Paranais | 1 |
| NonInsect_NotMites | Piscicolidae | 16 |
| NonInsect_NotMites | Planorbella | 9 |
| NonInsect_NotMites | Potamopyrgus | 18 |
| NonInsect_NotMites | Potamopyrgus antipodarum | 18 |
| NonInsect_NotMites | Potamothrix | 2 |
| NonInsect_NotMites | Quistradrilus | 2 |
| NonInsect_NotMites | Radix | 7 |
| NonInsect_NotMites | Rhyacodrilinae | 22 |
| NonInsect_NotMites | Rhyacodrilus | 19 |
| NonInsect_NotMites | Slavina | 11 |
| NonInsect_NotMites | Specaria | 3 |
| NonInsect_NotMites | Sphaerium | 16 |
| NonInsect_NotMites | Stygobromus | 10 |
| NonInsect_NotMites | Stylaria | 4 |
| NonInsect_NotMites | Telmatodrilus | 19 |
| NonInsect_NotMites | Telmatodrilus vejdovskyi | 19 |
| NonInsect_NotMites | Tubifex | 1 |
| NonInsect_NotMites | Unionoida | 28 |
| NonInsect_NotMites | Unionoidea | 28 |
| NonInsect_NotMites | Valvata | 12 |
| NonInsect_NotMites | Valvatidae | 12 |
| NonInsect_NotMites | Varichaetadrilus | 2 |
| NonInsect_NotMites | Vejdovskyella | 2 |
| NonInsect_NotMites | Vorticifex | 14 |
| Plecoptera | Alloperla | 2 |
| Plecoptera | Bolshecapnia | 1 |
| Plecoptera | Calliperla | 13 |
| Plecoptera | Cultus | 18 |
| Plecoptera | Diura | 18 |
| Plecoptera | Frisonia | 17 |
| Plecoptera | Isogenoides | 5 |
| Plecoptera | Perlomyia | 1 |
| Plecoptera | Podmosta | 1 |
| Plecoptera | Pteronarcys dorsata | 2 |
| Plecoptera | Salmoperla | 3 |
| Plecoptera | Salmoperla sylvanica | 3 |
| Plecoptera | Sierraperla | 22 |
| Plecoptera | Taeniopteryx | 15 |
| Trichoptera | Allomyia | 15 |
| Trichoptera | Amphicosmoecus | 1 |
| Trichoptera | Arctopsyche californica | 1 |
| Trichoptera | Arctopsyche grandis | 3 |
| Trichoptera | Ceraclea | 7 |
| Trichoptera | Chyrandra | 19 |
| Trichoptera | Clostoeca | 4 |
| Trichoptera | Culoptila | 1 |
| Trichoptera | Eocosmoecus | 3 |
| Trichoptera | Eocosmoecus frontalis | 2 |
| Trichoptera | Goeracea | 11 |
| Trichoptera | Hesperophylax | 11 |
| Trichoptera | Himalopsyche | 9 |
| Trichoptera | Homophylax | 7 |
| Trichoptera | Homoplectra | 1 |
| Trichoptera | Limnephilus | 2 |
| Trichoptera | Marilia | 3 |
| Trichoptera | Monophylax | 6 |
| Trichoptera | Moselyana | 1 |
| Trichoptera | Namamyia | 2 |
| Trichoptera | Nectopsyche | 27 |
| Trichoptera | Neophylax occidentis | 20 |
| Trichoptera | Neotrichia | 17 |
| Trichoptera | Nerophilus | 8 |
| Trichoptera | Neureclipsis | 1 |
| Trichoptera | Odontoceridae | 16 |
| Trichoptera | Parthina | 1 |
| Trichoptera | Philocasca | 5 |
| Trichoptera | Phryganeidae | 6 |
| Trichoptera | Pseudostenophylax | 15 |
| Trichoptera | Psychoglypha bella | 3 |
| Trichoptera | Psychoglypha subborealis | 2 |
| Trichoptera | Ptilostomis | 1 |
| Trichoptera | Rhyacophila basalis group | 2 |
| Trichoptera | Rhyacophila chilsia | 1 |
| Trichoptera | Rhyacophila coloradensis group | 24 |
| Trichoptera | Rhyacophila nevadensis group | 16 |
| Trichoptera | Rhyacophila oreta group | 3 |
| Trichoptera | Rhyacophila rotunda group | 13 |
| Trichoptera | Rhyacophila viquaea group | 2 |
| Trichoptera | Stactobiella | 1 |
| Trichoptera | Thremmatidae | 19 |
| Trichoptera | Tinodes | 8 |
| Trichoptera | Triaenodes | 2 |
| Trichoptera | Yphria | 1 |
