# Supplementary material for: Improved thermal preferences and a stressor index derived from modeled stream temperatures and regional taxonomic standards for freshwater macroinvertebrates of the Pacific Northwest, USA
Source: Ecol Indic. Author manuscript; Available in PMC 2025 Apr 9. (PMC11980781; doi:10.1016/j.ecolind.2024.111869)
Supplement: Supplement12 [file NIHMS2055599-supplement-Supplement12.docx]

**APPENDIX A**

Assigning macroinvertebrate taxa to thermal preference categories

**Macroinvertebrate-MWMT relationship**

We based thermal preference assignments on a combination of the following three thermal response metrics:

- central tendency, based on weighted average optima calculations and relative abundance data
- lower and upper thermal limits, based on the 10th and 90^th^ percentiles of taxon occurrence
- thermal response shape, based on Generalized Additive Model (GAM) plots (Hastie and Tibshirani 1999)

**Weighted average optima** is commonly used for estimating the central tendency of a taxon along an environmental gradient (ter Braak and Looman 1986, Yuan 2006). Values were calculated with the “analogue” R package (Simpson 2007) using the formula

$$WAopt_{j}=\frac{sum\left( MWMT_{i}*RA_{j} \right)}{sum\left( RA_{j} \right)}$$

where *i* is the sample index, *j* is the taxa index, and *RA* is the relative abundance. The calculation involves multiplying MWMT by taxon relative abundance (the weighting factor) for each sample, summing the resulting products, then dividing that by the sum of all the relative abundances (weights).

**The lower and upper thermal limits** were calculated based on presence data. We selected the 10^th^ and 90^th^ percentiles to exclude potential outliers (despite our QC procedures, there are likely occasional inaccuracies in the dataset, such as misidentification of a macroinvertebrate taxon, or modeled temperature being off at a site with unique characteristics). We used the thermal limits to help identify taxa that were likely to disappear with increased thermal stress (sensitive) and those that were able to persist in thermally altered environments (tolerant).

For **thermal response shape**, we used GAM plots to evaluate curve shapes (Yuan 2006) and probability of each taxon’s occurrence across the MWMT gradient using the formula

$$Probability\left( Presence_{i} \right)=Intercept+s\left( MWMT_{i} \right)+e_{i}$$

where *i* is the sample index, *Intercept* is an estimated parameter, *s(.)* is an estimated nonlinear smoothing function, and *e* is the residual error. Plots were fitted with the “mgcv” R package (Wood 2004). The GAM plots are independent of the weighted average optima calculations and are based on presence/absence data instead of relative abundance data. GAMs are an extension of multiple linear regression, where the relationship between the response and predictor can be nonlinear. We grouped shapes of the modeled curve lines into seven categories (Figure A1), as follows:

1. Decreaser: lower confidence bounds (LCB) at minimum temperature > upper confidence bounds (UCB) at maximum temperature
2. Increaser. LCB at maximum temperature > UCB at minimum temperature
3. Unimodal: maximum probability of occurrence (POC) > POC at both the maximum and minimum temperatures
4. Uni-decreaser: exhibits traits of both unimodal and decreaser
5. Uni-increaser: exhibits traits of both unimodal and increaser
6. Flat: range of POC < 0.02.

- Unclear: maximum POC values (< 0.15) when confidence interval widths were <0.1 OR identified as both "Decreaser/Increaser" (see Heptageniida - Rhithrogena in Figure A1 as an example).

To aid in interpretation of results, we customized the GAM plots by overlaying a vertical line showing the weighted average optima, a line showing the range of the 10^th^ to 90^th^ percentiles and shading to show uncertainty, as measured by 90% confidence intervals (Figure A2).


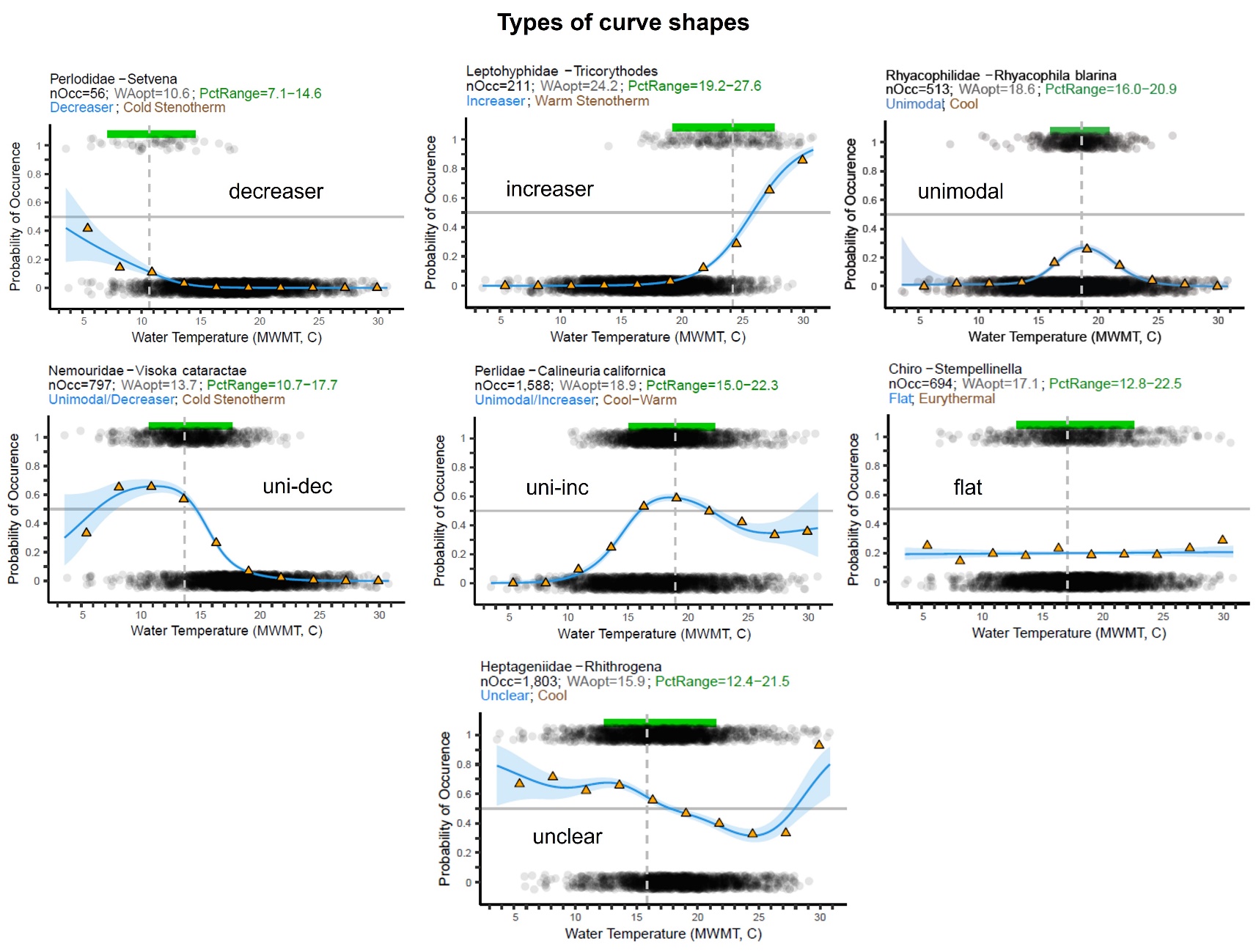


Figure A1. Examples of each type of thermal response shape: decreaser, increaser, unimodal, uni-decreaser, uni-increaser, flat and unclear.

**
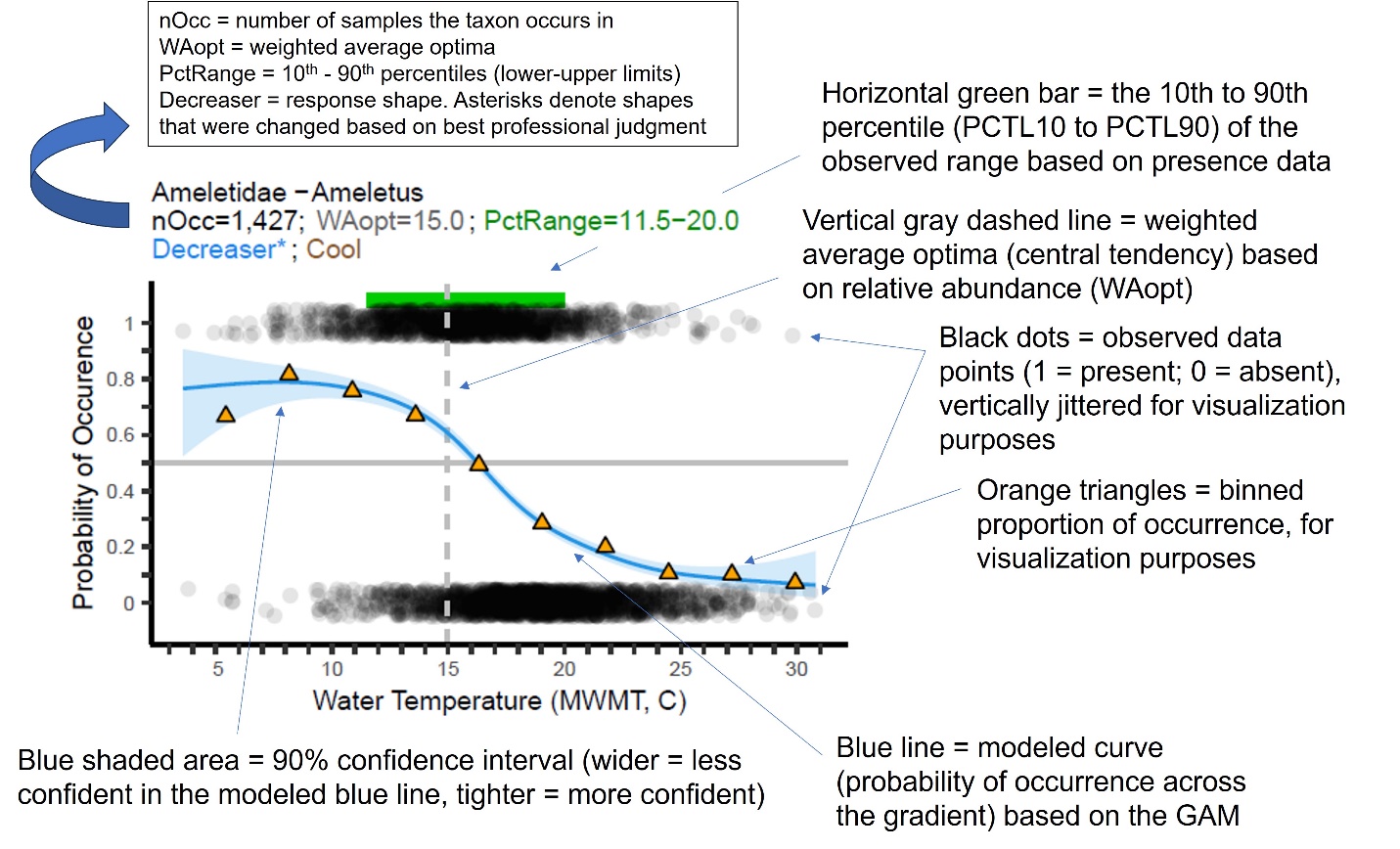
**

Figure A2. General additive model (GAM) plot interpretation guide. We overlaid a vertical line showing the weighted average optima to make it easier to evaluate the relative abundance-based metrics in combination with presence/absence-based metrics when making assessments. The number of samples in which the taxon was detected is indicated by nOcc.

**Thermal preference categories**

When deciding how many thermal preference categories to use and where to establish thresholds, we initially set up four categories (cold stenotherm, cold, cool and warm) that linked to the numeric thresholds being used in water temperature standards aimed at protecting salmonid habitat in Oregon and Washington (Table A1; Sturdevant 2008, Washington State 2020). The standards in both states are based on the 7-day moving averages of the daily maximum temperatures (metrics equivalent to MWMT). After reviewing hundreds of GAM plots, we started to recognize patterns that warranted three additional categories (eurythermal, warm stenotherm, and cool/warm). Eurythermal taxa occurred across a wide thermal gradient, spanning cold to warm streams. Warm stenotherms were ‘increaser’ taxa that occurred over a narrow range of the warmest streams (> 23°C). Cool/warm taxa straddled the 20°C threshold and had a more limited range than eurythermal taxa, dropping out in the coldest and warmest streams.

Table A1. Summary of Oregon and Washington regulatory temperature standards (Sturdevant 2008, Washington State 2020).

| Oregon | | Washington | |
| --- | --- | --- | --- |
| Beneficial Use | 7-day average maximum (°C) | Category | Highest 7-DADMax (°C)* |
| Bull trout spawning & juvenile rearing | 12°C | Char Spawning and Rearing | 12°C |
| Salmon & steelhead spawning | 13°C |  | |
| Core coldwater habitat | 16°C | Core Summer Salmonid Habitat | 16°C |
|  |  | Salmonid Spawning, Rearing, and Migration | 17.5°C |
|  |  | Salmonid Rearing and Migration Only |  |
| Salmon & trout rearing & migration | 18°C | Non-anadromous Interior Redband Trout | 18°C |
| Migration corridor (salmon & steelhead) | 20°C | Indigenous Warm Water Species | 20°C |
| Lahontan cutthroat or redband trout |  |  |  |

^*^7-DADMax = 7-day average of the daily maximum temperatures

**Making thermal preference assignments**

Assignments were made for taxa occurring in 30 or more samples. Results for 521 taxa are shown in Appendix B. Initial assignments were based on a combination of the thermal response metrics, per the quantitative criteria in Table 1 in the main article (included below). We wrote customized R code to automate the thermal preference assignments. If taxa met criteria for more than one category, they were assigned to the last thermal category they met, in this order: Cool-Warm, Cool, Cold, Cold Stenotherm, Warm, Warm Stenotherm, Eurythermal. Criteria were applied consistently and transparently to all taxa, regardless of whether the taxon was ‘borderline’ between two categories versus clearly in one group. There were five instances when taxa did not meet criteria for any of the seven categories. These taxa were marked as ‘inconclusive’ and assigned to categories by regional biologists. All other assignments were reviewed by regional biologists as well. Final designations were changed if at least three biologists agreed on the change. In total, 12 taxa (including the five ‘inconclusives’) had assignments that were changed. These taxa are marked in Appendix B in the ‘BPJ overrule’ column.

[Table 1 from main article]. Rules for assigning taxa to the seven thermal preference categories, using the three sets of thermal response metrics (weighted average optima, 10^th^/90^th^ percentiles, and GAM thermal response shapes) in combination.

| **Thermal category** | **MWMT (°C)** | | | **GAM thermal response shape** | **Indicator narrative** |
| --- | --- | --- | --- | --- | --- |
|  | **Weighted average optima** | **Percentiles** | |  |  |
|  |  | **10^th^** | **90^th^** |  |  |
| Cold stenotherm | < 16 | ≤ 12 | ≤ 18 | not an increaser or uni-increaser | Very cold habitat suitable for bull trout adult/sub-adult use |
| Cold | < 18 | ≤ 14 | ≤ 20 |  | Suitable for salmon and steelhead rearing |
| Cool | < 20 | ≤ 16 | ≤ 22 |  | Suitable for inland resident trout and cool water fish species (non-salmonid) |
| Cool-Warm | > 17.5 < 21.5 | > 14 | < 25 | not an increaser or decreaser | Inconclusive from a management standpoint; straddle the 20°C threshold |
| Warm | > 19 | > 14.5 | > 23.5 | not a decreaser or uni-decreaser | Suitable for certain indigenous warm water species |
| Warm stenotherm | > 23 | ≥ 19 | ≥ 26 |  | Very warm habitat |
| Eurythermal | -- | ≤ 14.5 | ≥ 22 | -- | Inconclusive from a management standpoint; ubiquitous across a wide thermal gradient |

**Flags**

We added flags to Appendix B to mark taxa that had thermal variability and potential source bias. For thermal variability, we flagged any genus or coarser-level taxa that had ‘children’ that spanned more than one thermal preference category (Appendix C). For potential source bias, we flagged taxa if they were missing in the ODEQ, WSDOE and NAMC datasets, which together comprised over 60% of the samples in our dataset and cover wide-ranging spatial distributions and thermal gradients (Supplement 1). Chironomidae (Diptera), Acari (Trombidiformes and Sarcoptiformes) and Oligochaeta worms were particularly affected. For example, with Chironomidae, some datasets had mostly subfamily-level identifications for earlier time periods, which could potentially affect the species-, genus- and tribal-level results by truncating spatial distributions and temperature gradients.

**References**

Hastie, T.J. and R.J. Tibshirani. 1999. Generalized additive models. Washington, DC: Chapman & Hall/CRC.

Hayslip, G. editor. 2007. Methods for the collection and analysis of benthic macroinvertebrate assemblages in wadeable streams of the Pacific Northwest. Pacific Northwest Aquatic Monitoring Partnership, Cook, Washington. <https://s3.us-west-2.amazonaws.com/prod-is-cms-assets/pnamp/prod/2007_0612PNAMP_macroinvert_protocol_final.pdf>

R Core Team. 2022. R: A language and environment for statistical computing. R Foundation for Statistical Computing, Vienna, Austria. URL https://www.R-project.org/.

Simpson, G.L. 2007. Analogue Methods in Palaeoecology: Using the analogue Package Journal of Statistical Software 22(2): 1-29.

Sturdevant, D. 2008. Temperature Water Quality Standard Implementation – Oregon Department of Environmental Quality Internal Management Directive. Available online: <https://www.oregon.gov/deq/Filtered%20Library/IMDTemperature.pdf>

ter Braak, C.J.F and C.W.N. Looman. 1986. Weighted averaging, logistic regression and the Gaussian response model. Vegetatio 65: 3–11.

Washington State. 2020. Table 200 (1)(c) Aquatic Life Temperature Criteria in Fresh Water. Available online: <https://apps.leg.wa.gov/WAC/default.aspx?cite=173-201A-200&pdf=true>

Wisseman, R., Sullivan, S., Pfeiffer, J. and S. Salter. 2015. Northwest Standard Taxonomic Effort.

https://www.pnamp.org/project/northwest-standard-taxonomic-effort

Wood, S.N. 2004. Stable and efficient multiple smoothing parameter estimation for generalized additive models. Journal of the American Statistical Association 99: 673-686.

Yuan, L. 2006. Estimation and Application of Macroinvertebrate Tolerance Values. Report No. EPA/600/P-04/116F. National Center for Environmental Assessment, Office of Research and Development, U.S. Environmental Protection Agency, Washington, D.C.
