# Supplementary material for: Improved thermal preferences and a stressor index derived from modeled stream temperatures and regional taxonomic standards for freshwater macroinvertebrates of the Pacific Northwest, USA
Source: Ecol Indic. Author manuscript; Available in PMC 2025 Apr 9. (PMC11980781; doi:10.1016/j.ecolind.2024.111869)
Supplement: Supplement13 [file NIHMS2055599-supplement-Supplement13.docx]

Supplement 4

# Macroinvertebrate Thermal Tolerance Index (MTTI) - additional supporting information

- Calibration (CAL) and Validation (VAL) datasets
  - Summary Statistics
  - Boxplots of natural and disturbance variables
  - PCA biplots
- Modeling techniques
  - deshrinking and tolerance downweighting
- Comparisons between field temperature and modeled temperatures (MWMT, MTTI)
- Correlations with other stressors

# Comparing the Calibration (CAL) and Validation (VAL) datasets

Below are supporting data showing comparisons of the two datasets used for the MTTI. The calibration dataset (CAL, n = 2891) was used to build the MTTI model. The validation dataset (VAL, n = 319) was used as an independent assessment of the ability of the MTTI to assess novel datasets across our study area.

Table S4-1. Environmental and disturbance metrics for the calibration (CAL) and validation (VAL) datasets.

| Variable | Metric name | Dataset | q1 | median | q3 | Range |
| --- | --- | --- | --- | --- | --- | --- |
| Stream Temperature (oC) | MWMT ^1^ | CAL | 15.4 | 17.8 | 20.1 | 3.6 - 30.8 |
|  |  | VAL | 15.5 | 18 | 20 | 7.5 - 29.7 |
| Air Temperature (oC) | Tmean8110Ws ^2^ | CAL | 6.5 | 9 | 10.7 | 0 - 12.2 |
|  |  | VAL | 6.3 | 9.1 | 10.8 | 1.8 - 12 |
| Precipitation (mm) | Precip8110Ws ^2,^ ^4^ | CAL | 1072 | 1644 | 2239 | 207 - 4948 |
|  |  | VAL | 1051 | 1631 | 2284 | 298 - 4363 |
| Elevation (m) | ElevWS ^2, 4^ | CAL | 314 | 773 | 1265 | 26 - 2348 |
|  |  | VAL | 297 | 774 | 1265 | 44 - 2535 |
| Watershed Slope (%) | SLOPE ^3, 5^ | CAL | 1.21 | 2.98 | 6.41 | 0 - 53 |
|  |  | VAL | 1.33 | 2.89 | 6.81 | 0 - 33.87 |
| Watershed Area (km2) | WsAreaSqKm ^2, 5^ | CAL | 7 | 18 | 52 | 0.7 - 14318 |
|  |  | VAL | 8 | 19 | 55 | 1 – 192542* |
| Index of Watershed Integrity | IWI ^2^ | CAL | 0.79 | 0.85 | 0.88 | 0.15 - 0.93 |
|  |  | VAL | 0.8 | 0.85 | 0.88 | 0.2 - 0.92 |
| Collection Day of Year | daynum | CAL | 211 | 230 | 252 | 156 - 304 |
|  |  | VAL | 208 | 228 | 245 | 157 - 287 |
| Year of Collection | Year | CAL | 2005 | 2010 | 2015 | 1991 - 2019 |
|  |  | VAL | 2004 | 2010 | 2015 | 1993 - 2019 |

* Maximum value was deemed a mapping error.

Data sources: ^1^ = NorWeST (Isaak et al. 2017), ^2^ = StreamCat (Hill et al. 2016), ^3^ = NHDPlusV2 (McKay et al. 2012)

PCA transformations: ^4^ = square root, ^5^ = log10


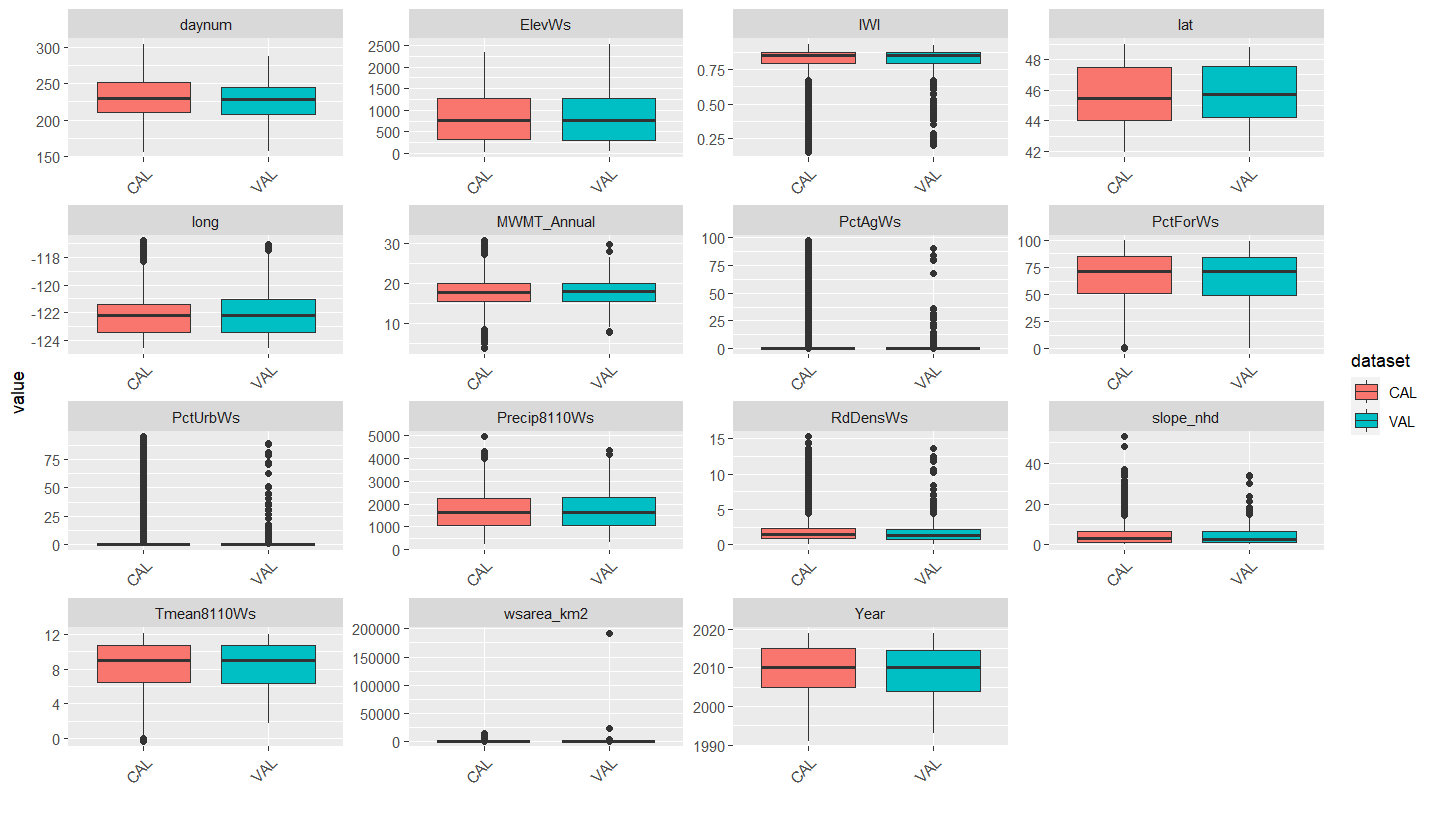

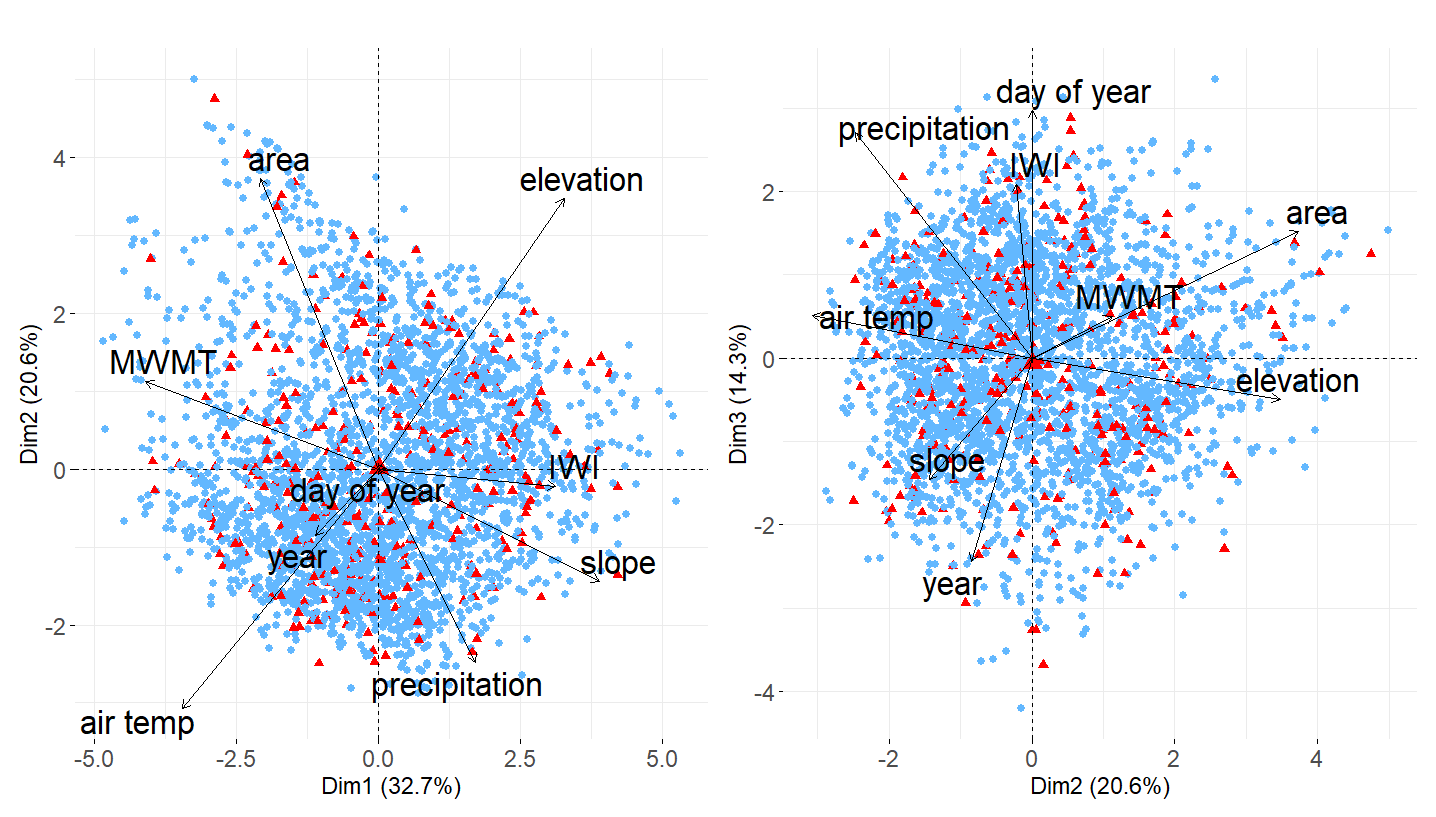


Figure S4-1. Boxplots comparing natural and disturbance gradient metrics for the Calibration (CAL) and Validation (VAL) datasets. Horizontal lines represent medians, boxes represent the upper and lower quartile range, vertical lines represent non-outlier ranges, and black circles represent outliers.


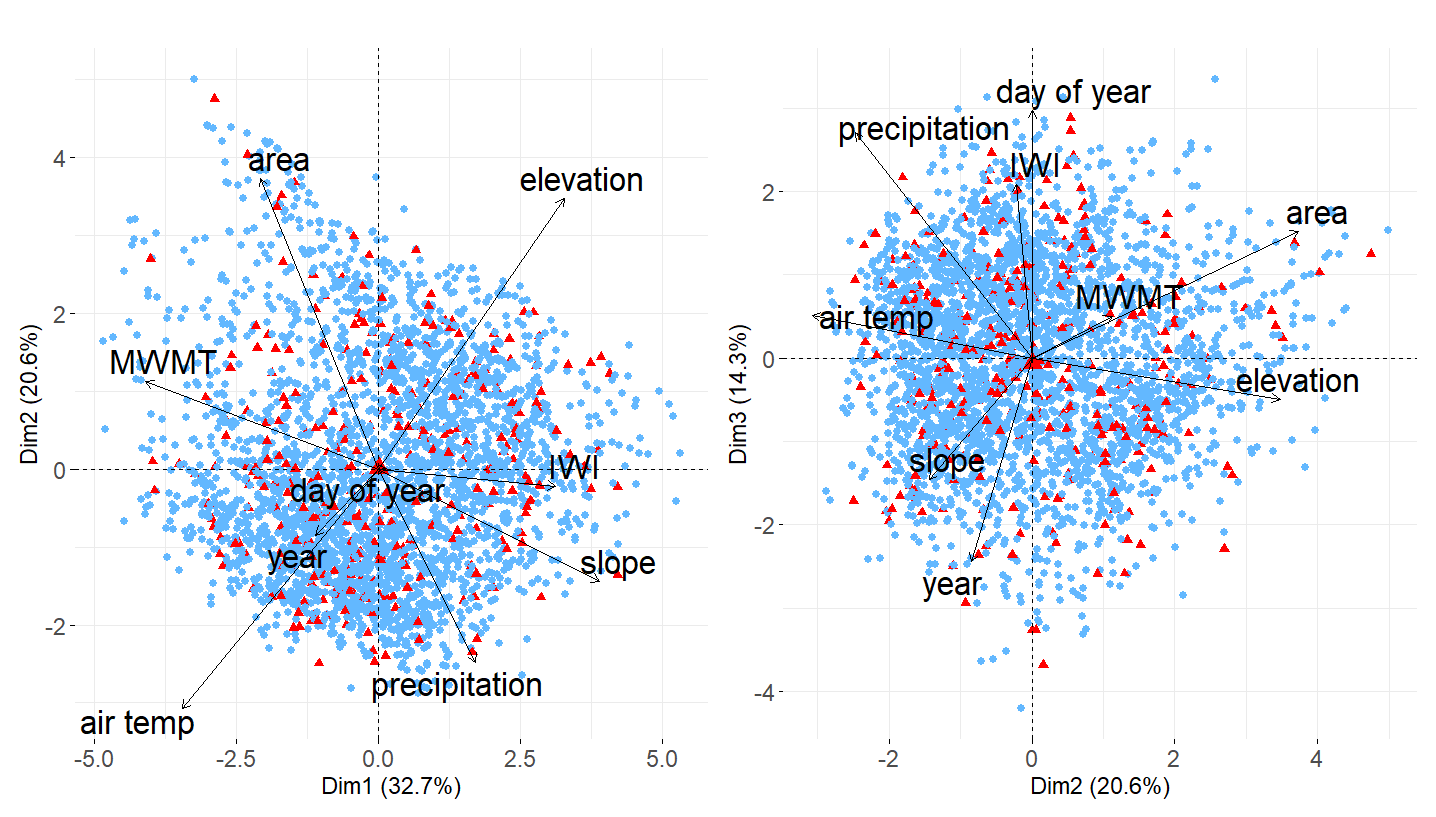


Figure S4-2. Principal components analysis (PCA) biplots of sites and environmental/disturbance variables. Panel A shows the first and second principal components, Panel B shows the second and third principal components. The percentage of total variance explained by each principal component is shown in parentheses for each axis. Blue points = calibration dataset (CAL), red triangles = validation dataset ().

## Modeling techniques and choices in MTTI development

**Weighted Averaging (WA): deshrinking and tolerance downweighting**

To calculate the MTTI, we used WA calibration and regression (ter Braak and Looman 1986). In the calibration step, optima were calculated as described in Supplemental 1:

**Weighted average optima** is commonly used for estimating the central tendency of a taxon along an environmental gradient (ter Braak and Looman 1986, Yuan 2006). Values were calculated with the “analogue” R package (Simpson 2007) using the formula:


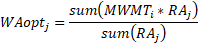


where *i* is the sample index, *j* is the taxa index, and *RA* is the relative abundance. The calculation involves multiplying MWMT by taxon relative abundance (the weighting factor) for each sample, summing the resulting products, then dividing that by the sum of all the relative abundances (weights).

The regression step is used to estimate environmental conditions at a site (MTTI), where WA optima for taxa (j) are weighted by relative abundances within a sample (i), then these weighted values were summed across all OTUs in a sample.


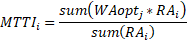


Because averages are taken twice, once to estimate thermal optima and again in averaging all the optima-weighted relative abundances, the resulting index has a reduced scale. To spread index values back out to their original range, WA models employ various deshrinking techniques. We explored three deshrinking options for our WA models: classical, inverse, and monotonic (Birks and Simpson 2013). While both inverse and monotonic deshrinking methods are better at reducing overall model prediction errors (RMSEP), they frequently result in biased predictions at the extremes of the environmental gradient. Classical deshrinking results in less biased predictions at environmental extremes (i.e., reduced maximum bias), typically at the expense of increased global errors (i.e., increased RMSEP). In addition, we examined models with and without tolerance downweighting (Birks et al. 1990). CAL model performance was assessed by the RMSEP, r2, and maximum bias in final MTTI predictions. We also used bootstrapped cross-validation (n = 1000) as another measure of CAL model performance. Finally, we calculated the same model performance measures for the VAL dataset. All MTTI analyses were performed using the ‘rioja’ package (Juggins 2020) in R Statistical software (R Core Team 2023).

# Comparisons between field and modeled temperature

The very strong relationship between field temperature and NorWeST modeled stream temperatures is well documented by Isaak et al. (2017). The global errors (RMSEP) in the NorWeST models for mean August temperatures was 1.1 ^o^C, with mean August temperatures showing a very high correlation to MWMT. But some may still be questioning what it means to use modeled data to model taxa and assemblage thermal niches.

First, as shown above, MWMT is HIGHLY related to field temperature. Thus, we should see similar optima and assemblage-level index values regardless of which data source is used. The real advantage of using modeled data is that it increases our sample sizes by an order of magnitude, because we no longer need paired continuous field temperature and macroinvertebrate samples. Some may say it is easy to collect continuous field temperature, but it should be noted that this requires at a minimum two visits to a site, while it only takes one visit to get a valid BMI sample. In addition, the BMI sample has the ability to detect other potential stressors beyond temperature.

Below is a comparison of field temperature, modeled stream temperatures (MWMT), and modeled assemblage-level thermal tolerances (MTTI) (Figure S2-3). Both the field data and MWMT represent the seven-day average maximum temperatures (the seven days with the highest average maximum temperatures), so they are directly equivalent. The data used in these comparisons comes from 248 samples throughout Oregon that were used to construct a similar macroinvertebrate temperature model for Oregon using field temperature as the basis for tolerances (Huff et al. 2008). Not surprisingly, the relationship between the MTTI showed almost the exact same relationship to the field data in this example (r^2^ = 0.70) as it did to modeled stream temperatures across all of Oregon and Washington (r^2^ = 0.68; main paper, Table 3). In addition, the model errors in this study (RMSEP = 2.6 – 2.7) and Huff et al. (RMSEP = 2.0 – 2.7) were nearly equivalent.

We also compared thermal optima for 100 taxa that were found in both this paper and Huff et al. (2008) (Figure S2-4). Both studies used weighted averaging, however, optima in Huff et al. were based on continuous temperature data collected from data loggers in the field. Modeled optima on average were slightly higher than field-based optima, with a slope near 1 and a high coefficient of determination (r2 = 0.82).

Taken together, this provides strong evidence that using modeled stream temperatures to derive thermal tolerances for individual taxa and for an assemblage-level index is equivalent to using field collected maximum summer temperatures.


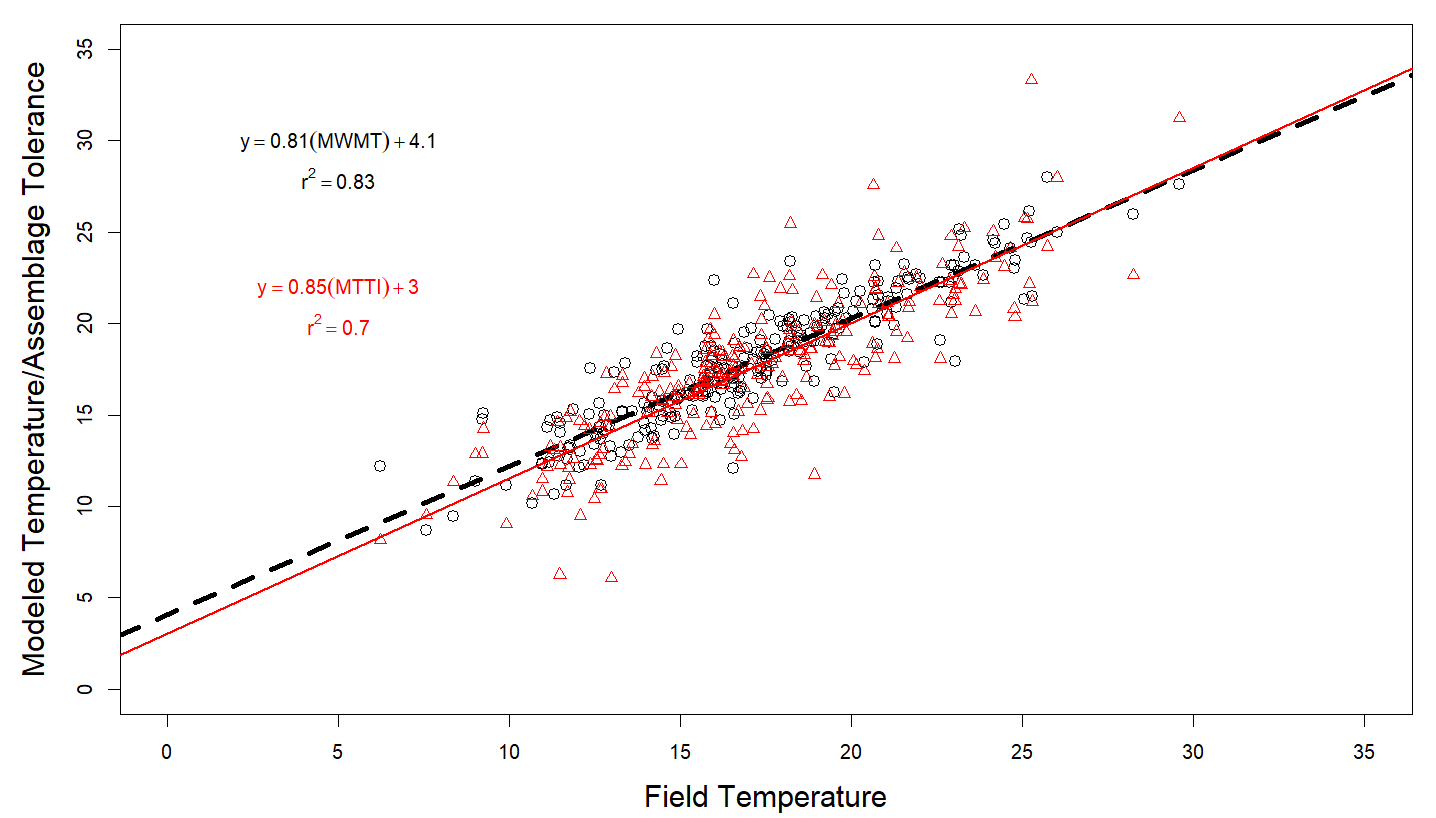


Figure S4-3. Relationships between field temperature (Huff et al. 2008), modeled stream temperatures (MWMT, black circles), and modeled benthic macroinvertebrate assemblage-level tolerances (MTTI, red triangles) at 248 sites in Oregon. All axes are on the same scale, degrees Celsius. Lines and text show results from linear regressions (black dashed = MWMT, red solid = MTTI).


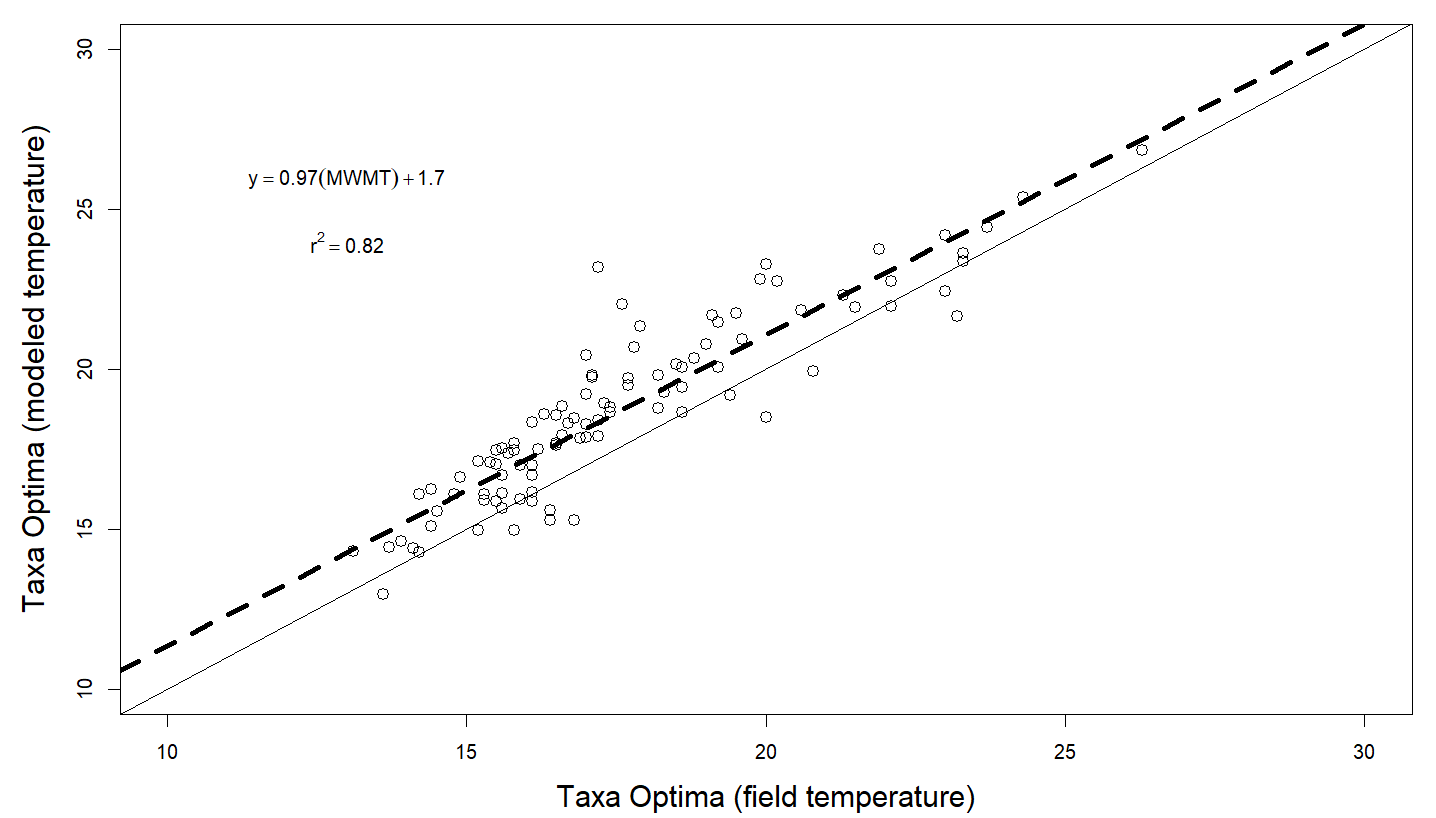


Figure S4-4. Comparisons of thermal optima based on field data and modeled stream temperature (MWMT), for 100 taxa also reported in Huff et al. (2008). All axes are on the same scale, degrees Celsius. The solid black line represents a perfect 1:1 relationship. The black dashed line and text show results of a linear regression between field and modeled temperature derived optima.

**Correlations with other stressors**

We used correlations analyses to see if the thermal signal of the MTTI could be influenced by covariance with other potential stressors (Table S2-2). Analyses were done in R (R Core Team 2023), using the ‘rcorr’ function from the ‘Hmisc’ package. Results were queried from the Oregon Department of Environmental Quality and Washington Department of Ecology databases and matched to MTTI values.

All parameters were significantly correlated with MTTI, except TSS. Only D.O. had a negative relationship to MTTI, which is to be expected as D.O. solubility decreases as temperature increases. Except for BSTI, all other significant correlations were weak, with the strongest correlations to Chloride (r = 0.31), Conductivity (r=0.33), and Percent Fines (r = 0.36). The highest correlations (r = 0.51) were between the two biological indexes, the MTTI and BSTI (Hubler et al. 2016).

Table S4-2. Pearson’s correlation coefficients (r) between the Macroinvertebrate Thermal Tolerance Index (MTTI) and select water quality parameters, a physical habitat parameter (fine sediment), and a biological index of fine sediment tolerances (BSTI, Hubler et al. 2016). ‘n’ = sample size. Bold values represent significant correlations. All data was sourced from Oregon Department of Environmental Quality and Washington Department of Ecology.

| **Parameter** | **units** | **n** | **r** |
| --- | --- | --- | --- |
| Dissolved Oxygen (D.O.) | mg/l | 2360 | **-0.26** |
| Total Suspended Solids (TSS) | mg/l | 2065 | 0.03 |
| Phosphorus | mg/l | 1034 | **0.18** |
| Nitrogen | mg/l | 1087 | **0.26** |
| Chloride | mg/l | 1885 | **0.31** |
| Conductivity | uS/cm | 2381 | **0.33** |
| Fine Sediments | % | 1027 | **0.36** |
| BSTI | % | 1027 | **0.51** |

## Fine sediments

Fine sediments in this study were defined as the smallest particles, with a diameter < 0.6 mm. Benthic sediments were assessed using a modified Wolmann pebble count and the same methods as used in the US EPA’s National Rivers and Streams Assessment (Kaufmann et al. 1999).

We also compared MTTI to a fine sediment stressor-specific model, the Biological Sediment Tolerance Index (BSTI, Hubler et al. 2016). Like the MTTI, the BSTI is a weighted average model where taxa optima are weighted by relative abundances, then summed to represent an assemblage-level tolerance for fine sediments. The BSTI was calibrated on the same percent fines metric as described above.

MTTI shows a weak, positive correlation (r = 0.36) with field measured fine sediments. A slightly higher and moderate, positive correlation (r = 0.51) was observed with the BSTI. This suggests the potential for one stressor-specific model to also be influenced by another stressor. In other words, a high MTTI could potentially be due to a sample containing high abundances of taxa that also have high tolerances to fine sediments. This is why it is important to validate each stressor index by examining the taxa lists and abundances and look for these co-tolerant taxa.

**Literature Cited**

Birks, H.J.B., Braak, C.T., Line, J.M., Juggins, S. and Stevenson, A.C., 1990. Diatoms and pH reconstruction. Philosophical transactions of the royal society of London. B, Biological Sciences, 327(1240), pp.263-278.

Birks, H.J.B. and Simpson, G.L., 2013. ‘Diatoms and pH reconstruction’ (1990) revisited. Journal of paleolimnology, 49, pp.363-371.

Hill, R.A., Weber, M.H., Leibowitz, S.G., Olsen, A.R. and Thornbrugh, D.J., 2016. The Stream‐Catchment (StreamCat) Dataset: A database of watershed metrics for the conterminous United States. JAWRA Journal of the American Water Resources Association, 52(1), pp.120-128.

Hubler, S., Huff, D.D., Edwards, P. and Pan, Y., 2016. The Biological Sediment Tolerance Index: Assessing fine sediments conditions in Oregon streams using macroinvertebrates. Ecological Indicators, 67, pp.132-145.

Huff, D.D., Hubler, S.L., Pan, Y. and Drake, D.L., 2008. Detecting shifts in macroinvertebrate assemblage requirements: implicating causes of impairment in streams. Oregon Department of Environmental Quality Watershed Assessment. Technical Report: DEQ06-LAB-0068-TR.

Isaak, D.J., Wenger, S.J., Peterson, E.E., Ver Hoef, J.M., Nagel, D.E., Luce, C.H., Hostetler, S.W., Dunham, J.B., Roper, B.B., Wollrab, S.P. and Chandler, G.L., 2017. The NorWeST summer stream temperature model and scenarios for the western US: A crowd‐sourced database and new geospatial tools foster a user community and predict broad climate warming of rivers and streams. Water Resources Research, 53(11), pp.9181-9205.

Juggins, S., Juggins, M.S., 2019. Package ‘rioja’. RCRAN .

Kaufmann, P.R., Levine, P., Peck, D.V., Robison, E.G. and Seeliger, C., 1999. Quantifying physical habitat in wadeable streams (p. 149). USEPA [National Health and Environmental Effects Research Laboratory, Western Ecology Division].

McKay, L., Bondelid, T., Dewald, T., Rea, A. and Moore, R., 2012. NHD Plus Version 2: User Guide. Application-ready geospatial framework of US surface-water data products associated with the USGS National Hydrography Dataset.

R Core Team, t., 2023. R: A language and environment for statistical computing.

Ter Braak, C.J. and Looman, C.W., 1986. Weighted averaging, logistic regression and the Gaussian response model. Vegetatio, 65, pp.3-11.
