# Supplementary material for: Improved thermal preferences and a stressor index derived from modeled stream temperatures and regional taxonomic standards for freshwater macroinvertebrates of the Pacific Northwest, USA
Source: Ecol Indic. Author manuscript; Available in PMC 2025 Apr 9. (PMC11980781; doi:10.1016/j.ecolind.2024.111869)

Chironomidae

Chiro −Ablabesmyia
nOcc=70; WAopt=21.5; PctRange=16.6−25.9
Unclear; Warm

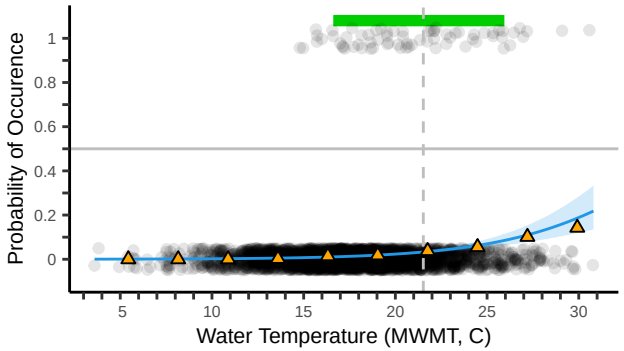

Chiro −Alotanypus
nOcc=42; WAopt=20.8; PctRange=16.6−24.2
Unclear; Warm

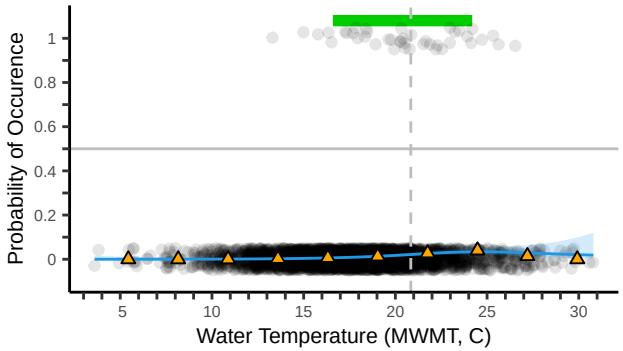

Chiro −Apedilum
nOcc=30; WAopt=22.9; PctRange=17.7−26.7
Unclear; Warm

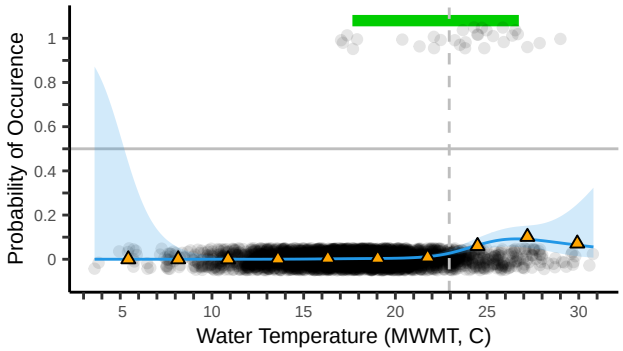

Chiro −Boreochlus
nOcc=72; WAopt=16.9; PctRange=12.2−20.0
Unclear; Cold

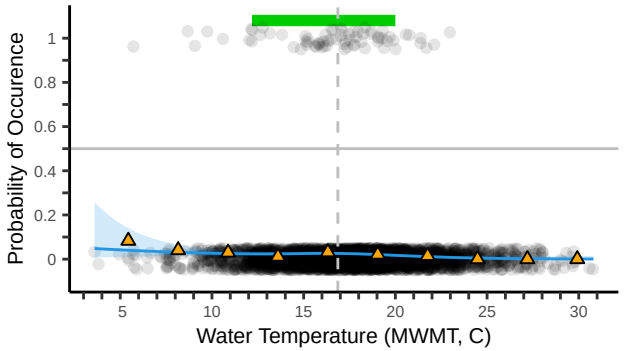

Chiro −Boreoheptagyia
nOcc=43; WAopt=14.2; PctRange=12.7−19.4
Unclear; Cold

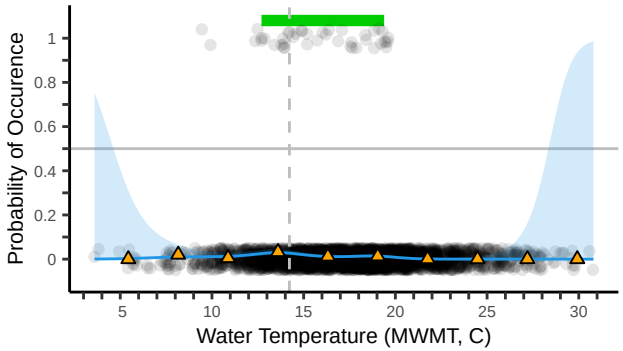

Chiro −Brillia
nOcc=1,442; WAopt=17.3; PctRange=13.3−20.9
Unimodal/Decreaser; Cool

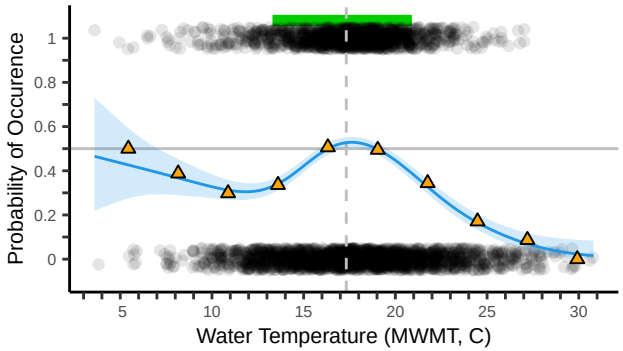

Chiro −Brundiniella
nOcc=250; WAopt=18.9; PctRange=14.5−21.7
Unclear; Cool

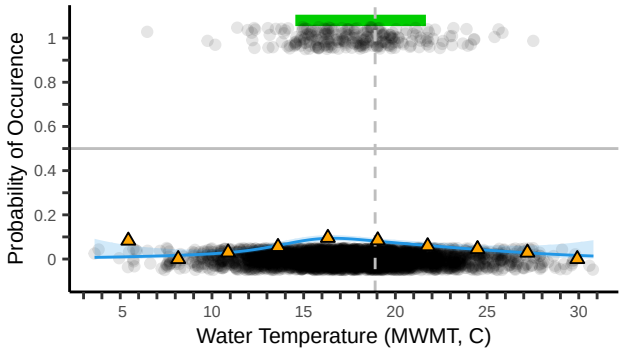

Chiro −Cardiocladius
nOcc=100; WAopt=22.2; PctRange=15.7−26.2
Increaser; Warm

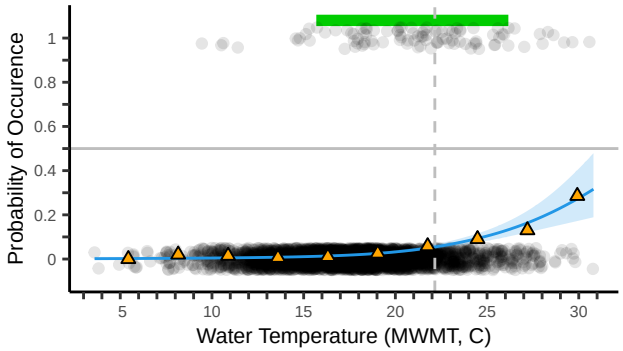

Chiro −Chaetocladius
nOcc=216; WAopt=15.4; PctRange=10.6−20.2
Decreaser; Cool

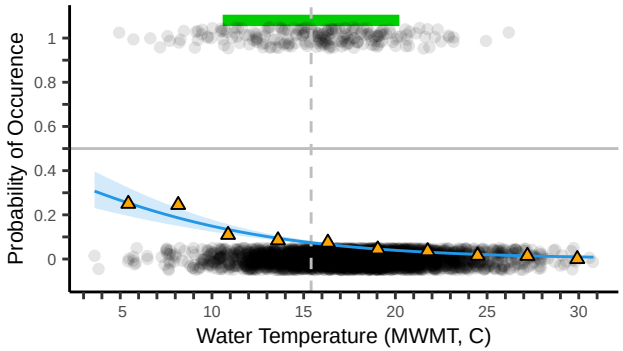

Chironomidae

Chiro −Chironominae
nOcc=3,227; WAopt=18.7; PctRange=13.2−22.6
Increaser*; Eurythermal

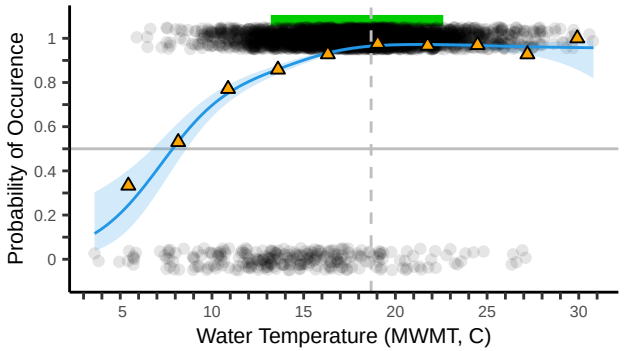

Chiro −Chironomini
nOcc=1,693; WAopt=20.3; PctRange=15.7−23.5
Increaser; Warm

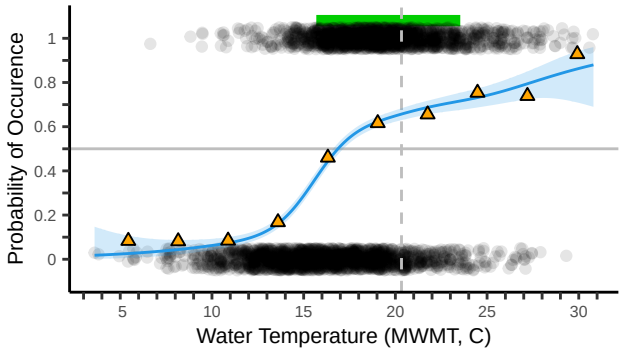

Chiro −Chironomus
nOcc=96; WAopt=20.4; PctRange=16.2−24.2
Unclear; Warm

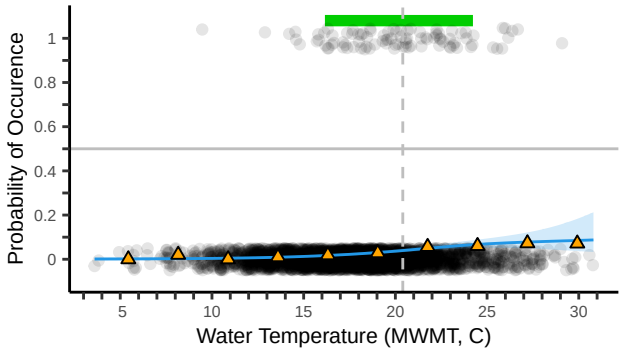

Chiro −Cladotanytarsus
nOcc=266; WAopt=19.6; PctRange=16.3−23.9
Unclear; Warm

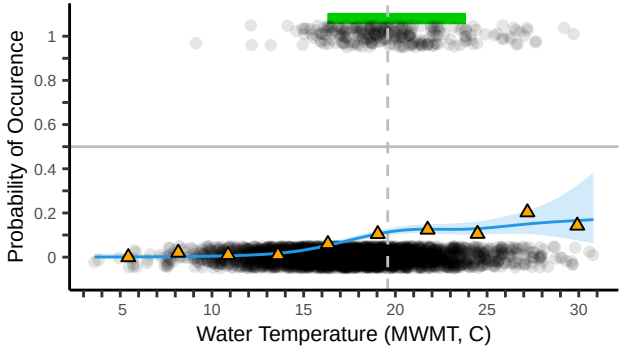

Chiro −Coelotanypodini
nOcc=43; WAopt=20.8; PctRange=16.7−24.2
Unclear; Warm

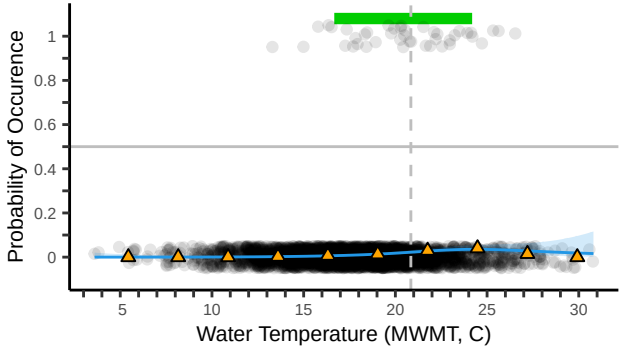

Chiro −Conchapelopia
nOcc=53; WAopt=20.0; PctRange=16.4−24.8
Unclear; Warm

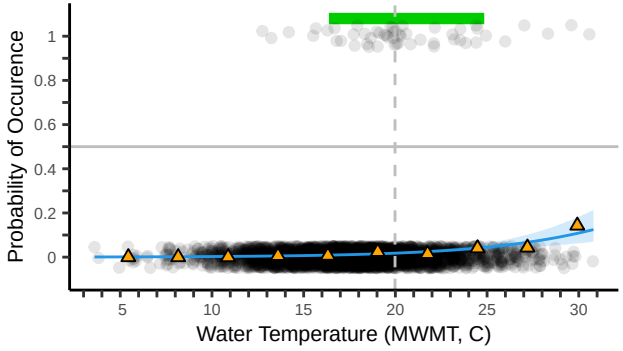

Chiro −Constempellina
nOcc=42; WAopt=17.6; PctRange=12.8−20.4
Unclear; Cool

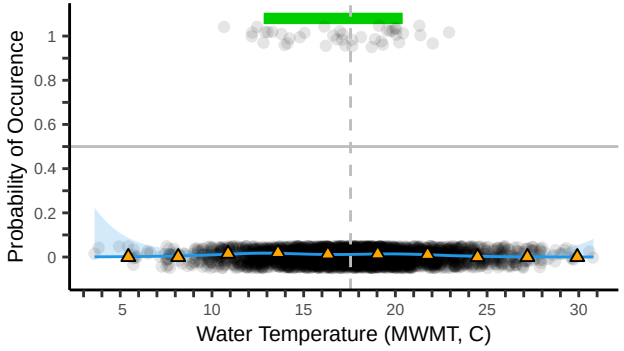

Chiro −Corynoneura
nOcc=714; WAopt=18.4; PctRange=13.2−22.9
Flat*; Eurythermal

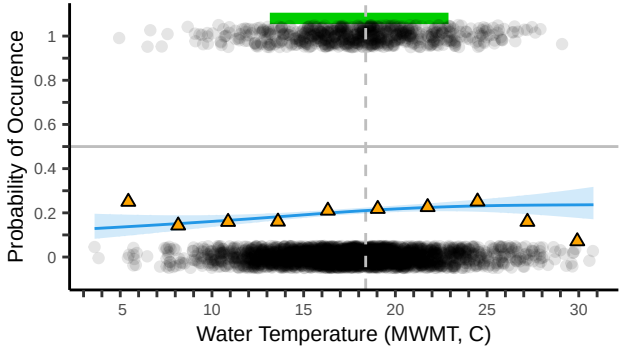

Chiro −Cricotopus
nOcc=643; WAopt=20.3; PctRange=14.5−25.1
Increaser; Warm

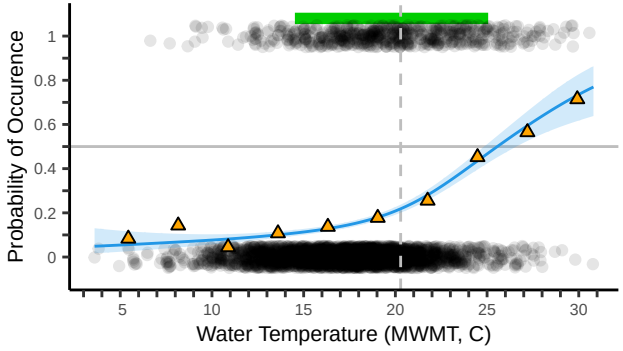

Chironomidae

Chiro −Cricotopus (Isocladius)
nOcc=35; WAopt=21.2; PctRange=15.8−27.0
Unclear; Warm

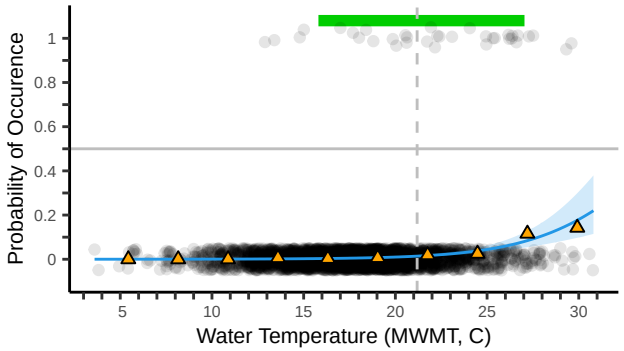

Chiro −Cricotopus (Nostococladius)
nOcc=141; WAopt=19.3; PctRange=12.5−23.4
Unclear; Eurythermal

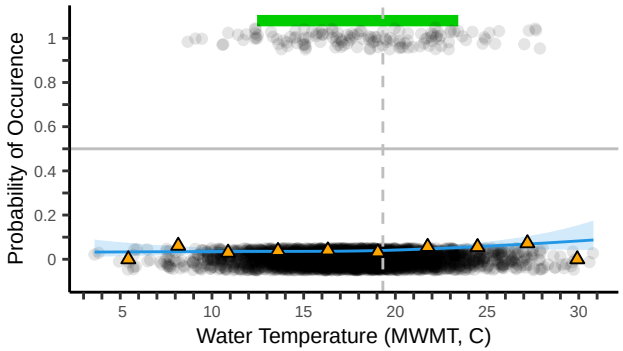

Chiro −Cricotopus bicinctus group
nOcc=101; WAopt=21.6; PctRange=18.2−25.9
Unclear; Warm

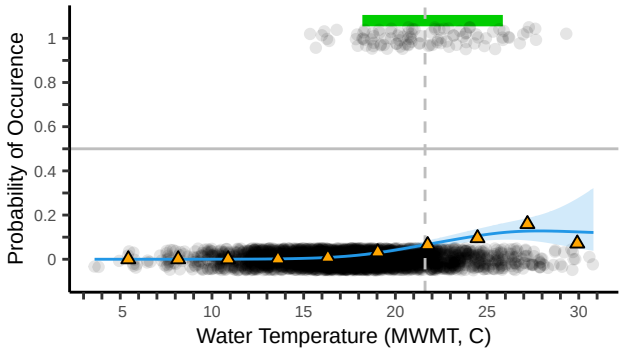

Chiro −Cricotopus trifascia group
nOcc=60; WAopt=23.4; PctRange=19.0−26.5
Unclear; Warm

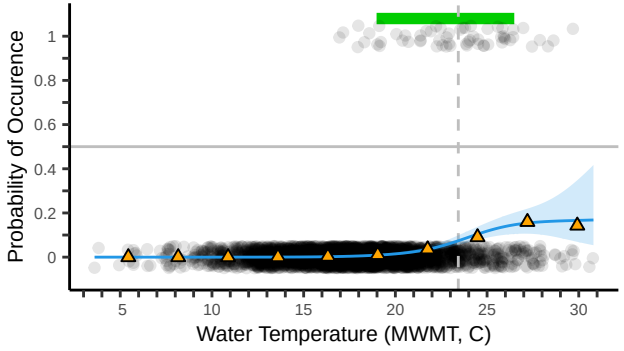

Chiro −Cryptochironomus
nOcc=105; WAopt=22.3; PctRange=18.9−26.0
Unclear; Warm

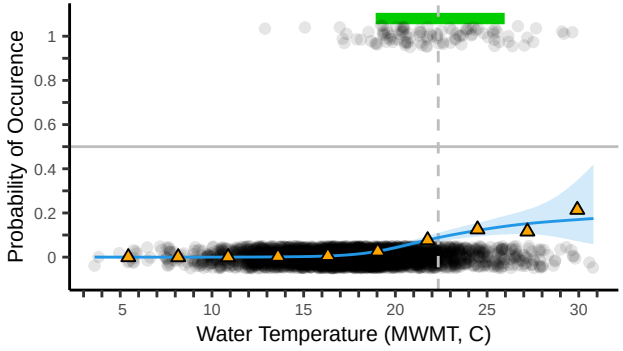

Chiro −Diamesa
nOcc=82; WAopt=9.4; PctRange=9.1−20.6
Unclear; Cold*

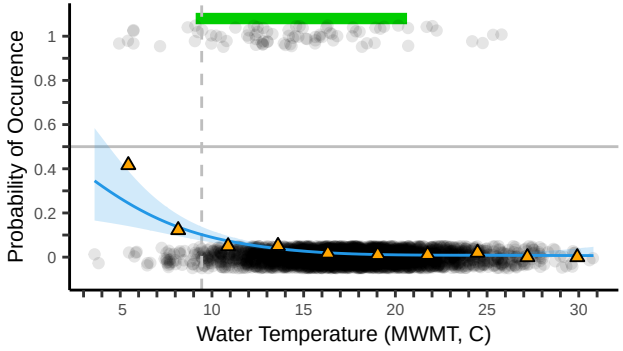

Chiro −Diamesinae
nOcc=953; WAopt=15.3; PctRange=12.5−22.4
Decreaser; Eurythermal

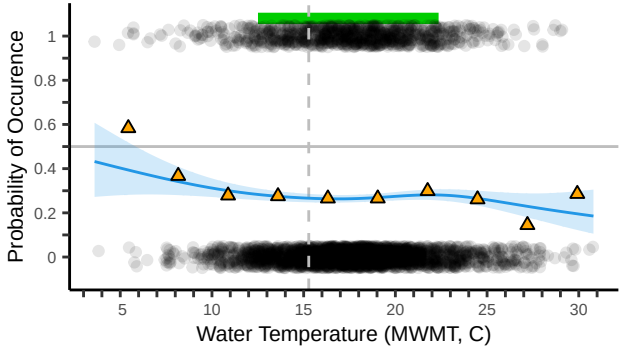

Chiro −Diamesini
nOcc=789; WAopt=14.9; PctRange=12.4−22.5
Decreaser; Eurythermal

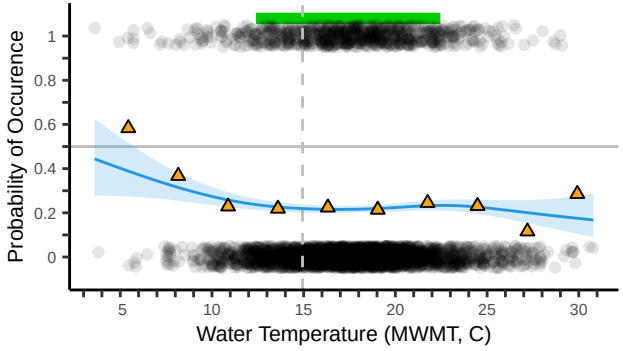

Chiro −Dicrotendipes
nOcc=74; WAopt=25.0; PctRange=17.8−27.1
Increaser; Warm

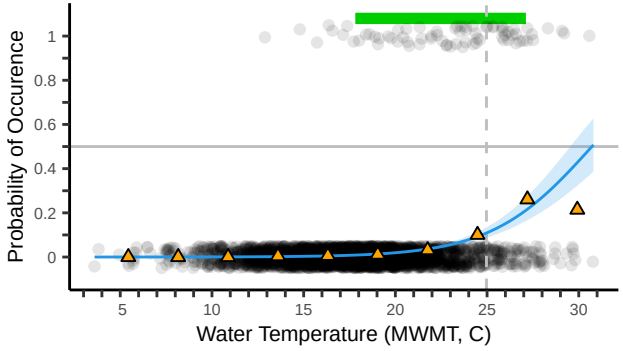

Chironomidae

Chiro −Diplocladius
nOcc=61; WAopt=17.8; PctRange=16.3−22.6
Unclear; Cool−Warm

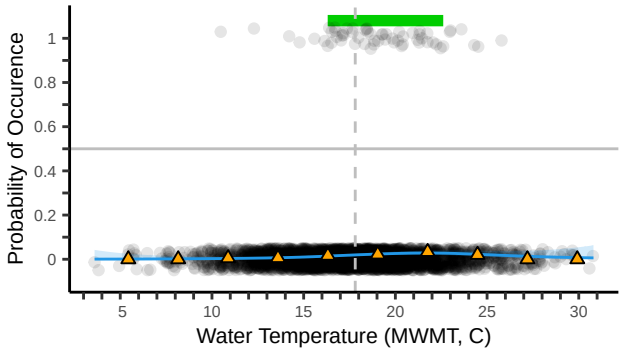

Chiro −Eukiefferiella
nOcc=1,446; WAopt=17.6; PctRange=13.2−22.8
Unclear*; Eurythermal

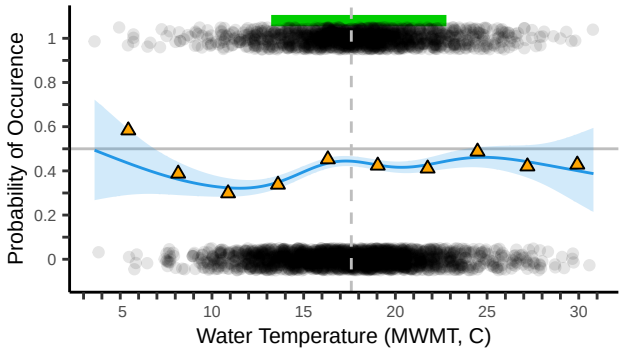

Chiro −Eukiefferiella brehmi group
nOcc=148; WAopt=19.3; PctRange=13.7−24.6
Unclear; Eurythermal

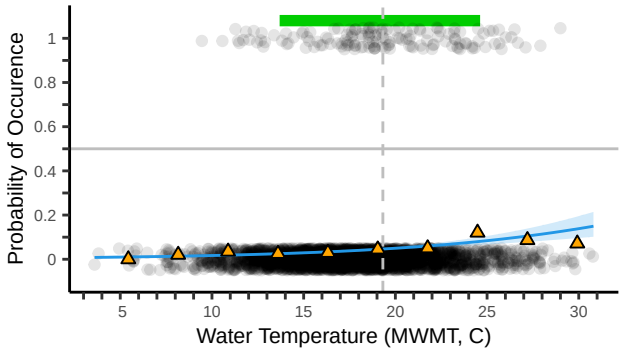

Chiro −Eukiefferiella claripennis group
nOcc=234; WAopt=18.4; PctRange=15.2−23.3
Unclear; Cool−Warm

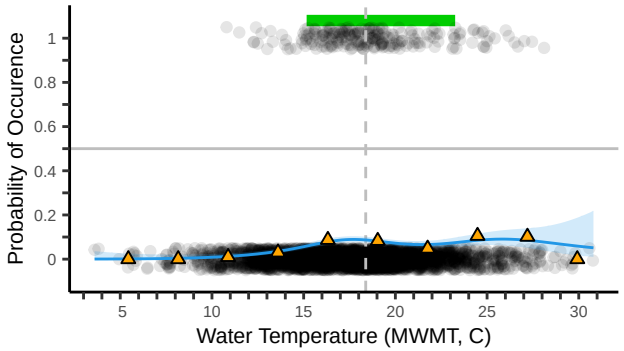

Chiro −Eukiefferiella devonica group
nOcc=152; WAopt=18.5; PctRange=12.3−24.5
Unclear; Eurythermal

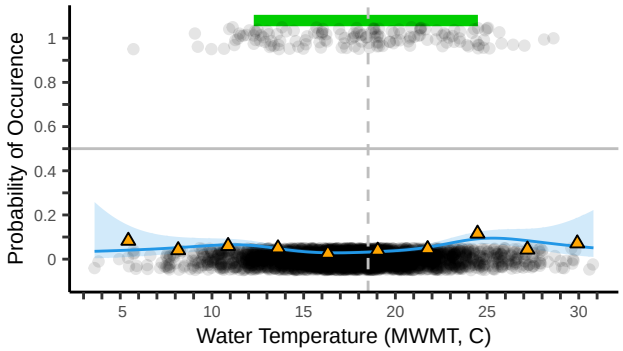

Chiro −Eukiefferiella gracei group
nOcc=43; WAopt=15.2; PctRange=11.4−24.1
Unclear; Eurythermal

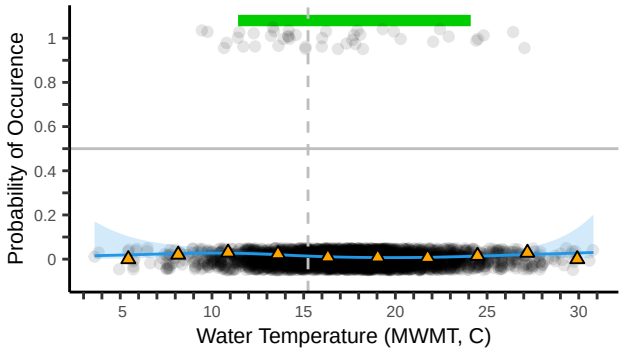

Chiro −Eukiefferiella pseudomontana group
nOcc=132; WAopt=18.6; PctRange=16.1−21.8
Unclear; Cool−Warm

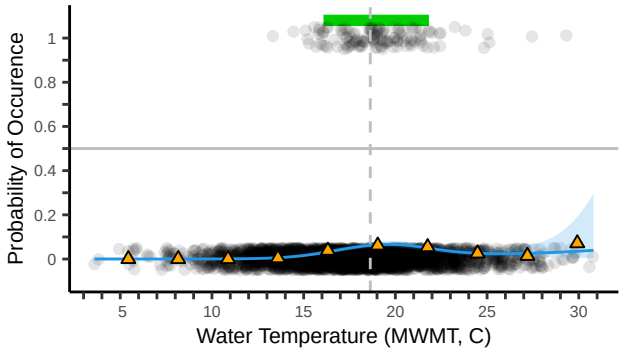

Chiro −Eukiefferiella tirolensis group
nOcc=66; WAopt=17.4; PctRange=14.7−18.9
Unclear; Cool

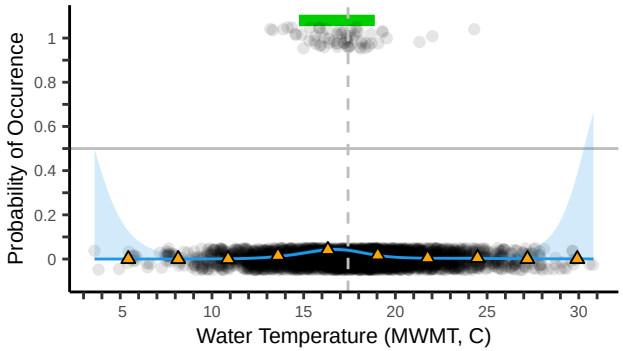

Chiro −Heleniella
nOcc=271; WAopt=16.6; PctRange=13.4−20.7
Unclear; Cool

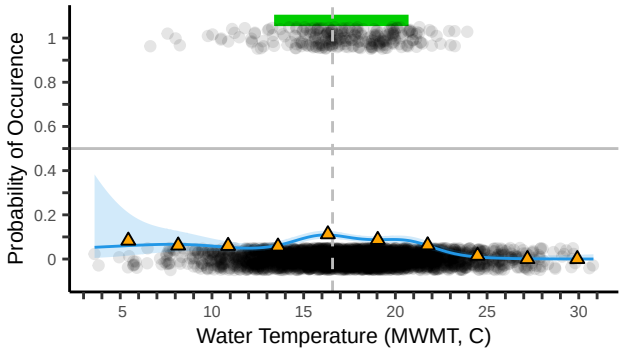

Chironomidae

Chiro −Heterotrissocladius
nOcc=303; WAopt=18.0; PctRange=13.8−21.8
Unclear; Cool

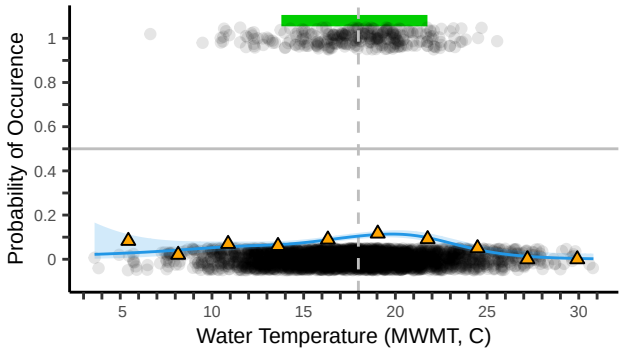

Chiro −Heterotrissocladius marcidus group
nOcc=98; WAopt=18.2; PctRange=15.2−21.8
Unclear; Cool

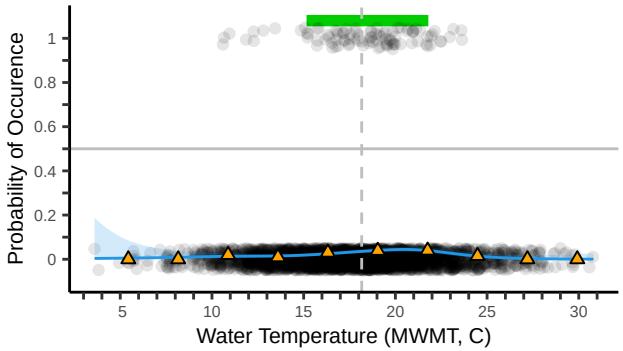

Chiro −Hydrobaenus
nOcc=68; WAopt=13.4; PctRange=10.7−20.2
Unclear; Cool

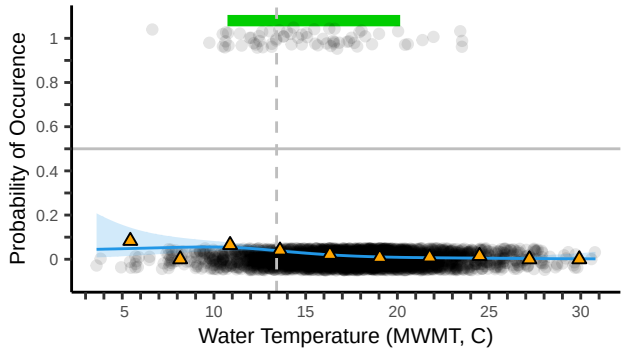

Chiro −Krenosmittia
nOcc=177; WAopt=14.8; PctRange=11.3−19.4
Unclear; Cold

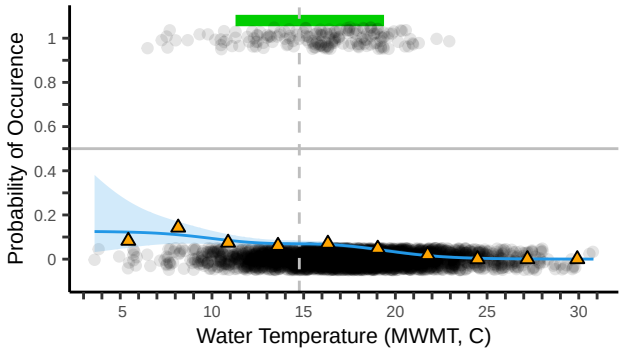

Chiro −Larsia
nOcc=169; WAopt=17.5; PctRange=13.1−22.3
Unclear; Eurythermal

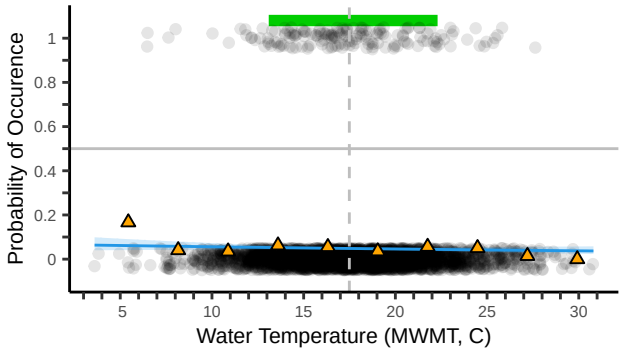

Chiro −Limnophyes
nOcc=227; WAopt=19.0; PctRange=12.7−23.2
Unclear; Eurythermal

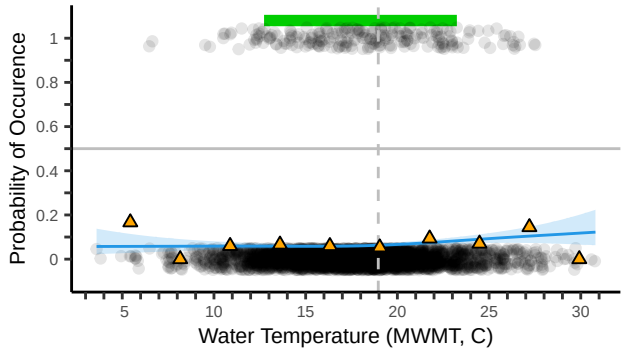

Chiro −Lopescladius
nOcc=63; WAopt=23.4; PctRange=18.7−26.9
Unclear; Warm

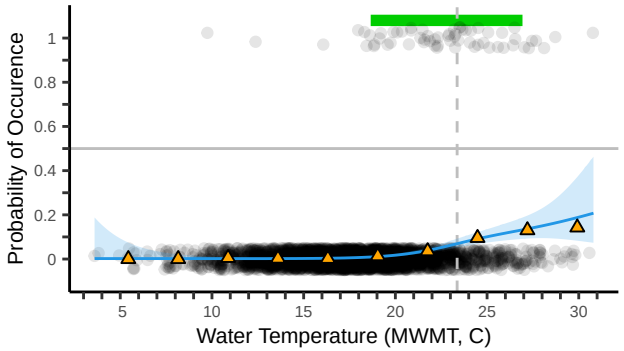

Chiro −Macropelopia
nOcc=73; WAopt=17.5; PctRange=12.0−20.8
Unclear; Cool

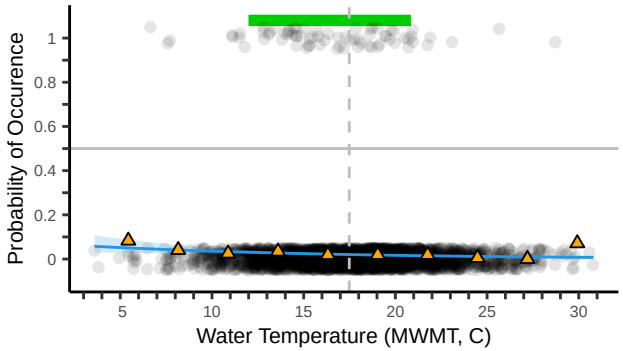

Chiro −Macropelopiini
nOcc=388; WAopt=18.8; PctRange=13.1−22.3
Unclear; Eurythermal

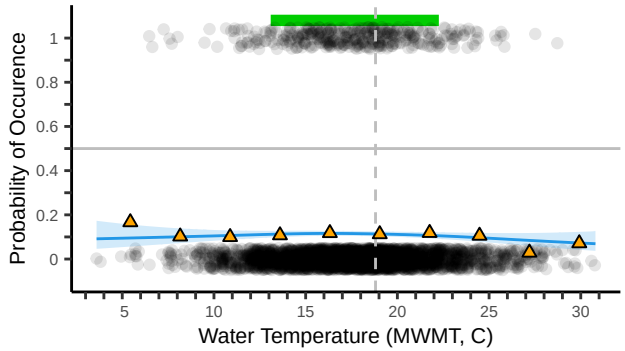

Chironomidae

Chiro −Metriocnemus
nOcc=76; WAopt=19.1; PctRange=11.7−22.4
Unclear; Eurythermal

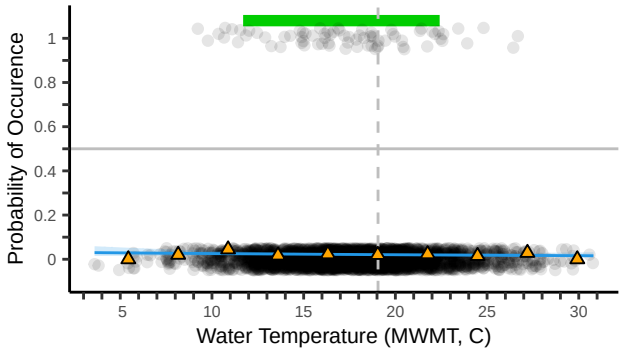

Chiro −Micropsectra
nOcc=1,580; WAopt=17.7; PctRange=13.4−22.2
Unimodal; Eurythermal

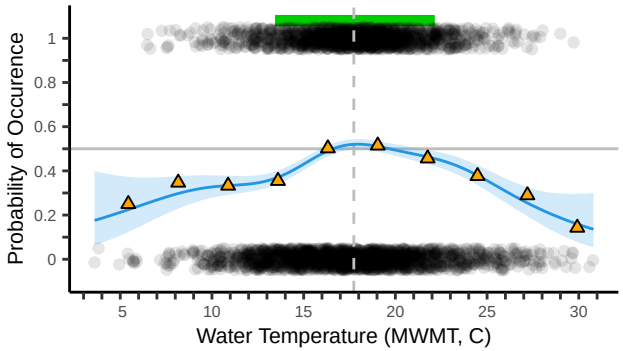

Chiro −Microtendipes
nOcc=357; WAopt=20.3; PctRange=16.2−23.6
Increaser; Warm

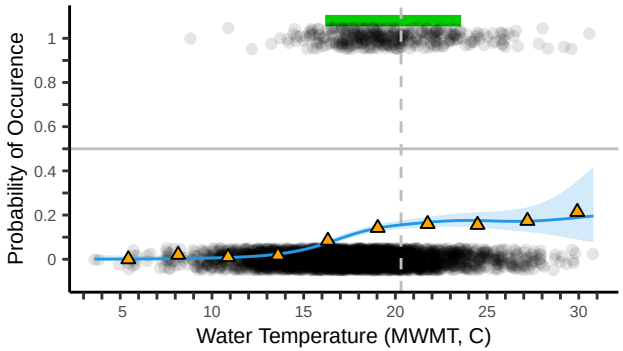

Chiro −Microtendipes pedellus group
nOcc=63; WAopt=20.2; PctRange=17.1−23.5
Unclear; Cool−Warm

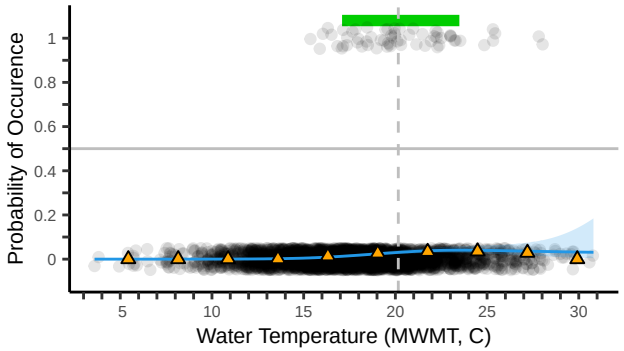

Chiro −Microtendipes rydalensis group
nOcc=36; WAopt=20.2; PctRange=15.6−22.2
Unclear; Cool−Warm

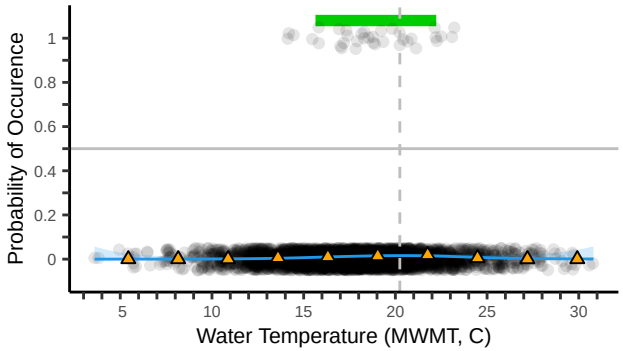

Chiro −Monodiamesa
nOcc=70; WAopt=16.7; PctRange=13.5−20.4
Unclear; Cool

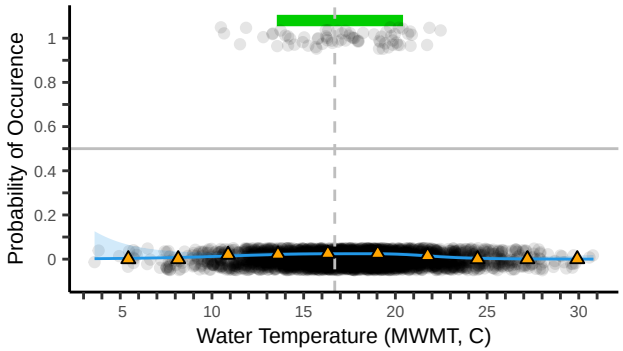

Chiro −Nanocladius
nOcc=107; WAopt=20.0; PctRange=15.5−25.9
Unclear; Warm

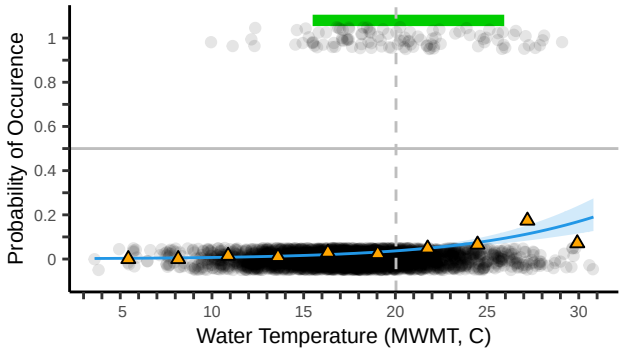

Chiro −Natarsia
nOcc=69; WAopt=19.3; PctRange=15.8−21.8
Unclear; Cool

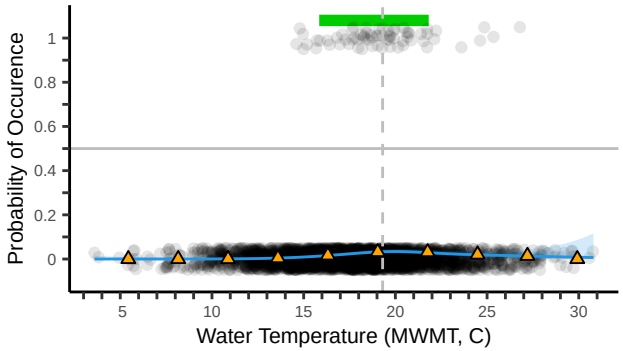

Chiro −Nilotanypus
nOcc=106; WAopt=21.0; PctRange=17.2−24.9
Unclear; Warm

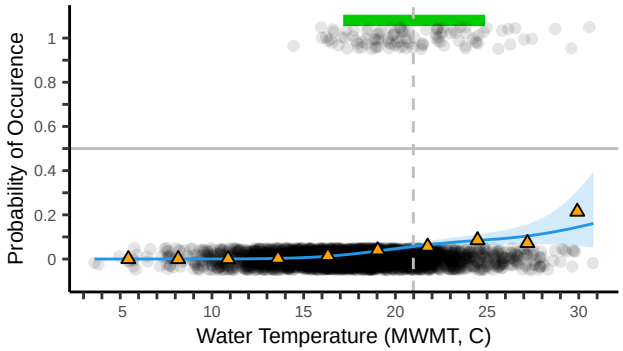

Chironomidae

Chiro −Odontomesa
nOcc=85; WAopt=20.2; PctRange=15.7−22.4
Unclear; Cool−Warm

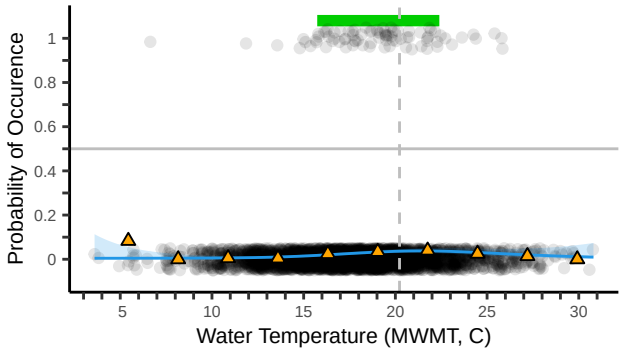

Chiro −Oropuella
nOcc=56; WAopt=13.5; PctRange=11.6−17.5
Unclear; Cold Stenotherm

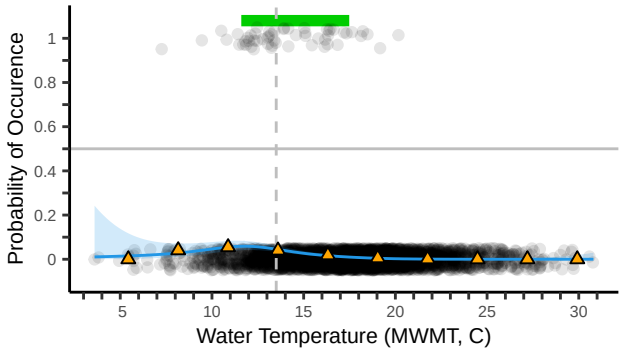

Chiro −Orthocladiinae
nOcc=3,332; WAopt=17.7; PctRange=13.0−22.5
Unclear*; Eurythermal

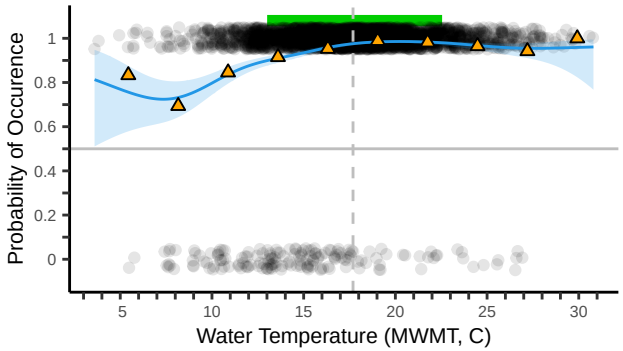

Chiro −Orthocladius
nOcc=776; WAopt=17.1; PctRange=12.6−22.6
Flat*; Eurythermal

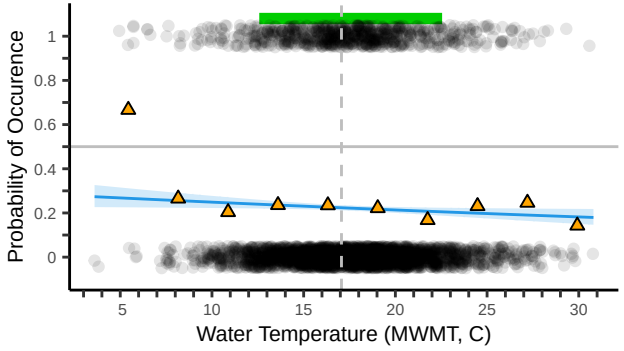

Chiro −Orthocladius (Symposiocladius)
nOcc=191; WAopt=16.8; PctRange=13.5−19.4
Unclear; Cold

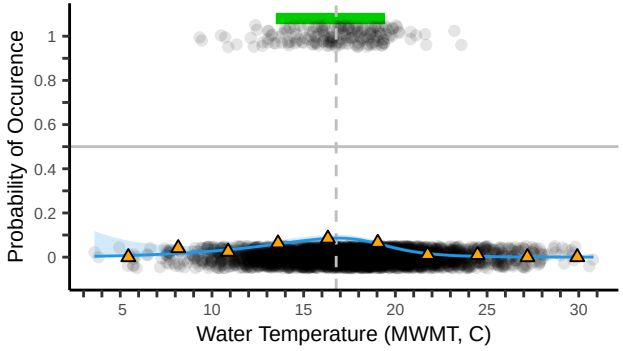

Chiro −Orthocladius complex
nOcc=439; WAopt=20.8; PctRange=15.6−25.6
Increaser; Warm

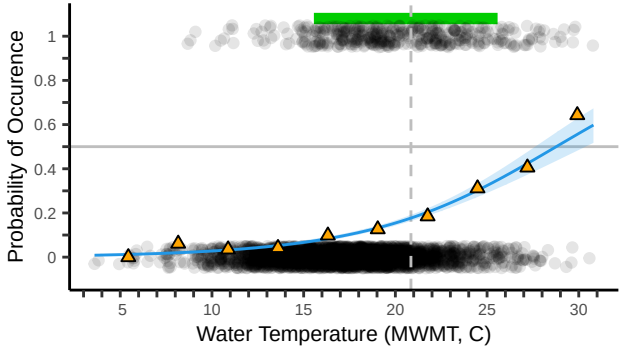

Chiro −Pagastia
nOcc=591; WAopt=16.4; PctRange=12.5−22.1
Decreaser; Eurythermal

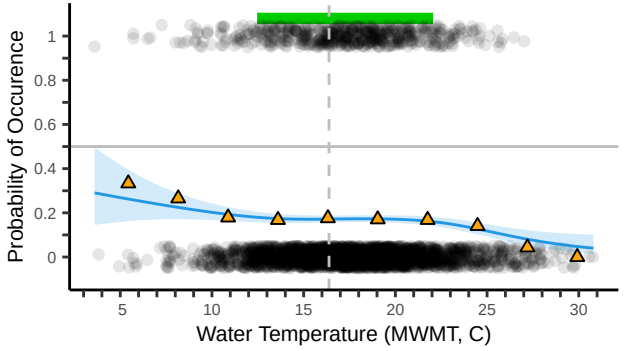

Chiro −Parachaetocladius
nOcc=31; WAopt=15.0; PctRange=10.7−21.2
Unclear; Cool

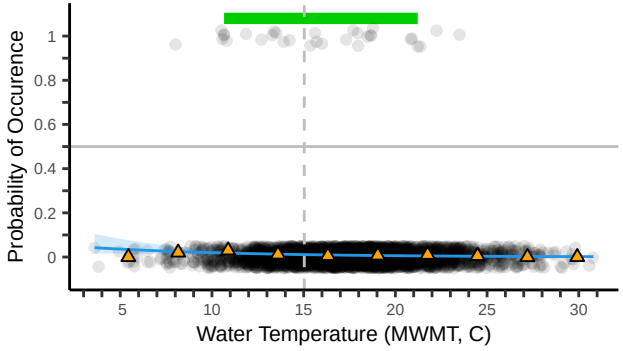

Chiro −Paracladopelma
nOcc=73; WAopt=19.0; PctRange=15.1−22.1
Unclear; Cool−Warm

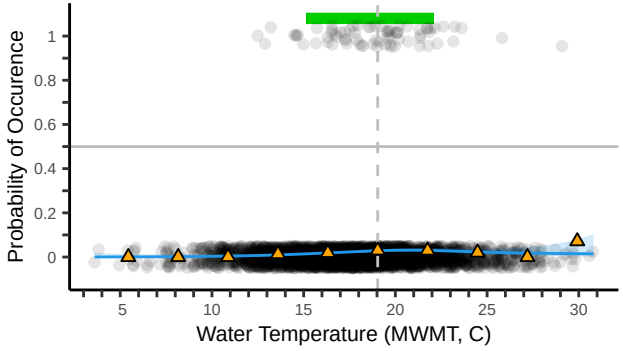

Chironomidae

Chiro −Parakiefferiella
nOcc=200; WAopt=19.8; PctRange=14.2−22.7
Unclear; Eurythermal

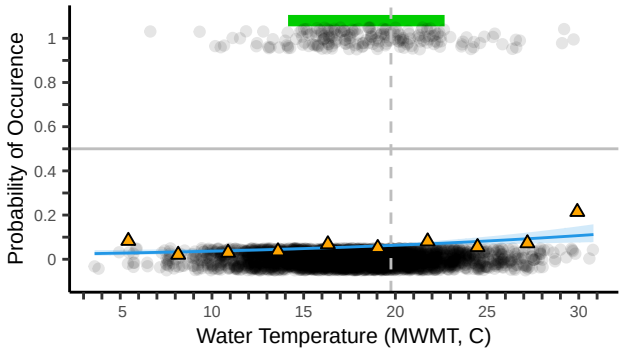

Chiro −Parametriocnemus
nOcc=1,301; WAopt=18.0; PctRange=14.0−22.3
Unimodal; Eurythermal

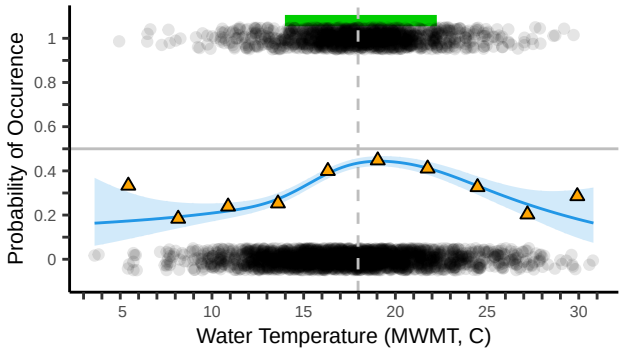

Chiro −Paraphaenocladius
nOcc=163; WAopt=15.5; PctRange=11.3−20.6
Unclear; Cool

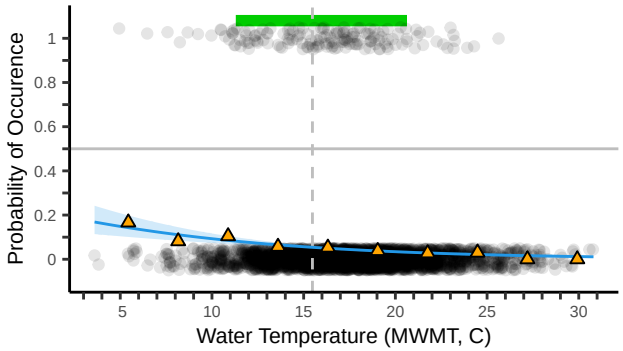

Chiro −Paratanytarsus
nOcc=235; WAopt=20.6; PctRange=16.8−24.8
Increaser*; Warm

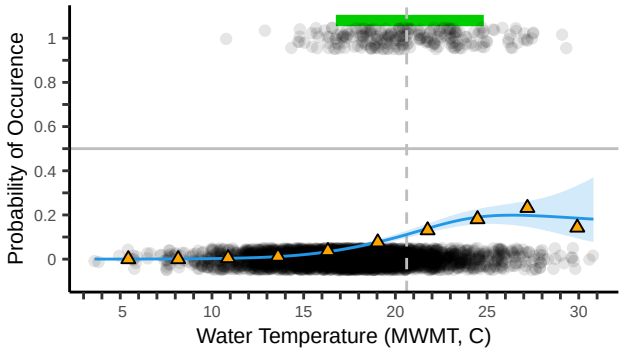

Chiro −Paratendipes
nOcc=97; WAopt=21.0; PctRange=14.8−24.2
Unclear; Warm

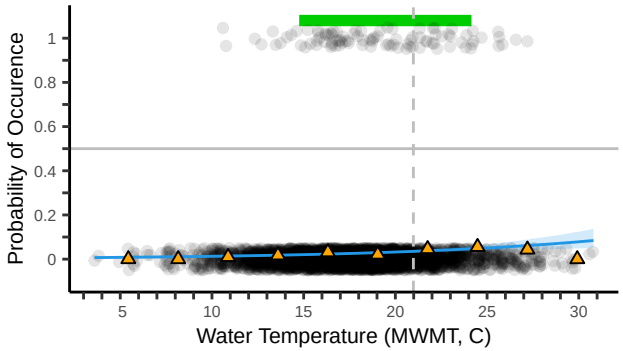

Chiro −Parorthocladius
nOcc=123; WAopt=14.4; PctRange=11.8−18.0
Unclear; Cold

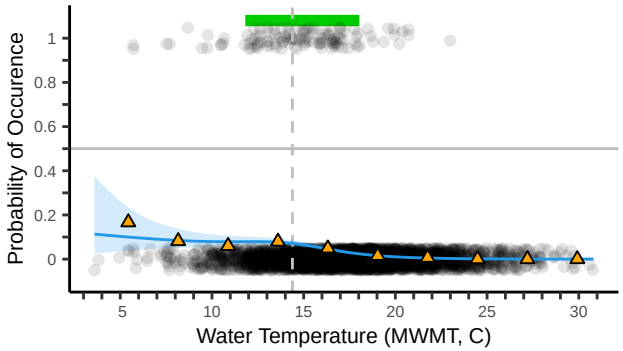

Chiro −Pentaneura
nOcc=136; WAopt=22.8; PctRange=17.3−27.0
Increaser; Warm

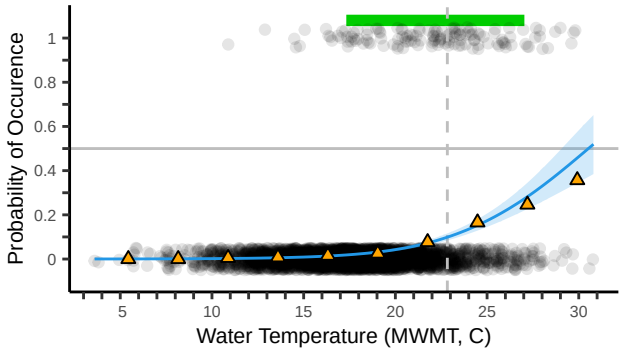

Chiro −Pentaneurini
nOcc=1,433; WAopt=19.3; PctRange=14.4−23.3
Increaser; Eurythermal

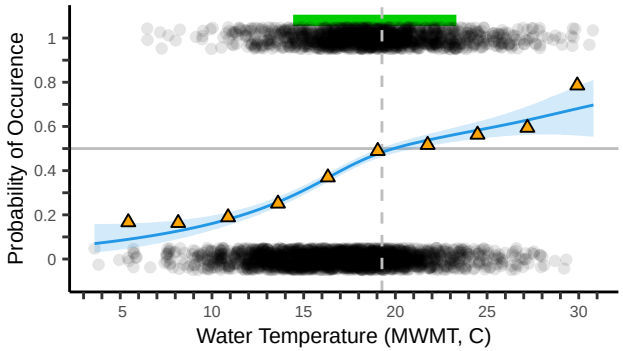

Chiro −Phaenopsectra
nOcc=326; WAopt=21.2; PctRange=17.2−25.5
Increaser; Warm

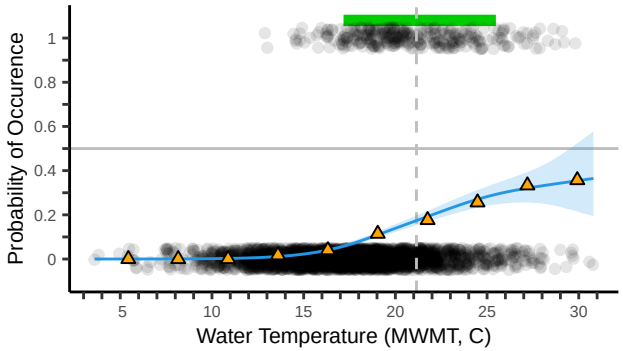

Chironomidae

Chiro − Podonominae
nOcc=85; WAopt=16.8; PctRange=12.2−20.3
Unclear; Cool

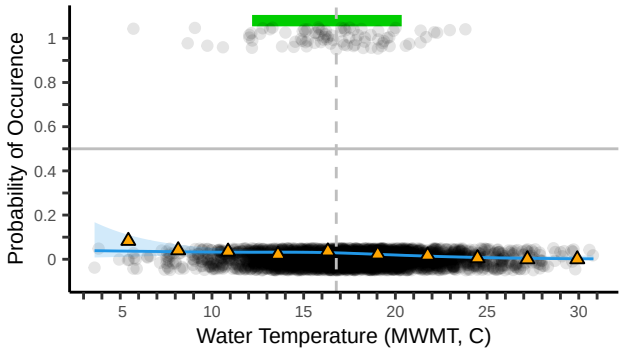

Chiro − Polypedilum
nOcc=1,289; WAopt=19.6; PctRange=15.6−23.5
Increaser; Warm

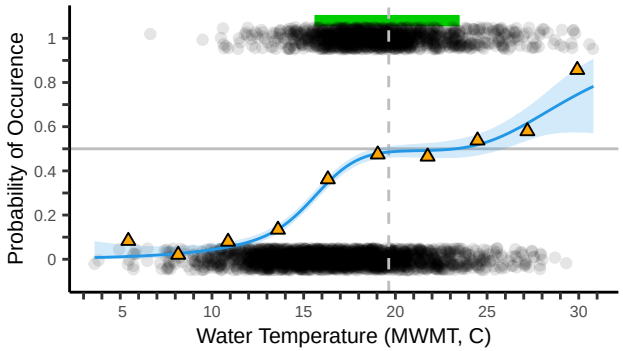

Chiro − Potthastia
nOcc=197; WAopt=20.2; PctRange=15.1−24.1
Increaser; Warm

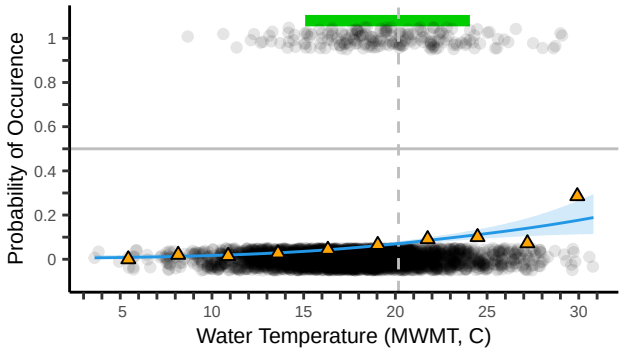

Chiro − Potthastia gaedii group
nOcc=145; WAopt=20.1; PctRange=14.5−23.8
Unclear; Warm*

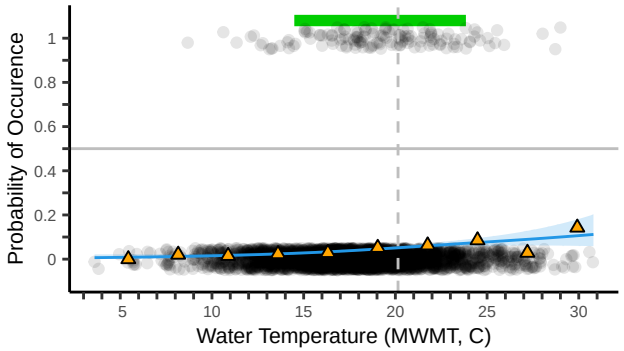

Chiro − Procladius
nOcc=98; WAopt=20.1; PctRange=17.1−25.1
Unclear; Warm

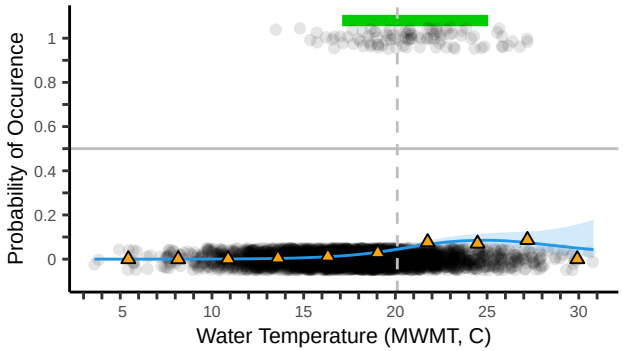

Chiro − Prodiamesa
nOcc=81; WAopt=19.6; PctRange=15.8−22.3
Unclear; Cool−Warm

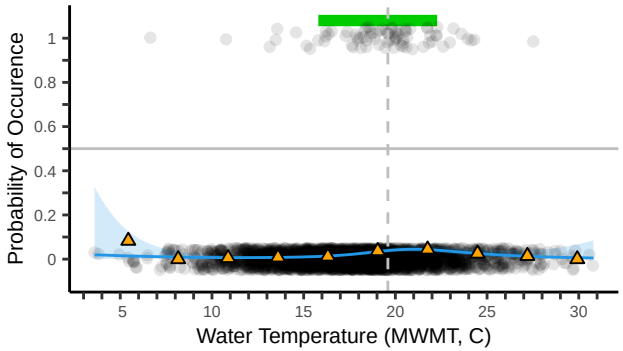

Chiro − Prodiamesinae
nOcc=206; WAopt=19.2; PctRange=14.5−22.3
Unclear; Cool−Warm

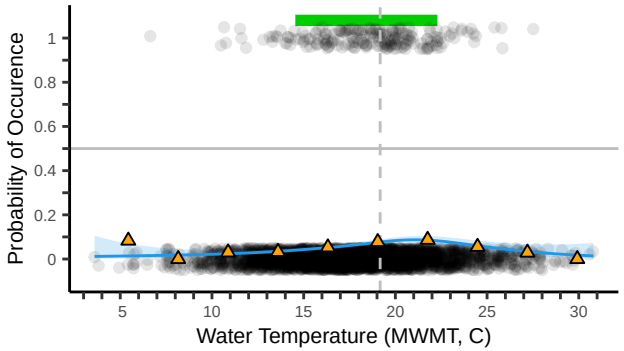

Chiro − Psectrocladius
nOcc=81; WAopt=18.9; PctRange=16.2−23.2
Unclear; Cool−Warm

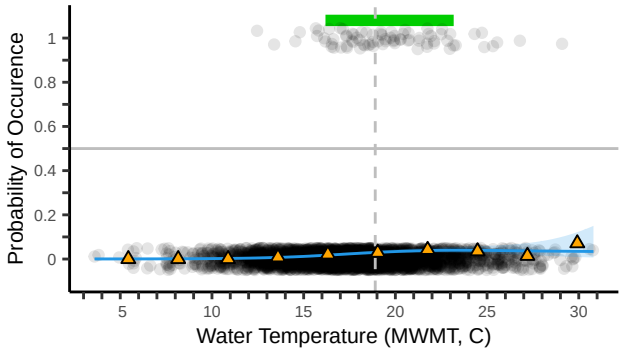

Chiro − Pseudodiamesa
nOcc=103; WAopt=13.1; PctRange=9.5−16.8
Unclear; Cold Stenotherm

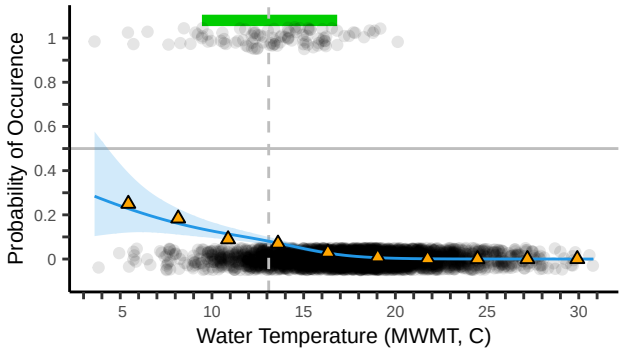

Chironomidae

Chiro −Pseudosmittia
nOcc=34; WAopt=18.7; PctRange=16.0−22.5
Unclear; Cool−Warm

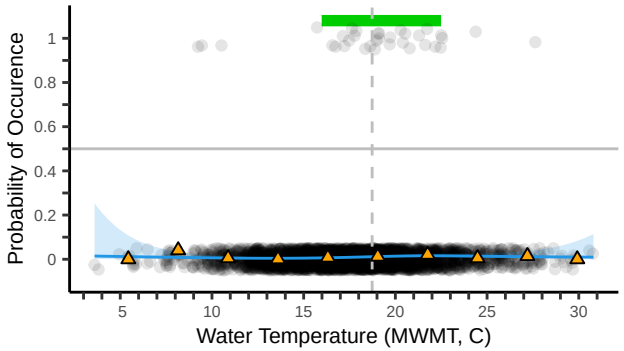

Chiro −Psilometriocnemus
nOcc=137; WAopt=17.0; PctRange=13.0−20.0
Unclear; Cold

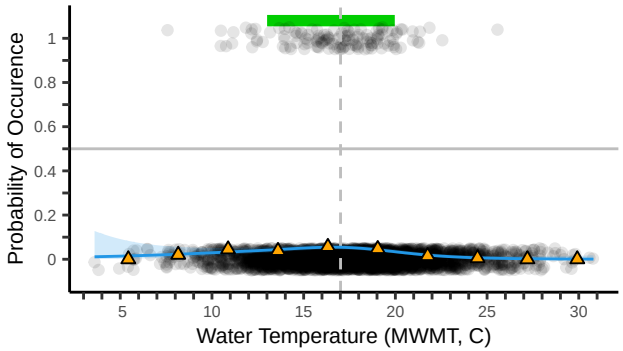

Chiro −Radotanypus
nOcc=57; WAopt=20.7; PctRange=15.3−23.7
Unclear; Warm

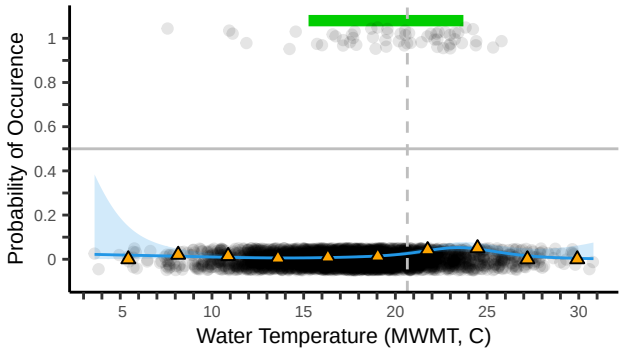

Chiro −Rheocricotopus
nOcc=756; WAopt=17.7; PctRange=12.6−23.1
Flat*; Eurythermal

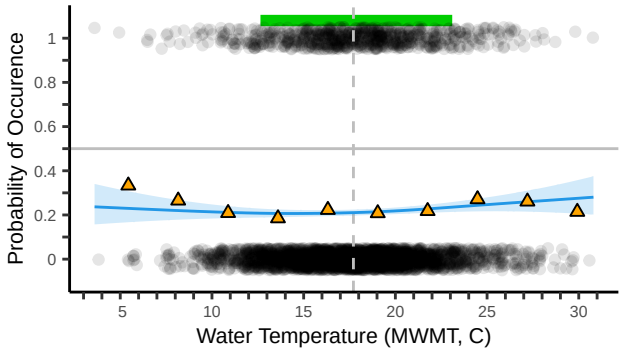

Chiro −Rheotanytarsus
nOcc=1,005; WAopt=19.9; PctRange=15.5−23.8
Increaser; Warm

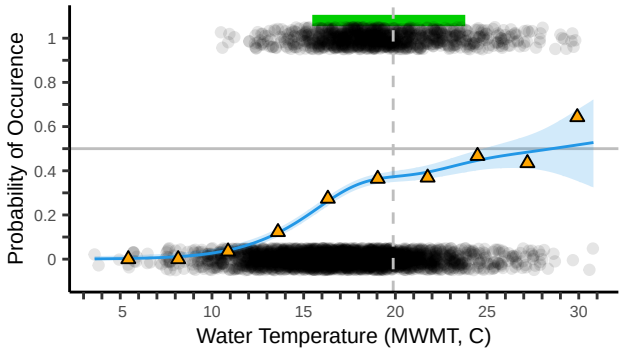

Chiro −Stempellina
nOcc=112; WAopt=17.6; PctRange=13.7−23.0
Unclear; Eurythermal

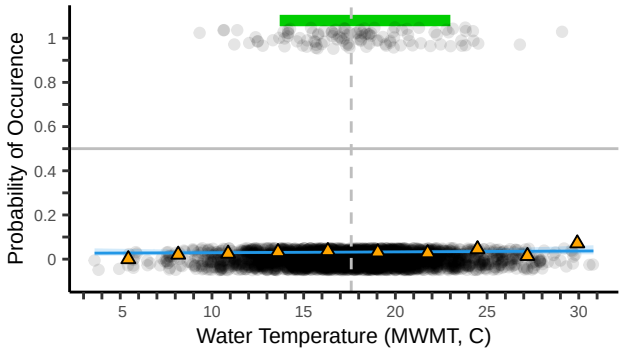

Chiro −Stempellinella
nOcc=694; WAopt=17.1; PctRange=12.8−22.5
Flat; Eurythermal

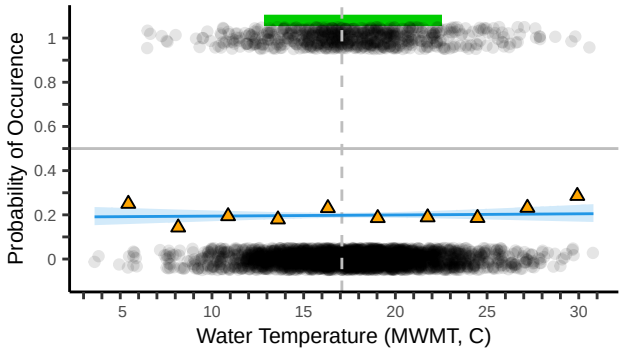

Chiro −Stictochironomus
nOcc=79; WAopt=21.5; PctRange=17.3−23.3
Unclear; Cool−Warm*

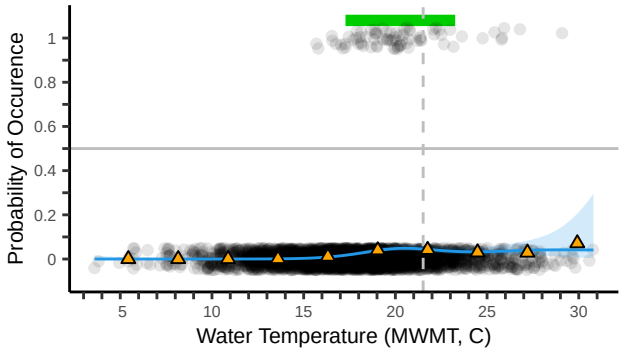

Chiro −Stilocladius
nOcc=47; WAopt=15.8; PctRange=10.7−19.6
Unclear; Cold

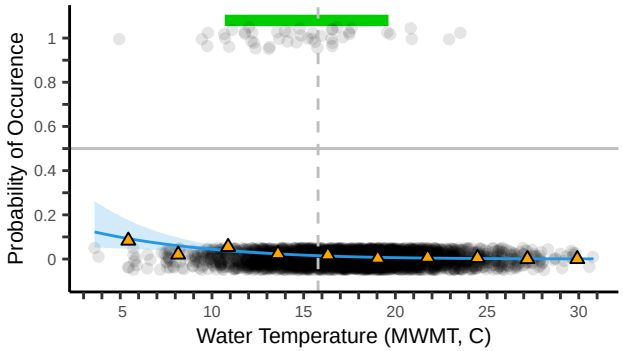

Chironomidae

Chiro − Sublettea
nOcc=73; WAopt=20.4; PctRange=17.2−23.5
Unclear; Warm

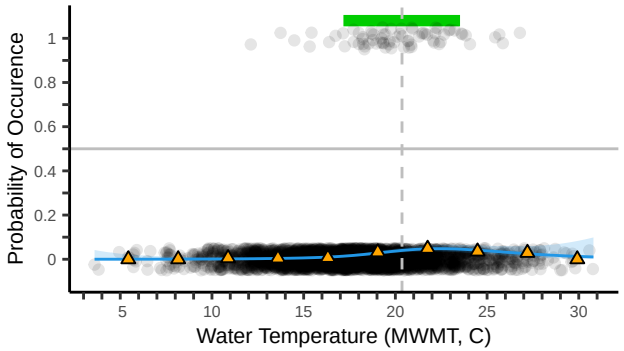

Chiro − Synorthocladius
nOcc=312; WAopt=18.7; PctRange=15.0−22.5
Unclear; Cool−Warm

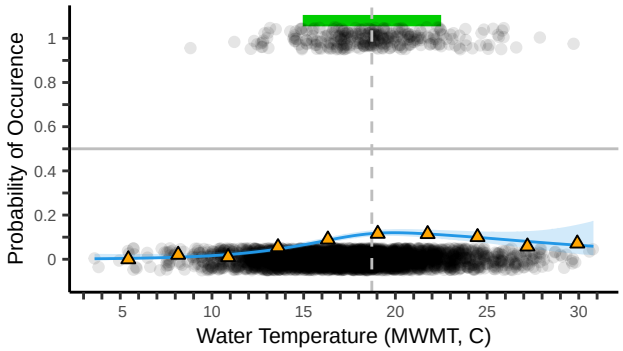

Chiro − Tanypodinae
nOcc=2,474; WAopt=18.9; PctRange=13.7−22.9
Increaser*; Eurythermal

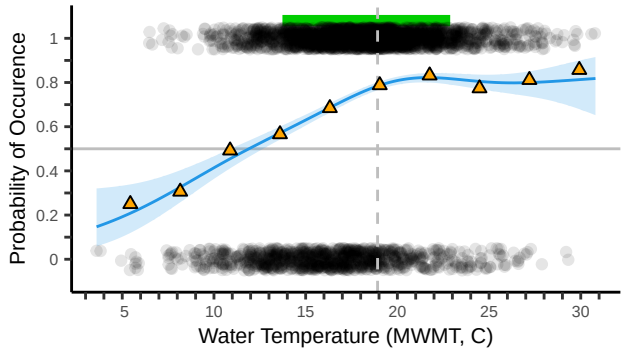

Chiro − Tanytarsini
nOcc=2,293; WAopt=18.5; PctRange=13.9−23.1
Increaser; Eurythermal

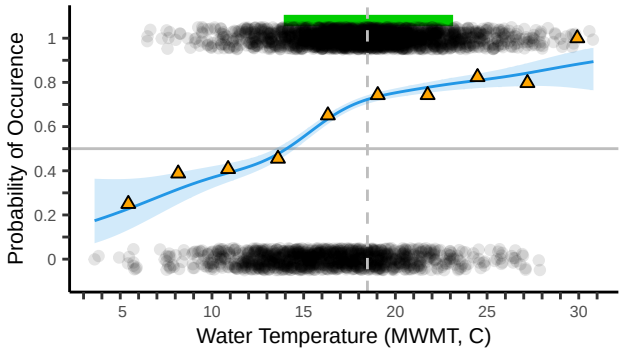

Chiro − Tanytarsus
nOcc=696; WAopt=19.8; PctRange=15.7−24.2
Increaser; Warm

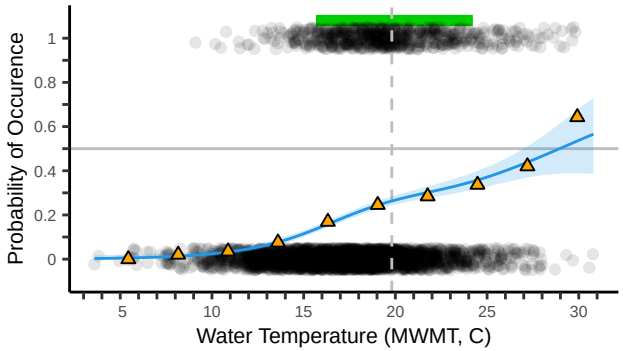

Chiro − Thienemanniella
nOcc=716; WAopt=19.0; PctRange=13.8−23.8
Increaser*; Eurythermal

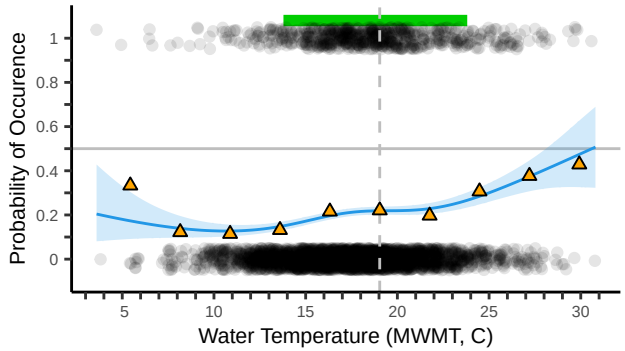

Chiro − Thienemannimyia group
nOcc=1,057; WAopt=20.0; PctRange=15.5−23.7
Increaser; Warm

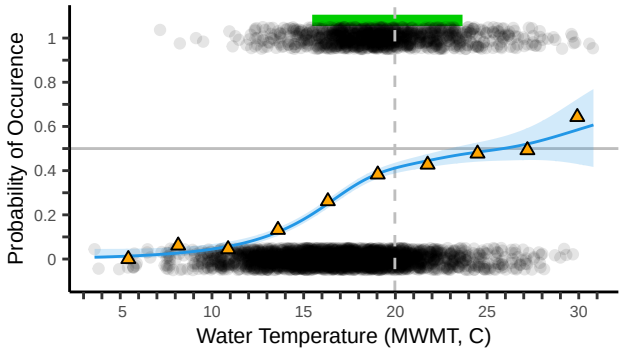

Chiro − Tribelos
nOcc=42; WAopt=19.4; PctRange=16.3−24.6
Unclear; Warm

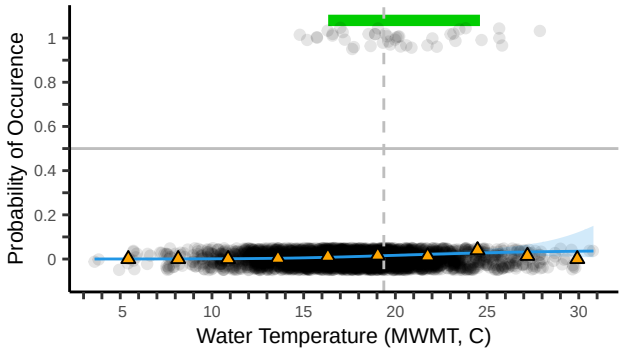

Chiro − Tvetenia
nOcc=1,297; WAopt=17.3; PctRange=13.1−21.4
Unimodal/Decreaser; Cool

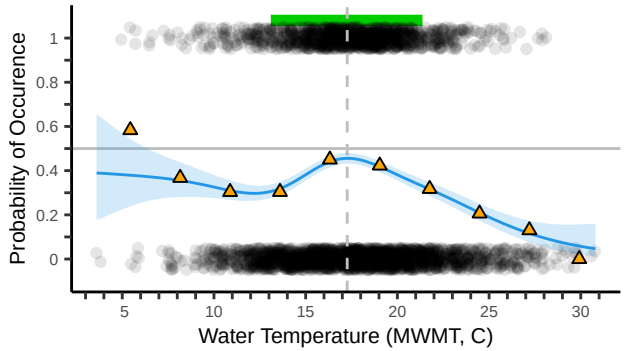

Chironomidae

Chiro −Tvetenia bavarica group
nOcc=1,149; WAopt=17.5; PctRange=13.2−21.4
Unimodal/Decreaser; Cool

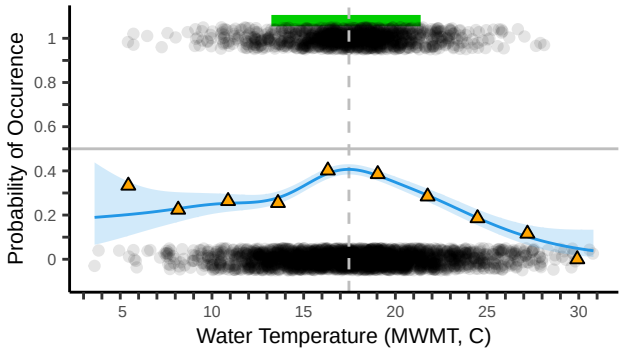

Chiro −Zavrelimyia
nOcc=636; WAopt=17.8; PctRange=13.8−21.4
Unimodal; Cool

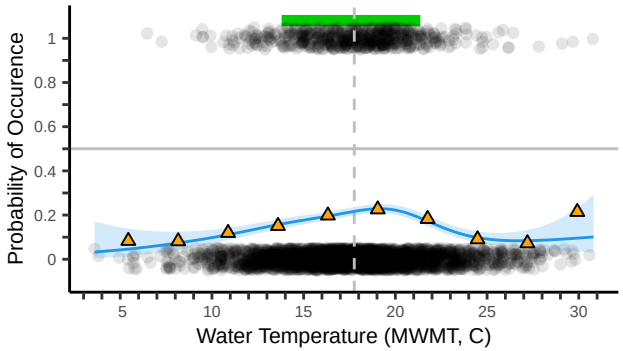

Supplement: Supplement17 [file NIHMS2055599-supplement-Supplement17.pdf]
