# Supplementary material for: Improved thermal preferences and a stressor index derived from modeled stream temperatures and regional taxonomic standards for freshwater macroinvertebrates of the Pacific Northwest, USA
Source: Ecol Indic. Author manuscript; Available in PMC 2025 Apr 9. (PMC11980781; doi:10.1016/j.ecolind.2024.111869)
Supplement: Supplement18 [file NIHMS2055599-supplement-Supplement18.pdf]

# Plecoptera

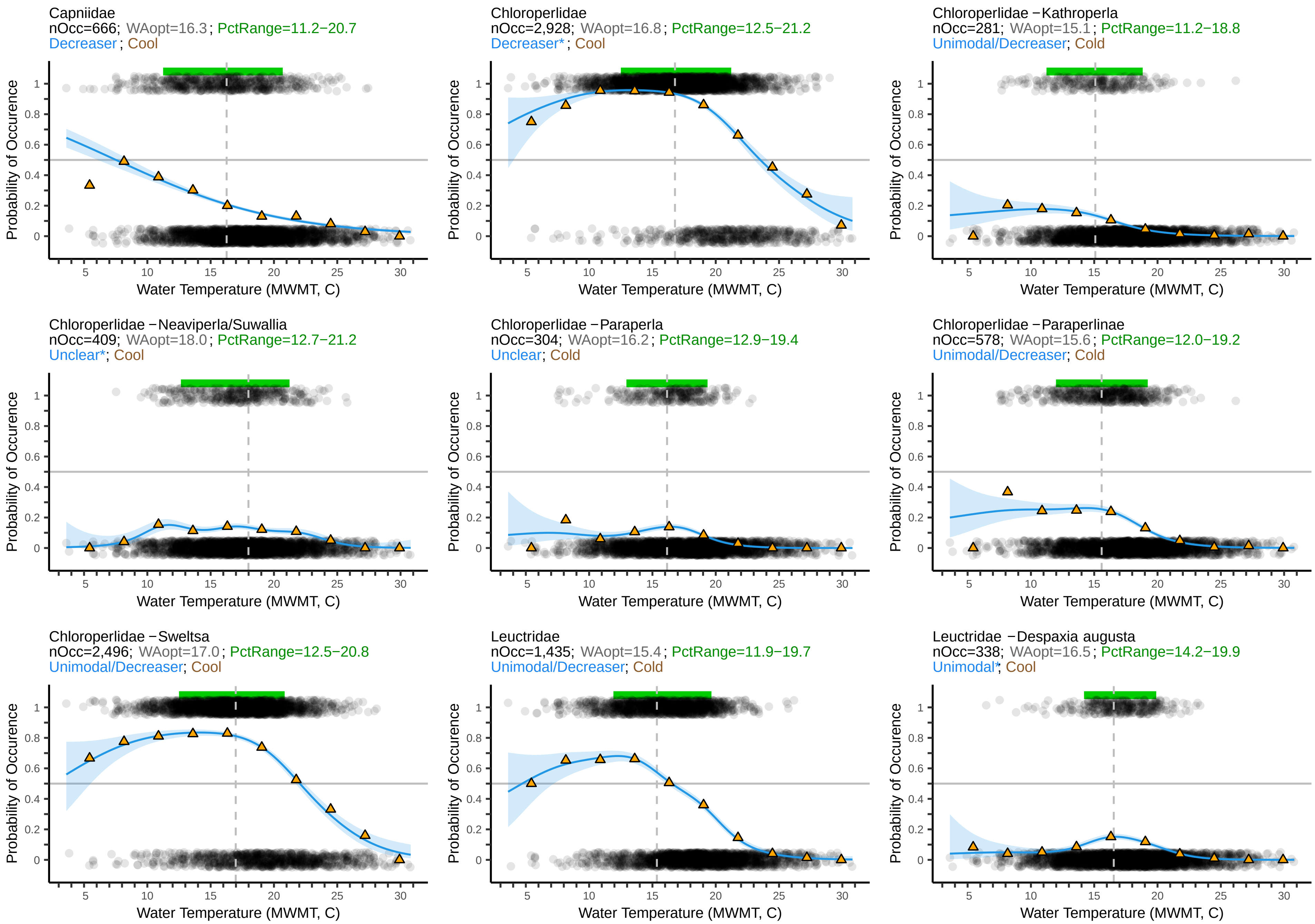

Capniidae
nOcc=666; WAopt=16.3; PctRange=11.2−20.7
Decreaser; Cool
Chloroperlidae
nOcc=2,928; WAopt=16.8; PctRange=12.5−21.2
Decreaser*; Cool
Chloroperlidae −Kathroperla
nOcc=281; WAopt=15.1; PctRange=11.2−18.8
Unimodal/Decreaser; Cold
Chloroperlidae −Neaviperla/Suwallia
nOcc=409; WAopt=18.0; PctRange=12.7−21.2
Unclear*; Cool
Chloroperlidae −Paraperla
nOcc=304; WAopt=16.2; PctRange=12.9−19.4
Unclear; Cold
Chloroperlidae −Paraperlinae
nOcc=578; WAopt=15.6; PctRange=12.0−19.2
Unimodal/Decreaser; Cold
Chloroperlidae −Sweltsa
nOcc=2,496; WAopt=17.0; PctRange=12.5−20.8
Unimodal/Decreaser; Cool
Leuctridae
nOcc=1,435; WAopt=15.4; PctRange=11.9−19.7
Unimodal/Decreaser; Cold
Leuctridae −Despaxia augusta
nOcc=338; WAopt=16.5; PctRange=14.2−19.9
Unimodal*; Cool
Probability of Occurence
0
0.2
0.4
0.6
0.8
1
Water Temperature (MWMT, C)
5
10
15
20
25
30

# Plecoptera

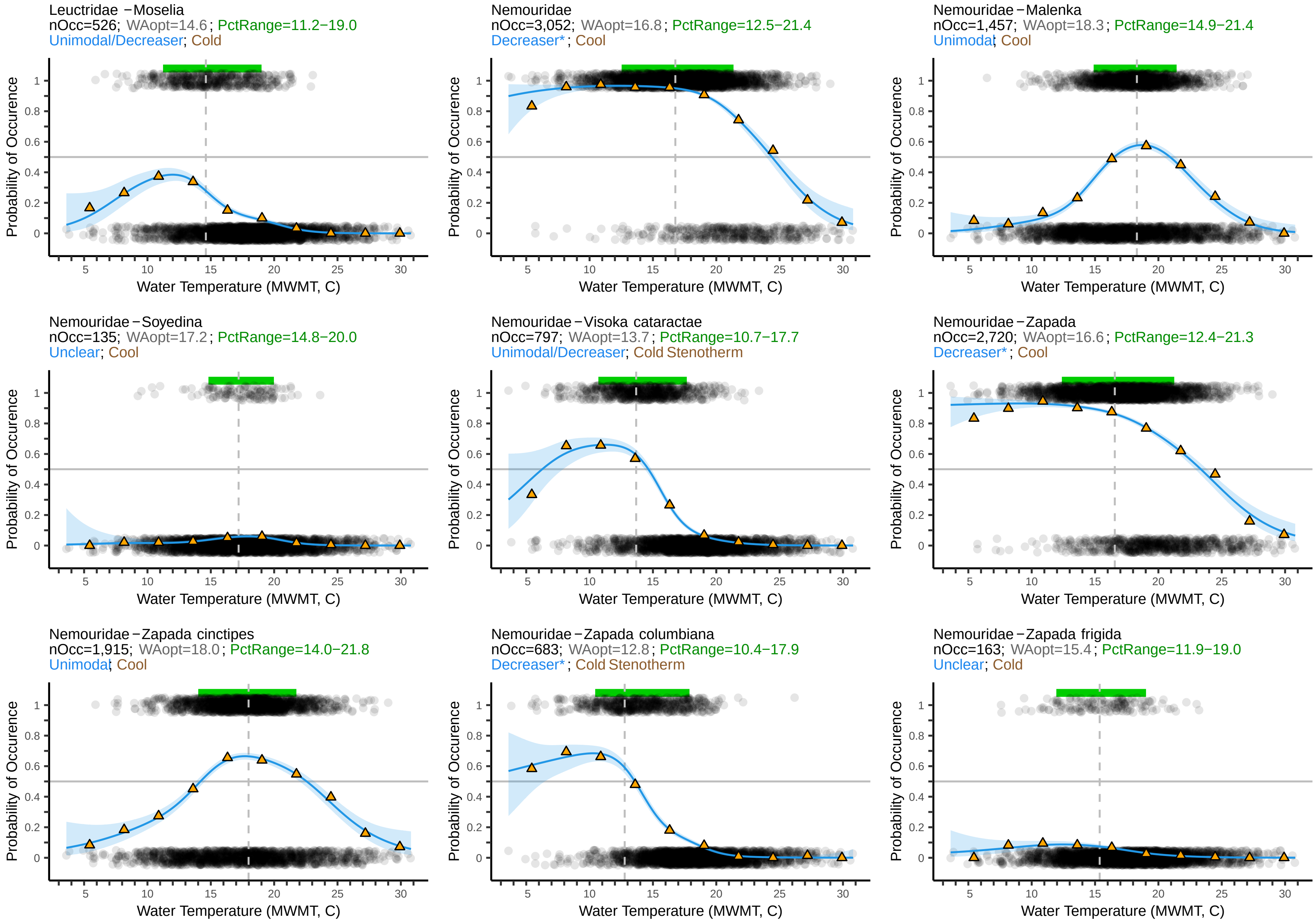

Leuctridae −Moselia
nOcc=526; WAopt=14.6; PctRange=11.2−19.0
Unimodal/Decreaser; Cold
Nemouridae
nOcc=3,052; WAopt=16.8; PctRange=12.5−21.4
Decreaser*; Cool
Nemouridae−Malenka
nOcc=1,457; WAopt=18.3; PctRange=14.9−21.4
Unimodal; Cool
Nemouridae−Soyedina
nOcc=135; WAopt=17.2; PctRange=14.8−20.0
Unclear; Cool
Nemouridae−Visoka cataractae
nOcc=797; WAopt=13.7; PctRange=10.7−17.7
Unimodal/Decreaser; Cold Stenotherm
Nemouridae−Zapada
nOcc=2,720; WAopt=16.6; PctRange=12.4−21.3
Decreaser*; Cool
Nemouridae−Zapada cinctipes
nOcc=1,915; WAopt=18.0; PctRange=14.0−21.8
Unimodal; Cool
Nemouridae−Zapada columbiana
nOcc=683; WAopt=12.8; PctRange=10.4−17.9
Decreaser*; Cold Stenotherm
Nemouridae−Zapada frigida
nOcc=163; WAopt=15.4; PctRange=11.9−19.0
Unclear; Cold
Probability of Occurence
Water Temperature (MWMT, C)

# Plecoptera

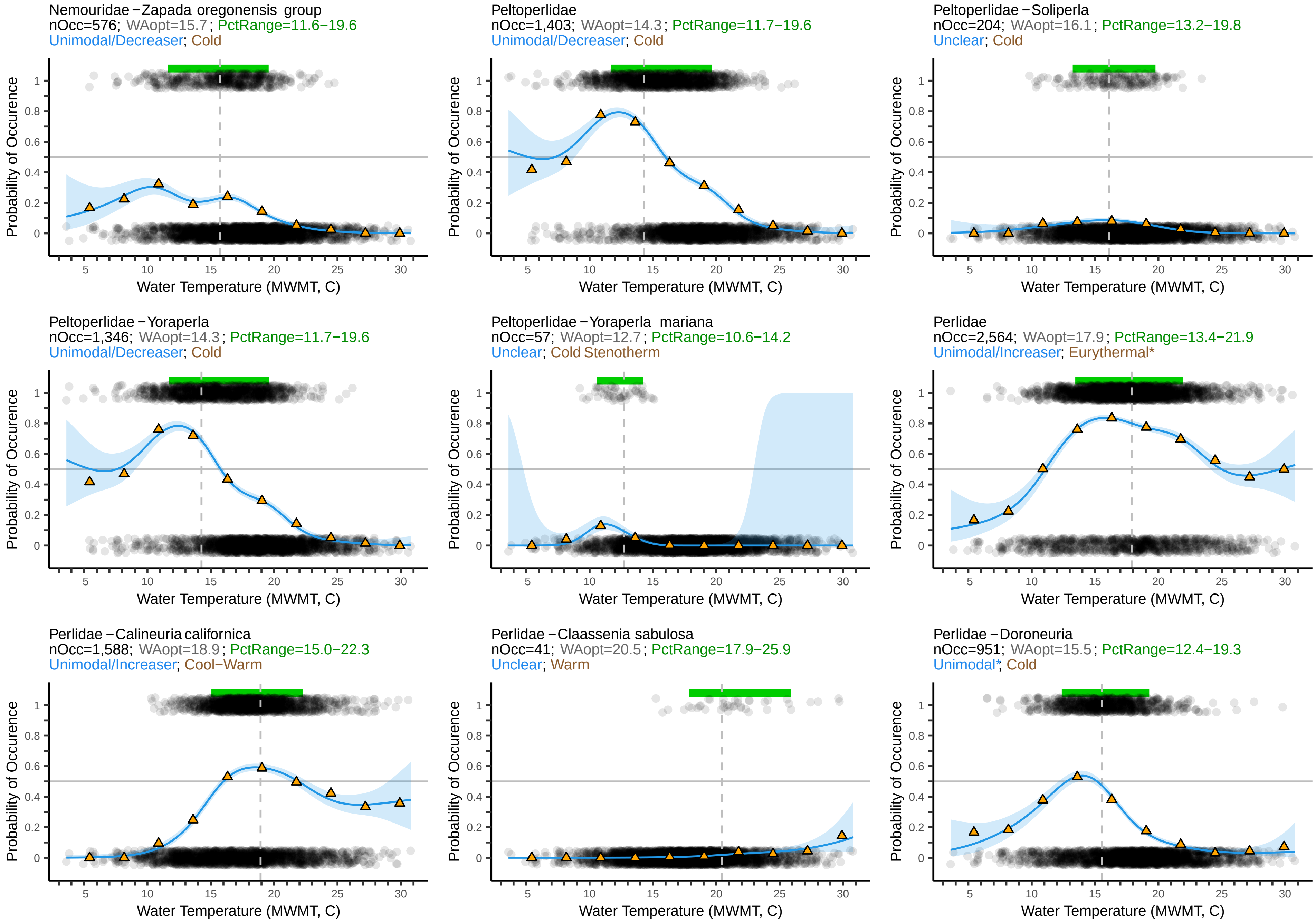
Nemouridae − Zapada oregonensis group
nOcc=576; WAopt=15.7; PctRange=11.6−19.6
Unimodal/Decreaser; Cold
Peltoperlidae
nOcc=1,403; WAopt=14.3; PctRange=11.7−19.6
Unimodal/Decreaser; Cold
Peltoperlidae − Soliperla
nOcc=204; WAopt=16.1; PctRange=13.2−19.8
Unclear; Cold
Peltoperlidae − Yoraperla
nOcc=1,346; WAopt=14.3; PctRange=11.7−19.6
Unimodal/Decreaser; Cold
Peltoperlidae − Yoraperla mariana
nOcc=57; WAopt=12.7; PctRange=10.6−14.2
Unclear; Cold Stenotherm
Perlidae
nOcc=2,564; WAopt=17.9; PctRange=13.4−21.9
Unimodal/Increaser; Eurythermal*
Perlidae − Calineuria californica
nOcc=1,588; WAopt=18.9; PctRange=15.0−22.3
Unimodal/Increaser; Cool−Warm
Perlidae − Claassenia sabulosa
nOcc=41; WAopt=20.5; PctRange=17.9−25.9
Unclear; Warm
Perlidae − Doroneuria
nOcc=951; WAopt=15.5; PctRange=12.4−19.3
Unimodal*; Cold
Probability of Occurence
Water Temperature (MWMT, C)

# Plecoptera

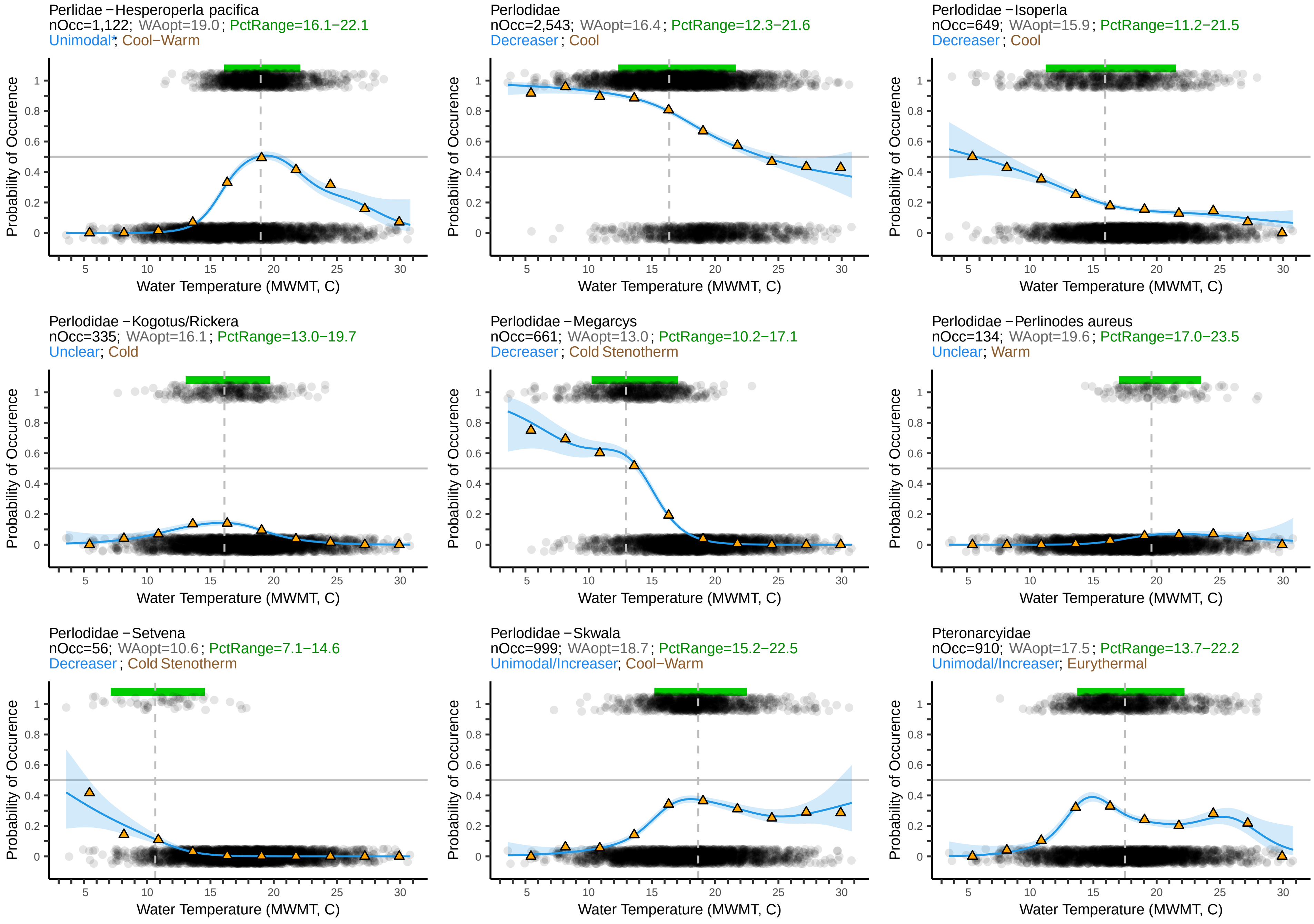
Perlidae −Hesperoperla pacifica
nOcc=1,122; WAopt=19.0; PctRange=16.1−22.1
Unimodal*; Cool−Warm
Perlodidae
nOcc=2,543; WAopt=16.4; PctRange=12.3−21.6
Decreaser; Cool
Perlodidae −Isoperla
nOcc=649; WAopt=15.9; PctRange=11.2−21.5
Decreaser; Cool
Perlodidae −Kogotus/Rickera
nOcc=335; WAopt=16.1; PctRange=13.0−19.7
Unclear; Cold
Perlodidae −Megarcys
nOcc=661; WAopt=13.0; PctRange=10.2−17.1
Decreaser; Cold Stenotherm
Perlodidae −Perlinodes aureus
nOcc=134; WAopt=19.6; PctRange=17.0−23.5
Unclear; Warm
Perlodidae −Setvena
nOcc=56; WAopt=10.6; PctRange=7.1−14.6
Decreaser; Cold Stenotherm
Perlodidae −Skwala
nOcc=999; WAopt=18.7; PctRange=15.2−22.5
Unimodal/Increaser; Cool−Warm
Pteronarcyidae
nOcc=910; WAopt=17.5; PctRange=13.7−22.2
Unimodal/Increaser; Eurythermal
Probability of Occurence
Water Temperature (MWMT, C)

# Plecoptera

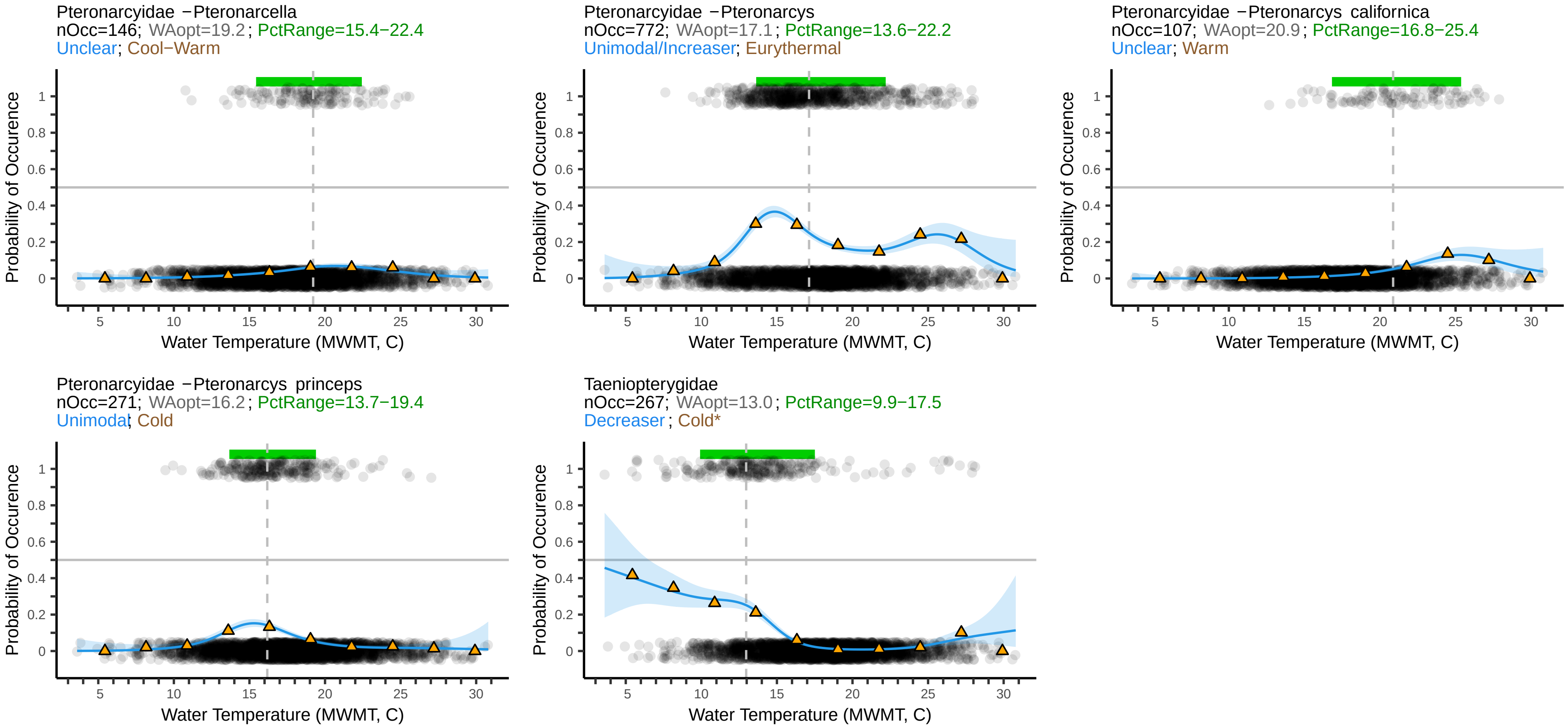

Pteronarcyidae −Pteronarcella
nOcc=146; WAopt=19.2; PctRange=15.4−22.4
Unclear; Cool−Warm
Pteronarcyidae −Pteronarcys
nOcc=772; WAopt=17.1; PctRange=13.6−22.2
Unimodal/Increaser; Eurythermal
Pteronarcyidae −Pteronarcys californica
nOcc=107; WAopt=20.9; PctRange=16.8−25.4
Unclear; Warm
Pteronarcyidae −Pteronarcys princeps
nOcc=271; WAopt=16.2; PctRange=13.7−19.4
Unimodal; Cold
Taeniopterygidae
nOcc=267; WAopt=13.0; PctRange=9.9−17.5
Decreaser; Cold*
Probability of Occurence
0
0.2
0.4
0.6
0.8
1
5
10
15
20
25
30
Water Temperature (MWMT, C)
