# Supplementary material for: Improved thermal preferences and a stressor index derived from modeled stream temperatures and regional taxonomic standards for freshwater macroinvertebrates of the Pacific Northwest, USA
Source: Ecol Indic. Author manuscript; Available in PMC 2025 Apr 9. (PMC11980781; doi:10.1016/j.ecolind.2024.111869)
Supplement: Supplement19 [file NIHMS2055599-supplement-Supplement19.pdf]

Supplement 2

GAM Plot Interpretation Guide

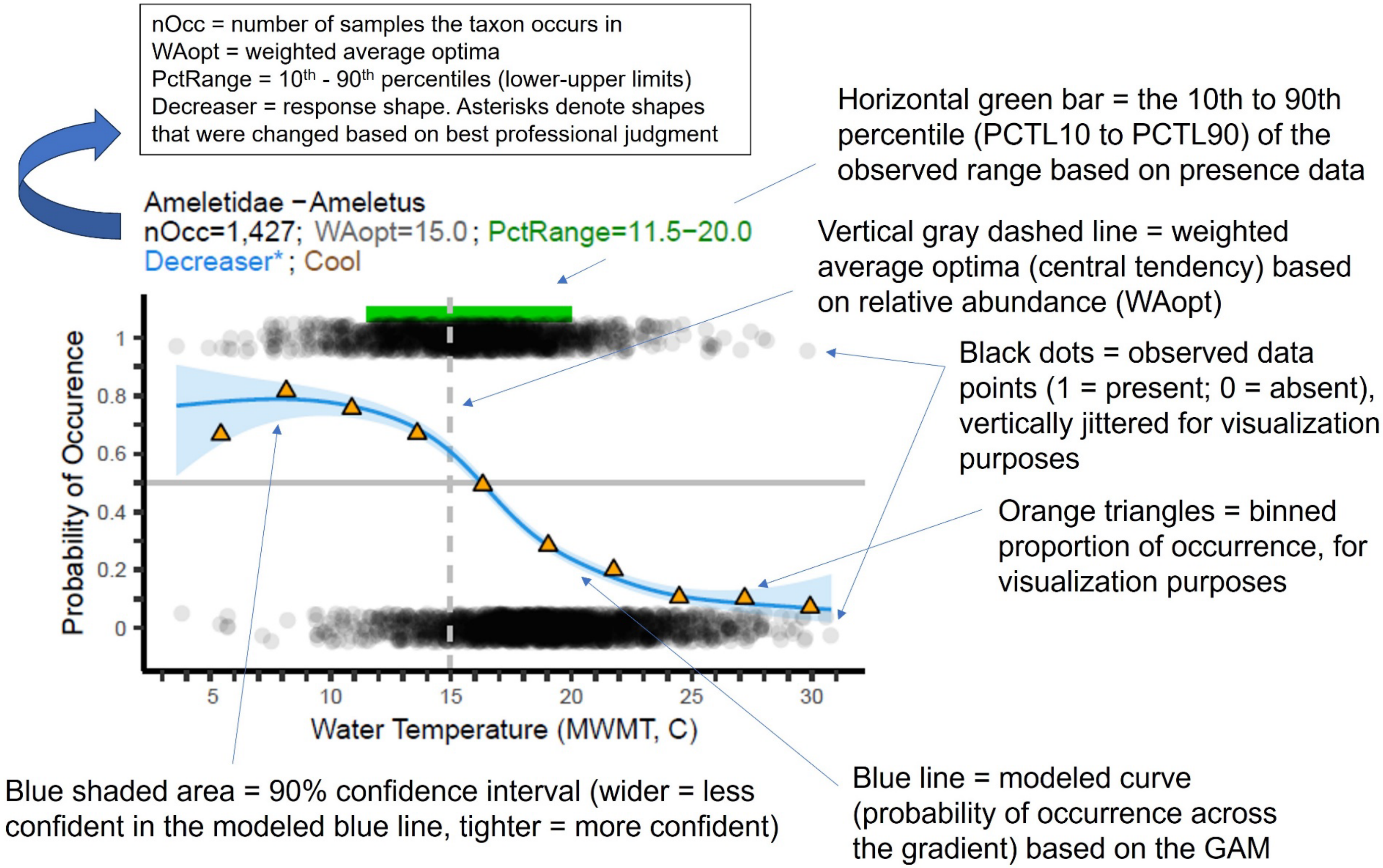

Figure S2-1. General additive model (GAM) plot interpretation guide.

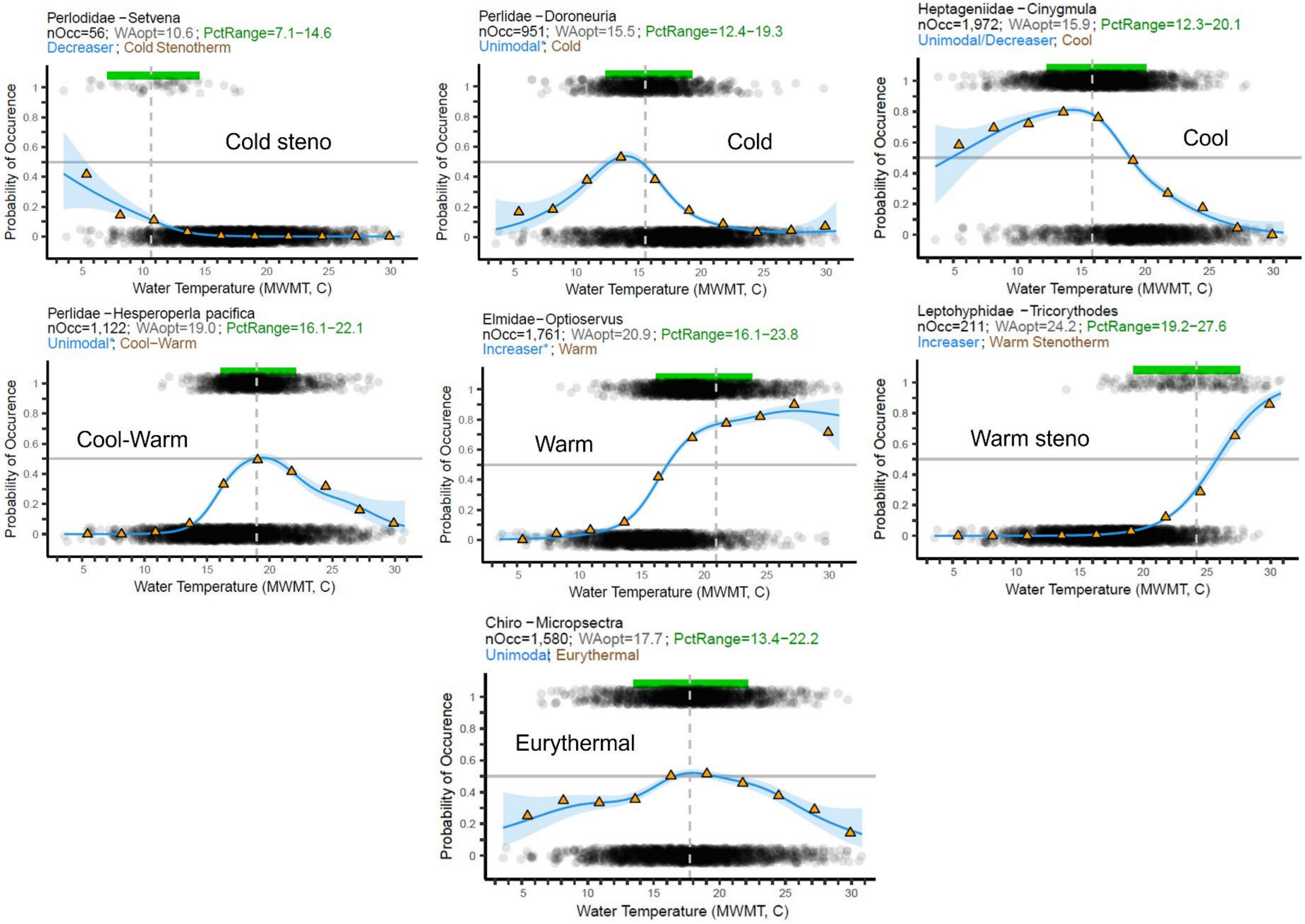

Figure S2-2. Examples of typical patterns seen in GAM plots for representative taxa from each thermal preference category.
