# Supplementary material for: Improved thermal preferences and a stressor index derived from modeled stream temperatures and regional taxonomic standards for freshwater macroinvertebrates of the Pacific Northwest, USA
Source: Ecol Indic. Author manuscript; Available in PMC 2025 Apr 9. (PMC11980781; doi:10.1016/j.ecolind.2024.111869)
Supplement: Supplement20 [file NIHMS2055599-supplement-Supplement20.docx]

Supplement 3

**R scripts for calculating the MTTI and thermal preference metrics and**

**MACROINVERTEBRATE TEMPERATURE TOLERANCE INDEX (MTTI)**

We provide an example R script (S3-2_MTTI_example_20230907.R) for calculating the MTTI. It uses several different packages, including rioja, and includes a test file. The files are copied to the tempdir() as the working directory.

An internet connection is needed because the R code references two tables that are posted on GitHub:

- **ORWA_TaxaTranslator** – this converts original taxon names to the Operational Taxonomic Unit (OTU) that should be used for the MTTI calculation
- **ORWA_Attribute** – this assigns the correct WA optima values to the OTUs

GitHub link: <https://github.com/leppott/BioMonTools_SupportFiles/tree/main/data/taxa_official>

It is important that the proper OTUs and WA optima values be used for the MTTI calculation.

There are three required fields in the input file:

- SampleID (unique sample identifier. Includes any replicate/duplicate information)
- TaxaID (unique taxa name)
- Count of individuals

Primary developer of the OR/WA MTTI R code: Shannon Hubler ([Shannon.L.HUBLER@deq.oregon.gov](mailto:Shannon.L.HUBLER@deq.oregon.gov))

**THERMAL PREFERENCE METRICS**

We provide an example R script (S3-3_ThermMetricCalc_20240208.R), which uses the BioMonTools R package to calculate thermal preference metrics, using the **Thermal_indicator** categories in Appendix B.

R package: <https://github.com/leppott/BioMonTools>

The thermal preference metric calculator calculates three types of metrics (number of taxa, % taxa and % individuals) for seven thermal preference categories:

- Cold stenotherm
- Cold
- Cool
- Cool/warm
- Warm
- Warm stenotherm
- Eurythermal

The R code requires the input file to be formatted a particular way, columns to be labeled a specific way and thermal preference categories to be entered a certain way (Table 1).

When creating your input file, use the thermal preference entries from the **Thermal_indicator** column in Appendix B, which are as follows:

- stenoc (this is the abbreviation for cold stenotherm)
- cold
- cool
- cowa (this is the abbreviation for cool/warm)
- warm
- stenow (this is the abbreviation for warm stenotherm)
- eurythermal

Table 2 has translations of the abbreviated metric names in the output file.

Primary developer of the BioMonTools R package: Erik W. Leppo ([Erik.Leppo@tetratech.com](mailto:Erik.Leppo@tetratech.com))

More metrics (beyond thermal preference) are available in the BioMonTools R package. For more information, visit the BioMonTools GitHub page (<https://github.com/leppott/BioMonTools>**).**

Table S3-1. Input file requirements

| **Column Name** | **Data Type** | **Allowable entries** | **Description** |
| --- | --- | --- | --- |
| SAMPLEID | text |  | Unique sample identifier (typically comprised of the site name, sample date, and replicate number). |
| TAXAID | text |  | Taxonomic identification of organisms in each sample. |
| N_TAXA | numeric | non-negative real number | Number of individuals. |
| NONTARGET | text | TRUE, FALSE | Non-target taxa should be marked "TRUE". The R code excludes non-target taxa from all metric calculations. |
| EXCLUDE | text | TRUE, FALSE | Redundant (non-distinct) taxa should marked "TRUE". Redundant taxa are excluded from richness metric calculations but are included in the composition (percent individuals) metrics. |
| THERMAL_INDICATOR | text | stenoc, cold, cool, cowa, warm, stenow, eurythermal | Cold stenotherm =stenoc; cold=cold; cool=cool; cool/warm=cowa; warm=warm; warm stenotherm=stenow; eurythermal=eurythermal |

Table S3-2. ‘Translations’ of the abbreviated metric names in the output file.

| **Metric_abbrev** | **Full metric description** |
| --- | --- |
| ni_total | # total individuals |
| nt_total | # total taxa |
| nt_ti_stenocold | # cold stenotherm taxa |
| nt_ti_cold | # cold taxa |
| nt_ti_cool | # cool taxa |
| nt_ti_warm | # warm taxa |
| nt_ti_stenowarm | # warm stenotherm taxa |
| nt_ti_eury | # eurythermal taxa |
| nt_ti_cowa | # cool/warm taxa |
| nt_ti_na | # taxa no assignment |
| nt_ti_stenocold_cold | # cold stenotherm + cold taxa |
| nt_ti_stenocold_cold_cool | # cold stenotherm + cold + cool taxa |
| nt_ti_cowa_warm_stenowarm | # cool/warm + warm + warm stenotherm taxa |
| nt_ti_warm_stenowarm | # warm + warm stenotherm taxa |
| pi_ti_stenocold | % cold stenotherm indiv |
| pi_ti_cold | % cold indiv |
| pi_ti_cool | % cool indiv |
| pi_ti_warm | % warm indiv |
| pi_ti_stenowarm | % warm stenotherm indiv |
| pi_ti_eury | % eurythermal indiv |
| pi_ti_cowa | % cool/warm indiv |
| pi_ti_na | % indiv no assignment |
| pi_ti_stenocold_cold | % cold stenotherm + cold indiv |
| pi_ti_stenocold_cold_cool | % cold stenotherm + cold + cool indiv |
| pi_ti_cowa_warm_stenowarm | % cool/warm + warm + warm stenotherm indiv |
| pi_ti_warm_stenowarm | % warm + warm stenotherm indiv |
| pt_ti_stenocold | % cold stenotherm taxa |
| pt_ti_cold | % cold taxa |
| pt_ti_cool | % cool taxa |
| pt_ti_warm | % warm taxa |
| pt_ti_stenowarm | % warm stenotherm taxa |
| pt_ti_eury | % eurythermal taxa |
| pt_ti_cowa | % cool/warm taxa |
| pt_ti_na | % taxa no assignment |
| pt_ti_stenocold_cold | % cold stenotherm + cold taxa |
| pt_ti_stenocold_cold_cool | % cold stenotherm + cold + cool taxa |
| pt_ti_cowa_warm_stenowarm | % cool/warm + warm + warm stenotherm taxa |
| pt_ti_warm_stenowarm | % warm + warm stenotherm taxa |
